# Comparative Methylome Analysis of the Occasional Ruminant Respiratory Pathogen *Bibersteinia trehalosi*

**DOI:** 10.1371/journal.pone.0161499

**Published:** 2016-08-24

**Authors:** Brian P. Anton, Gregory P. Harhay, Timothy P. L. Smith, Jochen Blom, Richard J. Roberts

**Affiliations:** 1 New England Biolabs, Ipswich, Massachusetts, United States of America; 2 USDA-ARS U.S. Meat Animal Research Center, Clay Center, Nebraska, United States of America; 3 Bioinformatics and Systems Biology, Justus-Liebig-University Giessen, Giessen, Germany; University of Edinburgh, UNITED KINGDOM

## Abstract

We examined and compared both the methylomes and the modification-related gene content of four sequenced strains of *Bibersteinia trehalosi* isolated from the nasopharyngeal tracts of Nebraska cattle with symptoms of bovine respiratory disease complex. The methylation patterns and the encoded DNA methyltransferase (MTase) gene sets were different between each strain, with the only common pattern being that of Dam (GATC). Among the observed patterns were three novel motifs attributable to Type I restriction-modification systems. In some cases the differences in methylation patterns corresponded to the gain or loss of MTase genes, or to recombination at target recognition domains that resulted in changes of enzyme specificity. However, in other cases the differences could be attributed to differential expression of the same MTase gene across strains. The most obvious regulatory mechanism responsible for these differences was slipped strand mispairing within short sequence repeat regions. The combined action of these evolutionary forces allows for alteration of different parts of the methylome at different time scales. We hypothesize that pleiotropic transcriptional modulation resulting from the observed methylomic changes may be involved with the switch between the commensal and pathogenic states of this common member of ruminant microflora.

## Introduction

Bovine respiratory disease complex (BRDC) is a multifactorial disease caused by an array of viral and bacterial agents with contributions from environmental factors and animal stress. In North America alone, BRDC is the most common disease in feed yard cattle and is the predominant driver of antibiotic metaphylaxis to maintain cattle health [1–4]. The most severe manifestations of the disease appear to involve immune suppression by viral infection or stress, followed by bacterial lung infection. These bacteria include the opportunistic pathogens *Bibersteinia trehalosi* and *Mannheimia haemolytica*, which are found inhabiting the upper respiratory tract of apparently healthy cattle as commensals. In the lungs these bacteria secrete leukotoxins, which lyse leukocytes causing fibronecrotic lung lesions, manifesting as pneumonia with variable morbidity and mortality. These bacterial species are also associated with respiratory disease in wild bighorn sheep. Some strains of *B*. *trehalosi* and *M*. *haemolytica* are more aggressive and communicable than others, and these aggressive strains are responsible for increased disease severity and economic losses and a reduction in animal well-being. In order to improve animal well-being, reduce antibiotic use, and reduce financial losses, developing improved BRDC mitigation strategies is critical.

Arresting the development of virulent populations of *B*. *trehalosi*, *M*. *haemolytica*, and similar bovine respiratory pathogens would likely be an effective means to control BRDC. However, it is not understood how populations of these opportunistic pathogenic bacteria convert into virulent populations in the lungs. Therefore, we embarked on a combined genomic and epigenomic study of these bacteria to shed light on these conversion mechanisms.

Slipped-strand mispairing (SSM) is a mechanism that generates genetically heterogeneous populations of bacteria by DNA polymerase slippage at hypermutable sites called simple sequence repeats (SSRs) [5]. This diversity may enable the population to adapt to changing host environments. SSM has been observed in the human pathogens *Haemophilus influenzae* and *Neisseria gonorrhoeae* [6], and its role in virulence demonstrated in *Bordetella pertussis* [7], *Campylobacter jejuni* [8] and *Streptococcus pneumonia*e [9].

In the case of restriction-modification (R-M) systems, SSRs have been observed at or near the 5’ end of methyltransferase (MTase) genes, with the number of repeats governing whether the coding sequence is in or out of frame with the start codon; examples include the Type III MTase M.MmyCI [10] and the Type I MTase M.PhaAI [11]. This phasing acts as a MTase on/off switch, which in turn alters the overall genomic methylation pattern, referred to as the methylome. Alteration of methylation patterns has been associated with virulence in *Salmonella enterica* [12], *Neisseria gonorrhoeae* [13], *Edwardsiella tarda* [14], and other bacteria [15].

Besides SSM, R-M systems evolve relatively rapidly by other methods including homologous recombination [16], gene conversion, and site-specific inversion [17], often coupled with horizontal gene transfer [18]. Furthermore, target recognition domains (TRDs) of Type I, Type III, and some Type IIG R-M systems have been shown to move between and within loci, thereby generating allelic diversity and altering the recognition sequences of MTases and REases [17, 19, 20]. In the case of Type I specificity proteins, which typically contain two TRDs, this can occur not only by replacement of one or both TRDs, but also by loss of one [21].

Single-Molecule Real-Time (SMRT) sequencing, developed by Pacific Biosciences, has greatly facilitated the study of bacterial DNA methylation *in situ*, and the methylome (the methylation pattern that results from the combined action of the DNA MTases active in a cell) has become an increasingly studied topic [12, 20, 22–24]. In the present study, we have characterized the methylomes and the related gene content of four strains of *B*. *trehalosi* previously sequenced using the SMRT platform [25, 26]. These strains have some methylation patterns in common as well as others restricted to one or a subset of the strains. In some cases, the genome sequences provide clues as to how these differences have arisen, including evidence for SSM and TRD exchange.

## Materials and Methods

### Growth and genomic DNA isolation of *B*. *trehalosi* strains

Cultures were grown in 10 mL BHI broth at 37°C for 20 hours without agitation, and cells were collected by centrifugation. DNA was extracted using Genomic-tip 100/G columns and buffers (Qiagen; Valencia, CA) as directed by the manufacturer with some modifications. Specifically, cells were resuspended in 5 mL Qiagen buffer B1 with 50 μg/mL RNase A and 250 μL 0.5 M EDTA by vortexing. The cells were incubated at 70°C for 10 minutes and vortexed again. The solution was equilibrated at 37°C, and 100 μL fresh 100 mg/mL lysozyme (Sigma-Aldrich; St. Louis, MO) was added and mixed by brief vortexing. The mixture was incubated for 10 minutes, then 150 μL proteinase K (20 mg/mL) was added and the solution incubated at 37°C until clearing of the supernatant occurred (30 minutes to 2 hours). Once the lysate cleared, 1.7 mL of Qiagen buffer B2 was added and mixed by vortexing, followed by 30 minute incubation at 50°C, and then diluted by addition of 6.7 mL Qiagen buffer QBT and additional brief vortexing. This mixture was added to the equilibrated 100/G column, avoiding any foam that accrued, until the entire mixture passed through the column. The column was washed 2x with 10 mL Qiagen buffer QC, and DNA was eluted with 7 mL of Qiagen buffer QF at 50°C and precipitated by addition of 4.9 mL 100% isopropanol and gentle inversion. The DNA precipitate was removed from the solution with a glass hook, and dipped consecutively into 2 mL 70% ethanol and 2 mL 95% ethanol. The washed pellet was dried briefly and dislodged from the hook into 250 μL TE (0.1 mM EDTA, 10 mM Tris pH 7.9) and allowed to dissolve at room temperature overnight.

### Cloning of genes and host genomic DNA isolation

To unambiguously assign methylation recognition sequences to MTase and specificity genes, candidate genes were subcloned into the high copy vector pRRS [27], and the methylation of the heterologous *E*. *coli* host DNA was observed. Genes were amplified from *B*. *trehalosi* gDNA templates by PCR using Phusion or Q5 DNA polymerase (New England Biolabs; Ipswich, MA). Primers used for amplification, and the genes amplified, are shown in S1 Table. Genes were subcloned using one of three methods: (1) digestion of pRRS vector and PCR products with PstI-HF or SbfI-HF on one end and BamHI-HF on the other, followed by treatment with Proteinase K, purification using the QIAQuick PCR Purification Kit (Qiagen; Valencia, CA), and ligation with T4 DNA ligase; (2) Gibson assembly of PCR-amplified genes with pRRS vector digested with NdeI and BamHI; or (3) Gibson assembly of PCR-amplified genes with pRRS100 vector digested with HpaI and BamHI-HF. Construction steps were performed in *E*. *coli* ER2683, and completed constructs were then passaged through the methylation-deficient strain *E*. *coli* ER2796 [28] before sequencing.

Plasmid DNA was purified using the GenElute HP Plasmid Miniprep Kit (Sigma-Aldrich; St. Louis, MO). Total DNA was purified from 50 mL of overnight bacterial culture as follows, with all mixing steps performed by gentle inversion. The pellet from 50 mL of culture was resuspended in 5 mL [25% sucrose, 50 mM Tris pH 8.0, 1 mM EDTA]. To this was added 4 mL [250 mM Tris pH 8.0, 250 mM EDTA, 10 mg/mL chicken egg-white lysozyme (Sigma-Aldrich)], and the solution was incubated for 2 hrs at 37°C. To this was added 6 mL [50 mM Tris pH 8.0, 62.5 mM EDTA, 1% Triton X-100]. The resulting mixture was extracted 1x with phenol and 1x with methylene chloride. DNA was precipitated with 0.1 volumes 5 M NaCl and 0.7 volumes isopropanol. The DNA pellet was washed twice with 70% ethanol, air dried, and resuspended in 400 μL [10 mM Tris pH 8.0, 1 mM EDTA].

### Genome assembly and closure

*B*. *trehalosi* 188, 189, 190, and 192 SMRT sequencing reads were compared with 454 reads using the PBcR pipeline to ensure accuracy and assembled with the Celera Assembler as previously described [25, 26, 29]. Circularization was confirmed by overlapping the 5’- and 3’- ends of the chromosome evident in a dotplot. The overlapping 3’-end was trimmed, the origin of replication approximated using GenSkew and the chromosome base numbering re-indexed at this position to base number 1. The chromosome was re-sequenced through the RS_Resequencing pipeline to generate a final consensus. In all cases the agreement between the PacBio reads and final consensus sequence was greater the 99.9% with no difference in read coverage through the initial joined region compared to the rest of the genome. Base modifications were derived from interpulse duration (IPD) ratios using the RS_Modification_and_Motif_Analysis.1 pipeline (Pacific Biosciences; Menlo Park, CA) with the default threshold of QV = 30. The Do-It-Yourself-Annotation pipeline [30] was used to annotate the final consensus for submission to GenBank.

### Gene Ontology term annotation with BLAST2GO

Blast2GO 3.1.2 was used to compare translated coding sequences with a database of microbial proteins from the GenBank Genome archive (ftp.ncbi.nlm.nih.gov/genbank/genomes/Bacteria). A concatenated FASTA file of the hits was fed into USEARCH [31] to create another FASTA file of sequences sorted from longest to shortest, with all description sequence stripped out. This sorted file was clustered with USEARCH using the cluster_fast option at the 90% level of identity to create a reduced FASTA file. A perl script was used to transfer the sequence descriptions from the concatenated FASTA file to the sequences in the clustered FASTA file and it was this file that was used to create a local BLAST database. Gene Ontology (GO) terms were mapped to BLAST hits using the Blast2GO mapper followed by annotation of the CDS with GO terms inferred from their BLAST hits. Lists of CDS were assessed for GO enrichment relative to a genomic reference using the BLAST2GO Fisher Exact Test (two-tailed) with multiple test correction [32].

### BLASTX Search for Proteins with an N-Terminal SSR

CDS with upstream SSR were screened against either the NCBI non-redundant protein database or the microbial protein database created for our BLAST2GO analyses. A 750 bp contiguous region consisting of the SSR and the 5’-end of the CDS was extracted and screened against these local databases with BLASTX using the BLOSUM45 matrix and a maximum e-value of 0.01.

## Results

### Genome Features and Gene Content

**Fig 1:**
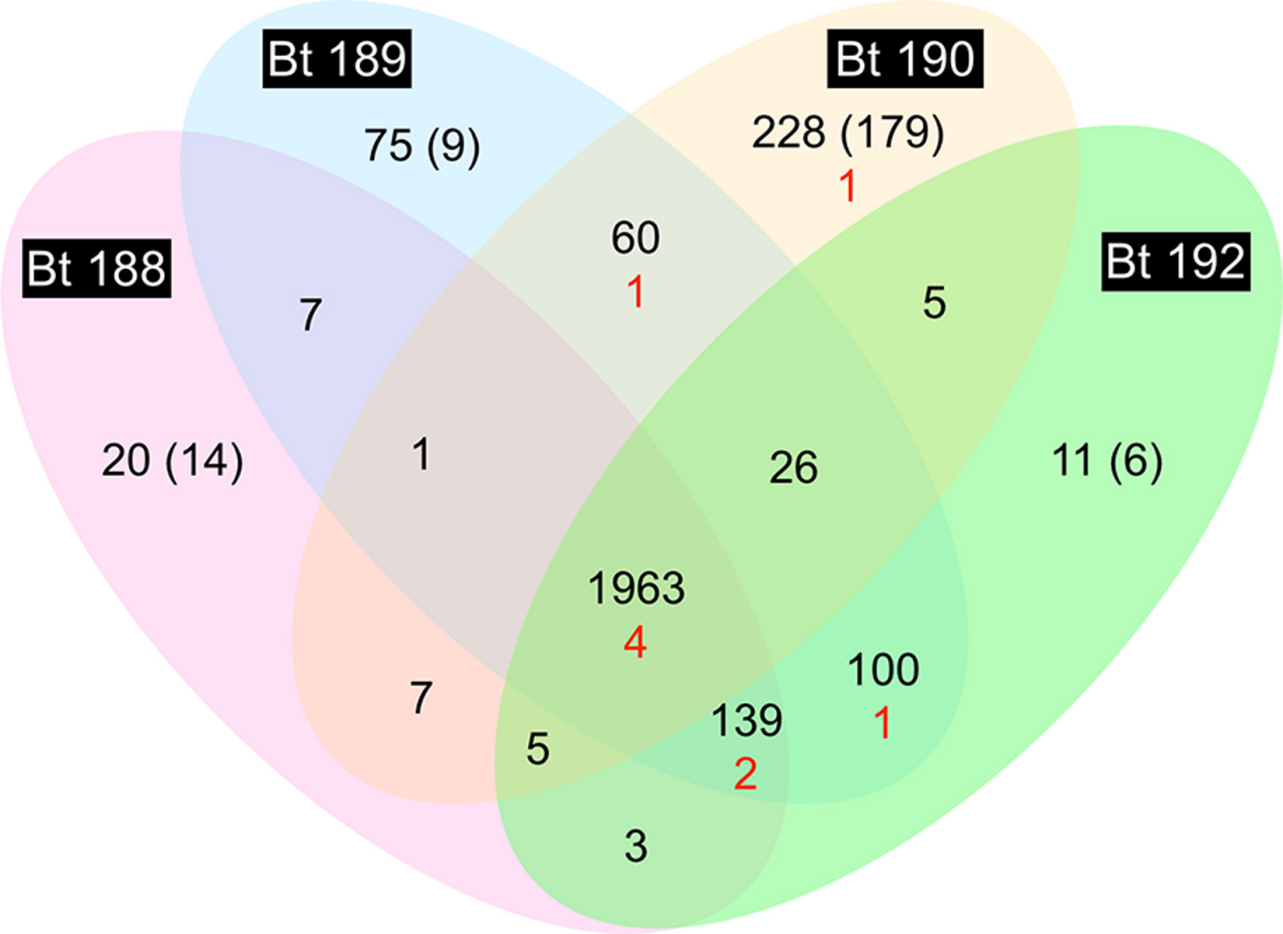
Orthologs in the four *B*. *trehalosi* isolates. The pan genome as determined by EDGAR, consisting of 2,650 ORFs, was used to build the Venn diagram. Numbers in intersecting regions correspond to the number of orthologs shared by those strains. Numbers in non-intersecting regions correspond to the number of ORFs without an ortholog in any of the other three strains, and in parentheses are the numbers of these that are singletons (that is, without non-orthologous homologs, and therefore unique to the strain). Numbers in black represent total ORFs, and numbers in red represent MTases (i.e., represented in Table 2).

**Fig 2:**
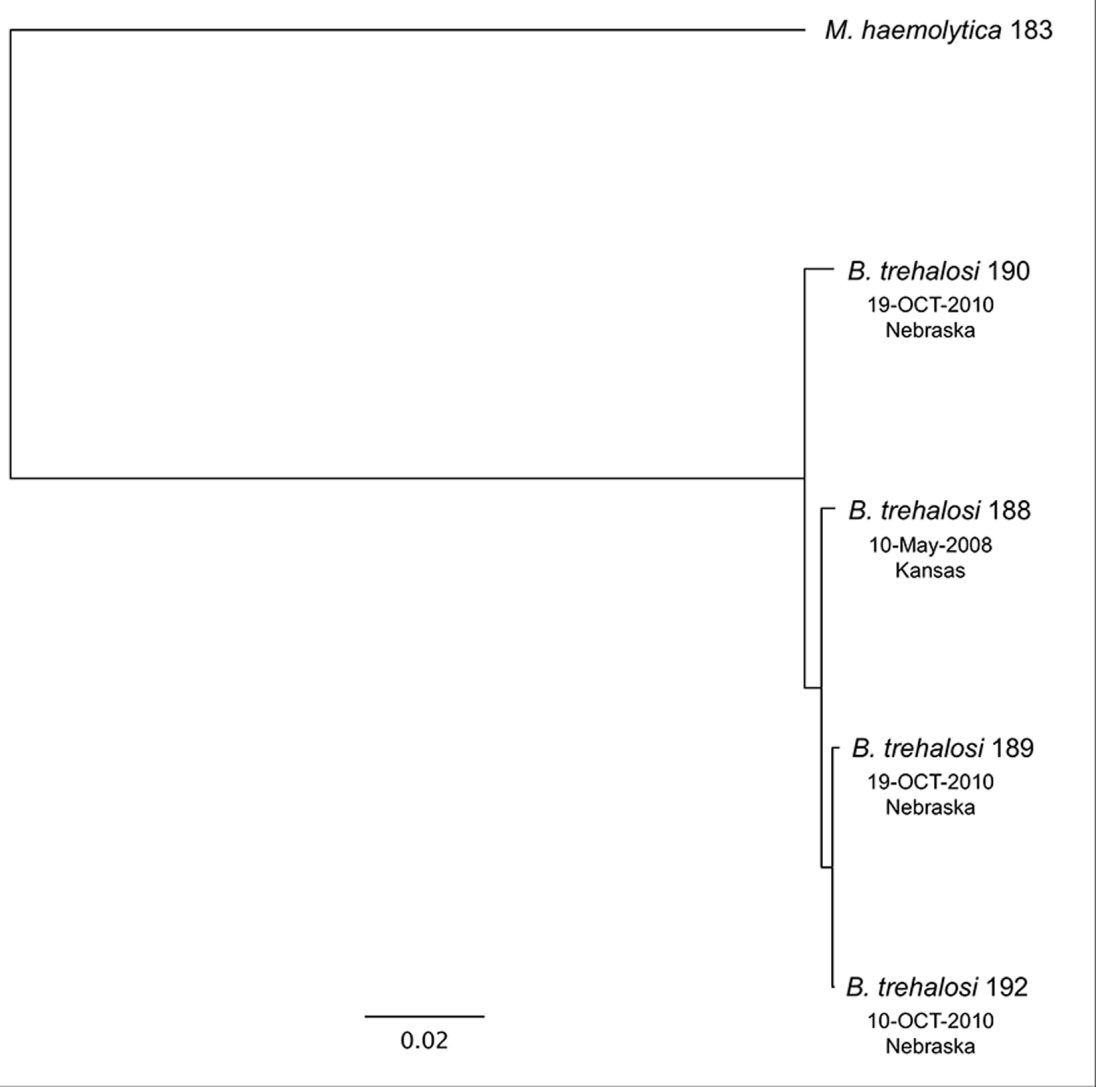
Phylogenetic tree of the four *B*. *trehalosi* isolates sequenced in this work. The tree was built from an amino acid MUSCLE alignment of all the core genes (a total of 1,969,970 amino acid residues per genome). This alignment was used to compute a Kimura distance matrix that was input to PHYLIP [34] to create the Neighbor-Joining tree as described at the EDGAR website. The genome of a related Pasteurellales species, *Mannheimia haemolytica* 183 (CP004752.1), was used as the outgroup to root the tree. The tree topology showed 100% concordance with 500 bootstrapping iterations.

We compared the protein coding sequence (CDS) content of four sequenced strains of *B*. *trehalosi* (Table 1) using the EDGAR platform [33]. A CDS may have an orthologous gene in another strain (defined as the reciprocal best BLASTP hit of its translated sequence to a translated CDS in another strain), may have a non-orthologous homolog (defined as the best BLASTP hit, although non-reciprocal), or may be a singleton (defined as having no BLASTP hit above the default EDGAR threshold, Score Ratio Value 0.3). Results show a core genome of 1,963 CDS (defined as having an ortholog in all four strains), and a pan genome of 2,650 CDS (Fig 1 and S2–S4 Tables). There are 353 “auxiliary” CDS that are shared by at least two, but not all, genomes, while 334 non-core CDS do not have an ortholog (reciprocal best BLASTP hit) in any other strain, of which 208 are singletons. A phylogenetic tree based on sequence alignment of core proteins shows that *B*. *trehalosi* strains 189 and 192 are the most similar, while strain 190 is the most dissimilar (Fig 2), and consistent with this, *B*. *trehalosi* strains 188, 189 and 192 share the greatest number of orthologs of any three strains, at 139 (Fig 1 and S2 Table).

**Table 1:** Genome sequence data for four *B*. *trehalosi* strains.

Isolate	GenBank	Size (bp)	%G+C	Protein-coding genes	R-M systems
USDA-ARS-USMARC 188	CP006954	2,340,975	38.3	2,053	6
USDA-ARS-USMARC 189	CP006955	2,454,127	41.0	2,127	8
USDA-ARS-USMARC 190	CP006956	2,443,169	36.8	2,160	6
USDA-ARS-USMARC 192	CP003745	2,407,846	41.0	2,137	7

For each strain, we examined two sets of CDS using Blast2GO to identify enriched biological functions: the CDS with no orthologs but at least one non-orthologous homolog in another strain, and the singletons. The only sets with statistically significant [false discovery rate (FDR) of < 0.05] enrichment of GO terms were the two sets from *B*. *trehalosi* 190. The 228 non-orthologous CDS in *B*. *trehalosi* 190 are enriched in terms such as DNA integration, DNA recombination, DNA binding, multi-organism process and transposase activity, and depleted in terms such as cytoplasm, single-organism biosynthetic process, alpha-amino acid metabolic process and cellular protein metabolic process. The 179 singletons are depleted in terms such as single-organism cellular process, organic substance biosynthetic process, cytoplasm and cellular biosynthetic process. This is not unexpected given that “non-core” or “accessory” genes are generally thought to be acquired by horizontal transfer, requiring the action of mobility and recombination functions [35, 36].

We also looked for large-scale chromosomal differences using ProgressiveMAUVE and identified several distinguishing features (S1 Fig). These include an insertion sequence (IS)-mediated inversion of 12 kb in strain 190; loss of a large multi-drug resistance gene cluster in strain 188; an apparent 56 kb integrated plasmid in strain 190; and rearrangement of the multi-drug resistance region in strain 190 to a new location adjacent to the integrated plasmid (S1 Fig).

### Methylome Analysis

We also analyzed the kinetic data for these four strains, which were sequenced using the SMRT platform, to determine the patterns of methylation. Five distinct methylated motifs were detected in these strains (Table 3), which showed varying degrees of conservation ranging from those present in all strains to those present in only a single strain. For all motifs, the methylation appeared evenly distributed across the genomes (examples shown in S2 Fig), and the minority of unmethylated sites did not show significant positional bias (examples shown in S3 Fig). The Dam methylation pattern G^m6^ATC was present in all four strains, which is as expected since the *dam* gene was acquired prior to the formation of the *Pasteurellaceae*, the order to which *B*. *trehalosi* belongs [37]. All four *B*. *trehalosi* genomes also encode other proteins functionally associated with Dam such as SeqA, MutH, HN-S, StpA, PriB, and MukFEB [37].

**Fig 3:**
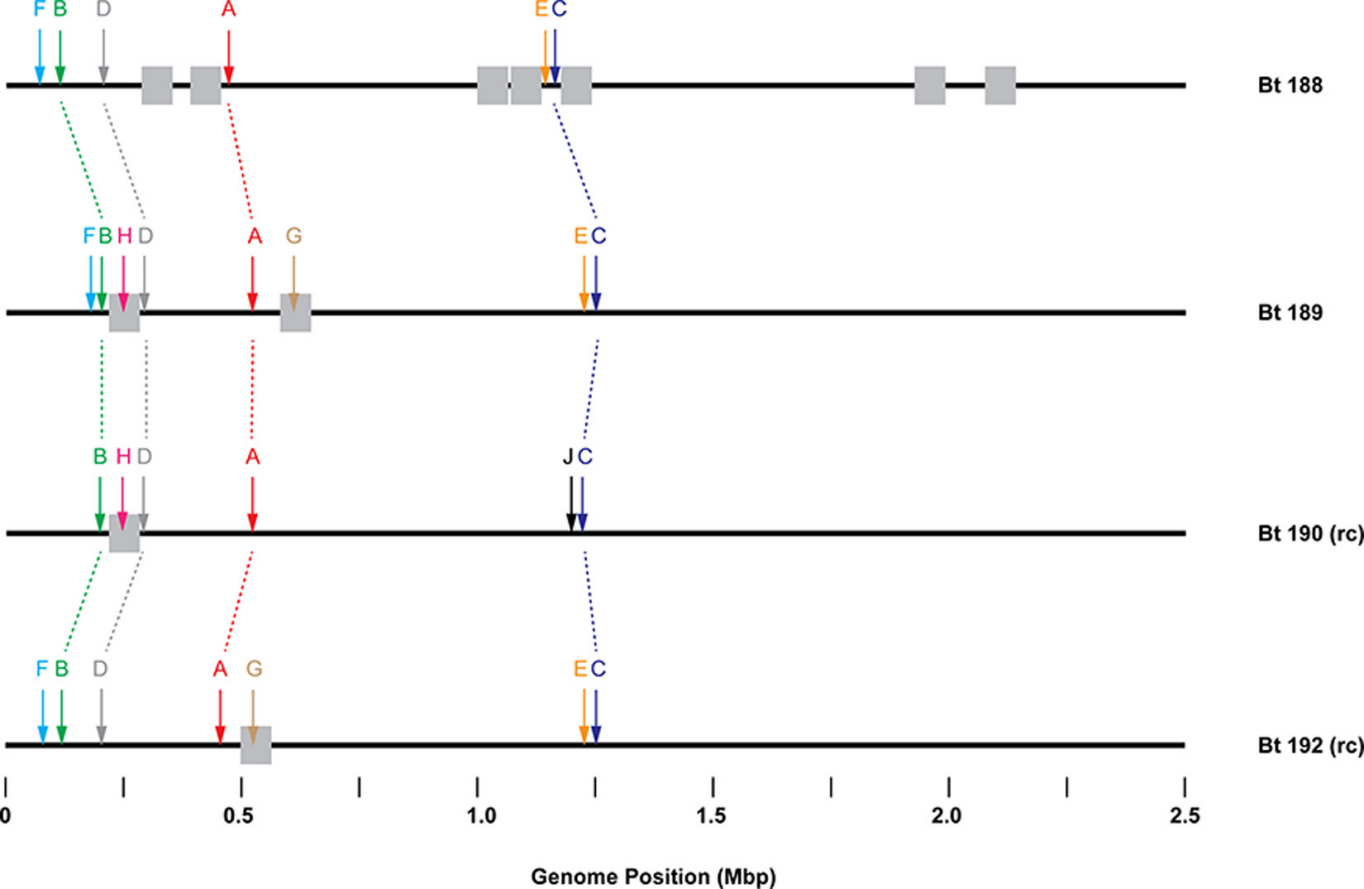
Schematic representation of the four *B*. *trehalosi* genomes, showing approximate locations of prophages and MTase genes. Gray boxes (not to scale) mark the locations of prophage regions, as annotated by PHAST. Colored arrows mark the locations of MTase genes, with arrows of the same color marking orthologous genes. Genes are labeled with the letter groups defined in Table 2. Those genes shared by all four genomes are connected by dotted lines. Strains 190 and 192 are shown in reverse-complement orientation relative to the NCBI sequences, so that all four are in the same relative orientation.

DNA MTase and other RM-associated genes were identified in each genome using SEQWARE [24], and the MTases were grouped into sets of putative orthologs, each assigned a letter designation, based on sequence similarity and gene neighborhood criteria (Table 2). Protein sequences of orthologous genes were typically 100% identical to one another, although there were minor differences in some cases. The approximate locations of these genes are shown in Fig 3, and we found that two MTase groups (G and H) are located within putative prophage regions, as identified by PHAST [38]. Except for groups A (*dam*) and G (prophage-encoded), all of the MTases are clustered in two genomic “hot spots,” one between 0–0.25 Mb and the other around 1.25 Mb (Fig 3). These hot spots are not located in the regions of genomic rearrangement discussed above (S1 Fig and Fig 3).

**Table 2:** MTase genes in four *B*. *trehalosi* strains.^a^

Group^a^	Type	Organization^b^	Site	188^c^	189^c^	190^c^	192^c^
A	Orphan	M	G^m6^ATC	**M.Btr188I**	**M.Btr189II**	**M.Btr190IV**	**M.Btr192I**
B	I	M-x-S-x-R	^m6^ACGN_6_CGT	**M.Btr188II**^**d**^	**M.Btr189I**		**M.Btr192III**^**d**^
B			G^m6^AGN_6_GTC			**M.Btr190I**^**d**^	
C	III	M-R	AC^m6^ATC	10530	11930		**M.Btr192II**^**d**^
C			RGTA^m6^AT			M.Btr190II^d^	
D	II	M^e^	no activity	1790–1760	2480–2420	20590–20600^d^	20770–20830
E	I	M-S-x-R	AC^m6^AN_6_TTTA	**M.Btr188III**^**d**^	11560	–	11890
F	II	M-R	C^m5^CWGG	820	850	–	M.Btr192V^d^
G	Orphan	M	^m6^A	–	5700	–	M.Btr192IV^d^
H	Orphan	M	G^m6^ATC^f^	–	2030	M.Btr190III^d^	–
J	IIG	MR-S	no activity	–	–	12260^d^	–

^a^ Orthologous groups are assigned letters for comparison with Table 3.

^b^ M = MTase, R = REase, S = specificity protein, and x = any other gene; genes are separated by hyphens.

^c^ If the gene has not been assigned an activity by REBASE, only the locus tag number is given, with “M.Btr…P” removed for clarity; absence of an ortholog is denoted by a hyphen. Those MTases that are active in the native hosts and responsible for one of the observed activities in Table 3 are shown in boldface.

^d^ Activity tested by cloning.

^e^ Gene broken; frameshifted or multiple subunits required.

^f^ Significant off-target activity was also observed in the clone.

**Table 3:** Methylated motifs in four *B*. *trehalosi* strains.^a^

Site	188	189	190	192	MTase Group^b^
G^m6^ATC	95	98	95	91	A
^m6^ACGN_6_CGT	86	97	^–^	95	B
G^m6^AGN_6_GTC	–	–	93	–	B
AC^m6^ATC	–	–	–	94	C
AC^m6^AN_6_TTTA	88	–	–	–	E

^a^ Output from the PacBio “RS_Modification_and_Motif_Analysis.1” program. Numbers indicate the percentage of all sites determined by the program to be so modified. Instances where this motif was not detected are indicated by hyphens.

^b^ This refers to the group of orthologous MTase genes, as defined in Table 2, that is responsible for each observed methylated motif.

General predictions of which motifs were associated with which MTases could be made for some genes based on evidence such as the type of R-M system, similarity to previously characterized MTases, and patterns of presence and absence of genes and motifs across strains. However, precise matching of genes and activities was determined by cloning MTase genes in isolation to separate the activities. At least one representative from most sets of orthologs (i.e., one member of each row in Table 2) was cloned and overexpressed in a methylation-deficient *E*. *coli* strain, and the genomic DNA of the clone was sequenced using the SMRT platform to identify the methylation activity. Using this process, the genes responsible for all five methylated motifs identified in the four native *B*. *trehalosi* genomes were determined. In addition, four MTases (M.Btr190II, M.Btr190III, M.Btr192IV, and M.Btr192V) demonstrated activity when cloned that was not observed in the native context, suggesting their expression is silenced in *B*. *trehalosi* under the growth conditions we used.

### MTases Shared Between All Four *B*. *trehalosi* Strains

#### M.Btr188I (Group A)

The product of this gene is 60% identical to M.EcoKDam. Furthermore, it is an orphan (i.e., lacking a cognate REase), shares some genomic context with *E*. *coli dam* (downstream of 3-dehydroquinate synthase and shikimic acid kinase genes), and has an ortholog in all four strains we examined, suggesting it is the *dam* ortholog. An example of this group was not cloned, but based on homology and synteny we presume that M.Btr188I and other members of group A are functionally equivalent to *E*. *coli dam* and responsible for the G^m6^ATC modification observed in all four strains of *B*. *trehalosi*. A second MTase capable of methylating G^m6^ATC is present in strains 189 and 190 [see the entry for M.Btr190III (group H) below]. However, in both strains 189 and 190, that MTase is encoded on a prophage (Fig 3), where DNA MTases are in some cases rendered inactive [39]. This second G^m6^ATC MTase exhibited significant off-target activity when cloned, which was not observed in the *B*. *trehalosi* 189 and 190 genomic DNA, further suggesting its inactivity in the genome.

#### Btr188II and Btr190I (Group B)

All four *B*. *trehalosi* strains contain a Type I R-M system with the gene order M-S-R, where there are one or two intervening ORFs between M and S, and between S and R. The systems in strains 188, 189, and 192, including the intervening ORFs, are essentially identical, and all three of these strains exhibit methylation at the palindromic site ^m6^ACGN_6_CGT. We cloned the MTase and specificity-governing subunit from two of these three, *btr188IIMS* and *btr192IIIMS*, and found that they both indeed methylated this sequence.

**Fig 4:**
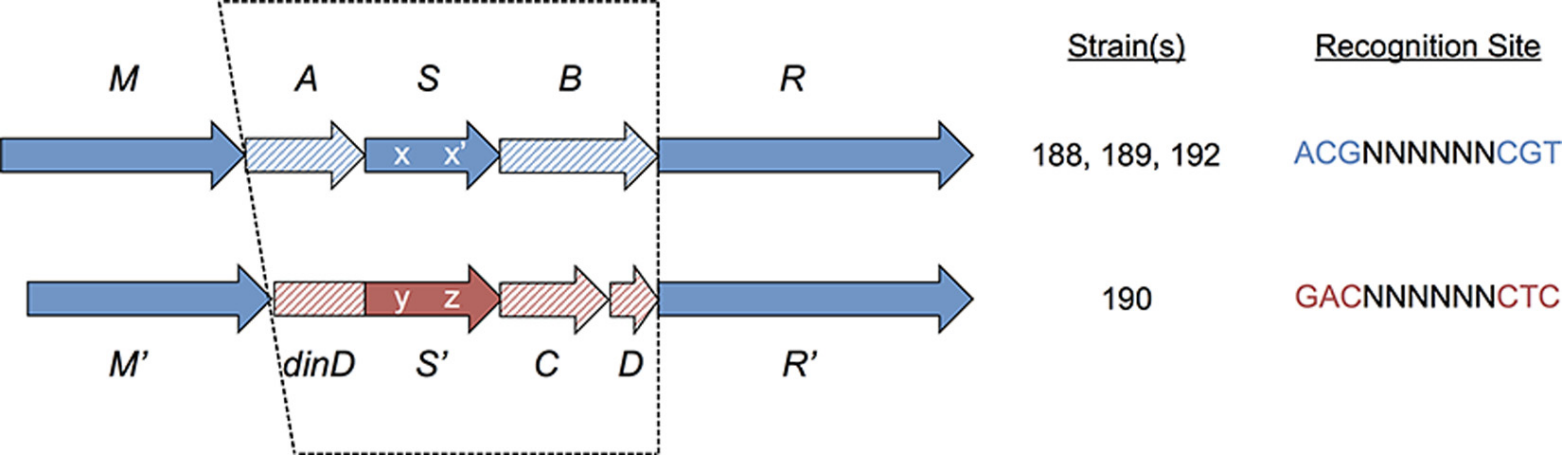
Gene comparison of *btr188II* R-M system orthologs. Top row, gene order of orthologous systems from *B*. *trehalosi* strains 188, 189 and 192. Bottom row, gene order of orthologous but functionally distinct system from strain 190, *btr190I*. R-M system genes are shown in solid color, and apparent non-R-M related genes are cross-hatched. Genes from the *btr190I* system similar to those in the other three strains are shown in blue, and genes unrelated to those in the other three strains are shown in red; the region of apparent replacement is shown by the dotted lines. Note *btr190IS* is fused to the upstream gene, a homolog of *dinD*. The TRDs of the two *S* genes are indicated by lower-case letters, showing that the *S* gene in the top line comprises two imperfect copies of the same TRD, resulting in a palindromic recognition site.

The Btr190I system occurs at the same locus as the other three and its R and M genes are highly similar to the other group B members. However, its S gene and the intervening ORFs that flank it are not homologous, suggesting a gene conversion event that involved homologous recombination at or outside the R and M genes (Fig 4). Furthermore, the S gene is fused at its 5’ end to a copy of the DNA damage-inducible gene *dinD*, a feature not found in the other group B members. We cloned *btr190IMS* and found that it methylated the site G^m6^AGN_6_GTC, which was observed only in strain 190 (Tables 2 and 3) and is consistent with the novel S subunit sequence. Interestingly, the fusion of *hsdS* to *dinD* does not seem to have prevented its activity as a specificity protein for this system.

#### M.Btr192II (Group C)

This MTase is part of a Type III R-M system present in all four strains. The MTase gene, which in Type III systems contains the specificity determinant, is 100% identical in strains 188, 189 and 192, while that in strain 190 has undergone an apparent gene conversion event that has replaced the central TRD region with a non-homologous sequence. (The TRD region in strain 190 bears closest similarity to that of M.Sdy378ORF287P, of unknown specificity.) Only one methylated motif indicative of a Type III system was observed in the *B*. *trehalosi* strains, namely AC^m6^ATC, which was observed only in strain 192. We cloned the *btr192IIM* and *R* genes together and found they methylated this sequence, but interestingly, we observed no activity when *btr192IIM* was cloned in isolation.

We also cloned the homologous system from strain 190, *btr190IIM* and *R* together, and found that it modified the sequence RGTA^m6^AT, a different sequence from Btr192II, consistent with the replacement of the TRD. This site is not modified in strain 190 or any of the other three, suggesting that the Btr190II R-M system is likewise silenced in its native host under the conditions we observed. We did not attempt to clone *btr190IIM* in isolation.

The accompanying REase gene appears to be intact and nearly identical in all four strains, so the requirement for the REase probably does not account for the silencing of this system in strains 188, 189, and 190. There is an SSR that lies upstream of the gene, but its relevance to silencing is unclear (see below).

#### Btr188ORF1790 and Btr188ORF1760 (Group D)

These genes lie near each other in all four genomes, and may represent pieces of the same ancient Type IIG R-M system, bearing greatest similarity to that of DrdVI (29). The incarnations in strains 188, 189 and 192 are nearly identical to one another, while that in strain 190 has diverged considerably. Btr188ORF1790, which is either frameshifted or present as multiple ORFs, bears similarity to both M1.DrdVI at its N-terminus and M2.DrdVI at its C-terminus. Btr188ORF1760, which contains a GIY-YIG endonuclease domain, resembles DrdVI (a fused translocase and REase), and is present in its full length only in strain 190. In the other three strains, the gene is truncated by a frameshift and represents only an N-terminal fragment. Several small ORFs now intervene between Btr188ORF1790 and Btr188ORF1760 in all four *B*. *trehalosi* strains, and may represent additional fragments of the original gene. Due to its fragmented nature, and because it is not active in any of the four strains examined in this work, this system was not analyzed further.

### MTases Shared by Three of Four *B*. *trehalosi* Strains

Two sets of MTase genes are encoded by three of the four strains under study. In all cases, the strain from which it is missing is 190.

#### M.Btr188III (Group E)

This MTase is part of a Type I R-M system found in *B*. *trehalosi* strains 188, 189, and 192 but only active in strain 188. At the 5’ ends of these MTases is a five base SSR (ACAGC) that exhibits copy number variation between strains (see below). We cloned *btr188IIIMS* and found it methylated the sequence AC^m6^AN_6_TTTA. Although an ortholog of this gene is found in three strains, the sequence AC^m6^AN_6_TTTA is methylated only in strain 188, likely as a result of changes in the SSR copy number. Consistent with this, only in *btr188IIIM*, which contains 27 repeats, is the gene in-frame with the ATG start codon.

Strain 190 also contains an R-M system (group J; see below) at the same locus as group E (Fig 3). However, it is a Type IIG system and bears no similarity to group E. There are no obvious repeat sequences surrounding this locus, so the change may have been driven by homologous recombination at or beyond the flanking genes, which encode an aspartate––tRNA ligase and a *glmU* homolog.

#### M.Btr192V (Group F)

This enzyme is encoded by an m^5^C DNA MTase gene that appears to be part of a Type II R-M system found in strains 188, 189, and 192. It is the only identifiable m^5^C MTase gene in the *B*. *trehalosi* strains analyzed here. We cloned the homolog from strain 192, and TET treatment of the clone gDNA amplified its kinetic signal enough to deduce a recognition sequence using SMRT sequencing at 118x coverage. Under various modification QV thresholds between 30 and 100, the most likely recognition sequence appeared to be C^m5^CGGG as determined by the RS_Modification_and_Motif_Analysis software (PacBio), with modification primarily or exclusively on one strand. As expected, this modification was not detected by SMRT sequencing of the untreated *B*. *trehalosi* 192 gDNA.

To further elucidate the recognition site, we challenged both the clone gDNA and the native *B*. *trehalosi* 192 gDNA with several restriction enzymes: HpaII (CCGG), NciI (CCSGG), PspGI (CCWGG), and StyD4I (CCNGG). The clone exhibited virtually complete protection from PspGI cleavage, but not the others, while *B*. *trehalosi* 192 gDNA exhibited weak protection from PspGI and StyD4I cleavage (data not shown). PspGI is blocked by full m^5^C methylation, but not hemi-methylation, at either C position [40], indicating the correct recognition sequence for M.Btr192V is C^m5^CWGG. The fact that the gDNA of the native host *B*. *trehalosi* 192 was only partially protected indicates that the MTase may be poorly expressed under native conditions.

The group F genes are flanked by short direct repeats with the sequence TCTTTATA. Recombination between these sites may have caused the loss of this R-M system in strain 190.

### MTases Shared by Two of Four *B*. *trehalosi* Strains

Two sets of MTases are encoded by only two of the four strains under study, both of which appear to be orphans. Both are encoded on apparent prophages as determined by PHAST analysis (Fig 3), consistent with the sporadic distribution among the strains.

#### M.Btr192IV (Group G)

The orphan MTase M.Btr192IV is found only in *B*. *trehalosi* strains 192 and 189, and was not obviously responsible for any of the observed methylated patterns in the *B*. *trehalosi* genomes. It is highly similar to the promiscuous m^6^A MTase M.HindVII (also called *hia5*) from *Haemophilus influenzae* [39], which modifies B^m6^A sequences, is found on a prophage and we expected it to have similar properties. We cloned *btr192IVM* and found it was indeed a promiscuous m^6^A MTase modifying essentially all adenine residues (^m6^A).

#### M.Btr190III (Group H)

The gene encoding this MTase, present only in strains 189 and 190, is related to the G^m6^ATC-modifying M.EcoT1Dam (35% amino acid identity). We cloned the gene encoding M.Btr190III and found it indeed methylated the sequence G^m6^ATC, but it also exhibited significant off-target activity, modifying significant fractions of many related sequences as well. Any methylation of G^m6^ATC by this protein in the native host would be masked by the action of the group A (*dam*) ortholog, which we know to be active in strains 188 and 192 (Table 3). In strains 189 and 190, where both groups A and H are present, we cannot determine how much of the observed G^m6^ATC methylation is due to each gene. However, because we see no modification of these off-target sequences in the native *B*. *trehalosi* genomes, we suspect that the predominant activity is due to the *dam* ortholog and not to the prophage-encoded group H.

#### MTase Exclusive to One *B*. *trehalosi* Strain (Group J)

Btr190ORF12260P is a two-gene R-M system that is found only in strain 190, and it consists of a Type IIG fused R-M gene and a companion S gene, similar to the BcgI R-M system. We cloned the two genes together but did not observe any activity in the clone, consistent with the fact that it does not seem to be responsible for any of the observed activities on the original genomic DNA. The reason for its inactivity is not immediately clear, since both genes appear to be full-length with no frameshifts. As described above, it occurs at the same locus as group E in the other three strains, but is not homologous to them.

### Simple Sequence Repeats (SSR) Affecting MTase Genes

In several other systems MTase genes have been found to vary in their expression as a result of SSRs present at their transcription/translation start sites. Of the eight sets of non-fragmented DNA MTase genes in these *B*. *trehalosi* strains (Table 2), three have apparent SSRs close to the CDS start codon.

In group G, both M.Btr192IV and M.Btr189ORF5700P are located on prophages, and both have 12 copies of CAAG within their coding sequences starting 63 bp downstream of the initiator codon. Neither of these genes is expressed in its native host, so the significance of the SSR is unclear.

Group C members, present in all four strains, are Type III MTases. The MTase is inactive in *B*. *trehalosi* strains 188, 189, and 190 but active in strain 192. In strains 190 and 192 there are 15 copies of the pentanucleotide CGCAA, which form an SSR the 3’ end of which lies 43 bp upstream of the initiator ATG of the MTase gene. This same 43 bp spacing is preserved in strains 188 and 189 (allowing for one imperfect copy at the end), but the SSR length itself varies, with 17 copies of the repeat in strain 188 and 14 copies in 189. Given that the length of the SSR is different between the “active” strain 192 and the “inactive” strains 188 and 189, and the fact that there are no other differences within either the MTase ORF or 1 kb upstream of it, it might be suggested that the SSR is playing a role in silencing. However, the SSR length is identical between the “active” strain 192 and the “inactive” strain 190. Strain 190 has additional single-base changes upstream of the MTase ORF, so its silencing may be due to a different mechanism. This phenomenon warrants further study.

In group E, M.Btr188III has 27 copies of the pentanucleotide ACAGC within its coding region resulting in an in frame version of this repeat. However, in strains 189 and 192 there are only 17 copies of the repeat putting it out of frame. The translated N-terminal protein sequence of these genes is MPN(STAQH)_*n*_, where *n* is dependent on the repeat copy number.

## Discussion

Within a bacterial species, strains can undergo rapid changes in genetic content and phenotype due to horizontal gene transfer, recombination, and epigenetic mechanisms [5, 41, 42]. Such changes, the scale of which far outstrips that of base-by-base mutation, enable bacteria to quickly respond to environmental changes and to exploit new ecological niches [43–45]. With regard to bacteria such as *B*. *trehalosi*, which dwell within metazoan hosts, these changes can involve alternation between commensal and pathogenic states. Our comparative study of four strains of this organism has uncovered several mechanisms of rapid change, which we hope will ultimately shed light on the etiology of ruminant respiratory disease. Further study will necessarily include association of strain genotypes with virulence and pathology and thus uncover connections between genotype, epigenetic patterns, and fitness for populating the ruminant lung.

In the case of the *B*. *trehalosi* strains we have examined, epigenetic patterns and genotypes are directly linked through MTase genes. We have observed changes in MTase activity that appear to have arisen through several mechanisms, which differ in reversibility and persistence: (1) non-reversible gain or loss of MTases through bacteriophage integration and recombination; (2) reversible activation or inactivation of MTase expression through SSM at SSRs; (3) non-reversible alteration of activity by recombination at TRD regions within R-M systems; and (4) silencing of MTases through unknown mechanisms, which may or may not be reversible. The results of these changes, in aggregate, are the alteration of methylomes, which we were able to examine using SMRT sequencing (Table 3).

We observed several cases where MTase genes were present but not active. The reasons for this inactivity are in all cases unknown, but it is possible that the *in vitro* conditions used for growing the strains did not replicate the environment(s) necessary for expression. However, in the case of group E described above, our failure to detect activity in two of the three strains where the gene is present is parsimoniously explained through the effect of SSM on MTase activity. Within the CDS N-terminal region of the only active homolog, M.Btr188III, there are 27 copies of the tandem repeat ACAGC. However, *B*. *trehalosi* 189 and 192 have 17 copies of this repeat, resulting in a frameshifted and a shorter open reading frame that apparently does not code for an active MTase. A similar mechanism may be at work in groups C and G as well, although the association with SSR copy number and activity is less obvious.

Methylation in bacteria has been traditionally viewed as a mechanism to protect the host from the action of REases, and thus the methylome has been viewed as simply a byproduct of MTases performing this protective function. However, it is becoming increasingly apparent that specific methylation marks can affect bacterial gene expression, and therefore can affect cellular processes not obviously connected to restriction-modification. Well known examples include the orphan MTases Dam in Gamma-Proteobacteria [46] and CcrM in Alpha-Proteobacteria [47], and more recent work has shown that MTases within R-M systems can also have consequential transcriptional effects [43, 44, 48]. Therefore, changes in the activity of any given MTase may, through resulting changes in the methylome, have unknown, pleiotropic effects. It is in such effects that the links to pathogenicity may lie, and a more detailed study of gene expression in one or more of the strains described here, under varying growth conditions, is likely to be rewarding. Understanding the nuances of epigenetic variation within the methylome, and the effect of such variation on the transcriptome, could ultimately be important in developing strategies to mitigate ruminant respiratory disease associated with infection by *B*. *trehalosi* and possibly other Pasteurellaceae.

## Supporting Information

S1 Fig ProgressiveMAUVE alignment of the four *B*. *trehalosi* chromosomes sequenced in this work. The two general “hot spots” where MTases are located are indicated at the top. Lower case letters indicate the following regions of interest: (a) IS-mediated inversion of a 12 kb region in strain 190; (b) Short region containing a MarR family transcriptional regulator and tetracycline efflux gene, partially duplicated in region d, and largely missing from strains 190 and 188; (c) Multi-drug resistance region, missing from strain 188; (d) Short region containing a beta-lactamase gene and a partial duplication of region b, missing from strain 188 and 190 with the exception of the beta-lactamase gene in strain 190; (e) Possible integrated plasmid, 56 kb, including such functions as ParB and TraG, and present only in strain 190. Note that region c and the beta-lactamase gene from region d are found at a different location in strain 190 than in strains 189 and 192.
(PDF)

S2 Fig Circos plots showing the distribution of methylated sites for the three motifs in strain 192. The outermost track carries the base modification signal with height proportional to the interpulse distance ratio for each modified base on the positive strand. The next track within marks the positions of the motif on the positive strand. This scheme is reversed for motifs on the negative strand, with the inner most track showing the methylation signal. The plots were generated using BaseModFunctions.v2.1.R [49] and Circos v.0.69–3 [50].
(PDF)

S3 Fig Distribution of unmethylated sites for each methylated motif in strain 188. Unmethylated sites were identified using the getUnmodifiedMotifKin function from BaseModFunctions.v2.1.R [49], and the plots were generated using the same software based on a 6 kb window.
(PDF)

S1 Table Sequences of primers used in this work. (DOCX)

S2 Table ORFs in at least three of the four *B*. *trehalosi* strains. (DOCX)

S3 Table ORFs in at most two of the four *B*. *trehalosi* strains. (DOCX)

S4 Table List of orthologs shared between strains. (PDF)
