# Supplementary figures and images for: Comparative Methylome Analysis of the Occasional Ruminant Respiratory Pathogen Bibersteinia trehalosi
Source: PLoS One. 2016 Aug 24;11(8):e0161499. doi: 10.1371/journal.pone.0161499 (PMC4996451; doi:10.1371/journal.pone.0161499)

S1 Fig

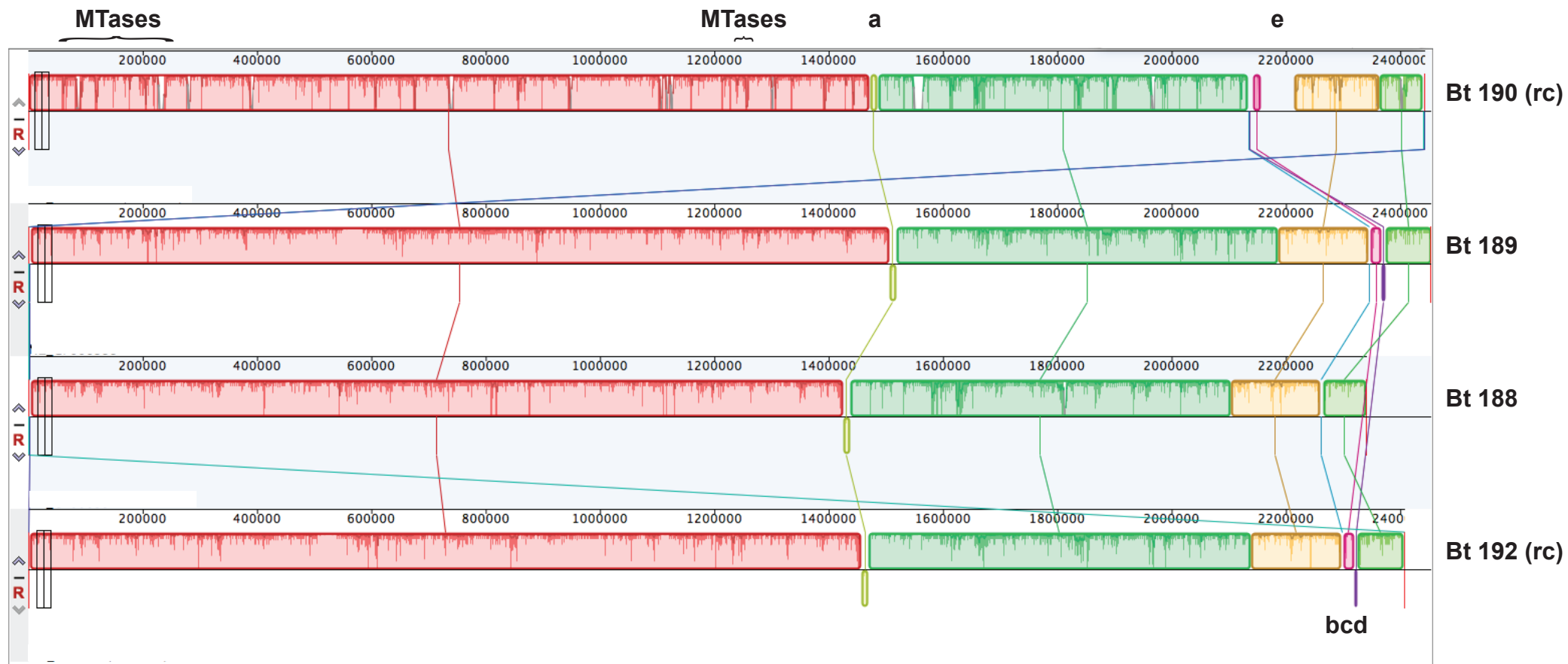

Supplement: S1 Fig — The two general “hot spots” where MTases are located are indicated at the top. Lower case letters indicate the following regions of interest: (a) IS-mediated inversion of a 12 kb region in strain 190; (b) Short region containing a MarR family transcriptional regulator and tetracycline efflux gene, partially duplicated in region d, and largely missing from strains 190 and 188; (c) Multi-drug resistance region, missing from strain 188; (d) Short region containing a beta-lactamase gene and a partial duplication of region b, missing from strain 188 and 190 with the exception of the beta-lactamase gene in strain 190; (e) Possible integrated plasmid, 56 kb, including such functions as ParB and TraG, and present only in strain 190. Note that region c and the beta-lactamase gene from region d are found at a different location in strain 190 than in strains 189 and 192. (PDF) [file pone.0161499.s001.pdf]

*B. trehalosi* 192

G<sup>m6</sup>ATC

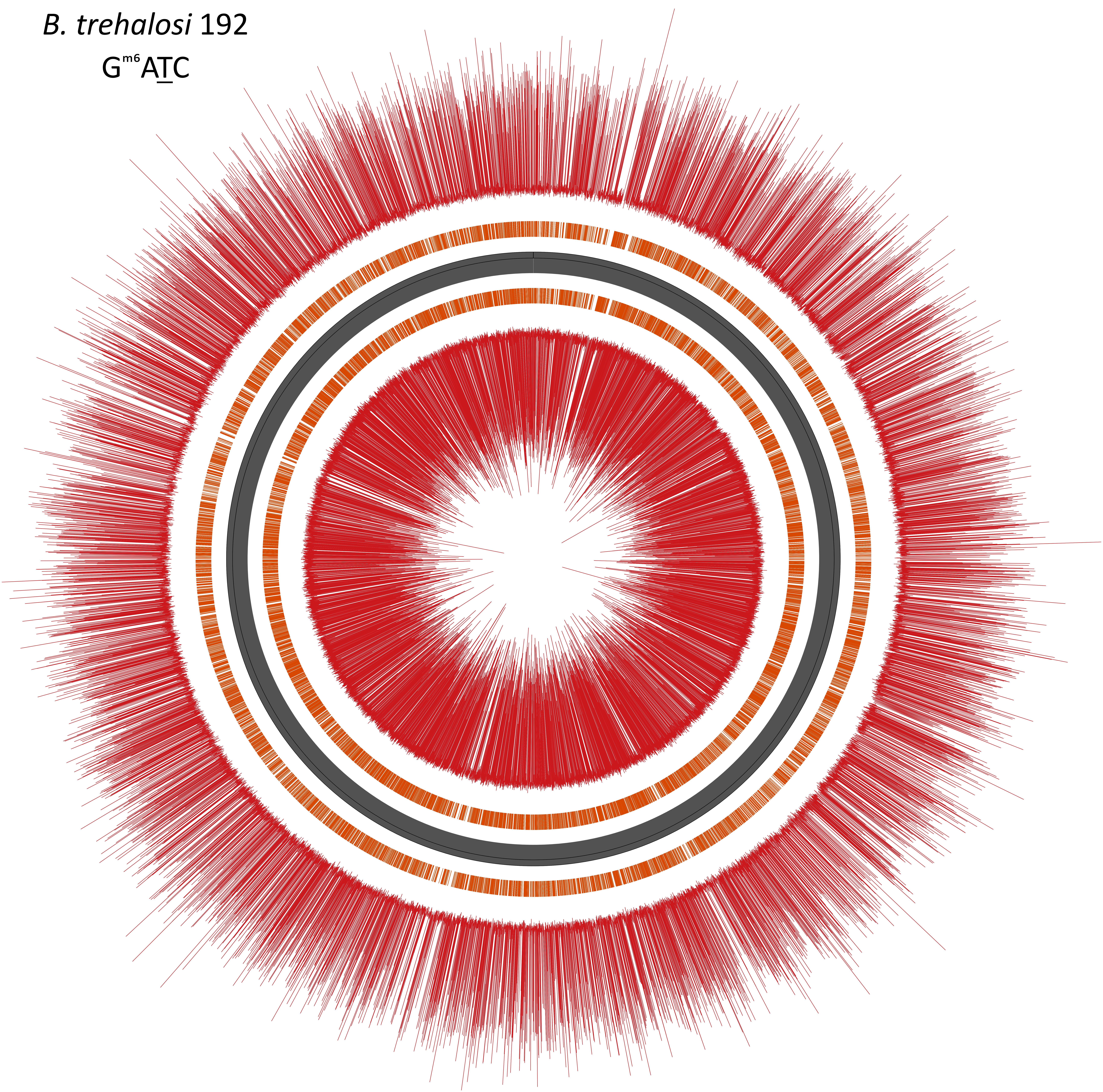

*B. trehalosi* 192

AC<sup>m6</sup>ATC

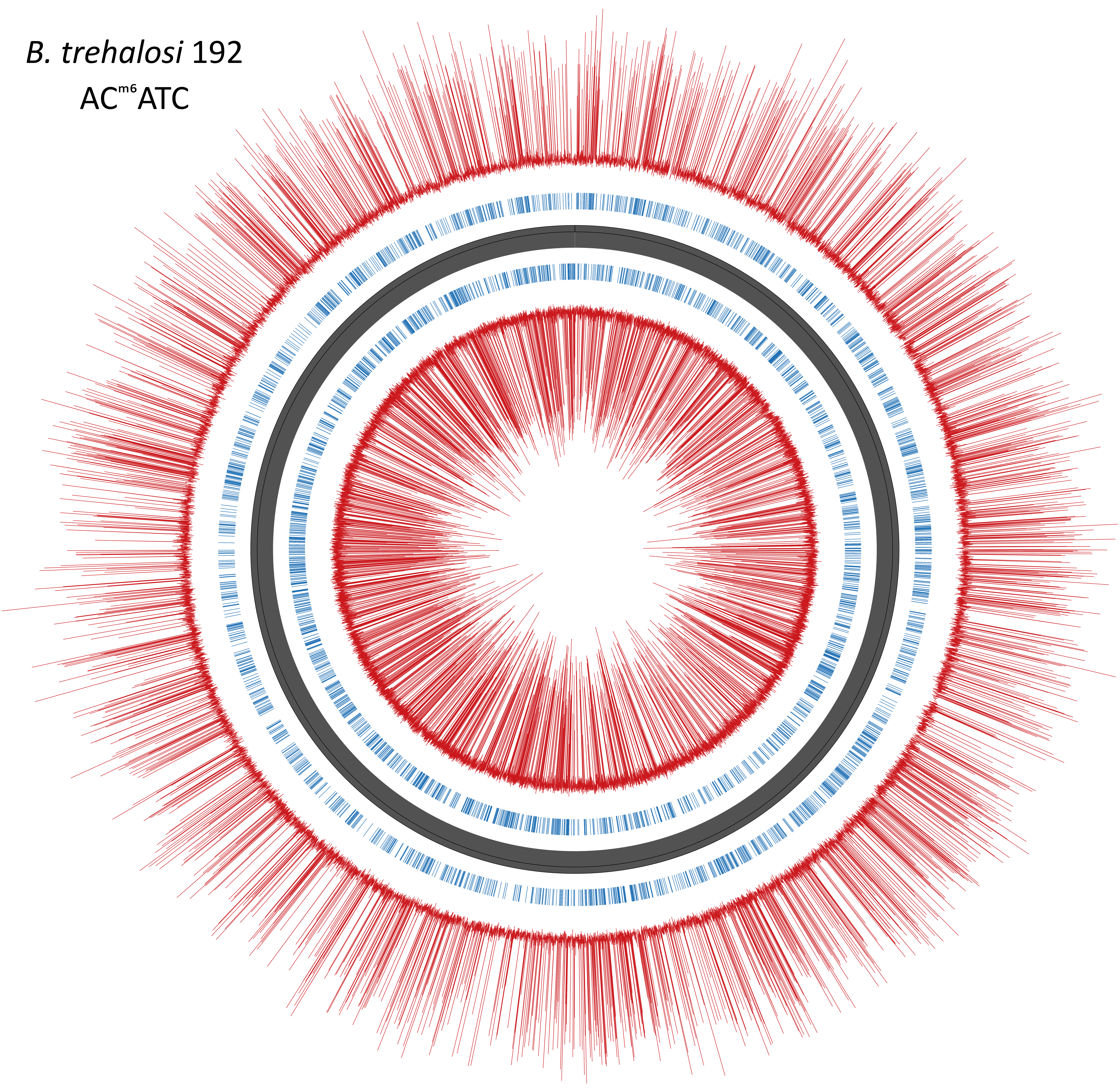

*B. trehalosi* 192

<sup>m6</sup>ACGN CGT

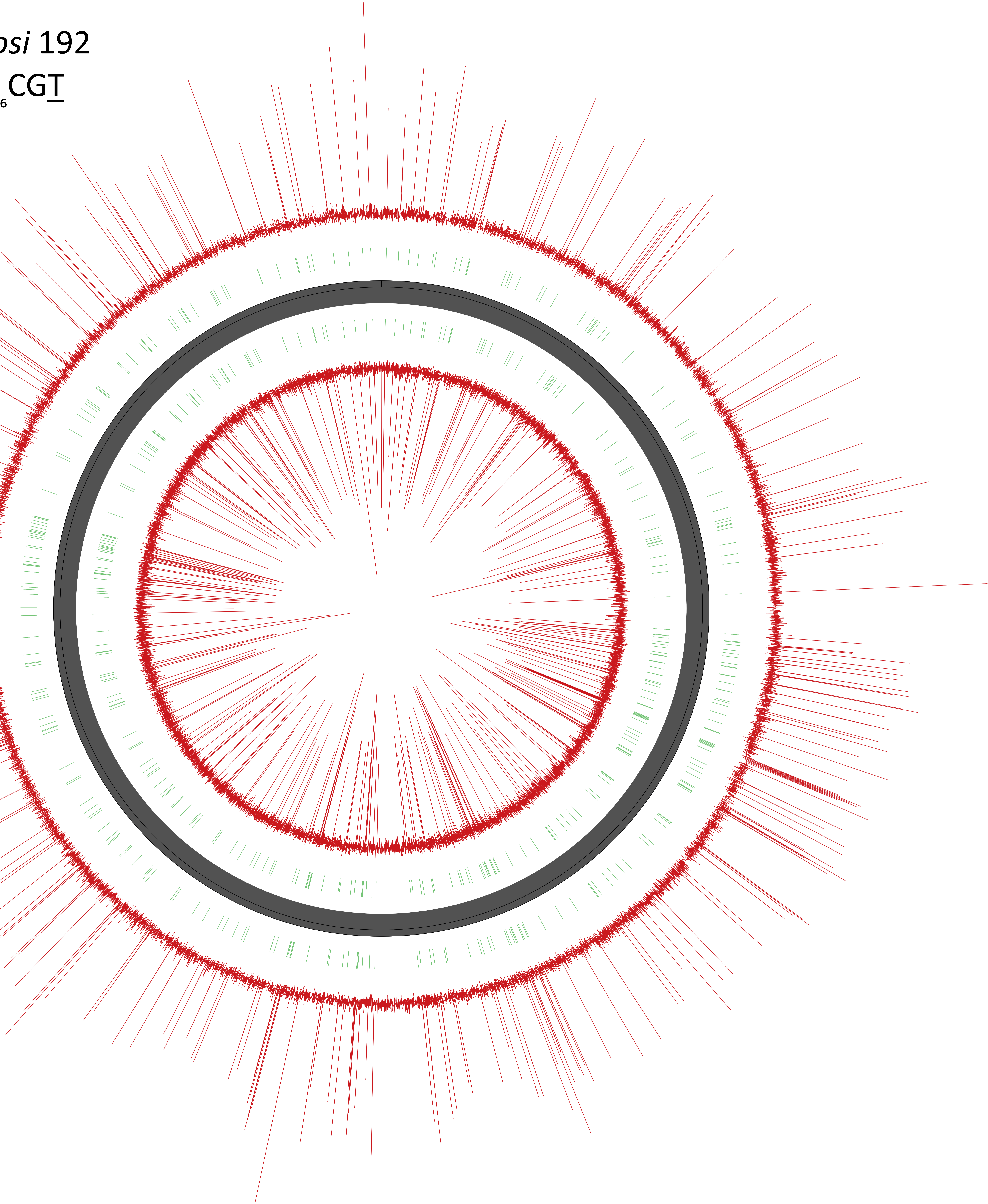

Supplement: S2 Fig — The outermost track carries the base modification signal with height proportional to the interpulse distance ratio for each modified base on the positive strand. The next track within marks the positions of the motif on the positive strand. This scheme is reversed for motifs on the negative strand, with the inner most track showing the methylation signal. The plots were generated using BaseModFunctions.v2.1.R [49] and Circos v.0.69–3 [50]. (PDF) [file pone.0161499.s002.pdf]

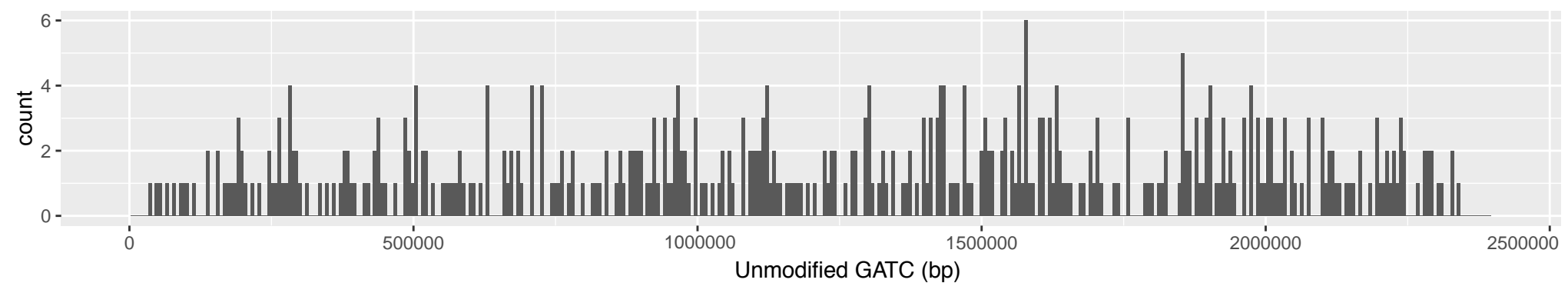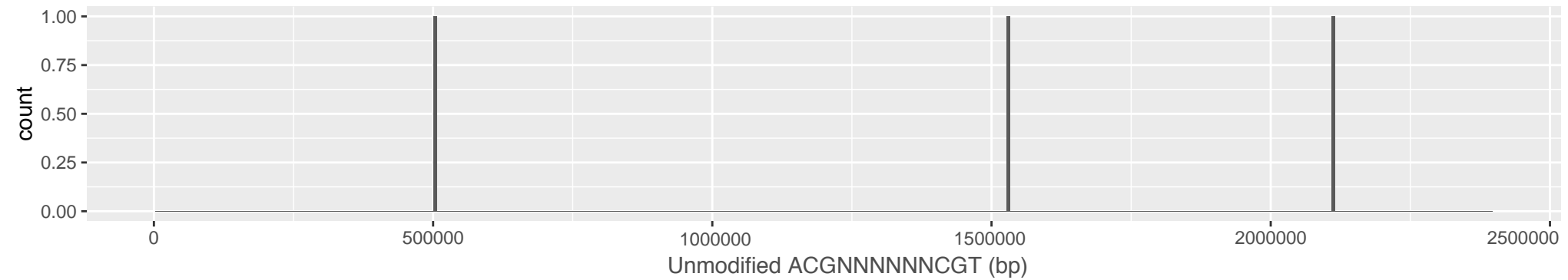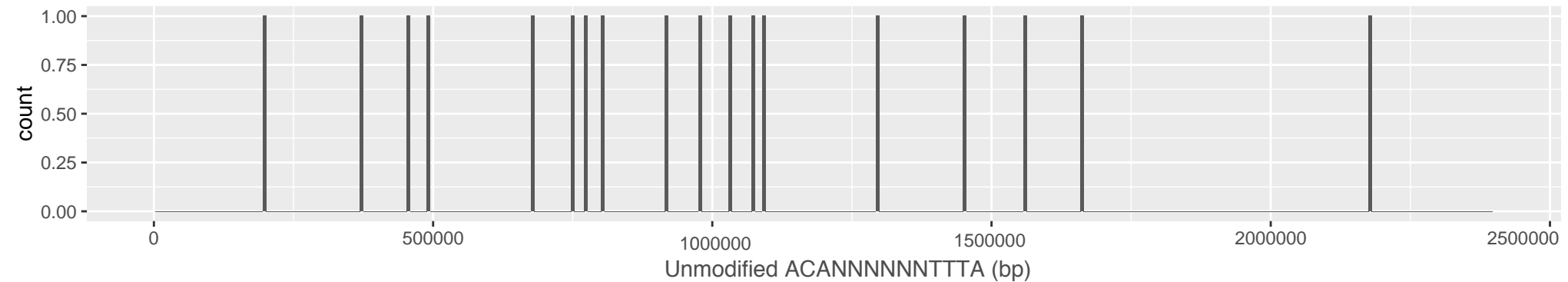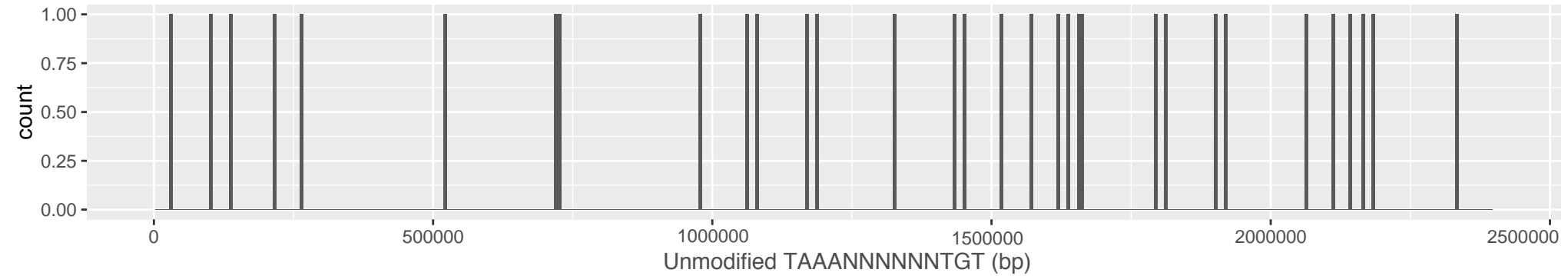

Supplement: S3 Fig — Unmethylated sites were identified using the getUnmodifiedMotifKin function from BaseModFunctions.v2.1.R [49], and the plots were generated using the same software based on a 6 kb window. (PDF) [file pone.0161499.s003.pdf]
