# Supplementary material for: Comparative Methylome Analysis of the Occasional Ruminant Respiratory Pathogen Bibersteinia trehalosi
Source: PLoS One. 2016 Aug 24;11(8):e0161499. doi: 10.1371/journal.pone.0161499 (PMC4996451; doi:10.1371/journal.pone.0161499)
Supplement: S1 Table — (DOCX) [file pone.0161499.s004.docx]

**Supplementary Table S1.** Sequences of primers used in this work.

| **Description** | **Sequence** |
| --- | --- |
| M.Btr188II forward | AAACTGCAGTTAAGGTTAATCATATGAACCACTCAGCCCACAACCGCCTTGTTTCG |
| M.Btr188II reverse | AAAGGATCCACGGTTTCCCGTAAATTAGGGTGTTTAACCTTGCTTAGCAAGGGGATAATC |
| M.Btr190I forward | AAACTGCAGTTAAGGTTAATCATATGAACCACTCAGCCCACAACCGCCTTGTTTCG |
| M.Btr190I reverse | AAAGGATCCGAGGCCAATGTCTTAATTAACTCCAATATTGTTTTCTAAATTACTTACACG |
| M.Btr192III forward | AAACTGCAGTTAAGGTTAATCATATGAACCACTCAGCCCACAACCGCCTTGTTTCG |
| M.Btr192III reverse | AAAGGATCCACGCAGGTTCGTTTAACCTTGCTTAGCAAGGGGATAATCTTGCTTAATCC |
| Btr190II forward | GCAGGTAAGGTGGAAGTTATGCAACAACTAAAAATGCACAGTGCTGATC |
| Btr190II reverse | CTAGATCTTCCCCGGGTTATTTCACCAGACTTATCAATCCTTCAAAATC |
| M.Btr192II forward | AAACTGCAGGTAAGGTTAATCATATGCAACAACTAAAAATGCACAGTGCTGATCAAACC |
| M.Btr192II reverse | AAAGGATCCTTAAATCACATTCAATCCTGTATTTGGGCAAATCTGTTTAAAAATCTGC |
| M.Btr188III forward | AAACTGCAGTTAAGGTTAATCATATGCCAAACAGCACAGCACAGCACAGCACAGC |
| M.Btr188III reverse | AAAGGATCCACGGTTTCCCGTAAATTAGGGTGTTTATAAGTGGATTTCTCCACTTAATAA |
| Btr192 ORF21780 forward | AAACCTGCAGGTAAGGTTAATCATATGTCAAAAAAATTACTCTTTCTTGATTTATTTGC |
| Btr192 ORF21780 reverse | AAAGGATCCTTATATCCATTTTTCCCTAAGTTTTTCAGCAATTTTTCTAGCTAATAATAC |
| M.Btr192IV forward | AAACTGCAGTTAAGGTTAATCATATGAAGAAAACTTTAACCGCATTAGCTGTTGCTTC |
| M.Btr192IV reverse | AAAGGATCCTTAGAATTTATAAACCATATTATCCTCATACTTGCCGTTATAACTAGC |
| Btr190III forward | GCAGGTAAGGTGGAAGTTATGAGTGATTTTGATAGAGATAGTTATCG |
| Btr190III reverse | CTAGATCTTCCCCGGGTTATTTTCCCCACTGCTTAATAAAATCCAAGCT |
| Btr190 ORF12260 forward | AAACTGCAGTTAAGGTTAATCATATGTTAGCGTGGAAATTAGAAAATGACG |
| Btr190 ORF12260 reverse | AAAGGATCCGAGTCGGAAGTCTTAAACGAGCAAGTTATCGTTATCGGTAATGGCTTGC |
| Btr190 ORF20600 forward | GCAGGTAAGGTGGAAGTTATGCTGGATTTAGTCGAAGCCCAAACCGAAC |
| Btr190 ORF20600 reverse | CTAGATCTTCCCCGGGTTACT CATCTCCCAACTCCATCGGTCTAACC |
| AACGC forward | TTTTGCGCGGTTTGATCAGC |
| AACGC reverse | CCGTTTCTGGCGCATTGATT |
| AAGC forward | GCTCCGAGCGTTATGGTTGT |
| AAGC reverse | AACCAACCGCACGAGAAGAT |
| Leukotoxin forward | CTCTGCTTCACGGAACCAGT |
| Leukotoxin reverse | GCCGAGCGTGTAATTGCAAT |
