# Supplementary material for: Comparative Methylome Analysis of the Occasional Ruminant Respiratory Pathogen Bibersteinia trehalosi
Source: PLoS One. 2016 Aug 24;11(8):e0161499. doi: 10.1371/journal.pone.0161499 (PMC4996451; doi:10.1371/journal.pone.0161499)
Supplement: S2 Table — (DOCX) [file pone.0161499.s005.docx]

**Supplementary Table S2.** ORFs in at least three of the four *B. trehalosi* strains.^a^

|  | Present in all strains *except*: | | | |  |
| --- | --- | --- | --- | --- | --- |
| Number of strains | 188 | 189 | 190 | 192 | Total |
| 4 | – | – | – | – | 1963 *(4)* |
| 3 | 26 | 5 | 139 *(2)* | 1 | 171 *(2)* |

^a^ In italics and parentheses is the number of these ORFs that are MTases (i.e., represented in Table 4).
