# Supplementary material for: Comparative Methylome Analysis of the Occasional Ruminant Respiratory Pathogen Bibersteinia trehalosi
Source: PLoS One. 2016 Aug 24;11(8):e0161499. doi: 10.1371/journal.pone.0161499 (PMC4996451; doi:10.1371/journal.pone.0161499)
Supplement: S3 Table — (DOCX) [file pone.0161499.s006.docx]

**Supplementary Table S3.** ORFs in at most two of the four *B. trehalosi* strains.^a,b^

| Strain 1 | Strain 2 | | | | Total (1 strain)^c^ | Total (2 strains)^d^ |
| --- | --- | --- | --- | --- | --- | --- |
|  | 188 | 189 | 190 | 192 |  |  |
| 188 | **20** | 7 | 7 | 3 | 20 [14] | 17 |
| 189 | 7 | **75** | 60 *(1)* | 100 *(1)* | 75 [9] | 167 *(2)* |
| 190 | 7 | 60 *(1)* | **228** *(1)* | 5 | 228 *(1)* [179] | 72 *(1)* |
| 192 | 3 | 100 *(1)* | 5 | **11** | 11 [6] | 108 *(1)* |
| Total |  |  |  |  | 334 [208] | 182 *(2)* |

^a^ In italics and parentheses is the number of these ORFs that are MTases (i.e., represented in Table 4).

^b^ Table shows ORFs shared only by Strain 1 and Strain 2, but no other strains. When Strain 1 is the same as Strain 2, the number indicates the ORFs that have no reciprocal best BLAST hit to another strain (boldface type).

^c^ Total ORFs without reciprocal best BLAST hit for Strain 1 (i.e., “non-orthologous”); same as the boldface entry in this row. Shown in brackets is the subset of these ORFs that are “singletons” that have no BLAST hit to an ORF in another strain and may be considered unique to Strain 1.

^d^ Total ORFs in Strain 1 shared by only one other strain; sum of the Roman-type entries in this row. The grand total in the bottom row is non-redundant, so is the column sum x 0.5.
