# Supplementary material for: Comparative Methylome Analysis of the Occasional Ruminant Respiratory Pathogen Bibersteinia trehalosi
Source: PLoS One. 2016 Aug 24;11(8):e0161499. doi: 10.1371/journal.pone.0161499 (PMC4996451; doi:10.1371/journal.pone.0161499)
Supplement: S4 Table — (PDF) [file pone.0161499.s007.pdf]

Number_of_Genomes Sharing Orthologs	Bt_188_Locus	Bt_188_Locus_Name	Bt_189_Locus	Bt_189_Locus_Name	Bt_190_Locus	Bt_190_Locus_Name	Bt_192_Locus	Bt_192_Locus_Name
4	FS42_10	Plant lojap-like protein	FS43_20	Plant lojap-like protein	FS44_20	Plant lojap-like protein	WQG_22600	hypothetical protein
4	FS42_100	Signal recognition particle protein	FS43_110	Signal recognition particle protein	FS44_23030	Signal recognition particle protein	WQG_22510	Signal recognition particle protein
4	FS42_1000	Peptide deformylase	FS43_1030	Peptide deformylase	FS44_22140	Peptide deformylase	WQG_21600	Peptide deformylase
4	FS42_10000	hypothetical protein	FS43_11400	hypothetical protein	FS44_12430	hypothetical protein	WQG_12040	hypothetical protein
4	FS42_10010	Acetolactate synthase small subunit	FS43_11410	Acetolactate synthase small subunit	FS44_12420	Acetolactate synthase small subunit	WQG_12030	Acetolactate synthase small subunit
4	FS42_10020	Acetolactate synthase isozyme 3 large subunit	FS43_11420	Acetolactate synthase isozyme 3 large subunit	FS44_12410	Acetolactate synthase isozyme 3 large subunit	WQG_12020	Acetolactate synthase isozyme 3 large subunit
4	FS42_10030	NADP-dependent malic enzyme	FS43_11430	NADP-dependent malic enzyme	FS44_12400	NADP-dependent malic enzyme	WQG_12010	NADP-dependent malic enzyme
4	FS42_10040	Hemolysin activation/secretion protein	FS43_11440	Hemolysin activation/secretion protein	FS44_12390	Hemolysin activation/secretion protein	WQG_12000	Hemolysin activation/secretion protein
4	FS42_10050	Large exoprotein involved in heme utilizationor adhesion	FS43_11450	Large exoprotein involved in heme utilizationor adhesion	FS44_12370	Large exoprotein involved in heme utilizationor adhesion	WQG_11990	Large exoprotein involved in heme utilizationor adhesion
4	FS42_10090	soluble lytic murein transglycosylase	FS43_11490	soluble lytic murein transglycosylase	FS44_12290	soluble lytic murein transglycosylase	WQG_11960	soluble lytic murein transglycosylase
4	FS42_1010	Catabolite gene activator protein	FS43_1040	Catabolite gene activator protein	FS44_22130	Catabolite gene activator protein	WQG_21590	Catabolite gene activator
4	FS42_10100	Hypoxanthine phosphoribosyltransferase	FS43_11500	Hypoxanthine phosphoribosyltransferase	FS44_12280	Hypoxanthine phosphoribosyltransferase	WQG_11950	Hypoxanthine phosphoribosyltransferase
4	FS42_10110	Asparaginyl-tRNA synthetase	FS43_11510	Asparaginyl-tRNA synthetase	FS44_12270	Asparaginyl-tRNA synthetase	WQG_11940	Asparaginyl-tRNA synthetase
4	FS42_10170	Bifunctional protein GlmU	FS43_11570	Bifunctional protein GlmU	FS44_12240	Bifunctional protein GlmU	WQG_11880	Bifunctional protein GlmU
4	FS42_10180	30S ribosomal protein S2	FS43_11580	30S ribosomal protein S2	FS44_12230	30S ribosomal protein S2	WQG_11870	30S ribosomal protein S2
4	FS42_10190	Elongation factor Ts	FS43_11590	Elongation factor Ts	FS44_12220	Elongation factor Ts	WQG_11860	Elongation factor Ts
4	FS42_1020	Cytochrome c biogenesis factor-like protein	FS43_1050	Cytochrome c biogenesis factor-like protein	FS44_22120	Cytochrome c biogenesis factor-like protein	WQG_21580	Cytochrome c biogenesis factor-like protein
4	FS42_10200	Uridylate kinase	FS43_11600	Uridylate kinase	FS44_12210	Uridylate kinase	WQG_11850	Uridylate kinase
4	FS42_10210	Transcriptional regulatory protein tyrR	FS43_11610	Transcriptional regulatory protein tyrR	FS44_12200	Transcriptional regulatory protein tyrR	WQG_11840	Transcriptional regulatory protein tyrR
4	FS42_10220	Peptide transport periplasmic protein SapA	FS43_11620	Peptide transport periplasmic protein SapA	FS44_12190	Peptide transport periplasmic protein SapA	WQG_11830	Peptide transport periplasmic protein SapA
4	FS42_10230	Peptide transport system permease protein SapB	FS43_11630	Peptide transport system permease protein SapB	FS44_12180	Peptide transport system permease protein SapB	WQG_11820	Peptide transport system permease protein SapB
4	FS42_10240	Binding-protein-dependent transport system inner membrane component	FS43_11640	Binding-protein-dependent transport system inner membrane component	FS44_12170	Binding-protein-dependent transport system inner membrane component	WQG_11810	Binding-protein-dependent transport system inner membrane component
4	FS42_10250	Peptide transport system ATP-binding protein SapD	FS43_11650	Peptide transport system ATP-binding protein SapD	FS44_12160	Peptide transport system ATP-binding protein SapD	WQG_11800	Peptide transport system ATP-binding protein SapD
4	FS42_10260	DNA topoisomerase 1	FS43_11660	DNA topoisomerase 1	FS44_12150	DNA topoisomerase 1	WQG_11790	DNA topoisomerase 1
4	FS42_10270	Transposase, Mutator	FS43_11670	Transposase, Mutator	FS44_12140	Transposase, Mutator	WQG_11780	Transposase, Mutator
4	FS42_10290	4-hydroxybenzoate octaprenyltransferase	FS43_11690	4-hydroxybenzoate octaprenyltransferase	FS44_12130	4-hydroxybenzoate octaprenyltransferase	WQG_11760	4-hydroxybenzoate octaprenyltransferase
4	FS42_1030	cytochrome c-type biogenesis protein CcmH	FS43_1060	cytochrome c-type biogenesis protein CcmH	FS44_22110	cytochrome c-type biogenesis protein CcmH	WQG_21570	cytochrome c-type biogenesis protein CcmH
4	FS42_10300	Glycerol-3-phosphate regulon repressor	FS43_11700	Glycerol-3-phosphate regulon repressor	FS44_12120	Glycerol-3-phosphate regulon repressor	WQG_11750	Glycerol-3-phosphate regulon repressor
4	FS42_10310	Adenylosuccinate lyase	FS43_11710	Adenylosuccinate lyase	FS44_12110	Adenylosuccinate lyase	WQG_11740	Adenylosuccinate lyase
4	FS42_10320	High frequency lysogenization protein HflD	FS43_11720	High frequency lysogenization protein HflD	FS44_12100	High frequency lysogenization protein HflD	WQG_11730	High frequency lysogenization protein HflD
4	FS42_10330	ATP-dependent protease La	FS43_11730	ATP-dependent protease La	FS44_12090	ATP-dependent protease La	WQG_11720	ATP-dependent protease La
4	FS42_10340	SsrA-binding protein	FS43_11740	SsrA-binding protein	FS44_12080	SsrA-binding protein	WQG_11710	SsrA-binding protein
4	FS42_10350	Seryl-tRNA synthetase	FS43_11750	Seryl-tRNA synthetase	FS44_12070	Seryl-tRNA synthetase	WQG_11700	Seryl-tRNA synthetase
4	FS42_10360	Protein FdhE	FS43_11760	Protein FdhE	FS44_12060	Protein FdhE	WQG_11690	Protein FdhE
4	FS42_10380	bifunctional amino acid-binding protein/DNA-binding protein	FS43_11780	bifunctional amino acid-binding protein/DNA-binding protein	FS44_12050	bifunctional amino acid-binding protein/DNA-binding protein	WQG_11670	bifunctional amino acid-binding protein/DNA-binding protein
4	FS42_10390	Cytochrome c-type protein TorY	FS43_11790	Cytochrome c-type protein TorY	FS44_12040	Cytochrome c-type protein TorY	WQG_11660	Cytochrome c-type protein TorY
4	FS42_1040	Thiol:disulfide interchange protein DsbE	FS43_1070	Thiol:disulfide interchange protein DsbE	FS44_22100	Thiol:disulfide interchange protein DsbE	WQG_21560	Thiol:disulfide interchange protein DsbE
4	FS42_10400	hypothetical protein	FS43_11800	hypothetical protein	FS44_12030	hypothetical protein	WQG_11650	hypothetical protein
4	FS42_10410	hypothetical protein	FS43_11810	hypothetical protein	FS44_12020	hypothetical protein	WQG_11640	hypothetical protein
4	FS42_10420	Methionine synthase	FS43_11820	Methionine synthase	FS44_12010	Methionine synthase	WQG_11630	Methionine synthase
4	FS42_10430	Trimethylamine-N-oxide reductase	FS43_11830	Trimethylamine-N-oxide reductase	FS44_12000	Trimethylamine-N-oxide reductase	WQG_11620	Trimethylamine-N-oxide reductase
4	FS42_10440	Endonuclease III	FS43_11840	Endonuclease III	FS44_11990	Endonuclease III	WQG_11610	Endonuclease III
4	FS42_10450	UPF0721 transmembrane protein	FS43_11850	UPF0721 transmembrane protein	FS44_11980	UPF0721 transmembrane protein	WQG_11600	UPF0721 transmembrane protein
4	FS42_10460	Glutamate 5-kinase	FS43_11860	Glutamate 5-kinase	FS44_11970	Glutamate 5-kinase	WQG_11590	Glutamate 5-kinase
4	FS42_10470	hypothetical protein	FS43_11870	hypothetical protein	FS44_11960	hypothetical protein	WQG_11580	hypothetical protein
4	FS42_10480	Transcriptional regulator acrR protein	FS43_11880	Transcriptional regulator acrR protein	FS44_11950	Transcriptional regulator acrR protein	WQG_11570	Transcriptional regulator acrR protein
4	FS42_10490	Membrane-fusion protein	FS43_11900	Membrane-fusion protein	FS44_11940	Membrane-fusion protein	WQG_11550	Membrane-fusion protein
4	FS42_1050	membrane protein	FS43_1080	membrane protein	FS44_22090	membrane protein	WQG_21550	membrane protein
4	FS42_10500	Acriflavine resistance protein	FS43_11910	Acriflavine resistance protein	FS44_11930	Acriflavine resistance protein	WQG_11540	Acriflavine resistance protein
4	FS42_10510	hypothetical protein	FS43_11920	hypothetical protein	FS44_11920	hypothetical protein	WQG_11530	hypothetical protein
4	FS42_10530	Site-specific DNA-methyltransferase	FS43_11930	Site-specific DNA-methyltransferase	FS44_11900	Type III restriction-modification system methyltransferase	WQG_11520	Site-specific DNA-methyltransferase
4	FS42_10540	Type III restriction-modification system restriction subunit	FS43_11940	Type III restriction-modification system restriction subunit	FS44_11890	Type III restriction-modification system restriction subunit	WQG_11510	Type III restriction-modification system restriction subunit
4	FS42_10550	esterase/lipase	FS43_11950	esterase/lipase	FS44_11880	esterase/lipase	WQG_11500	esterase/lipase
4	FS42_10560	DNA replication inhibitor protein	FS43_11960	DNA replication inhibitor protein	FS44_11870	DNA replication inhibitor protein	WQG_11490	DNA replication inhibitor protein
4	FS42_10570	O-succinylbenzoic acid--CoA ligase	FS43_11970	O-succinylbenzoic acid--CoA ligase	FS44_11860	O-succinylbenzoic acid--CoA ligase	WQG_11480	O-succinylbenzoic acid--CoA ligase
4	FS42_10580	Potassium efflux protein KefA, small-conductance mechanosensitive channel	FS43_11980	Potassium efflux protein KefA, small-conductance mechanosensitive channel	FS44_11850	Potassium efflux protein KefA, small-conductance mechanosensitive channel	WQG_11470	Potassium efflux protein KefA, small-conductance mechanosensitive channel
4	FS42_10590	Chorismate synthase	FS43_12010	Chorismate synthase	FS44_11840	Chorismate synthase	WQG_11460	Chorismate synthase
4	FS42_1060	Tyrosine recombinase xerD	FS43_1090	Tyrosine recombinase xerD	FS44_22080	Tyrosine recombinase xerD	WQG_21540	Tyrosine recombinase xerD
4	FS42_10600	hypothetical protein	FS43_12020	hypothetical protein	FS44_11830	hypothetical protein	WQG_11450	hypothetical protein
4	FS42_10610	Outer membrane protein P2-like protein	FS43_12030	Outer membrane protein P2-like protein	FS44_11820	Outer membrane protein P2-like protein	WQG_11440	Outer membrane protein P2-like protein
4	FS42_10620	2-C-methyl-D-erythritol 2,4-cyclodiphosphate synthase	FS43_12040	2-C-methyl-D-erythritol 2,4-cyclodiphosphate synthase	FS44_11810	2-C-methyl-D-erythritol 2,4-cyclodiphosphate synthase	WQG_11430	2-C-methyl-D-erythritol 2,4-cyclodiphosphate synthase
4	FS42_10630	2-C-methyl-D-erythritol 4-phosphate cytidylyltransferase	FS43_12050	2-C-methyl-D-erythritol 4-phosphate cytidylyltransferase	FS44_11800	2-C-methyl-D-erythritol 4-phosphate cytidylyltransferase	WQG_11420	2-C-methyl-D-erythritol 4-phosphate cytidylyltransferase
4	FS42_10640	Cell division protein FtsB	FS43_12060	Cell division protein FtsB	FS44_11790	Cell division protein FtsB	WQG_11410	Cell division protein FtsB
4	FS42_10650	Phosphoenolpyruvate carboxykinase	FS43_12070	Phosphoenolpyruvate carboxykinase	FS44_11780	Phosphoenolpyruvate carboxykinase	WQG_11400	Phosphoenolpyruvate carboxykinase
4	FS42_10660	Asparaginase	FS43_12080	Asparaginase	FS44_11760	Asparaginase	WQG_11390	Asparaginase
4	FS42_10680	Pyruvate dehydrogenase E1 component	FS43_12090	Pyruvate dehydrogenase E1 component	FS44_11750	Pyruvate dehydrogenase E1 component	WQG_11380	Pyruvate dehydrogenase E1 component
4	FS42_10690	Dihydrolipoamide acetyltransferase	FS43_12100	Dihydrolipoamide acetyltransferase	FS44_11740	Dihydrolipoamide acetyltransferase	WQG_11370	Dihydrolipoamide acetyltransferase
4	FS42_1070	hypothetical protein	FS43_1100	hypothetical protein	FS44_22070	hypothetical protein	WQG_21530	hypothetical protein
4	FS42_10700	Dihydrolipoyl dehydrogenase	FS43_12130	Dihydrolipoyl dehydrogenase	FS44_11730	Dihydrolipoyl dehydrogenase	WQG_11360	Dihydrolipoyl dehydrogenase
4	FS42_10710	Universal stress protein A	FS43_12140	Universal stress protein A	FS44_11720	Universal stress protein A	WQG_11350	Universal stress protein A
4	FS42_10720	hypothetical protein	FS43_12150	hypothetical protein	FS44_11710	hypothetical protein	WQG_11340	hypothetical protein
4	FS42_10730	Alanyl-tRNA synthetase	FS43_12160	Alanyl-tRNA synthetase	FS44_11700	Alanyl-tRNA synthetase	WQG_11330	Alanyl-tRNA synthetase
4	FS42_10740	Carbon storage regulator	FS43_12170	Carbon storage regulator	FS44_11690	Carbon storage regulator	WQG_11320	Carbon storage regulator
4	FS42_10750	UTP--glucose-1-phosphate uridylyltransferase	FS43_12180	UTP--glucose-1-phosphate uridylyltransferase	FS44_11680	UTP--glucose-1-phosphate uridylyltransferase	WQG_11310	UTP--glucose-1-phosphate uridylyltransferase
4	FS42_10760	Serine endoprotease DegS	FS43_12190	Serine endoprotease DegS	FS44_11670	Serine endoprotease DegS	WQG_11300	Serine endoprotease DegS
4	FS42_10770	Cytidylate kinase	FS43_12200	Cytidylate kinase	FS44_11660	Cytidylate kinase	WQG_11290	Cytidylate kinase
4	FS42_10780	30S ribosomal protein S1	FS43_12210	30S ribosomal protein S1	FS44_11650	30S ribosomal protein S1	WQG_11280	30S ribosomal protein S1
4	FS42_10790	Integration host factor subunit beta	FS43_12220	Integration host factor subunit beta	FS44_11640	Integration host factor subunit beta	WQG_11270	Integration host factor subunit beta
4	FS42_1080	NADPH-dependent FMN reductase	FS43_1110	NADPH-dependent FMN reductase	FS44_22060	NADPH-dependent FMN reductase	WQG_21520	NADPH-dependent FMN reductase
4	FS42_10800	Predictive membrane protein	FS43_12230	Predictive membrane protein	FS44_11630	Predictive membrane protein	WQG_11260	Predictive membrane protein
4	FS42_10810	hypothetical protein	FS43_12240	hypothetical protein	FS44_11620	hypothetical protein	WQG_11250	hypothetical protein
4	FS42_10820	Orotidine 5'-phosphate decarboxylase	FS43_12250	Orotidine 5'-phosphate decarboxylase	FS44_11610	Orotidine 5'-phosphate decarboxylase	WQG_11240	Orotidine 5'-phosphate decarboxylase
4	FS42_10830	hypothetical protein	FS43_12260	hypothetical protein	FS44_11600	hypothetical protein	WQG_11230	hypothetical protein
4	FS42_10840	ATP-dependent dethiobiotin synthetase BioD 1	FS43_12270	ATP-dependent dethiobiotin synthetase BioD 1	FS44_11590	ATP-dependent dethiobiotin synthetase BioD 1	WQG_11220	ATP-dependent dethiobiotin synthetase BioD 1
4	FS42_10850	Transcriptional repressor of carbohydrate metabolism	FS43_12280	Transcriptional repressor of carbohydrate metabolism	FS44_11580	Transcriptional repressor of carbohydrate metabolism	WQG_11210	Transcriptional repressor of carbohydrate metabolism
4	FS42_10860	Ribonuclease D	FS43_12290	Ribonuclease D	FS44_11570	Ribonuclease D	WQG_11200	Ribonuclease D
4	FS42_10870	Acetate kinase	FS43_12300	Acetate kinase	FS44_11560	Acetate kinase	WQG_11190	Acetate kinase
4	FS42_10880	Phosphate acetyltransferase	FS43_12310	Phosphate acetyltransferase	FS44_11550	Phosphate acetyltransferase	WQG_11180	Phosphate acetyltransferase
4	FS42_10890	D-lactate dehydrogenase	FS43_12320	D-lactate dehydrogenase	FS44_11540	D-lactate dehydrogenase	WQG_11170	D-lactate dehydrogenase
4	FS42_1090	quercetin 2,3-dioxygenase yhhW	FS43_1120	quercetin 2,3-dioxygenase yhhW	FS44_22050	quercetin 2,3-dioxygenase yhhW	WQG_21510	quercetin 2,3-dioxygenase yhhW
4	FS42_10900	3-phosphoshikimate 1-carboxyvinyltransferase	FS43_12330	3-phosphoshikimate 1-carboxyvinyltransferase	FS44_11530	3-phosphoshikimate 1-carboxyvinyltransferase	WQG_11160	3-phosphoshikimate 1-carboxyvinyltransferase
4	FS42_10910	Histidinol-phosphate aminotransferase 1	FS43_12340	Histidinol-phosphate aminotransferase 1	FS44_11520	Histidinol-phosphate aminotransferase 1	WQG_11150	Histidinol-phosphate aminotransferase 1
4	FS42_10920	Phosphoribosylaminoimidazole-succinocarboxamide synthase	FS43_12350	Phosphoribosylaminoimidazole-succinocarboxamide synthase	FS44_11510	Phosphoribosylaminoimidazole-succinocarboxamide synthase	WQG_11140	Phosphoribosylaminoimidazole-succinocarboxamide synthase
4	FS42_10930	Amidophosphoribosyltransferase	FS43_12360	Amidophosphoribosyltransferase	FS44_11500	Amidophosphoribosyltransferase	WQG_11130	Amidophosphoribosyltransferase
4	FS42_10940	Phosphoribosylformylglycinamidine cyclo-ligase	FS43_12380	Phosphoribosylformylglycinamidine cyclo-ligase	FS44_11490	Phosphoribosylformylglycinamidine cyclo-ligase	WQG_11120	Phosphoribosylformylglycinamidine cyclo-ligase
4	FS42_10950	Phosphoribosylglycinamide formyltransferase	FS43_12390	Phosphoribosylglycinamide formyltransferase	FS44_11480	Phosphoribosylglycinamide formyltransferase	WQG_11110	Phosphoribosylglycinamide formyltransferase
4	FS42_10960	Bifunctional purine biosynthesis protein PurH	FS43_12400	Bifunctional purine biosynthesis protein PurH	FS44_11470	Bifunctional purine biosynthesis protein PurH	WQG_11100	Bifunctional purine biosynthesis protein PurH
4	FS42_10970	Phosphoribosylamine--glycine ligase	FS43_12410	Phosphoribosylamine--glycine ligase	FS44_11460	Phosphoribosylamine--glycine ligase	WQG_11090	Phosphoribosylamine--glycine ligase
4	FS42_10980	hypothetical protein	FS43_12420	hypothetical protein	FS44_11450	hypothetical protein	WQG_11080	hypothetical protein
4	FS42_10990	Phosphoribosylaminoimidazole carboxylase catalytic subunit	FS43_12430	Phosphoribosylaminoimidazole carboxylase catalytic subunit	FS44_11440	Phosphoribosylaminoimidazole carboxylase catalytic subunit	WQG_11070	Phosphoribosylaminoimidazole carboxylase catalytic subunit
4	FS42_110	Phosphotransferase	FS43_120	Phosphotransferase	FS44_23020	Phosphotransferase	WQG_22500	Phosphotransferase
4	FS42_1100	LysR protein	FS43_1130	LysR protein	FS44_22040	LysR protein	WQG_21500	LysR protein
4	FS42_11000	5-(Carboxyamino)imidazole ribonucleotide synthase	FS43_12440	5-(Carboxyamino)imidazole ribonucleotide synthase	FS44_11430	Phosphoribosylaminoimidazole carboxylase, ATPase subunit	WQG_11060	5-(Carboxyamino)imidazole ribonucleotide synthase
4	FS42_11010	hypothetical protein	FS43_12460	hypothetical protein	FS44_11420	hypothetical protein	WQG_11050	hypothetical protein
4	FS42_11020	Phosphoserine aminotransferase	FS43_12480	Phosphoserine aminotransferase	FS44_11410	Phosphoserine aminotransferase	WQG_11040	Phosphoserine aminotransferase
4	FS42_11040	Xaa-Pro aminopeptidase	FS43_12500	Xaa-Pro aminopeptidase	FS44_11390	Xaa-Pro aminopeptidase	WQG_11020	Xaa-Pro aminopeptidase
4	FS42_11050	DNA topoisomerase 3	FS43_12510	DNA topoisomerase 3	FS44_11380	DNA topoisomerase 3	WQG_11010	DNA topoisomerase 3
4	FS42_11060	Penicillin-insensitive murein endopeptidase	FS43_12520	Penicillin-insensitive murein endopeptidase	FS44_11370	Penicillin-insensitive murein endopeptidase	WQG_11000	Penicillin-insensitive murein endopeptidase
4	FS42_11070	Lysine-sensitive aspartokinase 3	FS43_12530	Lysine-sensitive aspartokinase 3	FS44_11360	Lysine-sensitive aspartokinase 3	WQG_10990	Lysine-sensitive aspartokinase 3
4	FS42_11080	hypothetical protein	FS43_12540	hypothetical protein	FS44_11350	hypothetical protein	WQG_10980	hypothetical protein
4	FS42_11090	Phospho-2-dehydro-3-deoxyheptonate aldolase	FS43_12550	Phospho-2-dehydro-3-deoxyheptonate aldolase	FS44_11340	Phospho-2-dehydro-3-deoxyheptonate aldolase	WQG_10970	Phospho-2-dehydro-3-deoxyheptonate aldolase
4	FS42_1110	Glutamate racemase	FS43_1140	Glutamate racemase	FS44_22030	Glutamate racemase	WQG_21490	Glutamate racemase
4	FS42_11100	protein ypfJ	FS43_12560	protein ypfJ	FS44_11330	protein ypfJ	WQG_10960	protein ypfJ
4	FS42_11110	DNA translocase FtsK involved in cell division, DNA segregation ATPase FtsK	FS43_12570	DNA translocase FtsK involved in cell division, DNA segregation ATPase FtsK	FS44_11320	DNA translocase FtsK involved in cell division, DNA segregation ATPase FtsK	WQG_10950	DNA translocase FtsK involved in cell division, DNA segregation ATPase FtsK
4	FS42_11120	Leucine-responsive regulatory protein	FS43_12580	Leucine-responsive regulatory protein	FS44_11310	Leucine-responsive regulatory protein	WQG_10940	Leucine-responsive regulatory protein
4	FS42_11130	SdaC protein	FS43_12590	SdaC protein	FS44_11300	SdaC protein	WQG_10930	SdaC protein
4	FS42_11140	metalloprotease	FS43_12600	metalloprotease	FS44_11290	metalloprotease	WQG_10920	metalloprotease
4	FS42_11150	Ribosomal RNA large subunit methyltransferase E	FS43_12610	Ribosomal RNA large subunit methyltransferase E	FS44_11280	Ribosomal RNA large subunit methyltransferase E	WQG_10910	Ribosomal RNA large subunit methyltransferase E
4	FS42_11160	reductase	FS43_12620	reductase	FS44_11270	reductase	WQG_10900	reductase
4	FS42_11170	Exoribonuclease 2	FS43_12630	Exoribonuclease 2	FS44_11260	Exoribonuclease 2	WQG_10890	Exoribonuclease 2
4	FS42_11180	Iron-regulated outer membrane protein/TonB-dependent receptor	FS43_12640	Iron-regulated outer membrane protein/TonB-dependent receptor	FS44_11250	Iron-regulated outer membrane protein/TonB-dependent receptor	WQG_10880	Iron-regulated outer membrane protein/TonB-dependent receptor
4	FS42_11190	hypothetical protein	FS43_12650	hypothetical protein	FS44_11240	hypothetical protein	WQG_10870	hypothetical protein
4	FS42_1120	4-hydroxybenzoate synthetase (Chorismate lyase)	FS43_1150	4-hydroxybenzoate synthetase (Chorismate lyase)	FS44_22020	4-hydroxybenzoate synthetase (Chorismate lyase)	WQG_21480	4-hydroxybenzoate synthetase (Chorismate lyase)
4	FS42_11200	outer membrane protein	FS43_12660	outer membrane protein	FS44_11230	outer membrane protein	WQG_10860	outer membrane protein
4	FS42_11210	Bis(5'-nucleosyl)-tetraphosphatase, symmetrical	FS43_12670	Bis(5'-nucleosyl)-tetraphosphatase, symmetrical	FS44_11220	Bis(5'-nucleosyl)-tetraphosphatase, symmetrical	WQG_10850	Bis(5'-nucleosyl)-tetraphosphatase, symmetrical
4	FS42_11220	Nicotinamide phosphoribosyl transferase	FS43_12680	Nicotinamide phosphoribosyl transferase	FS44_11210	Nicotinamide phosphoribosyl transferase	WQG_10840	Nicotinamide phosphoribosyl transferase
4	FS42_11230	Transcription elongation factor greA	FS43_12690	Transcription elongation factor greA	FS44_11200	Transcription elongation factor greA	WQG_10830	Transcription elongation factor greA
4	FS42_11240	Exodeoxyribonuclease 7 large subunit	FS43_12700	Exodeoxyribonuclease 7 large subunit	FS44_11190	Exodeoxyribonuclease 7 large subunit	WQG_10820	Exodeoxyribonuclease 7 large subunit
4	FS42_11250	Twin-arginine leader-binding protein DmsD	FS43_12710	Twin-arginine leader-binding protein DmsD	FS44_11180	Twin-arginine leader-binding protein DmsD	WQG_10810	Twin-arginine leader-binding protein DmsD
4	FS42_11260	hypothetical protein	FS43_12720	hypothetical protein	FS44_11170	hypothetical protein	WQG_10800	hypothetical protein
4	FS42_11270	reductase	FS43_12730	reductase	FS44_11160	reductase	WQG_10790	reductase
4	FS42_11280	reductase	FS43_12740	reductase	FS44_11150	reductase	WQG_10780	reductase
4	FS42_11290	reductase	FS43_12750	reductase	FS44_11140	DmsC	WQG_10770	reductase
4	FS42_1130	Acylneuraminate cytidylyltransferase	FS43_1160	Acylneuraminate cytidylyltransferase	FS44_22010	Acylneuraminate cytidylyltransferase	WQG_21470	Acylneuraminate cytidylyltransferase
4	FS42_11330	Histidine kinase	FS43_12800	Histidine kinase	FS44_11110	Histidine kinase	WQG_10730	Histidine kinase
4	FS42_11340	Molybdenum cofactor biosynthesis protein A	FS43_12810	Molybdenum cofactor biosynthesis protein A	FS44_11100	Molybdenum cofactor biosynthesis protein A	WQG_10720	Molybdenum cofactor biosynthesis protein A
4	FS42_11350	Molybdenum cofactor biosynthesis protein C	FS43_12830	Molybdenum cofactor biosynthesis protein C	FS44_11090	Molybdenum cofactor biosynthesis protein C	WQG_10710	Molybdenum cofactor biosynthesis protein C
4	FS42_11360	RNA methyltransferase, TrmH family, group 1	FS43_12840	RNA methyltransferase, TrmH family, group 1	FS44_11080	RNA methyltransferase, TrmH family, group 1	WQG_10700	RNA methyltransferase, TrmH family, group 1

4	**FS42_11370**	ABC transporter, substrate-binding protein	FS43_12850	ABC transporter, substrate-binding protein	FS44_11070	ABC transporter, substrate-binding protein	WQG_10690	ABC transporter, substrate-binding protein
4	**FS42_11380**	Stringent starvation protein B	FS43_12860	Stringent starvation protein B	FS44_11060	Stringent starvation protein B	WQG_10680	Stringent starvation protein B
4	**FS42_11390**	Stringent starvation protein A	FS43_12870	Stringent starvation protein A	FS44_11050	Stringent starvation protein A	WQG_10670	Stringent starvation protein A
4	**FS42_11400**	hypothetical protein	FS43_12880	hypothetical protein	FS44_11040	hypothetical protein	WQG_10660	hypothetical protein
4	**FS42_11410**	Lytic transglycosylase	FS43_12890	Lytic transglycosylase	FS44_11030	Lytic transglycosylase	WQG_10650	Lytic transglycosylase
4	**FS42_11420**	tRNA pseudouridine synthase B	FS43_12900	tRNA pseudouridine synthase B	FS44_11020	tRNA pseudouridine synthase B	WQG_10640	tRNA pseudouridine synthase B
4	**FS42_11430**	Ribosome-binding factor A	FS43_12910	Ribosome-binding factor A	FS44_11010	Ribosome-binding factor A	WQG_10630	Ribosome-binding factor A
4	**FS42_11440**	ABC transporter ATP-binding protein	FS43_12920	ABC transporter ATP-binding protein	FS44_11000	ABC transporter ATP-binding protein	WQG_10620	ABC transporter ATP-binding protein
4	**FS42_11460**	thiamine biosynthesis protein	FS43_12940	thiamine biosynthesis protein	FS44_10990	thiamine biosynthesis protein	WQG_10600	thiamine biosynthesis protein
4	**FS42_11470**	Hydroxyethylthiazole kinase	FS43_12950	Hydroxyethylthiazole kinase	FS44_10980	Hydroxyethylthiazole kinase	WQG_10590	Hydroxyethylthiazole kinase
4	**FS42_11480**	Phosphomethylpyrimidine kinase	FS43_12960	Phosphomethylpyrimidine kinase	FS44_10970	Phosphomethylpyrimidine kinase	WQG_10580	Phosphomethylpyrimidine kinase
4	**FS42_11490**	Thiamine-phosphate pyrophosphorylase	FS43_12970	Thiamine-phosphate pyrophosphorylase	FS44_10960	Thiamine-phosphate pyrophosphorylase	WQG_10570	Thiamine-phosphate pyrophosphorylase
4	**FS42_1150**	Uridine phosphorylase	FS43_1180	Uridine phosphorylase	FS44_22000	Uridine phosphorylase	WQG_21450	Uridine phosphorylase
4	**FS42_11500**	metabolite transport protein	FS43_12980	metabolite transport protein	FS44_10950	metabolite transport protein	WQG_10560	metabolite transport protein
4	**FS42_11510**	Lysyl-tRNA synthetase	FS43_12990	Lysyl-tRNA synthetase	FS44_10940	Lysyl-tRNA synthetase	WQG_10550	Lysyl-tRNA synthetase
4	**FS42_11520**	DNA gyrase subunit B	FS43_13000	DNA gyrase subunit B	FS44_10930	DNA gyrase subunit B	WQG_10540	DNA gyrase subunit B
4	**FS42_11530**	Protein FdhD	FS43_13010	Protein FdhD	FS44_10910	Protein FdhD	WQG_10530	Protein FdhD
4	**FS42_11540**	Formate dehydrogenase major subunit	FS43_13020	Formate dehydrogenase major subunit	FS44_10900	Formate dehydrogenase major subunit	WQG_10520	Formate dehydrogenase major subunit
4	**FS42_11550**	Formate dehydrogenase, alpha subunit	FS43_13030	Formate dehydrogenase, alpha subunit	FS44_10890	Formate dehydrogenase, alpha subunit	WQG_10510	Formate dehydrogenase, alpha subunit
4	**FS42_11560**	Formate dehydrogenase iron-sulfur subunit	FS43_13040	Formate dehydrogenase iron-sulfur subunit	FS44_10880	Formate dehydrogenase iron-sulfur subunit	WQG_10500	Formate dehydrogenase iron-sulfur subunit
4	**FS42_11570**	Formate dehydrogenase (Quinone-dependent) cytochrome b subunit	FS43_13050	Formate dehydrogenase (Quinone-dependent) cytochrome b subunit	FS44_10870	Formate dehydrogenase (Quinone-dependent) cytochrome b subunit	WQG_10490	Formate dehydrogenase (Quinone-dependent) cytochrome b subunit
4	**FS42_11580**	ABC transporter, CydDC cysteine exporter	FS43_13060	ABC transporter, CydDC cysteine exporter	FS44_10860	ABC transporter, CydDC cysteine exporter	WQG_10480	ABC transporter, CydDC cysteine exporter
4	**FS42_11590**	ABC transporter, ATP-binding subunit	FS43_13070	ABC transporter, ATP-binding subunit	FS44_10850	ABC transporter, ATP-binding subunit	WQG_10470	ABC transporter, ATP-binding subunit
4	**FS42_1160**	TonB-dependent hemoglobin/transferrin/lactoferrin family receptor	FS43_1190	TonB-dependent hemoglobin/transferrin/lactoferrin family receptor	FS44_21980	TonB-dependent hemoglobin/transferrin/lactoferrin family receptor	WQG_21440	TonB-dependent hemoglobin/transferrin/lactoferrin family receptor
4	**FS42_11600**	ChvD family ATP-binding cassette protein	FS43_13080	ChvD family ATP-binding cassette protein	FS44_10840	ChvD family ATP-binding cassette protein	WQG_10460	ChvD family ATP-binding cassette protein
4	**FS42_11610**	Surface antigen (D15), outer membrane protein	FS43_13090	Surface antigen (D15), outer membrane protein	FS44_10830	Surface antigen (D15), outer membrane protein	WQG_10450	Surface antigen (D15), outer membrane protein
4	**FS42_11620**	membrane-bound metallopeptidase	FS43_13110	membrane-bound metallopeptidase	FS44_10820	membrane-bound metallopeptidase	WQG_10430	membrane-bound metallopeptidase
4	**FS42_11630**	30S ribosomal protein S9	FS43_13120	30S ribosomal protein S9	FS44_10810	30S ribosomal protein S9	WQG_10420	30S ribosomal protein S9
4	**FS42_11640**	50S ribosomal protein L13	FS43_13130	50S ribosomal protein L13	FS44_10800	50S ribosomal protein L13	WQG_10410	50S ribosomal protein L13
4	**FS42_11650**	hypothetical protein	FS43_13140	hypothetical protein	FS44_10790	hypothetical protein	WQG_10400	hypothetical protein
4	**FS42_11660**	Dithiobiotin synthetase	FS43_13150	Dithiobiotin synthetase	FS44_10780	Dithiobiotin synthetase	WQG_10390	Dithiobiotin synthetase
4	**FS42_11670**	Single-stranded DNA-binding protein	FS43_13160	Single-stranded DNA-binding protein	FS44_10770	Single-stranded DNA-binding protein	WQG_10380	Single-stranded DNA-binding protein
4	**FS42_11680**	UvrABC system protein A	FS43_13170	UvrABC system protein A	FS44_10760	UvrABC system protein A	WQG_10370	UvrABC system protein A
4	**FS42_11690**	reductase	FS43_13180	reductase	FS44_10740	reductase	WQG_10360	reductase
4	**FS42_1170**	Beta-lactamase fold protein	FS43_1200	Beta-lactamase fold protein	FS44_21970	Beta-lactamase fold protein	WQG_21430	Beta-lactamase fold protein
4	**FS42_11700**	reductase	FS43_13190	reductase	FS44_10730	reductase	WQG_10350	reductase
4	**FS42_11750**	UvrABC system protein B	FS43_13240	UvrABC system protein B	FS44_10710	UvrABC system protein B	WQG_10300	UvrABC system protein B
4	**FS42_11760**	Glutamate dehydrogenase	FS43_13250	Glutamate dehydrogenase	FS44_10700	Glutamate dehydrogenase	WQG_10290	Glutamate dehydrogenase
4	**FS42_11770**	hypothetical protein	FS43_13260	hypothetical protein	FS44_10690	hypothetical protein	WQG_10280	hypothetical protein
4	**FS42_11780**	3-demethylubiquinone-9 3-methyltransferase	FS43_13270	3-demethylubiquinone-9 3-methyltransferase	FS44_10680	3-demethylubiquinone-9 3-methyltransferase	WQG_10270	3-demethylubiquinone-9 3-methyltransferase
4	**FS42_11790**	protein yidQ	FS43_13280	protein yidQ	FS44_10670	protein yidQ	WQG_10260	protein yidQ
4	**FS42_1180**	Type I restriction-modification system restriction subunit	FS43_1210	Type I restriction-modification system restriction subunit	FS44_21960	Type I restriction-modification system restriction subunit	WQG_21420	Type I restriction-modification system restriction subunit
4	**FS42_11800**	3-isopropylmalate dehydrogenase	FS43_13290	3-isopropylmalate dehydrogenase	FS44_10660	3-isopropylmalate dehydrogenase	WQG_10250	3-isopropylmalate dehydrogenase
4	**FS42_11810**	hypothetical protein	FS43_13300	hypothetical protein	FS44_10650	hypothetical protein	WQG_10240	hypothetical protein
4	**FS42_11820**	TRAP-T family tripartite ATP-independent periplasmic transporter, binding protein	FS43_13320	TRAP-T family tripartite ATP-independent periplasmic transporter, binding protein	FS44_10640	TRAP-T family tripartite ATP-independent periplasmic transporter, binding protein	WQG_10230	TRAP-T family tripartite ATP-independent periplasmic transporter, binding protein
4	**FS42_11830**	TRAP-T family transporter	FS43_13330	TRAP-T family transporter	FS44_10630	TRAP-T family transporter	WQG_10220	TRAP-T family transporter
4	**FS42_11840**	hypothetical protein	FS43_13340	hypothetical protein	FS44_10620	hypothetical protein	WQG_10210	hypothetical protein
4	**FS42_11850**	Hemerythrin HHE cation binding domain subfamily	FS43_13350	Hemerythrin HHE cation binding domain subfamily	FS44_10610	Hemerythrin HHE cation binding domain subfamily	WQG_10200	Hemerythrin HHE cation binding domain subfamily
4	**FS42_11860**	Queuine tRNA-ribosyltransferase	FS43_13360	Queuine tRNA-ribosyltransferase	FS44_10600	Queuine tRNA-ribosyltransferase	WQG_10190	Queuine tRNA-ribosyltransferase
4	**FS42_11870**	Bifunctional protein FolD	FS43_13370	Bifunctional protein FolD	FS44_10580	Bifunctional protein FolD	WQG_10180	Bifunctional protein FolD
4	**FS42_11880**	Dihydrodipicolinate synthase	FS43_13380	Dihydrodipicolinate synthase	FS44_10570	Dihydrodipicolinate synthase	WQG_10170	Dihydrodipicolinate synthase
4	**FS42_11890**	hydrolase	FS43_13390	hydrolase	FS44_10560	hydrolase	WQG_10160	hydrolase
4	**FS42_11900**	Lipoprotein Plp4	FS43_13400	Lipoprotein Plp4	FS44_10550	Lipoprotein Plp4	WQG_10150	Lipoprotein Plp4
4	**FS42_11910**	Single-stranded-DNA-specific exonuclease recJ	FS43_13410	Single-stranded-DNA-specific exonuclease recJ	FS44_10540	Single-stranded-DNA-specific exonuclease recJ	WQG_10140	Single-stranded-DNA-specific exonuclease recJ
4	**FS42_11920**	Thiol:disulfide interchange protein	FS43_13420	Thiol:disulfide interchange protein	FS44_10530	Thiol:disulfide interchange protein	WQG_10130	Thiol:disulfide interchange protein
4	**FS42_11930**	M23B subfamily peptidase	FS43_13430	M23B subfamily peptidase	FS44_10520	M23B subfamily peptidase	WQG_10120	M23B subfamily peptidase
4	**FS42_11940**	Zinc import ATP-binding protein ZnuC	FS43_13440	Zinc import ATP-binding protein ZnuC	FS44_10510	Zinc import ATP-binding protein ZnuC	WQG_10110	Zinc import ATP-binding protein ZnuC
4	**FS42_11950**	High-affinity zinc uptake system membrane protein znuB	FS43_13450	High-affinity zinc uptake system membrane protein znuB	FS44_10500	High-affinity zinc uptake system membrane protein znuB	WQG_10100	High-affinity zinc uptake system membrane protein znuB
4	**FS42_11960**	Class II glutamine amidotransferase domain protein	FS43_13460	glutamine amidotransferase	FS44_10490	Class II glutamine amidotransferase domain protein	WQG_10090	Class II glutamine amidotransferase domain protein
4	**FS42_11970**	Lytic murein transglycosylase A	FS43_13480	Lytic murein transglycosylase A	FS44_10480	Lytic murein transglycosylase A	WQG_10080	Lytic murein transglycosylase A
4	**FS42_11980**	D-galactose-binding periplasmic protein	FS43_13490	D-galactose-binding periplasmic protein	FS44_10470	D-galactose-binding periplasmic protein	WQG_10070	D-galactose-binding periplasmic protein
4	**FS42_11990**	bifunctional P-loop ATPase/acetyltransferase	FS43_13500	bifunctional P-loop ATPase/acetyltransferase	FS44_10460	bifunctional P-loop ATPase/acetyltransferase	WQG_10060	bifunctional P-loop ATPase/acetyltransferase
4	**FS42_120**	Transcriptional regulator	FS43_130	Transcriptional regulator	FS44_23010	Transcriptional regulator	WQG_22490	Transcriptional regulator
4	**FS42_12000**	rRNA maturation factor	FS43_13520	rRNA maturation factor	FS44_10450	rRNA maturation factor	WQG_10050	rRNA maturation factor
4	**FS42_12010**	Molybdopterin-converting factor subunit 2	FS43_13530	Molybdopterin-converting factor subunit 2	FS44_10440	Molybdopterin-converting factor subunit 2	WQG_10040	Molybdopterin-converting factor subunit 2
4	**FS42_12020**	Molybdopterin synthase sulfur carrier subunit	FS43_13540	Molybdopterin synthase sulfur carrier subunit	FS44_10430	Molybdopterin synthase sulfur carrier subunit	WQG_10030	Molybdopterin synthase sulfur carrier subunit
4	**FS42_12030**	DNA topoisomerase 4 subunit A	FS43_13550	DNA topoisomerase 4 subunit A	FS44_10420	DNA topoisomerase 4 subunit A	WQG_10020	DNA topoisomerase 4 subunit A
4	**FS42_12040**	Acetyl-CoA carboxylase, carboxyl transferase, beta subunit	FS43_13560	Acetyl-CoA carboxylase, carboxyl transferase, beta subunit	FS44_10410	Acetyl-CoA carboxylase, carboxyl transferase, beta subunit	WQG_10010	Acetyl-CoA carboxylase, carboxyl transferase, beta subunit
4	**FS42_12050**	Folylpolyglutamate synthase	FS43_13570	Folylpolyglutamate synthase	FS44_10400	Folylpolyglutamate synthase	WQG_10000	Folylpolyglutamate synthase
4	**FS42_12060**	Protein trpH	FS43_13580	Protein trpH	FS44_10390	Protein trpH	WQG_9990	Protein trpH
4	**FS42_12070**	hypothetical protein	FS43_13590	hypothetical protein	FS44_10380	hypothetical protein	WQG_9980	hypothetical protein
4	**FS42_12080**	hypothetical protein	FS43_13600	hypothetical protein	FS44_10370	hypothetical protein	WQG_9970	hypothetical protein
4	**FS42_12090**	Exodeoxyribonuclease I	FS43_13610	Exodeoxyribonuclease I	FS44_10360	Exodeoxyribonuclease I	WQG_9960	Exodeoxyribonuclease I
4	**FS42_12100**	Outer-membrane lipoprotein lolB	FS43_13630	Outer-membrane lipoprotein lolB	FS44_10350	Outer-membrane lipoprotein lolB	WQG_9950	Outer-membrane lipoprotein lolB
4	**FS42_12110**	4-diphosphocytidyl-2-C-methyl-D-erythritol kinase	FS43_13640	4-diphosphocytidyl-2-C-methyl-D-erythritol kinase	FS44_10340	4-diphosphocytidyl-2-C-methyl-D-erythritol kinase	WQG_9940	4-diphosphocytidyl-2-C-methyl-D-erythritol kinase
4	**FS42_12120**	Ribose-phosphate pyrophosphokinase	FS43_13650	Ribose-phosphate pyrophosphokinase	FS44_10330	Ribose-phosphate pyrophosphokinase	WQG_9930	Ribose-phosphate pyrophosphokinase
4	**FS42_12130**	Dihydroorotate dehydrogenase	FS43_13660	Dihydroorotate dehydrogenase	FS44_10320	Dihydroorotate dehydrogenase	WQG_9920	Dihydroorotate dehydrogenase
4	**FS42_12170**	Chromosome partition protein MukB	FS43_13700	Chromosome partition protein MukB	FS44_10310	Chromosome partition protein MukB	WQG_9880	Chromosome partition protein MukB
4	**FS42_12180**	Chromosome partition protein mukE	FS43_13720	Chromosome partition protein mukE	FS44_10300	Chromosome partition protein mukE	WQG_9870	Chromosome partition protein mukE
4	**FS42_12190**	Chromosome partition protein mukF	FS43_13730	Chromosome partition protein mukF	FS44_10290	Chromosome partition protein mukF	WQG_9860	Chromosome partition protein mukF
4	**FS42_1220**	N-6 DNA methylase	FS43_1260	N-6 DNA methylase	FS44_21920	Type I restriction-modification system DNA methylase	WQG_21370	N-6 DNA methylase
4	**FS42_12200**	Ribosomal protein S6 modification protein, glutamic acid ligase	FS43_13740	Ribosomal protein S6 modification protein, glutamic acid ligase	FS44_10280	Ribosomal protein S6 modification protein, glutamic acid ligase	WQG_9850	Ribosomal protein S6 modification protein, glutamic acid ligase
4	**FS42_12210**	Monofunctional biosynthetic peptidoglycan transglycosylase	FS43_13750	Monofunctional biosynthetic peptidoglycan transglycosylase	FS44_10270	Monofunctional biosynthetic peptidoglycan transglycosylase	WQG_9840	Monofunctional biosynthetic peptidoglycan transglycosylase
4	**FS42_12220**	Trp operon repressor	FS43_13760	Trp operon repressor	FS44_10260	Trp operon repressor	WQG_9830	Trp operon repressor
4	**FS42_12230**	Outer-membrane lipoprotein carrier protein	FS43_13770	Outer-membrane lipoprotein carrier protein	FS44_10250	Outer-membrane lipoprotein carrier protein	WQG_9820	Outer-membrane lipoprotein carrier protein
4	**FS42_12240**	hypothetical protein	FS43_13780	hypothetical protein	FS44_10240	hypothetical protein	WQG_9810	hypothetical protein
4	**FS42_12250**	hypothetical protein	FS43_13790	hypothetical protein	FS44_10230	hypothetical protein	WQG_9800	hypothetical protein
4	**FS42_12260**	Cold shock-like protein CspD	FS43_13800	Cold shock-like protein CspD	FS44_10220	Cold shock-like protein CspD	WQG_9790	Cold shock-like protein CspD
4	**FS42_12270**	Carbonic anhydrase	FS43_13810	Carbonic anhydrase	FS44_10210	Carbonic anhydrase	WQG_9780	Carbonic anhydrase
4	**FS42_12280**	hypothetical protein	FS43_13820	hypothetical protein	FS44_10200	hypothetical protein	WQG_9770	hypothetical protein
4	**FS42_12290**	alkaline phosphatase superfamily hydrolase	FS43_13830	alkaline phosphatase superfamily hydrolase	FS44_10190	alkaline phosphatase superfamily hydrolase	WQG_9760	alkaline phosphatase superfamily hydrolase
4	**FS42_1230**	AraC family transcriptional regulator	FS43_1270	AraC family transcriptional regulator	FS44_21910	AraC family transcriptional regulator	WQG_21360	AraC family transcriptional regulator
4	**FS42_12300**	lipoprotein	FS43_13840	lipoprotein	FS44_10180	lipoprotein	WQG_9750	lipoprotein
4	**FS42_12310**	Integration host factor subunit alpha	FS43_13850	Integration host factor subunit alpha	FS44_10170	Integration host factor subunit alpha	WQG_9740	Integration host factor subunit alpha
4	**FS42_12320**	Phenylalanyl-tRNA synthetase beta chain	FS43_13860	Phenylalanyl-tRNA synthetase beta chain	FS44_10160	Phenylalanyl-tRNA synthetase beta chain	WQG_9730	Phenylalanyl-tRNA synthetase beta chain
4	**FS42_12330**	Phenylalanyl-tRNA synthetase alpha chain	FS43_13870	Phenylalanyl-tRNA synthetase alpha chain	FS44_10150	Phenylalanyl-tRNA synthetase alpha chain	WQG_9720	Phenylalanyl-tRNA synthetase alpha chain
4	**FS42_12340**	UPF0111 protein	FS43_13880	UPF0111 protein	FS44_10140	UPF0111 protein	WQG_9710	UPF0111 protein
4	**FS42_12350**	phosphate permease	FS43_13890	phosphate permease	FS44_10130	phosphate permease	WQG_9700	phosphate permease
4	**FS42_12360**	hypothetical protein	FS43_13900	hypothetical protein	FS44_10120	hypothetical protein	WQG_9690	hypothetical protein
4	**FS42_12370**	Spermidine/putrescine-binding periplasmic protein 1	FS43_13910	Spermidine/putrescine-binding periplasmic protein 1	FS44_10110	Spermidine/putrescine-binding periplasmic protein 1	WQG_9680	Spermidine/putrescine-binding periplasmic protein 1
4	**FS42_12380**	Ribonuclease G	FS43_13920	Ribonuclease G	FS44_10100	Ribonuclease G	WQG_9670	Ribonuclease G
4	**FS42_12390**	Anaerobic ribonucleoside-triphosphate reductase-activating protein	FS43_13930	Anaerobic ribonucleoside-triphosphate reductase-activating protein	FS44_10090	Anaerobic ribonucleoside-triphosphate reductase-activating protein	WQG_9660	Anaerobic ribonucleoside-triphosphate reductase-activating protein
4	**FS42_1240**	Luciferase	FS43_1280	Luciferase	FS44_21900	Luciferase	WQG_21350	Luciferase
4	**FS42_12400**	Anaerobic ribonucleoside-triphosphate reductase	FS43_13940	Anaerobic ribonucleoside-triphosphate reductase	FS44_10080	Anaerobic ribonucleoside-triphosphate reductase	WQG_9650	Anaerobic ribonucleoside-triphosphate reductase
4	**FS42_12410**	UPF0142 protein	FS43_13950	UPF0142 protein	FS44_10070	UPF0142 protein	WQG_9640	UPF0142 protein
4	**FS42_12420**	KamA	FS43_13960	KamA	FS44_10050	KamA	WQG_9630	KamA
4	**FS42_12430**	Preprotein translocase subunit SecG	FS43_13970	Preprotein translocase subunit SecG	FS44_10040	Preprotein translocase subunit SecG	WQG_9620	Preprotein translocase subunit SecG
4	**FS42_12440**	N-acetylneuraminate lyase	FS43_13990	N-acetylneuraminate lyase	FS44_10020	N-acetylneuraminate lyase	WQG_9600	N-acetylneuraminate lyase
4	**FS42_12450**	HTH-type transcriptional regulator	FS43_14000	HTH-type transcriptional regulator	FS44_10010	HTH-type transcriptional regulator	WQG_9590	HTH-type transcriptional regulator
4	**FS42_12460**	hypothetical protein	FS43_14010	hypothetical protein	FS44_10000	hypothetical protein	WQG_9580	hypothetical protein
4	**FS42_12470**	Exodeoxyribonuclease III	FS43_14020	Exodeoxyribonuclease III	FS44_9990	Exodeoxyribonuclease III	WQG_9570	Exodeoxyribonuclease III
4	**FS42_12480**	membrane protein	FS43_14030	membrane protein	FS44_9980	membrane protein	WQG_9560	membrane protein
4	**FS42_12490**	tRNA 2-thiocytidine biosynthesis protein TtcA	FS43_14040	tRNA 2-thiocytidine biosynthesis protein TtcA	FS44_9960	tRNA 2-thiocytidine biosynthesis protein TtcA	WQG_9540	tRNA 2-thiocytidine biosynthesis protein TtcA
4	**FS42_1250**	hydrolase	FS43_1290	hydrolase	FS44_21890	hydrolase	WQG_21340	hydrolase
4	**FS42_12500**	hypothetical protein	FS43_14050	hypothetical protein	FS44_9950	hypothetical protein	WQG_9530	hypothetical protein
4	**FS42_12510**	Fatty oxidation complex, alpha subunit	FS43_14060	Fatty oxidation complex, alpha subunit	FS44_9940	Fatty oxidation complex, alpha subunit	WQG_9520	Fatty oxidation complex, alpha subunit
4	**FS42_12520**	Acetyl-CoA acetyltransferase	FS43_14070	Acetyl-CoA acetyltransferase	FS44_9930	Acetyl-CoA acetyltransferase	WQG_9510	Acetyl-CoA acetyltransferase
4	**FS42_12550**	Large exoprotein involved in heme utilizationor adhesion	FS43_14120	Large exoprotein involved in heme utilizationor adhesion	FS44_9870	Large exoprotein involved in heme utilizationor adhesion	WQG_9490	Large exoprotein involved in heme utilizationor adhesion
4	**FS42_12570**	Colicin V production protein	FS43_14140	Colicin V production protein	FS44_9850	Colicin V production protein	WQG_9470	Colicin V production protein
4	**FS42_12580**	Succinate dehydrogenase/fumarate reductase,flavoprotein subunit	FS43_14150	Succinate dehydrogenase/fumarate reductase,flavoprotein subunit	FS44_9840	Succinate dehydrogenase/fumarate reductase,flavoprotein subunit	WQG_9460	Succinate dehydrogenase/fumarate reductase,flavoprotein subunit
4	**FS42_12590**	ABC transporter protein	FS43_14160	ABC transporter protein	FS44_9830	ABC transporter protein	WQG_9450	ABC transporter protein
4	**FS42_1260**	2-succinyl-5-enolpyruvyl-6-hydroxy-3- cyclohexene-1-carboxylate synthase	FS43_1300	2-succinyl-5-enolpyruvyl-6-hydroxy-3- cyclohexene-1-carboxylate synthase	FS44_21880	2-succinyl-5-enolpyruvyl-6-hydroxy-3- cyclohexene-1-carboxylate synthase	WQG_21330	2-succinyl-5-enolpyruvyl-6-hydroxy-3- cyclohexene-1-carboxylate synthase
4	**FS42_12600**	ABC transporter protein	FS43_14170	ABC transporter protein	FS44_9820	ABC transporter protein	WQG_9440	ABC transporter protein
4	**FS42_12610**	Binding-protein-dependent transport system inner membrane component	FS43_14180	Binding-protein-dependent transport system inner membrane component	FS44_9810	Binding-protein-dependent transport system inner membrane component	WQG_9430	Binding-protein-dependent transport system inner membrane component
4	**FS42_12620**	Extracellular solute-binding protein family 5	FS43_14190	Extracellular solute-binding protein family 5	FS44_9800	Extracellular solute-binding protein family 5	WQG_9420	Extracellular solute-binding protein family 5
4	**FS42_12630**	Ribonuclease HI	FS43_14200	Ribonuclease HI	FS44_9790	Ribonuclease HI	WQG_9410	Ribonuclease HI
4	**FS42_12640**	DNA-binding protein H-NS	FS43_14210	DNA-binding protein H-NS	FS44_9780	DNA-binding protein H-NS	WQG_9400	DNA-binding protein H-NS
4	**FS42_12650**	hypothetical protein	FS43_14220	hypothetical protein	FS44_9770	hypothetical protein	WQG_9390	hypothetical protein
4	**FS42_12660**	hypothetical protein	FS43_14230	hypothetical protein	FS44_9760	hypothetical protein	WQG_9380	hypothetical protein
4	**FS42_12670**	DNA repair protein RadA	FS43_14240	DNA repair protein RadA	FS44_9750	DNA repair protein RadA	WQG_9370	DNA repair protein RadA
4	**FS42_12680**	prepilin peptidase dependent protein D	FS43_14250	prepilin peptidase dependent protein D	FS44_9740	prepilin peptidase dependent protein D	WQG_9360	prepilin peptidase dependent protein D
4	**FS42_12690**	transport protein	FS43_14260	transport protein	FS44_9730	transport protein	WQG_9350	transport protein
4	**FS42_1270**	Glutamate-1-semialdehyde 2,1-aminomutase	FS43_1310	Glutamate-1-semialdehyde 2,1-aminomutase	FS44_21870	Glutamate-1-semialdehyde 2,1-aminomutase	WQG_21320	Glutamate-1-semialdehyde 2,1-aminomutase
4	**FS42_12700**	Tfp pilus assembly pathway, component PilC	FS43_14280	Tfp pilus assembly pathway, component PilC	FS44_9720	Tfp pilus assembly pathway, component PilC	WQG_9340	Tfp pilus assembly pathway, component PilC
4	**FS42_12710**	Tfp pilus assembly pathway, fimbrial leader peptidase PilD	FS43_14290	Tfp pilus assembly pathway, fimbrial leader peptidase PilD	FS44_9710	Tfp pilus assembly pathway, fimbrial leader peptidase PilD	WQG_9330	Tfp pilus assembly pathway, fimbrial leader peptidase PilD
4	**FS42_12720**	Dephospho-CoA kinase	FS43_14300	Dephospho-CoA kinase	FS44_9700	Dephospho-CoA kinase	WQG_9320	Dephospho-CoA kinase
4	**FS42_12730**	DNA gyrase inhibitor YacG	FS43_14310	DNA gyrase inhibitor YacG	FS44_9690	DNA gyrase inhibitor YacG	WQG_9310	DNA gyrase inhibitor YacG
4	**FS42_12740**	Lipoprotein HlpB	FS43_14320	Lipoprotein HlpB	FS44_9680	Lipoprotein HlpB	WQG_9300	Lipoprotein HlpB
4	**FS42_12750**	FAD-dependent monooxygenase	FS43_14330	FAD-dependent monooxygenase	FS44_9670	FAD-dependent monooxygenase	WQG_9290	FAD-dependent monooxygenase
4	**FS42_12760**	hypothetical protein	FS43_14340	hypothetical protein	FS44_9660	hypothetical protein	WQG_9280	hypothetical protein
4	**FS42_12770**	PTS system, nitrogen regulatory IIA-like protein	FS43_14350	PTS system, nitrogen regulatory IIA-like protein	FS44_9650	PTS system, nitrogen regulatory IIA-like protein	WQG_9270	PTS system, nitrogen regulatory IIA-like protein
4	**FS42_12780**	Lipopolysaccharide export system ATP-binding protein LptB	FS43_14360	Lipopolysaccharide export system ATP-binding protein LptB	FS44_9640	Lipopolysaccharide export system ATP-binding protein LptB	WQG_9260	Lipopolysaccharide export system ATP-binding protein LptB
4	**FS42_12790**	Cell envelope bioproteinis YhbN	FS43_14370	Cell envelope bioproteinis YhbN	FS44_9630	Cell envelope bioproteinis YhbN	WQG_9250	Cell envelope bioproteinis YhbN

4	**FS42_1280**	Prolipoprotein diacylglyceryl transferase	FS43_1320	Prolipoprotein diacylglyceryl transferase	FS44_21860	Prolipoprotein diacylglyceryl transferase	WQG_21310	Prolipoprotein diacylglyceryl transferase
4	**FS42_12800**	hypothetical protein	FS43_14380	hypothetical protein	FS44_9620	hypothetical protein	WQG_9240	hypothetical protein
4	**FS42_12810**	SanA protein, membrane protein	FS43_14390	SanA protein, membrane protein	FS44_9610	SanA protein, membrane protein	WQG_9230	SanA protein, membrane protein
4	**FS42_12820**	Homoserine O-acetyltransferase	FS43_14400	Homoserine O-acetyltransferase	FS44_9600	Homoserine O-acetyltransferase	WQG_9220	Homoserine O-acetyltransferase
4	**FS42_12830**	DNA repair protein recO	FS43_14410	DNA repair protein recO	FS44_9590	DNA repair protein recO	WQG_9210	DNA repair protein recO
4	**FS42_12840**	GTP-binding protein Era	FS43_14420	GTP-binding protein Era	FS44_9580	GTP-binding protein Era	WQG_9200	GTP-binding protein Era
4	**FS42_12850**	Ribonuclease 3	FS43_14440	Ribonuclease 3	FS44_9550	Ribonuclease 3	WQG_9190	Ribonuclease 3
4	**FS42_12860**	Signal peptidase I	FS43_14450	Signal peptidase I	FS44_9540	Signal peptidase I	WQG_9180	Signal peptidase I
4	**FS42_12870**	Elongation factor 4	FS43_14470	Elongation factor 4	FS44_9530	Elongation factor 4	WQG_9170	Elongation factor 4
4	**FS42_12880**	Na+/alanine symporter	FS43_14480	Na+/alanine symporter	FS44_9520	Na+/alanine symporter	WQG_9160	Na+/alanine symporter
4	**FS42_12890**	ABC-type nickel/cobalt efflux system, permease component	FS43_14490	ABC-type nickel/cobalt efflux system, permease component	FS44_9510	ABC-type nickel/cobalt efflux system, permease component	WQG_9150	ABC-type nickel/cobalt efflux system, permease component
4	**FS42_1290**	Permease	FS43_1330	Permease	FS44_21850	Permease	WQG_21300	Permease
4	**FS42_12900**	ABC-type nickel/cobalt efflux system	FS43_14500	ABC-type nickel/cobalt efflux system	FS44_9500	ABC-type nickel/cobalt efflux system	WQG_9140	ABC-type nickel/cobalt efflux system
4	**FS42_12910**	2,3-bisphosphoglycerate-dependent phosphoglycerate mutase	FS43_14510	2,3-bisphosphoglycerate-dependent phosphoglycerate mutase	FS44_9490	2,3-bisphosphoglycerate-dependent phosphoglycerate mutase	WQG_9130	2,3-bisphosphoglycerate-dependent phosphoglycerate mutase
4	**FS42_12920**	Molybdenum import ATP-binding protein ModC	FS43_14520	Molybdenum import ATP-binding protein ModC	FS44_9480	Molybdenum import ATP-binding protein ModC	WQG_9120	Molybdenum import ATP-binding protein ModC
4	**FS42_12930**	Molybdenum transport system permease protein modB	FS43_14540	Molybdenum transport system permease protein modB	FS44_9470	Molybdenum transport system permease protein modB	WQG_9110	Molybdenum transport system permease protein modB
4	**FS42_12940**	Molybdate-binding periplasmic protein	FS43_14550	Molybdate-binding periplasmic protein	FS44_9460	Molybdate-binding periplasmic protein	WQG_9100	Molybdate-binding periplasmic protein
4	**FS42_12950**	DNA-binding protein	FS43_14560	DNA-binding protein	FS44_9450	DNA-binding protein	WQG_9090	DNA-binding protein
4	**FS42_12960**	hypothetical protein	FS43_14570	hypothetical protein	FS44_9440	hypothetical protein	WQG_9080	hypothetical protein
4	**FS42_12970**	Transcriptional regulator modE	FS43_14580	Transcriptional regulator modE	FS44_9400	Transcriptional regulator modE	WQG_9070	Transcriptional regulator modE
4	**FS42_12980**	Tryptophan synthase alpha chain	FS43_14590	Tryptophan synthase alpha chain	FS44_9390	Tryptophan synthase alpha chain	WQG_9060	Tryptophan synthase alpha chain
4	**FS42_12990**	Tryptophan synthase beta chain	FS43_14600	Tryptophan synthase beta chain	FS44_9380	Tryptophan synthase beta chain	WQG_9050	Tryptophan synthase beta chain
4	**FS42_130**	PfkB domain protein	FS43_140	PfkB domain protein	FS44_23000	PfkB domain protein	WQG_22480	PfkB domain protein
4	**FS42_1300**	RNA pyrophosphohydrolase	FS43_1340	RNA pyrophosphohydrolase	FS44_21840	RNA pyrophosphohydrolase	WQG_21290	RNA pyrophosphohydrolase
4	**FS42_13000**	Protein mrp	FS43_14610	Protein mrp	FS44_9370	Protein mrp	WQG_9040	Protein mrp
4	**FS42_13010**	oligopeptide transporter, periplasmic-binding protein, OppA	FS43_14620	oligopeptide transporter, periplasmic-binding protein, OppA	FS44_9360	oligopeptide transporter, periplasmic-binding protein, OppA	WQG_9030	oligopeptide transporter, periplasmic-binding protein, OppA
4	**FS42_13020**	hypothetical protein	FS43_14630	hypothetical protein	FS44_9350	hypothetical protein	WQG_9020	hypothetical protein
4	**FS42_13030**	Ribosomal small subunit pseudouridine synthase A	FS43_14640	Ribosomal small subunit pseudouridine synthase A	FS44_9340	Ribosomal small subunit pseudouridine synthase A	WQG_9010	Ribosomal small subunit pseudouridine synthase A
4	**FS42_13040**	Drug resistance transporter, Bcr/CflA subfamily	FS43_14650	Drug resistance transporter, Bcr/CflA subfamily	FS44_9330	Drug resistance transporter, Bcr/CflA subfamily	WQG_9000	Drug resistance transporter, Bcr/CflA subfamily
4	**FS42_13050**	hypothetical protein	FS43_14660	hypothetical protein	FS44_9320	hypothetical protein	WQG_8990	hypothetical protein
4	**FS42_13060**	3-isopropylmalate dehydratase large subunit-like protein	FS43_14670	3-isopropylmalate dehydratase large subunit-like protein	FS44_9310	3-isopropylmalate dehydratase large subunit-like protein	WQG_8980	3-isopropylmalate dehydratase large subunit-like protein
4	**FS42_13070**	3-isopropylmalate dehydratase large subunit-like protein	FS43_14680	3-isopropylmalate dehydratase large subunit-like protein	FS44_9300	3-isopropylmalate dehydratase large subunit-like protein	WQG_8970	3-isopropylmalate dehydratase large subunit-like protein
4	**FS42_13080**	NADP-dependent dehydrogenase	FS43_14690	NADP-dependent dehydrogenase	FS44_9290	NADP-dependent dehydrogenase	WQG_8960	NADP-dependent dehydrogenase
4	**FS42_13090**	hypothetical protein	FS43_14700	hypothetical protein	FS44_9280	hypothetical protein	WQG_8950	hypothetical protein
4	**FS42_1310**	thiol peroxidase	FS43_1350	thiol peroxidase	FS44_21820	thiol peroxidase	WQG_21280	thiol peroxidase
4	**FS42_13100**	hypothetical protein	FS43_14710	hypothetical protein	FS44_9270	hypothetical protein	WQG_8940	hypothetical protein
4	**FS42_13110**	Non-canonical purine NTP pyrophosphatase	FS43_14720	Non-canonical purine NTP pyrophosphatase	FS44_9260	Non-canonical purine NTP pyrophosphatase	WQG_8930	Non-canonical purine NTP pyrophosphatase
4	**FS42_13120**	Lipid A acyltransferase	FS43_14730	Lipid A acyltransferase	FS44_9250	Lipid A acyltransferase	WQG_8920	Lipid A acyltransferase
4	**FS42_13130**	Sigma 54 modulation protein/ribosomal protein S30EA	FS43_14740	Sigma 54 modulation protein/ribosomal protein S30EA	FS44_9240	Sigma 54 modulation protein/ribosomal protein S30EA	WQG_8910	Sigma 54 modulation protein/ribosomal protein S30EA
4	**FS42_13140**	Glutaminase	FS43_14750	Glutaminase	FS44_9230	Glutaminase	WQG_8900	Glutaminase
4	**FS42_13150**	Aspartyl-tRNA synthetase	FS43_14760	Aspartyl-tRNA synthetase	FS44_9220	Aspartyl-tRNA synthetase	WQG_8890	Aspartyl-tRNA synthetase
4	**FS42_13160**	Protein grpE	FS43_14780	Protein grpE	FS44_9210	Protein grpE	WQG_8880	Protein grpE
4	**FS42_13170**	hypothetical protein	FS43_14790	hypothetical protein	FS44_9200	hypothetical protein	WQG_8870	hypothetical protein
4	**FS42_13180**	hypothetical protein	FS43_14800	hypothetical protein	FS44_9190	hypothetical protein	WQG_8860	hypothetical protein
4	**FS42_13190**	ATP-dependent RNA helicase	FS43_14820	ATP-dependent RNA helicase	FS44_9180	ATP-dependent RNA helicase	WQG_8840	ATP-dependent RNA helicase
4	**FS42_1320**	1-acyl-sn-glycerol-3-phosphate acyltransferase	FS43_1360	1-acyl-sn-glycerol-3-phosphate acyltransferase	FS44_21810	1-acyl-sn-glycerol-3-phosphate acyltransferase	WQG_21270	1-acyl-sn-glycerol-3-phosphate acyltransferase
4	**FS42_13200**	Nodulation efficiency protein D	FS43_14830	Nodulation efficiency protein D	FS44_9170	Nodulation efficiency protein D	WQG_8830	Nodulation efficiency protein D
4	**FS42_13210**	Protein QmcA	FS43_14840	Protein QmcA	FS44_9160	Protein QmcA	WQG_8820	Protein QmcA
4	**FS42_13220**	Alanine racemase	FS43_14850	Alanine racemase	FS44_9150	Alanine racemase	WQG_8810	Alanine racemase
4	**FS42_13230**	hypothetical protein	FS43_14860	hypothetical protein	FS44_9000	hypothetical protein	WQG_8800	hypothetical protein
4	**FS42_13240**	hypothetical protein	FS43_14870	hypothetical protein	FS44_9010	hypothetical protein	WQG_8790	hypothetical protein
4	**FS42_13250**	tRNA pseudouridine synthase C	FS43_14880	tRNA pseudouridine synthase C	FS44_9020	tRNA pseudouridine synthase C	WQG_8780	tRNA pseudouridine synthase C
4	**FS42_13260**	hypothetical protein	FS43_14890	hypothetical protein	FS44_9030	hypothetical protein	WQG_8770	hypothetical protein
4	**FS42_13270**	tRNA pseudouridine synthase C	FS43_14900	tRNA pseudouridine synthase C	FS44_9040	tRNA pseudouridine synthase C	WQG_8760	tRNA pseudouridine synthase C
4	**FS42_13280**	Anhydro-N-acetylmuramic acid kinase	FS43_14910	Anhydro-N-acetylmuramic acid kinase	FS44_9050	Anhydro-N-acetylmuramic acid kinase	WQG_8750	Anhydro-N-acetylmuramic acid kinase
4	**FS42_13290**	Tryptophan biosynthesis protein TrpCF	FS43_14920	Tryptophan biosynthesis protein TrpCF	FS44_9060	Tryptophan biosynthesis protein TrpCF	WQG_8740	Tryptophan biosynthesis protein TrpCF
4	**FS42_1330**	Protein SufI/multicopper oxidase type 3	FS43_1370	Protein SufI/multicopper oxidase type 3	FS44_21800	Protein SufI/multicopper oxidase type 3	WQG_21260	Protein SufI/multicopper oxidase type 3
4	**FS42_13300**	Sulfur carrier protein moaD adenylyltransferase	FS43_14930	Sulfur carrier protein moaD adenylyltransferase	FS44_9070	Sulfur carrier protein moaD adenylyltransferase	WQG_8730	Sulfur carrier protein moaD adenylyltransferase
4	**FS42_13310**	Molybdopterin molybdenumtransferase	FS43_14940	Molybdopterin molybdenumtransferase	FS44_9080	Molybdopterin molybdenumtransferase	WQG_8720	Molybdopterin molybdenumtransferase
4	**FS42_13320**	Multiple antibiotic resistance (MarC)-related protein	FS43_14950	Multiple antibiotic resistance (MarC)-related protein	FS44_9090	Multiple antibiotic resistance (MarC)-related protein	WQG_8710	Multiple antibiotic resistance (MarC)-related protein
4	**FS42_13330**	ABC transporter ATP-binding protein	FS43_14960	ABC transporter ATP-binding protein	FS44_9100	ABC transporter ATP-binding protein	WQG_8700	ABC transporter ATP-binding protein
4	**FS42_13340**	GntP family transporter	FS43_14970	GntP family transporter	FS44_9110	GntP family transporter	WQG_8690	GntP family transporter
4	**FS42_13350**	D-beta-hydroxybutyrate dehydrogenase	FS43_14980	D-beta-hydroxybutyrate dehydrogenase	FS44_8960	D-beta-hydroxybutyrate dehydrogenase	WQG_8680	D-beta-hydroxybutyrate dehydrogenase
4	**FS42_13360**	Acetyl-CoA acetyltransferase	FS43_14990	Acetyl-CoA acetyltransferase	FS44_8950	Acetyl-CoA acetyltransferase	WQG_8670	Acetyl-CoA acetyltransferase
4	**FS42_13370**	Short-chain fatty acids transporter	FS43_15000	Short-chain fatty acids transporter	FS44_8940	Short-chain fatty acids transporter	WQG_8660	Short-chain fatty acids transporter
4	**FS42_13380**	Acetate CoA-transferase subunit beta	FS43_15010	Acetate CoA-transferase subunit beta	FS44_8930	Acetate CoA-transferase subunit beta	WQG_8650	Acetate CoA-transferase subunit beta
4	**FS42_13390**	Acetate CoA-transferase subunit alpha	FS43_15020	Acetate CoA-transferase subunit alpha	FS44_8920	Acetate CoA-transferase subunit alpha	WQG_8640	Acetate CoA-transferase subunit alpha
4	**FS42_1340**	Acetyl-coenzyme A carboxylase carboxyl transferase subunit alpha	FS43_1380	Acetyl-coenzyme A carboxylase carboxyl transferase subunit alpha	FS44_21790	Acetyl-coenzyme A carboxylase carboxyl transferase subunit alpha	WQG_21250	Acetyl-coenzyme A carboxylase carboxyl transferase subunit alpha
4	**FS42_13400**	HTH-type transcriptional regulator	FS43_15030	HTH-type transcriptional regulator	FS44_8910	HTH-type transcriptional regulator	WQG_8630	HTH-type transcriptional regulator
4	**FS42_13410**	O-succinylbenzoate synthase	FS43_15040	O-succinylbenzoate synthase	FS44_8900	O-succinylbenzoate synthase	WQG_8620	O-succinylbenzoate synthase
4	**FS42_13420**	Beta-propeller domain of methanol dehydrogenase type	FS43_15050	Beta-propeller domain of methanol dehydrogenase type	FS44_8890	Beta-propeller domain of methanol dehydrogenase type	WQG_8610	Beta-propeller domain of methanol dehydrogenase type
4	**FS42_13430**	membrane protein	FS43_15060	membrane protein	FS44_8880	membrane protein	WQG_8600	membrane protein
4	**FS42_13440**	LemA protein	FS43_15070	LemA protein	FS44_8870	LemA protein	WQG_8590	LemA protein
4	**FS42_13450**	hypothetical protein	FS43_15080	hypothetical protein	FS44_8860	hypothetical protein	WQG_8580	hypothetical protein
4	**FS42_13460**	DNA polymerase beta subunit	FS43_15090	DNA polymerase beta subunit	FS44_8850	DNA polymerase beta subunit	WQG_8570	DNA polymerase beta subunit
4	**FS42_13480**	tRNA-dihydrouridine synthase C	FS43_15110	tRNA-dihydrouridine synthase C	FS44_8820	tRNA-dihydrouridine synthase C	WQG_8550	tRNA-dihydrouridine synthase C
4	**FS42_13490**	DnaJ-like protein DjlA	FS43_15120	DnaJ-like protein DjlA	FS44_8810	DnaJ-like protein DjlA	WQG_8540	DnaJ-like protein DjlA
4	**FS42_1350**	hypothetical protein	FS43_1390	hypothetical protein	FS44_21780	hypothetical protein	WQG_21240	hypothetical protein
4	**FS42_13500**	Outer membrane protein P6	FS43_15130	Outer membrane protein P6	FS44_8800	Outer membrane protein P6	WQG_8530	Outer membrane protein P6
4	**FS42_13510**	Protein tolB	FS43_15140	Protein tolB	FS44_8790	Protein tolB	WQG_8520	Protein tolB
4	**FS42_13520**	Protein tolA	FS43_15150	Protein tolA	FS44_8780	Protein tolA	WQG_8510	Protein tolA
4	**FS42_13530**	Protein tolR	FS43_15160	Protein tolR	FS44_8770	Protein tolR	WQG_8500	Protein tolR
4	**FS42_13540**	Protein tolQ	FS43_15170	Protein tolQ	FS44_8760	Protein tolQ	WQG_8490	Protein tolQ
4	**FS42_13550**	thioesterase	FS43_15180	thioesterase	FS44_8750	thioesterase	WQG_8480	thioesterase
4	**FS42_13560**	Cyd operon protein YbgE	FS43_15190	Cyd operon protein YbgE	FS44_8740	Cyd operon protein YbgE	WQG_8470	Cyd operon protein YbgE
4	**FS42_13570**	Cytochrome d ubiquinol oxidase subunit 2	FS43_15200	Cytochrome d ubiquinol oxidase subunit 2	FS44_8730	Cytochrome d ubiquinol oxidase subunit 2	WQG_8460	Cytochrome d ubiquinol oxidase subunit 2
4	**FS42_13580**	Cytochrome d ubiquinol oxidase subunit 1	FS43_15210	Cytochrome d ubiquinol oxidase subunit 1	FS44_8720	Cytochrome d ubiquinol oxidase subunit 1	WQG_8450	Cytochrome d ubiquinol oxidase subunit 1
4	**FS42_13590**	HemY-like protein	FS43_15220	HemY-like protein	FS44_8710	HemY-like protein	WQG_8440	HemY-like protein
4	**FS42_1360**	Pyridoxamine kinase	FS43_1400	Pyridoxamine kinase	FS44_21770	Pyridoxamine kinase	WQG_21230	Pyridoxamine kinase
4	**FS42_13600**	Uroporphyrin-III C-methyltransferase	FS43_15230	Uroporphyrin-III C-methyltransferase	FS44_8700	Uroporphyrin-III C-methyltransferase	WQG_8430	Uroporphyrin-III C-methyltransferase
4	**FS42_13610**	Uroporphyrinogen-III synthase	FS43_15240	Uroporphyrinogen-III synthase	FS44_8690	Uroporphyrinogen-III synthase	WQG_8420	Uroporphyrinogen-III synthase
4	**FS42_13620**	Porphobilinogen deaminase	FS43_15250	Porphobilinogen deaminase	FS44_8680	Porphobilinogen deaminase	WQG_8410	Porphobilinogen deaminase
4	**FS42_13630**	hypothetical protein	FS43_15260	hypothetical protein	FS44_8670	hypothetical protein	WQG_8400	hypothetical protein
4	**FS42_13640**	transporter	FS43_15270	transporter	FS44_8660	transporter	WQG_8390	transporter
4	**FS42_13650**	23S rRNA (uracil-5-)-methyltransferase RumB	FS43_15280	23S rRNA (uracil-5-)-methyltransferase RumB	FS44_8640	23S rRNA (uracil-5-)-methyltransferase RumB	WQG_8380	23S rRNA (uracil-5-)-methyltransferase RumB
4	**FS42_13660**	Beta-hexosaminidase	FS43_15290	Beta-hexosaminidase	FS44_8630	Beta-hexosaminidase	WQG_8370	Beta-hexosaminidase
4	**FS42_13670**	DNA mismatch repair protein mutH	FS43_15300	DNA mismatch repair protein mutH	FS44_8620	DNA mismatch repair protein mutH	WQG_8360	DNA mismatch repair protein mutH
4	**FS42_13680**	DNA repair protein recN	FS43_15310	DNA repair protein recN	FS44_8610	DNA repair protein recN	WQG_8350	DNA repair protein recN
4	**FS42_13690**	hypothetical protein	FS43_15320	hypothetical protein	FS44_8600	hypothetical protein	WQG_8340	hypothetical protein
4	**FS42_1370**	Penicillin-binding protein 1B	FS43_1410	Penicillin-binding protein 1B	FS44_21760	Penicillin-binding protein 1B	WQG_21220	Penicillin-binding protein 1B
4	**FS42_13700**	Transporter, Drug/Metabolite Exporter	FS43_15330	Transporter, Drug/Metabolite Exporter	FS44_8590	Transporter, Drug/Metabolite Exporter	WQG_8330	Transporter, Drug/Metabolite Exporter
4	**FS42_13710**	inorganic polyphosphate/ATP-NAD kinase	FS43_15340	inorganic polyphosphate/ATP-NAD kinase	FS44_8580	inorganic polyphosphate/ATP-NAD kinase	WQG_8320	inorganic polyphosphate/ATP-NAD kinase
4	**FS42_13720**	10 kDa chaperonin	FS43_15350	10 kDa chaperonin	FS44_8570	10 kDa chaperonin	WQG_8310	10 kDa chaperonin
4	**FS42_13740**	60 kDa chaperonin	FS43_15360	60 kDa chaperonin	FS44_8560	60 kDa chaperonin	WQG_8300	60 kDa chaperonin
4	**FS42_13750**	PurR protein	FS43_15370	PurR protein	FS44_8550	Trehalose repressor	WQG_8290	PurR protein
4	**FS42_13760**	Pyruvate formate lyase-activating enzyme 1	FS43_15380	Pyruvate formate lyase-activating enzyme 1	FS44_8540	Pyruvate formate lyase-activating enzyme 1	WQG_8280	Pyruvate formate lyase-activating enzyme 1
4	**FS42_13770**	hypothetical protein	FS43_15390	hypothetical protein	FS44_8530	hypothetical protein	WQG_8270	hypothetical protein
4	**FS42_13780**	50S ribosomal protein L9	FS43_15400	50S ribosomal protein L9	FS44_8520	50S ribosomal protein L9	WQG_8260	50S ribosomal protein L9
4	**FS42_13790**	30S ribosomal protein S18	FS43_15410	30S ribosomal protein S18	FS44_8510	30S ribosomal protein S18	WQG_8250	30S ribosomal protein S18
4	**FS42_1380**	long-chain-fatty-acid--CoA ligase	FS43_1420	long-chain-fatty-acid--CoA ligase	FS44_21750	long-chain-fatty-acid--CoA ligase	WQG_21210	long-chain-fatty-acid--CoA ligase
4	**FS42_13800**	Primosomal replication protein n	FS43_15420	Primosomal replication protein n	FS44_8500	Primosomal replication protein n	WQG_8240	Primosomal replication protein n
4	**FS42_13810**	30S ribosomal protein S6	FS43_15430	30S ribosomal protein S6	FS44_8490	30S ribosomal protein S6	WQG_8230	30S ribosomal protein S6
4	**FS42_13820**	hypothetical protein	FS43_15440	hypothetical protein	FS44_8480	hypothetical protein	WQG_8220	hypothetical protein
4	**FS42_13830**	hypothetical protein	FS43_15450	hypothetical protein	FS44_8470	hypothetical protein	WQG_8210	hypothetical protein
4	**FS42_13840**	hypothetical protein	FS43_15460	hypothetical protein	FS44_8460	hypothetical protein	WQG_8200	hypothetical protein
4	**FS42_13850**	hypothetical protein	FS43_15470	hypothetical protein	FS44_8450	hypothetical protein	WQG_8190	hypothetical protein
4	**FS42_1390**	L-cystine transport system permease protein tcyB	FS43_1430	L-cystine transport system permease protein tcyB	FS44_21740	L-cystine transport system permease protein tcyB	WQG_21200	L-cystine transport system permease protein tcyB
4	**FS42_13940**	Protein ychN	FS43_15560	Protein ychN	FS44_8440	Protein ychN	WQG_8110	Protein ychN
4	**FS42_13950**	hypothetical protein	FS43_15570	hypothetical protein	FS44_8430	hypothetical protein	WQG_8100	hypothetical protein
4	**FS42_13960**	cysteine desulfurase	FS43_15580	cysteine desulfurase	FS44_8420	cysteine desulfurase	WQG_8090	cysteine desulfurase
4	**FS42_13970**	GDP-L-fucose synthase	FS43_15590	GDP-L-fucose synthase	FS44_8410	GDP-L-fucose synthase	WQG_8080	GDP-L-fucose synthase
4	**FS42_13980**	6-phosphofructokinase	FS43_15600	6-phosphofructokinase	FS44_8400	6-phosphofructokinase	WQG_8070	6-phosphofructokinase
4	**FS42_13990**	tRNA sulfurtransferase	FS43_15610	tRNA sulfurtransferase	FS44_8390	tRNA sulfurtransferase	WQG_8060	tRNA sulfurtransferase
4	**FS42_140**	IolD	FS43_150	IolD	FS44_22990	IolD	WQG_22470	IolD
4	**FS42_1400**	amino-acid ABC transporter ATP-binding protein	FS43_1440	amino-acid ABC transporter ATP-binding protein	FS44_21730	amino-acid ABC transporter ATP-binding protein	WQG_21190	amino-acid ABC transporter ATP-binding protein
4	**FS42_14000**	RND efflux system outer membrane lipoprotein	FS43_15620	RND efflux system outer membrane lipoprotein	FS44_8380	RND efflux system outer membrane lipoprotein	WQG_8050	RND efflux system outer membrane lipoprotein
4	**FS42_14010**	Guanylate kinase	FS43_15630	Guanylate kinase	FS44_8370	Guanylate kinase	WQG_8040	Guanylate kinase
4	**FS42_14020**	Xanthine phosphoribosyltransferase	FS43_15640	Xanthine phosphoribosyltransferase	FS44_8360	Xanthine phosphoribosyltransferase	WQG_8030	Xanthine phosphoribosyltransferase
4	**FS42_14030**	Aminoacyl-histidine dipeptidase	FS43_15650	Aminoacyl-histidine dipeptidase	FS44_8350	Aminoacyl-histidine dipeptidase	WQG_8020	Aminoacyl-histidine dipeptidase
4	**FS42_14040**	hypothetical protein	FS43_15660	hypothetical protein	FS44_17750	hypothetical protein	WQG_8010	hypothetical protein
4	**FS42_14060**	hypothetical protein	FS43_15680	hypothetical protein	FS44_8340	hypothetical protein	WQG_7990	hypothetical protein
4	**FS42_14070**	hypothetical protein	FS43_15690	hypothetical protein	FS44_8330	hypothetical protein	WQG_7980	hypothetical protein
4	**FS42_14080**	hypothetical protein	FS43_15700	hypothetical protein	FS44_8320	hypothetical protein	WQG_7970	hypothetical protein
4	**FS42_14090**	hypothetical protein	FS43_15710	hypothetical protein	FS44_8310	hypothetical protein	WQG_7960	hypothetical protein
4	**FS42_1410**	Bifunctional riboflavin kinase/FMN adenylyltransferase	FS43_1450	Bifunctional riboflavin kinase/FMN adenylyltransferase	FS44_21720	Bifunctional riboflavin kinase/FMN adenylyltransferase	WQG_21180	Bifunctional riboflavin kinase/FMN adenylyltransferase
4	**FS42_1420**	Large-conductance mechanosensitive channel	FS43_1460	Large-conductance mechanosensitive channel	FS44_21710	Large-conductance mechanosensitive channel	WQG_21170	Large-conductance mechanosensitive channel
4	**FS42_14240**	Bifunctional GntR family transcriptional regulator/aminotransferase	FS43_15890	Bifunctional GntR family transcriptional regulator/aminotransferase	FS44_8160	Bifunctional GntR family transcriptional regulator/aminotransferase	WQG_7810	Bifunctional GntR family transcriptional regulator/aminotransferase
4	**FS42_14250**	Pyridoxal biosynthesis lyase pdxS	FS43_15900	Pyridoxal biosynthesis lyase pdxS	FS44_8150	Pyridoxal biosynthesis lyase pdxS	WQG_7800	Pyridoxal biosynthesis lyase pdxS
4	**FS42_14260**	Glutamine amidotransferase subunit pdxT	FS43_15910	Glutamine amidotransferase subunit pdxT	FS44_8140	Glutamine amidotransferase subunit pdxT	WQG_7790	Glutamine amidotransferase subunit pdxT
4	**FS42_14270**	Glucitol operon repressor	FS43_15920	Glucitol operon repressor	FS44_8130	Glucitol operon repressor	WQG_7780	Glucitol operon repressor
4	**FS42_14280**	HAD-superfamily hydrolase, subfamily IA, variant 3	FS43_15930	HAD-superfamily hydrolase, subfamily IA, variant 3	FS44_8120	HAD-superfamily hydrolase, subfamily IA, variant 3	WQG_7770	HAD-superfamily hydrolase, subfamily IA, variant 3
4	**FS42_14290**	Glucitol operon activator	FS43_15940	Glucitol operon activator	FS44_8110	Glucitol operon activator	WQG_7760	Glucitol operon activator

4	**FS42_1430**	Protoporphyrinogen oxidase	FS43_1470	Protoporphyrinogen oxidase	FS44_21700	Protoporphyrinogen oxidase	WQG_21160	Protoporphyrinogen oxidase
4	**FS42_14300**	Sorbitol-6-phosphate 2-dehydrogenase	FS43_15950	Sorbitol-6-phosphate 2-dehydrogenase	FS44_8100	Sorbitol-6-phosphate 2-dehydrogenase	WQG_7750	Sorbitol-6-phosphate 2-dehydrogenase
4	**FS42_14310**	PTS system glucitol/sorbitol-specific IIA component	FS43_15960	PTS system glucitol/sorbitol-specific IIA component	FS44_8090	PTS system glucitol/sorbitol-specific IIA component	WQG_7740	PTS system glucitol/sorbitol-specific IIA component
4	**FS42_14320**	phosphotransferase	FS43_15970	phosphotransferase	FS44_8080	phosphotransferase	WQG_7730	phosphotransferase
4	**FS42_14330**	Glucitol/sorbitol permease IIC component	FS43_15990	Glucitol/sorbitol permease IIC component	FS44_8070	Glucitol/sorbitol permease IIC component	WQG_7720	Glucitol/sorbitol permease IIC component
4	**FS42_14340**	7-cyano-7-deazaguanine synthase	FS43_16000	7-cyano-7-deazaguanine synthase	FS44_8060	7-cyano-7-deazaguanine synthase	WQG_7710	7-cyano-7-deazaguanine synthase
4	**FS42_14350**	6-pyruvoyl tetrahydrobiopterin synthase	FS43_16010	6-pyruvoyl tetrahydrobiopterin synthase	FS44_8050	6-pyruvoyl tetrahydrobiopterin synthase	WQG_7700	6-pyruvoyl tetrahydrobiopterin synthase
4	**FS42_14360**	7-carboxy-7-deazaguanine synthase	FS43_16020	7-carboxy-7-deazaguanine synthase	FS44_8040	7-carboxy-7-deazaguanine synthase	WQG_7690	7-carboxy-7-deazaguanine synthase
4	**FS42_14370**	Glutathione-disulfide reductase	FS43_16030	Glutathione-disulfide reductase	FS44_8030	Glutathione-disulfide reductase	WQG_7680	Glutathione-disulfide reductase
4	**FS42_14380**	Membrane protein	FS43_16040	Membrane protein	FS44_8020	Membrane protein	WQG_7670	Membrane protein
4	**FS42_14390**	Nitroreductase	FS43_16050	Nitroreductase	FS44_8010	Nitroreductase	WQG_7660	Nitroreductase
4	**FS42_1440**	Ubiquinone/menaquinone biosynthesis methyltransferase ubiE	FS43_1480	Ubiquinone/menaquinone biosynthesis methyltransferase ubiE	FS44_21690	Ubiquinone/menaquinone biosynthesis methyltransferase ubiE	WQG_21150	Ubiquinone/menaquinone biosynthesis methyltransferase ubiE
4	**FS42_14400**	Peptide transport system ATP-binding protein SapF	FS43_16060	Peptide transport system ATP-binding protein SapF	FS44_8000	Peptide transport system ATP-binding protein SapF	WQG_7650	Peptide transport system ATP-binding protein SapF
4	**FS42_14410**	Lipoprotein E	FS43_16070	Lipoprotein E	FS44_7990	Lipoprotein E	WQG_7640	Lipoprotein E
4	**FS42_14420**	Fructose-bisphosphate aldolase	FS43_16080	Fructose-bisphosphate aldolase	FS44_7980	Fructose-bisphosphate aldolase	WQG_7630	Fructose-bisphosphate aldolase
4	**FS42_14430**	Phosphoglycerate kinase	FS43_16090	Phosphoglycerate kinase	FS44_7970	Phosphoglycerate kinase	WQG_7620	Phosphoglycerate kinase
4	**FS42_14440**	transporter	FS43_16100	transporter	FS44_7960	transporter	WQG_7610	transporter
4	**FS42_14450**	Heme exporter protein D	FS43_16110	Heme exporter protein D	FS44_7950	Heme exporter protein D	WQG_7600	Heme exporter protein D
4	**FS42_14460**	CcmC	FS43_16120	CcmC	FS44_7940	CcmC	WQG_7590	CcmC
4	**FS42_14470**	Heme exporter protein B	FS43_16130	Heme exporter protein B	FS44_7930	Heme exporter protein B	WQG_7580	Heme exporter protein B
4	**FS42_14480**	Cytochrome c biogenesis ATP-binding export protein CcmA	FS43_16140	Cytochrome c biogenesis ATP-binding export protein CcmA	FS44_7920	Cytochrome c biogenesis ATP-binding export protein CcmA	WQG_7570	Cytochrome c biogenesis ATP-binding export protein CcmA
4	**FS42_14490**	hypothetical protein	FS43_16150	hypothetical protein	FS44_7910	hypothetical protein	WQG_7560	hypothetical protein
4	**FS42_1450**	SCP-2 sterol transfer	FS43_1490	SCP-2 sterol transfer	FS44_21680	SCP-2 sterol transfer	WQG_21140	SCP-2 sterol transfer
4	**FS42_14500**	Nicotinamide mononucleotide transporter	FS43_16160	Nicotinamide mononucleotide transporter	FS44_7900	Nicotinamide mononucleotide transporter	WQG_7550	Nicotinamide mononucleotide transporter
4	**FS42_14510**	hypothetical protein	FS43_16170	hypothetical protein	FS44_7890	hypothetical protein	WQG_7540	hypothetical protein
4	**FS42_14520**	Histidyl-tRNA synthetase	FS43_16180	Histidyl-tRNA synthetase	FS44_7880	Histidyl-tRNA synthetase	WQG_7530	Histidyl-tRNA synthetase
4	**FS42_14530**	4-hydroxy-3-methylbut-2-en-1-yl diphosphate synthase	FS43_16190	4-hydroxy-3-methylbut-2-en-1-yl diphosphate synthase	FS44_7870	4-hydroxy-3-methylbut-2-en-1-yl diphosphate synthase	WQG_7520	4-hydroxy-3-methylbut-2-en-1-yl diphosphate synthase
4	**FS42_14540**	hypothetical protein	FS43_16200	hypothetical protein	FS44_7860	hypothetical protein	WQG_7510	hypothetical protein
4	**FS42_14550**	hypothetical protein	FS43_16210	hypothetical protein	FS44_7850	hypothetical protein	WQG_7500	hypothetical protein
4	**FS42_14560**	Thymidylate synthase	FS43_16220	Thymidylate synthase	FS44_7840	Thymidylate synthase	WQG_7490	Thymidylate synthase
4	**FS42_14570**	Nickel-binding periplasmic protein nikA	FS43_16230	Nickel-binding periplasmic protein nikA	FS44_7830	Nickel-binding periplasmic protein nikA	WQG_7480	Nickel-binding periplasmic protein nikA
4	**FS42_14580**	Binding-protein-dependent transport system inner membrane component	FS43_16240	Binding-protein-dependent transport system inner membrane component	FS44_7820	Binding-protein-dependent transport system inner membrane component	WQG_7470	Binding-protein-dependent transport system inner membrane component
4	**FS42_14590**	Nickel transport system permease protein	FS43_16250	Nickel transport system permease protein	FS44_7810	Nickel transport system permease protein	WQG_7460	Nickel transport system permease protein
4	**FS42_1460**	ubiquinone biosynthesis protein UbiB	FS43_1500	ubiquinone biosynthesis protein UbiB	FS44_21670	ubiquinone biosynthesis protein UbiB	WQG_21130	ubiquinone biosynthesis protein UbiB
4	**FS42_14600**	ABC transporter ATP-binding protein	FS43_16260	ABC transporter ATP-binding protein	FS44_7800	ABC transporter ATP-binding protein	WQG_7450	ABC transporter ATP-binding protein
4	**FS42_14610**	Sugar phosphate permease, required for N-linked glycosylation	FS43_16270	Sugar phosphate permease, required for N-linked glycosylation	FS44_7790	Sugar phosphate permease, required for N-linked glycosylation	WQG_7440	Sugar phosphate permease, required for N-linked glycosylation
4	**FS42_14620**	GTP-dependent nucleic acid-binding protein engD	FS43_16280	GTP-dependent nucleic acid-binding protein engD	FS44_7780	GTP-dependent nucleic acid-binding protein engD	WQG_7430	GTP-dependent nucleic acid-binding protein engD
4	**FS42_14630**	Peptidyl-tRNA hydrolase	FS43_16290	Peptidyl-tRNA hydrolase	FS44_7770	Peptidyl-tRNA hydrolase	WQG_7420	Peptidyl-tRNA hydrolase
4	**FS42_14640**	hypothetical protein	FS43_16300	hypothetical protein	FS44_7760	hypothetical protein	WQG_7410	hypothetical protein
4	**FS42_14650**	ATP-dependent RNA helicase SrmB	FS43_16310	ATP-dependent RNA helicase SrmB	FS44_7750	ATP-dependent RNA helicase SrmB	WQG_7400	ATP-dependent RNA helicase SrmB
4	**FS42_14660**	Ribosomal-protein-alanine acetyltransferase	FS43_16320	Ribosomal-protein-alanine acetyltransferase	FS44_7740	Ribosomal-protein-alanine acetyltransferase	WQG_7390	Ribosomal-protein-alanine acetyltransferase
4	**FS42_14680**	DNA polymerase III psi subunit protein	FS43_16340	DNA polymerase III psi subunit protein	FS44_7720	DNA polymerase III psi subunit protein	WQG_7370	DNA polymerase III psi subunit protein
4	**FS42_14690**	Ribosomal RNA small subunit methyltransferase C	FS43_16350	Ribosomal RNA small subunit methyltransferase C	FS44_7710	Ribosomal RNA small subunit methyltransferase C	WQG_7360	Ribosomal RNA small subunit methyltransferase C
4	**FS42_14700**	Glutaredoxin-4	FS43_16360	Glutaredoxin-4	FS44_7700	Glutaredoxin-4	WQG_7350	Glutaredoxin-4
4	**FS42_14710**	ABC transporter ATP-binding protein	FS43_16370	ABC transporter ATP-binding protein	FS44_7690	ABC transporter ATP-binding protein	WQG_7340	ABC transporter ATP-binding protein
4	**FS42_14720**	Autotransporter adhesin	FS43_16390	Autotransporter adhesin	FS44_7680	Autotransporter adhesin	WQG_7330	Autotransporter adhesin
4	**FS42_14730**	hypothetical protein	FS43_16400	hypothetical protein	FS44_7670	hypothetical protein	WQG_7320	hypothetical protein
4	**FS42_14740**	hypothetical protein	FS43_16410	hypothetical protein	FS44_7660	hypothetical protein	WQG_7310	hypothetical protein
4	**FS42_14750**	DMT superfamily drug/metabolite transporter	FS43_16420	DMT superfamily drug/metabolite transporter	FS44_7650	DMT superfamily drug/metabolite transporter	WQG_7300	DMT superfamily drug/metabolite transporter
4	**FS42_14760**	Lactoylglutathione lyase	FS43_16430	Lactoylglutathione lyase	FS44_7640	Lactoylglutathione lyase	WQG_7290	Lactoylglutathione lyase
4	**FS42_14770**	Ribonuclease T	FS43_16440	Ribonuclease T	FS44_7630	Ribonuclease T	WQG_7280	Ribonuclease T
4	**FS42_14780**	hypothetical protein	FS43_16450	hypothetical protein	FS44_7620	hypothetical protein	WQG_7270	hypothetical protein
4	**FS42_14790**	Transferrin binding protein B	FS43_16460	Transferrin binding protein B	FS44_7610	Transferrin binding protein B	WQG_7260	Transferrin binding protein B
4	**FS42_14800**	Transferrin binding protein A	FS43_16470	Transferrin binding protein A	FS44_7600	Transferrin binding protein A	WQG_7250	Transferrin binding protein A
4	**FS42_14810**	hypothetical protein	FS43_16480	hypothetical protein	FS44_7590	hypothetical protein	WQG_7240	hypothetical protein
4	**FS42_14820**	Spermidine/putrescine import ATP-binding protein PotA	FS43_16490	Spermidine/putrescine import ATP-binding protein PotA	FS44_7580	Spermidine/putrescine import ATP-binding protein PotA	WQG_7230	Spermidine/putrescine import ATP-binding protein PotA
4	**FS42_14830**	Spermidine/putrescine transport system permease protein PotB	FS43_16500	Spermidine/putrescine transport system permease protein PotB	FS44_7570	Spermidine/putrescine transport system permease protein PotB	WQG_7220	Spermidine/putrescine transport system permease protein PotB
4	**FS42_14840**	Spermidine/putrescine transport system permease protein PotC	FS43_16510	Spermidine/putrescine transport system permease protein PotC	FS44_7560	Spermidine/putrescine transport system permease protein PotC	WQG_7210	Spermidine/putrescine transport system permease protein PotC
4	**FS42_14850**	Holliday junction DNA helicase motor protein	FS43_16520	Holliday junction DNA helicase motor protein	FS44_7550	Holliday junction DNA helicase motor protein	WQG_7200	Holliday junction DNA helicase motor protein
4	**FS42_14860**	Holliday junction ATP-dependent DNA helicase RuvB	FS43_16530	Holliday junction ATP-dependent DNA helicase RuvB	FS44_7540	Holliday junction ATP-dependent DNA helicase RuvB	WQG_7190	Holliday junction ATP-dependent DNA helicase RuvB
4	**FS42_14870**	hypothetical protein	FS43_16540	hypothetical protein	FS44_7530	hypothetical protein	WQG_7180	hypothetical protein
4	**FS42_14880**	Rhizobiocin/RTX toxin	FS43_16550	Rhizobiocin/RTX toxin	FS44_7520	Rhizobiocin/RTX toxin	WQG_7170	Rhizobiocin/RTX toxin
4	**FS42_14890**	rRNA methyltransferase	FS43_16560	rRNA methyltransferase	FS44_7510	rRNA methyltransferase	WQG_7160	rRNA methyltransferase
4	**FS42_14900**	Inositol-1-monophosphatase	FS43_16570	Inositol-1-monophosphatase	FS44_7500	Inositol-1-monophosphatase	WQG_7150	Inositol-1-monophosphatase
4	**FS42_14910**	1-deoxy-D-xylulose-5-phosphate synthase	FS43_16580	1-deoxy-D-xylulose-5-phosphate synthase	FS44_7490	1-deoxy-D-xylulose-5-phosphate synthase	WQG_7140	1-deoxy-D-xylulose-5-phosphate synthase
4	**FS42_14920**	DNA-binding protein for site-specific recombination and inversion	FS43_16590	DNA-binding protein for site-specific recombination and inversion	FS44_7480	DNA-binding protein for site-specific recombination and inversion	WQG_7130	DNA-binding protein for site-specific recombination and inversion
4	**FS42_14930**	tRNA-dihydrouridine synthase B	FS43_16600	tRNA-dihydrouridine synthase B	FS44_7470	tRNA-dihydrouridine synthase B	WQG_7120	tRNA-dihydrouridine synthase B
4	**FS42_14940**	protease sohB	FS43_16610	protease sohB	FS44_7460	protease sohB	WQG_7110	protease sohB
4	**FS42_14950**	Thiosulfate sulfurtransferase glpE	FS43_16620	Thiosulfate sulfurtransferase glpE	FS44_7450	Thiosulfate sulfurtransferase glpE	WQG_7100	Thiosulfate sulfurtransferase glpE
4	**FS42_14960**	transport protein	FS43_16630	transport protein	FS44_7440	transport protein	WQG_7090	transport protein
4	**FS42_14970**	transport protein	FS43_16640	transport protein	FS44_7430	transport protein	WQG_7080	transport protein
4	**FS42_14980**	TonB2 energy transducing protein	FS43_16650	TonB2 energy transducing protein	FS44_7420	TonB2 energy transducing protein	WQG_7070	TonB2 energy transducing protein
4	**FS42_14990**	hypothetical protein	FS43_16660	hypothetical protein	FS44_7410	hypothetical protein	WQG_7060	hypothetical protein
4	**FS42_150**	Inosose dehydratase	FS43_160	Inosose dehydratase	FS44_22980	Inosose dehydratase	WQG_22460	Inosose dehydratase
4	**FS42_1500**	Sec-independent protein translocase protein TatA	FS43_1540	Sec-independent protein translocase protein TatA	FS44_21660	Sec-independent protein translocase protein TatA	WQG_21090	Sec-independent protein translocase protein TatA
4	**FS42_15000**	hypothetical protein	FS43_16670	hypothetical protein	FS44_7400	hypothetical protein	WQG_7050	hypothetical protein
4	**FS42_15010**	Lipoprotein Plp4/outer membrane protein and peptidoglycan-associated (Lipo)protein	FS43_16680	Lipoprotein Plp4/outer membrane protein and peptidoglycan-associated (Lipo)protein	FS44_7390	Lipoprotein Plp4/outer membrane protein and peptidoglycan-associated (Lipo)protein	WQG_7040	Lipoprotein Plp4/outer membrane protein and peptidoglycan-associated (Lipo)protein
4	**FS42_15050**	DNA helicase II	FS43_16720	DNA helicase II	FS44_7330	DNA helicase II	WQG_7000	DNA helicase II
4	**FS42_15060**	tRNA (cytidine(34)-2'-O)-methyltransferase	FS43_16730	tRNA (cytidine(34)-2'-O)-methyltransferase	FS44_7320	tRNA (cytidine(34)-2'-O)-methyltransferase	WQG_6990	tRNA (cytidine(34)-2'-O)-methyltransferase
4	**FS42_15070**	Ribosomal RNA large subunit methyltransferase L	FS43_16740	Ribosomal RNA large subunit methyltransferase L	FS44_7310	Ribosomal RNA large subunit methyltransferase L	WQG_6980	Ribosomal RNA large subunit methyltransferase L
4	**FS42_15090**	N-acetyl-gamma-glutamyl-phosphate reductase	FS43_16760	N-acetyl-gamma-glutamyl-phosphate reductase	FS44_7290	N-acetyl-gamma-glutamyl-phosphate reductase	WQG_6960	N-acetyl-gamma-glutamyl-phosphate reductase
4	**FS42_1510**	Sec-independent protein translocase protein TatB	FS43_1550	Sec-independent protein translocase protein TatB	FS44_21650	Sec-independent protein translocase protein TatB	WQG_21080	Sec-independent protein translocase protein TatB
4	**FS42_15100**	Arginine biosynthesis bifunctional protein ArgJ	FS43_16770	Arginine biosynthesis bifunctional protein ArgJ	FS44_7280	Arginine biosynthesis bifunctional protein ArgJ	WQG_6950	Arginine biosynthesis bifunctional protein ArgJ
4	**FS42_15110**	Acetylglutamate kinase	FS43_16780	Acetylglutamate kinase	FS44_7270	Acetylglutamate kinase	WQG_6940	Acetylglutamate kinase
4	**FS42_15120**	Acetylornithine aminotransferase	FS43_16790	Acetylornithine aminotransferase	FS44_7260	Acetylornithine aminotransferase	WQG_6930	Acetylornithine aminotransferase
4	**FS42_15130**	Biofilm PGA synthesis protein PgaA	FS43_16800	Biofilm PGA synthesis protein PgaA	FS44_7250	Biofilm PGA synthesis protein PgaA	WQG_6920	Biofilm PGA synthesis protein PgaA
4	**FS42_15140**	Biofilm PGA synthesis lipoprotein PgaB	FS43_16810	Biofilm PGA synthesis lipoprotein PgaB	FS44_7240	Biofilm PGA synthesis lipoprotein PgaB	WQG_6910	Biofilm PGA synthesis lipoprotein PgaB
4	**FS42_15150**	Biofilm PGA synthesis N-glycosyltransferase PgaC	FS43_16820	Biofilm PGA synthesis N-glycosyltransferase PgaC	FS44_7230	Biofilm PGA synthesis N-glycosyltransferase PgaC	WQG_6900	Biofilm PGA synthesis N-glycosyltransferase PgaC
4	**FS42_15160**	membrane protein YoaT	FS43_16830	membrane protein YoaT	FS44_7220	membrane protein YoaT	WQG_6890	hypothetical protein
4	**FS42_15180**	MazG nucleotide pyrophosphohydrolase	FS43_16850	MazG nucleotide pyrophosphohydrolase	FS44_7210	MazG nucleotide pyrophosphohydrolase	WQG_6880	MazG nucleotide pyrophosphohydrolase
4	**FS42_15190**	UvrABC system protein C	FS43_16860	UvrABC system protein C	FS44_7200	UvrABC system protein C	WQG_6870	UvrABC system protein C
4	**FS42_1520**	Sec-independent protein translocase protein TatC	FS43_1560	Sec-independent protein translocase protein TatC	FS44_21640	Sec-independent protein translocase protein TatC	WQG_21070	Sec-independent protein translocase protein TatC
4	**FS42_15200**	CDP-diacylglycerol--glycerol-3-phosphate 3-phosphatidyltransferase	FS43_16870	CDP-diacylglycerol--glycerol-3-phosphate 3-phosphatidyltransferase	FS44_7190	CDP-diacylglycerol--glycerol-3-phosphate 3-phosphatidyltransferase	WQG_6860	CDP-diacylglycerol--glycerol-3-phosphate 3-phosphatidyltransferase
4	**FS42_15210**	Protease 4	FS43_16880	Protease 4	FS44_7180	Protease 4	WQG_6850	Protease 4
4	**FS42_15220**	NADPH nitroreductase	FS43_16890	NADPH nitroreductase	FS44_7170	NADPH nitroreductase	WQG_6840	NADPH nitroreductase
4	**FS42_15230**	hypothetical protein	FS43_16900	hypothetical protein	FS44_7160	hypothetical protein	WQG_6830	hypothetical protein
4	**FS42_15240**	3-deoxy-manno-octulosonate cytidylyltransferase 1	FS43_16910	3-deoxy-manno-octulosonate cytidylyltransferase 1	FS44_7150	3-deoxy-manno-octulosonate cytidylyltransferase 1	WQG_6820	3-deoxy-manno-octulosonate cytidylyltransferase 1
4	**FS42_15250**	Lipoprotein VacJ-like protein	FS43_16920	Lipoprotein VacJ-like protein	FS44_7140	Lipoprotein VacJ-like protein	WQG_6810	Lipoprotein VacJ-like protein
4	**FS42_15260**	Glyoxalase II	FS43_16930	Glyoxalase II	FS44_7130	Glyoxalase II	WQG_6800	Glyoxalase II
4	**FS42_15270**	Twin-arginine translocation pathway signal	FS43_16940	Twin-arginine translocation pathway signal	FS44_7120	Twin-arginine translocation pathway signal	WQG_6790	Twin-arginine translocation pathway signal
4	**FS42_15280**	Periplasmic protein	FS43_16950	Periplasmic protein	FS44_7110	Periplasmic protein	WQG_6780	Periplasmic protein
4	**FS42_15290**	Tail-specific protease	FS43_16960	Tail-specific protease	FS44_7100	Tail-specific protease	WQG_6770	Tail-specific protease
4	**FS42_1530**	Lipopolysaccharide heptosyltransferase 1	FS43_1570	Lipopolysaccharide heptosyltransferase 1	FS44_21630	Lipopolysaccharide heptosyltransferase 1	WQG_21060	Lipopolysaccharide heptosyltransferase 1
4	**FS42_15300**	Protein proQ	FS43_16970	Protein proQ	FS44_7090	Protein proQ	WQG_6760	Protein proQ
4	**FS42_15310**	Thioredoxin reductase	FS43_16980	Thioredoxin reductase	FS44_7080	Thioredoxin reductase	WQG_6750	Thioredoxin reductase
4	**FS42_15320**	hypothetical protein	FS43_16990	hypothetical protein	FS44_7070	hypothetical protein	WQG_6740	hypothetical protein
4	**FS42_15330**	hypothetical protein	FS43_17000	hypothetical protein	FS44_7060	hypothetical protein	WQG_6730	hypothetical protein
4	**FS42_15340**	Malate dehydrogenase	FS43_17010	Malate dehydrogenase	FS44_7050	Malate dehydrogenase	WQG_6720	Malate dehydrogenase
4	**FS42_15350**	Arginine repressor	FS43_17020	Arginine repressor	FS44_7040	Arginine repressor	WQG_6710	Arginine repressor
4	**FS42_15360**	NAD-dependent epimerase/dehydratase	FS43_17030	NAD-dependent epimerase/dehydratase	FS44_7030	NAD-dependent epimerase/dehydratase	WQG_6700	NAD-dependent epimerase/dehydratase
4	**FS42_15370**	Ribosomal large subunit pseudouridine synthase D	FS43_17040	Ribosomal large subunit pseudouridine synthase D	FS44_7020	Ribosomal large subunit pseudouridine synthase D	WQG_6690	Ribosomal large subunit pseudouridine synthase D
4	**FS42_15380**	UPF0169 lipoprotein	FS43_17050	UPF0169 lipoprotein	FS44_7010	UPF0169 lipoprotein	WQG_6680	UPF0169 lipoprotein
4	**FS42_15390**	tRNA threonylcarbamoyladenosine biosynthesis protein Gcp	FS43_17060	tRNA threonylcarbamoyladenosine biosynthesis protein Gcp	FS44_7000	tRNA threonylcarbamoyladenosine biosynthesis protein Gcp	WQG_6670	tRNA threonylcarbamoyladenosine biosynthesis protein Gcp
4	**FS42_1540**	DNA-directed RNA polymerase subunit omega	FS43_1580	DNA-directed RNA polymerase subunit omega	FS44_21620	DNA-directed RNA polymerase subunit omega	WQG_21050	DNA-directed RNA polymerase subunit omega
4	**FS42_15400**	Lipid A biosynthesis (KDO)2-(lauroyl)-lipid IVA acyltransferase	FS43_17070	Lipid A biosynthesis (KDO)2-(lauroyl)-lipid IVA acyltransferase	FS44_6990	Lipid A biosynthesis (KDO)2-(lauroyl)-lipid IVA acyltransferase	WQG_6660	Lipid A biosynthesis (KDO)2-(lauroyl)-lipid IVA acyltransferase
4	**FS42_15420**	Electron transport complex protein rnfG	FS43_17080	Electron transport complex protein rnfG	FS44_6970	Electron transport complex protein rnfG	WQG_6640	Electron transport complex protein rnfG
4	**FS42_15430**	Electron transport complex protein rnfD	FS43_17090	Electron transport complex protein rnfD	FS44_6960	Electron transport complex protein rnfD	WQG_6630	Electron transport complex protein rnfD
4	**FS42_15440**	membrane protein	FS43_17100	membrane protein	FS44_6950	membrane protein	WQG_6620	membrane protein
4	**FS42_15450**	YbaK/prolyl-tRNA synthetase-associated domain protein	FS43_17110	YbaK/prolyl-tRNA synthetase-associated domain protein	FS44_6940	YbaK/prolyl-tRNA synthetase-associated domain protein	WQG_6610	YbaK/prolyl-tRNA synthetase-associated domain protein
4	**FS42_15460**	Electron transport complex protein rnfC	FS43_17130	Electron transport complex protein rnfC	FS44_6920	Electron transport complex protein rnfC	WQG_6600	Electron transport complex protein rnfC
4	**FS42_15470**	Electron transport complex protein rnfB	FS43_17140	Electron transport complex protein rnfB	FS44_6910	Electron transport complex protein rnfB	WQG_6590	Electron transport complex protein rnfB
4	**FS42_15490**	Electron transport complex protein rnfA	FS43_17160	Electron transport complex protein rnfA	FS44_6900	Electron transport complex protein rnfA	WQG_6570	Electron transport complex protein rnfA
4	**FS42_1550**	Guanosine-3',5'-bis(Diphosphate) 3'-pyrophosphohydrolase	FS43_1590	Guanosine-3',5'-bis(Diphosphate) 3'-pyrophosphohydrolase	FS44_21610	Guanosine-3',5'-bis(Diphosphate) 3'-pyrophosphohydrolase	WQG_21040	Guanosine-3',5'-bis(Diphosphate) 3'-pyrophosphohydrolase
4	**FS42_15500**	Acylphosphatase	FS43_17170	Acylphosphatase	FS44_6890	Acylphosphatase	WQG_6560	Acylphosphatase
4	**FS42_15510**	BtuC protein	FS43_17180	BtuC protein	FS44_6880	BtuC protein	WQG_6550	BtuC protein
4	**FS42_15520**	FepC protein	FS43_17190	FepC protein	FS44_6870	FepC protein	WQG_6540	FepC protein
4	**FS42_15530**	tRNA-specific 2-thiouridylase mnmA	FS43_17200	tRNA-specific 2-thiouridylase mnmA	FS44_6860	tRNA-specific 2-thiouridylase mnmA	WQG_6530	tRNA-specific 2-thiouridylase mnmA
4	**FS42_15540**	DNA polymerase beta domain protein region	FS43_17210	DNA polymerase beta domain protein region	FS44_6840	DNA polymerase beta domain protein region	WQG_6520	DNA polymerase beta domain protein region
4	**FS42_15550**	Nucleotidyltransferase substrate binding protein	FS43_17220	Nucleotidyltransferase substrate binding protein	FS44_6830	Nucleotidyltransferase substrate binding protein	WQG_6510	Nucleotidyltransferase substrate binding protein
4	**FS42_15560**	4-oxalocrotonate tautomerase	FS43_17230	4-oxalocrotonate tautomerase	FS44_6820	4-oxalocrotonate tautomerase	WQG_6500	4-oxalocrotonate tautomerase
4	**FS42_15570**	hypothetical protein	FS43_17240	hypothetical protein	FS44_6810	hypothetical protein	WQG_6490	hypothetical protein
4	**FS42_15580**	Thiamine biosynthesis lipoprotein ApbE	FS43_17250	Thiamine biosynthesis lipoprotein ApbE	FS44_6800	Thiamine biosynthesis lipoprotein ApbE	WQG_6480	Thiamine biosynthesis lipoprotein ApbE
4	**FS42_15590**	DNA polymerase beta subunit	FS43_17260	DNA polymerase beta subunit	FS44_6790	DNA polymerase beta subunit	WQG_6470	DNA polymerase beta subunit
4	**FS42_1560**	reductase	FS43_1600	reductase	FS44_21600	reductase	WQG_21030	reductase
4	**FS42_15600**	Nucleotidyltransferase substrate binding protein	FS43_17270	Nucleotidyltransferase substrate binding protein	FS44_6780	Nucleotidyltransferase substrate binding protein	WQG_6460	Nucleotidyltransferase substrate binding protein
4	**FS42_15610**	reductase	FS43_17280	reductase	FS44_6770	reductase	WQG_6450	reductase
4	**FS42_15620**	reductase	FS43_17290	reductase	FS44_6760	reductase	WQG_6440	reductase
4	**FS42_15630**	reductase	FS43_17300	reductase	FS44_6750	reductase	WQG_6430	reductase
4	**FS42_15640**	reductase	FS43_17310	reductase	FS44_6740	reductase	WQG_6420	reductase
4	**FS42_15650**	reductase	FS43_17320	reductase	FS44_6730	reductase	WQG_6410	reductase
4	**FS42_15660**	reductase	FS43_17330	reductase	FS44_6720	reductase	WQG_6400	reductase
4	**FS42_15670**	Fe/S biogenesis protein nfuA	FS43_17340	Fe/S biogenesis protein nfuA	FS44_6710	Fe/S biogenesis protein nfuA	WQG_6390	Fe/S biogenesis protein nfuA
4	**FS42_15680**	tRNA-dihydrouridine synthase A	FS43_17350	tRNA-dihydrouridine synthase A	FS44_6690	tRNA-dihydrouridine synthase A	WQG_6380	tRNA-dihydrouridine synthase A
4	**FS42_15690**	Periplasmic dipeptide transport protein	FS43_17360	Periplasmic dipeptide transport protein	FS44_6680	Periplasmic dipeptide transport protein	WQG_6370	Periplasmic dipeptide transport protein
4	**FS42_1570**	reductase	FS43_1610	reductase	FS44_21590	reductase	WQG_21020	reductase
4	**FS42_15700**	Periplasmic dipeptide transport protein	FS43_17380	Periplasmic dipeptide transport protein	FS44_6670	Periplasmic dipeptide transport protein	WQG_6350	Periplasmic dipeptide transport protein

4	**FS42_15710**	Dipeptide transport system permease protein dppB	FS43_17390	Dipeptide transport system permease protein dppB	FS44_6660	Dipeptide transport system permease protein dppB	WQG_6340	Dipeptide transport system permease protein dppB
4	**FS42_15720**	Dipeptide transport system permease protein dppC	FS43_17400	Dipeptide transport system permease protein dppC	FS44_6650	Dipeptide transport system permease protein dppC	WQG_6330	Dipeptide transport system permease protein dppC
4	**FS42_15730**	Dipeptide transport ATP-binding protein DppD	FS43_17410	Dipeptide transport ATP-binding protein DppD	FS44_6640	Dipeptide transport ATP-binding protein DppD	WQG_6320	Dipeptide transport ATP-binding protein DppD
4	**FS42_15740**	GCN5-related N-acetyltransferase	FS43_17420	GCN5-related N-acetyltransferase	FS44_6630	GCN5-related N-acetyltransferase	WQG_6310	GCN5-related N-acetyltransferase
4	**FS42_15750**	Dipeptide transport ATP-binding protein DppF	FS43_17430	Dipeptide transport ATP-binding protein DppF	FS44_6620	Dipeptide transport ATP-binding protein DppF	WQG_6300	Dipeptide transport ATP-binding protein DppF
4	**FS42_15760**	CitT protein	FS43_17440	CitT protein	FS44_6610	CitT protein	WQG_6290	CitT protein
4	**FS42_15770**	Menaquinone-specific isochorismate synthase	FS43_17450	Menaquinone-specific isochorismate synthase	FS44_6600	Menaquinone-specific isochorismate synthase	WQG_6280	Menaquinone-specific isochorismate synthase
4	**FS42_15780**	Aminotransferase AlaT	FS43_17460	Aminotransferase AlaT	FS44_6590	Aminotransferase AlaT	WQG_6270	Aminotransferase AlaT
4	**FS42_15790**	permease	FS43_17470	permease	FS44_6580	permease	WQG_6260	permease
4	**FS42_1580**	ATP-dependent DNA helicase recG	FS43_1620	ATP-dependent DNA helicase recG	FS44_21580	ATP-dependent DNA helicase recG	WQG_21010	ATP-dependent DNA helicase recG
4	**FS42_15800**	RarD protein	FS43_17480	RarD protein	FS44_6570	RarD protein	WQG_6250	RarD protein
4	**FS42_15810**	Primosomal protein N'	FS43_17490	Primosomal protein N'	FS44_6560	Primosomal protein N'	WQG_6240	Primosomal protein N'
4	**FS42_15820**	FbpA	FS43_17500	FbpA	FS44_6550	FbpA	WQG_6230	FbpA
4	**FS42_15830**	Binding-protein-dependent transport system inner membrane component	FS43_17510	Binding-protein-dependent transport system inner membrane component	FS44_6540	Binding-protein-dependent transport system inner membrane component	WQG_6220	Binding-protein-dependent transport system inner membrane component
4	**FS42_15840**	ABC transporter protein	FS43_17520	ABC transporter protein	FS44_6530	ABC transporter protein	WQG_6210	ABC transporter protein
4	**FS42_15850**	Adenylate kinase	FS43_17530	Adenylate kinase	FS44_6520	Adenylate kinase	WQG_6200	Adenylate kinase
4	**FS42_15860**	Iron-regulated outer membrane protein	FS43_17540	Iron-regulated outer membrane protein	FS44_6510	Iron-regulated outer membrane protein	WQG_6190	Iron-regulated outer membrane protein
4	**FS42_15870**	ABC transport system periplasmic protein	FS43_17560	ABC transport system periplasmic protein	FS44_6500	ABC transport system periplasmic protein	WQG_6180	ABC transport system periplasmic protein
4	**FS42_15880**	Transport system permease protein	FS43_17570	Transport system permease protein	FS44_6490	Transport system permease protein	WQG_6170	Transport system permease protein
4	**FS42_15890**	Iron chelate ABC transporter, permease protein	FS43_17580	Iron chelate ABC transporter, permease protein	FS44_6480	Transport system permease protein	WQG_6160	Iron chelate ABC transporter, permease protein
4	**FS42_1590**	30S ribosomal protein S10	FS43_1630	30S ribosomal protein S10	FS44_21570	30S ribosomal protein S10	WQG_21000	30S ribosomal protein S10
4	**FS42_15900**	Iron(III) ABC transporter, ATP-binding protein	FS43_17590	Iron(III) ABC transporter, ATP-binding protein	FS44_6470	Iron(III) ABC transporter, ATP-binding protein	WQG_6150	Iron(III) ABC transporter, ATP-binding protein
4	**FS42_15910**	AmpG-related permease	FS43_17600	AmpG-related permease	FS44_6460	AmpG-related permease	WQG_6140	AmpG-related permease
4	**FS42_15920**	UDP-glucose 4-epimerase	FS43_17610	UDP-glucose 4-epimerase	FS44_6450	UDP-glucose 4-epimerase	WQG_6130	UDP-glucose 4-epimerase
4	**FS42_15930**	Pseudouridine synthase	FS43_17620	Pseudouridine synthase	FS44_6440	Pseudouridine synthase	WQG_6120	Pseudouridine synthase
4	**FS42_15940**	Bifunctional prephenate dehydratase/chorismate mutase	FS43_17630	Bifunctional prephenate dehydratase/chorismate mutase	FS44_6430	Bifunctional prephenate dehydratase/chorismate mutase	WQG_6110	Bifunctional prephenate dehydratase/chorismate mutase
4	**FS42_15950**	Glycosyltransferase, group 2	FS43_17640	Glycosyltransferase, group 2	FS44_6420	Glycosyltransferase, group 2	WQG_6100	Glycosyltransferase, group 2
4	**FS42_15960**	Succinyl-CoA ligase [ADP-forming] subunit alpha	FS43_17650	Succinyl-CoA ligase [ADP-forming] subunit alpha	FS44_6410	Succinyl-CoA ligase [ADP-forming] subunit alpha	WQG_6090	Succinyl-CoA ligase [ADP-forming] subunit alpha
4	**FS42_15970**	Succinyl-CoA ligase [ADP-forming] subunit beta	FS43_17660	Succinyl-CoA ligase [ADP-forming] subunit beta	FS44_6400	Succinyl-CoA ligase [ADP-forming] subunit beta	WQG_6080	Succinyl-CoA ligase [ADP-forming] subunit beta
4	**FS42_15980**	Oligopeptidase A	FS43_17670	Oligopeptidase A	FS44_6390	Oligopeptidase A	WQG_6070	Oligopeptidase A
4	**FS42_15990**	Iron-containing alcohol dehydrogenase	FS43_17680	Iron-containing alcohol dehydrogenase	FS44_6380	Iron-containing alcohol dehydrogenase	WQG_6060	Iron-containing alcohol dehydrogenase
4	**FS42_160**	Inositol 2-dehydrogenase	FS43_170	Inositol 2-dehydrogenase	FS44_22970	Inositol 2-dehydrogenase	WQG_22450	Inositol 2-dehydrogenase
4	**FS42_1600**	50S ribosomal protein L3	FS43_1640	50S ribosomal protein L3	FS44_21560	50S ribosomal protein L3	WQG_20990	50S ribosomal protein L3
4	**FS42_16000**	Pyruvate formate-lyase 1-activating enzyme	FS43_17690	Pyruvate formate-lyase 1-activating enzyme	FS44_6370	Pyruvate formate-lyase 1-activating enzyme	WQG_6050	Pyruvate formate-lyase 1-activating enzyme
4	**FS42_16010**	Coproporphyrinogen III oxidase	FS43_17700	Coproporphyrinogen III oxidase	FS44_6360	Coproporphyrinogen III oxidase	WQG_6040	Coproporphyrinogen III oxidase
4	**FS42_16020**	Formate acetyltransferase	FS43_17710	Formate acetyltransferase	FS44_6350	Formate acetyltransferase	WQG_6030	Formate acetyltransferase
4	**FS42_16030**	formate transporter	FS43_17720	formate transporter	FS44_6340	formate transporter	WQG_6020	formate transporter
4	**FS42_16040**	hypothetical protein	FS43_17730	hypothetical protein	FS44_6330	hypothetical protein	WQG_6010	hypothetical protein
4	**FS42_16050**	hypothetical protein	FS43_17740	hypothetical protein	FS44_6320	hypothetical protein	WQG_6000	hypothetical protein
4	**FS42_16060**	3'(2'),5'-bisphosphate nucleotidase CysQ	FS43_17750	3'(2'),5'-bisphosphate nucleotidase CysQ	FS44_6310	3'(2'),5'-bisphosphate nucleotidase CysQ	WQG_5990	3'(2'),5'-bisphosphate nucleotidase CysQ
4	**FS42_16070**	Glucose-6-phosphate 1-dehydrogenase	FS43_17760	Glucose-6-phosphate 1-dehydrogenase	FS44_6300	Glucose-6-phosphate 1-dehydrogenase	WQG_5980	Glucose-6-phosphate 1-dehydrogenase
4	**FS42_16080**	hypothetical protein	FS43_17770	hypothetical protein	FS44_6290	hypothetical protein	WQG_5970	hypothetical protein
4	**FS42_16090**	hypothetical protein	FS43_17780	hypothetical protein	FS44_6280	hypothetical protein	WQG_5960	hypothetical protein
4	**FS42_1610**	50S ribosomal protein L4	FS43_1650	50S ribosomal protein L4	FS44_21550	50S ribosomal protein L4	WQG_20980	50S ribosomal protein L4
4	**FS42_16100**	6-phosphogluconate dehydrogenase, decarboxylating	FS43_17790	6-phosphogluconate dehydrogenase, decarboxylating	FS44_6270	6-phosphogluconate dehydrogenase, decarboxylating	WQG_5950	6-phosphogluconate dehydrogenase, decarboxylating
4	**FS42_16110**	hypothetical protein	FS43_17800	hypothetical protein	FS44_6260	hypothetical protein	WQG_5940	hypothetical protein
4	**FS42_16120**	Cysteine synthetase	FS43_17810	Cysteine synthetase	FS44_6250	Cysteine synthetase	WQG_5930	Cysteine synthetase
4	**FS42_16130**	Cell division protein ZipA	FS43_17820	Cell division protein ZipA	FS44_6240	Cell division protein ZipA	WQG_5920	Cell division protein ZipA
4	**FS42_16140**	DNA ligase	FS43_17830	DNA ligase	FS44_6230	DNA ligase	WQG_5910	DNA ligase
4	**FS42_16150**	hypothetical protein	FS43_17840	hypothetical protein	FS44_6220	hypothetical protein	WQG_5900	hypothetical protein
4	**FS42_16160**	CTP synthase	FS43_17850	CTP synthase	FS44_6210	CTP synthase	WQG_5890	CTP synthase
4	**FS42_16170**	OMR family outer membrane receptor	FS43_17860	OMR family outer membrane receptor	FS44_6200	OMR family outer membrane receptor	WQG_5880	OMR family outer membrane receptor
4	**FS42_16180**	CirA protein	FS43_17870	CirA protein	FS44_6190	CirA protein	WQG_5870	CirA protein
4	**FS42_16190**	Argininosuccinate lyase	FS43_17880	Argininosuccinate lyase	FS44_6170	Argininosuccinate lyase	WQG_5860	Argininosuccinate lyase
4	**FS42_1620**	50S ribosomal protein L23	FS43_1660	50S ribosomal protein L23	FS44_21540	50S ribosomal protein L23	WQG_20970	50S ribosomal protein L23
4	**FS42_16200**	Xaa-Pro dipeptidase	FS43_17890	Xaa-Pro dipeptidase	FS44_6160	Xaa-Pro dipeptidase	WQG_5850	Xaa-Pro dipeptidase
4	**FS42_16210**	Sulfurtransferase TusA	FS43_17900	Sulfurtransferase TusA	FS44_6150	Sulfurtransferase TusA	WQG_5840	Sulfurtransferase TusA
4	**FS42_16220**	Regulator of ribonuclease activity B	FS43_17910	Regulator of ribonuclease activity B	FS44_6140	Regulator of ribonuclease activity B	WQG_5830	Regulator of ribonuclease activity B
4	**FS42_16230**	hypothetical protein	FS43_17920	hypothetical protein	FS44_6130	hypothetical protein	WQG_5820	hypothetical protein
4	**FS42_16240**	GTP-binding protein EngB	FS43_17930	GTP-binding protein EngB	FS44_6120	GTP-binding protein EngB	WQG_5810	GTP-binding protein EngB
4	**FS42_16250**	hypothetical protein	FS43_17940	hypothetical protein	FS44_6110	Membrane protein	WQG_5800	Membrane protein
4	**FS42_16260**	Aminopeptidase N	FS43_17950	Aminopeptidase N	FS44_6100	Aminopeptidase N	WQG_5790	Aminopeptidase N
4	**FS42_16270**	hypothetical protein	FS43_17960	hypothetical protein	FS44_6090	hypothetical protein	WQG_5780	hypothetical protein
4	**FS42_16280**	hypothetical protein	FS43_17970	hypothetical protein	FS44_6080	hypothetical protein	WQG_5770	hypothetical protein
4	**FS42_16290**	CDP-diacylglycerol--serine O-phosphatidyltransferase	FS43_17980	CDP-diacylglycerol--serine O-phosphatidyltransferase	FS44_6070	CDP-diacylglycerol--serine O-phosphatidyltransferase	WQG_5760	CDP-diacylglycerol--serine O-phosphatidyltransferase
4	**FS42_1630**	50S ribosomal protein L2	FS43_1670	50S ribosomal protein L2	FS44_21530	50S ribosomal protein L2	WQG_20960	50S ribosomal protein L2
4	**FS42_16300**	Isoleucyl-tRNA synthetase	FS43_17990	Isoleucyl-tRNA synthetase	FS44_6060	Isoleucyl-tRNA synthetase	WQG_5750	Isoleucyl-tRNA synthetase
4	**FS42_16310**	Riboflavin biosynthesis protein	FS43_18000	Riboflavin biosynthesis protein	FS44_6050	Riboflavin biosynthesis protein	WQG_5740	Riboflavin biosynthesis protein
4	**FS42_16340**	hypothetical protein	FS43_18030	hypothetical protein	FS44_6030	hypothetical protein	WQG_5710	hypothetical protein
4	**FS42_16350**	protease	FS43_18040	protease	FS44_6020	protease	WQG_5700	protease
4	**FS42_16360**	ABC transporter domain protein	FS43_18060	ABC transporter domain protein	FS44_6010	ABC transporter domain protein	WQG_5690	ABC transporter domain protein
4	**FS42_16370**	Peptidase M16 domain protein	FS43_18070	Peptidase M16 domain protein	FS44_6000	Peptidase M16 domain protein	WQG_5680	Peptidase M16 domain protein
4	**FS42_16380**	hypothetical protein	FS43_18080	hypothetical protein	FS44_5990	hypothetical protein	WQG_5670	hypothetical protein
4	**FS42_16390**	TonB-dependent receptor plug	FS43_18090	TonB-dependent receptor plug	FS44_5980	TonB-dependent receptor plug	WQG_5660	TonB-dependent receptor plug
4	**FS42_1640**	30S ribosomal protein S19	FS43_1680	30S ribosomal protein S19	FS44_21520	30S ribosomal protein S19	WQG_20950	30S ribosomal protein S19
4	**FS42_16400**	hypothetical protein	FS43_18100	hypothetical protein	FS44_5970	hypothetical protein	WQG_5650	hypothetical protein
4	**FS42_16410**	hypothetical protein	FS43_18110	hypothetical protein	FS44_5960	hypothetical protein	WQG_5640	hypothetical protein
4	**FS42_16420**	hypothetical protein	FS43_18120	hypothetical protein	FS44_5950	hypothetical protein	WQG_5630	hypothetical protein
4	**FS42_16430**	5-methyltetrahydropteroyltriglutamate-- homocysteine methyltransferase	FS43_18130	5-methyltetrahydropteroyltriglutamate-- homocysteine methyltransferase	FS44_5940	5-methyltetrahydropteroyltriglutamate-- homocysteine methyltransferase	WQG_5620	5-methyltetrahydropteroyltriglutamate-- homocysteine methyltransferase
4	**FS42_16440**	UDP-3-O-[3-hydroxymyristoyl] N-acetylglucosamine deacetylase	FS43_18140	UDP-3-O-[3-hydroxymyristoyl] N-acetylglucosamine deacetylase	FS44_5930	UDP-3-O-[3-hydroxymyristoyl] N-acetylglucosamine deacetylase	WQG_5610	UDP-3-O-[3-hydroxymyristoyl] N-acetylglucosamine deacetylase
4	**FS42_16450**	Cell division protein FtsZ/Cell division GTPase	FS43_18150	Cell division protein FtsZ/Cell division GTPase	FS44_5920	Cell division protein FtsZ/Cell division GTPase	WQG_5600	Cell division protein FtsZ/Cell division GTPase
4	**FS42_16460**	Cell division protein ftsA	FS43_18160	Cell division protein ftsA	FS44_5910	Cell division protein ftsA	WQG_5590	Cell division protein ftsA
4	**FS42_16470**	Cell division protein FtsQ	FS43_18170	Cell division protein FtsQ	FS44_5900	Cell division protein FtsQ	WQG_5580	Cell division protein FtsQ
4	**FS42_16480**	D-alanine--D-alanine ligase	FS43_18180	D-alanine--D-alanine ligase	FS44_5890	D-alanine--D-alanine ligase	WQG_5570	D-alanine--D-alanine ligase
4	**FS42_16490**	UDP-N-acetylmuramate--L-alanine ligase	FS43_18190	UDP-N-acetylmuramate--L-alanine ligase	FS44_5880	UDP-N-acetylmuramate--L-alanine ligase	WQG_5560	UDP-N-acetylmuramate--L-alanine ligase
4	**FS42_1650**	50S ribosomal protein L22	FS43_1690	50S ribosomal protein L22	FS44_21510	50S ribosomal protein L22	WQG_20940	50S ribosomal protein L22
4	**FS42_16500**	UDP-N-acetylglucosamine-N-acetylmuramyl pyrophosphoryl-undecaprenol N-acetylglucosamine transferase	FS43_18200	UDP-N-acetylglucosamine-N-acetylmuramyl pyrophosphoryl-undecaprenol N-acetylglucosamine transferase	FS44_5870	UDP-N-acetylglucosamine-N-acetylmuramyl pyrophosphoryl-undecaprenol N-acetylglucosamine transferase	WQG_5550	UDP-N-acetylglucosamine--N-acetylmuramyl- (pentapeptide) pyrophosphoryl-undecaprenol N-acetylglucosamine transferase
4	**FS42_16510**	FtsW protein	FS43_18210	FtsW protein	FS44_5860	FtsW protein	WQG_5540	FtsW protein
4	**FS42_16520**	UDP-N-acetylmuramoylalanine--D-glutamate ligase	FS43_18220	UDP-N-acetylmuramoylalanine--D-glutamate ligase	FS44_5850	UDP-N-acetylmuramoylalanine--D-glutamate ligase	WQG_5530	UDP-N-acetylmuramoylalanine--D-glutamate ligase
4	**FS42_16530**	ADP-ribosylation/crystallin J1	FS43_18240	ADP-ribosylation/crystallin J1	FS44_5830	ADP-ribosylation/crystallin J1	WQG_5510	ADP-ribosylation/crystallin J1
4	**FS42_16550**	Phospho-N-acetylmuramoyl-pentapeptide- transferase	FS43_18260	Phospho-N-acetylmuramoyl-pentapeptide- transferase	FS44_5820	Phospho-N-acetylmuramoyl-pentapeptide- transferase	WQG_5490	Phospho-N-acetylmuramoyl-pentapeptide- transferase
4	**FS42_16560**	UDP-N-acetylmuramoyl-tripeptide--D-alanyl-D- alanine ligase	FS43_18270	UDP-N-acetylmuramoyl-tripeptide--D-alanyl-D- alanine ligase	FS44_5810	UDP-N-acetylmuramoyl-tripeptide--D-alanyl-D- alanine ligase	WQG_5480	UDP-N-acetylmuramoyl-tripeptide--D-alanyl-D- alanine ligase
4	**FS42_16570**	UDP-N-acetylmuramoyl-L-alanyl-D-glutamate--2,6- diaminopimelate ligase	FS43_18280	UDP-N-acetylmuramoyl-L-alanyl-D-glutamate--2,6- diaminopimelate ligase	FS44_5800	UDP-N-acetylmuramoyl-L-alanyl-D-glutamate--2,6- diaminopimelate ligase	WQG_5470	UDP-N-acetylmuramoyl-L-alanyl-D-glutamate--2,6- diaminopimelate ligase
4	**FS42_16590**	Penicillin-binding protein 3	FS43_18290	Penicillin-binding protein 3	FS44_5790	Penicillin-binding protein 3	WQG_5460	Penicillin-binding protein 3
4	**FS42_1660**	30S ribosomal protein S3	FS43_1700	30S ribosomal protein S3	FS44_21500	30S ribosomal protein S3	WQG_20930	30S ribosomal protein S3
4	**FS42_16600**	Cell division protein FtsL	FS43_18300	Cell division protein FtsL	FS44_5780	Cell division protein FtsL	WQG_5450	Cell division protein FtsL
4	**FS42_16610**	Ribosomal RNA small subunit methyltransferase H	FS43_18310	Ribosomal RNA small subunit methyltransferase H	FS44_5770	Ribosomal RNA small subunit methyltransferase H	WQG_5440	Ribosomal RNA small subunit methyltransferase H
4	**FS42_16620**	MraZ protein	FS43_18320	MraZ protein	FS44_5760	MraZ protein	WQG_5430	MraZ protein
4	**FS42_16630**	Fatty acid metabolism regulator protein	FS43_18330	Fatty acid metabolism regulator protein	FS44_5750	Fatty acid metabolism regulator protein	WQG_5420	Fatty acid metabolism regulator protein
4	**FS42_16640**	Na(+)/H(+) antiporter nhaB	FS43_18340	Na(+)/H(+) antiporter nhaB	FS44_5740	Na(+)/H(+) antiporter nhaB	WQG_5410	Na(+)/H(+) antiporter nhaB
4	**FS42_16650**	Disulfide bond formation protein B	FS43_18350	Disulfide bond formation protein B	FS44_5730	Disulfide bond formation protein B	WQG_5400	Disulfide bond formation protein B
4	**FS42_16660**	Methionyl-tRNA synthetase	FS43_18370	Methionyl-tRNA synthetase	FS44_5720	Methionyl-tRNA synthetase	WQG_5390	Methionyl-tRNA synthetase
4	**FS42_16670**	Nucleoside diphosphate kinase	FS43_18380	Nucleoside diphosphate kinase	FS44_5710	Nucleoside diphosphate kinase	WQG_5380	Nucleoside diphosphate kinase
4	**FS42_16680**	PTS system, fructose-specific IIA/FPr component	FS43_18390	PTS system, fructose-specific IIA/FPr component	FS44_5700	PTS system, fructose-specific IIA/FPr component	WQG_5370	PTS system, fructose-specific IIA/FPr component
4	**FS42_16690**	1-phosphofructokinase	FS43_18400	1-phosphofructokinase	FS44_5690	1-phosphofructokinase	WQG_5360	1-phosphofructokinase
4	**FS42_1670**	50S ribosomal protein L16	FS43_1710	50S ribosomal protein L16	FS44_21490	50S ribosomal protein L16	WQG_20920	50S ribosomal protein L16
4	**FS42_16700**	FruA protein	FS43_18410	FruA protein	FS44_5680	FruA protein	WQG_5350	FruA protein
4	**FS42_16710**	RacX protein	FS43_18420	RacX protein	FS44_5670	RacX protein	WQG_5340	RacX protein
4	**FS42_16720**	Dihydroxy-acid dehydratase	FS43_18430	Dihydroxy-acid dehydratase	FS44_5660	Dihydroxy-acid dehydratase	WQG_5330	Dihydroxy-acid dehydratase
4	**FS42_16730**	GutA-like protein	FS43_18440	GutA-like protein	FS44_5640	GutA-like protein	WQG_5320	GutA-like protein
4	**FS42_16740**	4-alpha-glucanotransferase	FS43_18450	4-alpha-glucanotransferase	FS44_5630	4-alpha-glucanotransferase	WQG_5310	4-alpha-glucanotransferase
4	**FS42_16750**	Maltodextrin phosphorylase	FS43_18460	Maltodextrin phosphorylase	FS44_5610	Maltodextrin phosphorylase	WQG_5300	Maltodextrin phosphorylase
4	**FS42_16760**	HTH-type transcriptional regulator malT	FS43_18470	HTH-type transcriptional regulator malT	FS44_5600	HTH-type transcriptional regulator malT	WQG_5290	HTH-type transcriptional regulator malT
4	**FS42_16770**	Alpha-amylase	FS43_18480	Alpha-amylase	FS44_5590	Alpha-amylase	WQG_5280	Alpha-amylase
4	**FS42_16780**	Maltose transport system permease protein malG	FS43_18490	Maltose transport system permease protein malG	FS44_5580	Maltose transport system permease protein malG	WQG_5270	Maltose transport system permease protein malG
4	**FS42_16790**	MalF protein	FS43_18500	MalF protein	FS44_5570	MalF protein	WQG_5260	MalF protein
4	**FS42_1680**	50S ribosomal protein L29	FS43_1720	50S ribosomal protein L29	FS44_21480	50S ribosomal protein L29	WQG_20910	50S ribosomal protein L29
4	**FS42_16800**	Maltose-binding periplasmic protein	FS43_18510	Maltose-binding periplasmic protein	FS44_5560	Maltose-binding periplasmic protein	WQG_5250	Maltose-binding periplasmic protein
4	**FS42_16810**	Maltose/maltodextrin import ATP-binding protein MalK	FS43_18520	Maltose/maltodextrin import ATP-binding protein MalK	FS44_5540	Maltose/maltodextrin import ATP-binding protein MalK	WQG_5240	Maltose/maltodextrin import ATP-binding protein MalK
4	**FS42_16820**	Maltoporin	FS43_18530	Maltoporin	FS44_5530	Maltoporin	WQG_5230	Maltoporin
4	**FS42_16830**	Maltose operon periplasmic	FS43_18540	Maltose operon periplasmic	FS44_5520	Maltose operon periplasmic	WQG_5220	Maltose operon periplasmic
4	**FS42_16840**	hypothetical protein	FS43_18550	hypothetical protein	FS44_5510	hypothetical protein	WQG_5210	hypothetical protein
4	**FS42_16850**	Recombination protein recR	FS43_18560	Recombination protein recR	FS44_5500	Recombination protein recR	WQG_5200	Recombination protein recR
4	**FS42_16860**	GTP-binding protein TypA/BipA	FS43_18570	GTP-binding protein TypA/BipA	FS44_5490	GTP-binding protein TypA/BipA	WQG_5190	GTP-binding protein TypA/BipA
4	**FS42_16870**	Glutaredoxin	FS43_18580	Glutaredoxin	FS44_5480	Glutaredoxin	WQG_5180	Glutaredoxin
4	**FS42_16880**	Heme-binding protein A	FS43_18590	Heme-binding protein A	FS44_5470	Heme-binding protein A	WQG_5170	Heme-binding protein A
4	**FS42_16890**	Fructose-1,6-bisphosphatase class 1	FS43_18600	Fructose-1,6-bisphosphatase class 1	FS44_5460	Fructose-1,6-bisphosphatase class 1	WQG_5160	Fructose-1,6-bisphosphatase class 1
4	**FS42_1690**	30S ribosomal protein S17	FS43_1730	30S ribosomal protein S17	FS44_21470	30S ribosomal protein S17	WQG_20900	30S ribosomal protein S17
4	**FS42_16900**	UDP-N-acetylmuramate:L-alanyl-gamma-D-glutamyl- meso-diaminopimelate ligase	FS43_18610	UDP-N-acetylmuramate:L-alanyl-gamma-D-glutamyl- meso-diaminopimelate ligase	FS44_5450	UDP-N-acetylmuramate:L-alanyl-gamma-D-glutamyl- meso-diaminopimelate ligase	WQG_5150	UDP-N-acetylmuramate:L-alanyl-gamma-D-glutamyl- meso-diaminopimelate ligase
4	**FS42_16910**	Ribonuclease R	FS43_18620	Ribonuclease R	FS44_5430	Ribonuclease R	WQG_5140	Ribonuclease R
4	**FS42_16920**	Serine acetyltransferase	FS43_18640	Serine acetyltransferase	FS44_5420	Serine acetyltransferase	WQG_5130	Serine acetyltransferase
4	**FS42_16930**	Glycerol-3-phosphate dehydrogenase [NAD(P)+]	FS43_18660	Glycerol-3-phosphate dehydrogenase [NAD(P)+]	FS44_5410	Glycerol-3-phosphate dehydrogenase [NAD(P)+]	WQG_5120	Glycerol-3-phosphate dehydrogenase [NAD(P)+]
4	**FS42_16940**	Protein-export protein secB	FS43_18670	Protein-export protein secB	FS44_5400	Protein-export protein secB	WQG_5110	Protein-export protein secB
4	**FS42_16950**	Rhodanese-related sulfurtransferase	FS43_18680	Rhodanese-related sulfurtransferase	FS44_5390	Rhodanese-related sulfurtransferase	WQG_5100	Rhodanese-related sulfurtransferase
4	**FS42_16960**	Trigger factor	FS43_18690	Trigger factor	FS44_5380	Trigger factor	WQG_5090	Trigger factor
4	**FS42_16970**	hypothetical protein	FS43_18700	hypothetical protein	FS44_5370	hypothetical protein	WQG_5080	hypothetical protein
4	**FS42_16980**	XkdP protein	FS43_18710	XkdP protein	FS44_5360	XkdP protein	WQG_5070	XkdP protein
4	**FS42_16990**	Histidine biosynthesis bifunctional protein HisIE	FS43_18720	Histidine biosynthesis bifunctional protein HisIE	FS44_5350	Histidine biosynthesis bifunctional protein HisIE	WQG_5060	Histidine biosynthesis bifunctional protein HisIE
4	**FS42_1700**	Chromosomal replication initiator protein DnaA	FS43_1740	Chromosomal replication initiator protein DnaA	FS44_21460	Chromosomal replication initiator protein DnaA	WQG_20890	Chromosomal replication initiator protein DnaA
4	**FS42_17000**	Imidazole glycerol phosphate synthase subunit HisF	FS43_18730	Imidazole glycerol phosphate synthase subunit HisF	FS44_5340	Imidazole glycerol phosphate synthase subunit HisF	WQG_5050	Imidazole glycerol phosphate synthase subunit HisF
4	**FS42_17010**	1-(5-phosphoribosyl)-5-[(5- phosphoribosylamino)methylideneamino] imidazole-4-carboxamide isomerase	FS43_18740	1-(5-phosphoribosyl)-5-[(5- phosphoribosylamino)methylideneamino] imidazole-4-carboxamide isomerase	FS44_5330	1-(5-phosphoribosyl)-5-[(5- phosphoribosylamino)methylideneamino] imidazole-4-carboxamide isomerase	WQG_5040	1-(5-phosphoribosyl)-5-[(5- phosphoribosylamino)methylideneamino] imidazole-4-carboxamide isomerase
4	**FS42_17040**	Imidazole glycerol phosphate synthase subunit HisH	FS43_18770	Imidazole glycerol phosphate synthase subunit HisH	FS44_5300	Imidazole glycerol phosphate synthase subunit HisH	WQG_5010	Imidazole glycerol phosphate synthase subunit HisH
4	**FS42_17060**	Histidine biosynthesis bifunctional protein hisB	FS43_18790	Histidine biosynthesis bifunctional protein hisB	FS44_5280	Histidine biosynthesis bifunctional protein hisB	WQG_4990	Histidine biosynthesis bifunctional protein hisB
4	**FS42_17070**	Transposase IS200-like protein	FS43_18800	Transposase IS200-like protein	FS44_5270	Transposase IS200-like protein	WQG_4980	Transposase IS200-like protein

4	FS42_17080	Histidinol-phosphate aminotransferase	FS43_18810	Histidinol-phosphate aminotransferase	FS44_5260	Histidinol-phosphate aminotransferase	WQG_4970	Histidinol-phosphate aminotransferase
4	FS42_17090	GNAT family acetyltransferase	FS43_18820	GNAT family acetyltransferase	FS44_5250	GNAT family acetyltransferase	WQG_4960	GNAT family acetyltransferase
4	FS42_1710	DNA polymerase III subunit beta	FS43_1750	DNA polymerase III subunit beta	FS44_21450	DNA polymerase III subunit beta	WQG_20880	DNA polymerase III subunit beta
4	FS42_17100	Death-on-curing protein	FS43_18830	Death-on-curing protein	FS44_5230	Death-on-curing protein	WQG_4950	Death-on-curing protein
4	FS42_17110	Histidinol dehydrogenase	FS43_18840	Histidinol dehydrogenase	FS44_5220	Histidinol dehydrogenase	WQG_4940	Histidinol dehydrogenase
4	FS42_17120	ATP phosphoribosyltransferase	FS43_18850	ATP phosphoribosyltransferase	FS44_5210	ATP phosphoribosyltransferase	WQG_4930	ATP phosphoribosyltransferase
4	FS42_17130	Ribosomal RNA large subunit methyltransferase N	FS43_18860	Ribosomal RNA large subunit methyltransferase N	FS44_5200	Ribosomal RNA large subunit methyltransferase N	WQG_4920	Ribosomal RNA large subunit methyltransferase N
4	FS42_17140	hypothetical protein	FS43_18870	hypothetical protein	FS44_5190	hypothetical protein	WQG_4910	hypothetical protein
4	FS42_17150	Cell division protein ZapA	FS43_18880	Cell division protein ZapA	FS44_5180	Cell division protein ZapA	WQG_4900	Cell division protein ZapA
4	FS42_17160	5-formyltetrahydrofolate cyclo-ligase	FS43_18890	5-formyltetrahydrofolate cyclo-ligase	FS44_5170	5-formyltetrahydrofolate cyclo-ligase	WQG_4890	5-formyltetrahydrofolate cyclo-ligase
4	FS42_17170	hypothetical protein	FS43_18900	hypothetical protein	FS44_5160	putative membrane protein	WQG_4880	hypothetical protein
4	FS42_17180	HIT-like protein	FS43_18910	HIT-like protein	FS44_5150	HIT-like protein	WQG_4870	HIT-like protein
4	FS42_17190	Periplasmic lipoprotein	FS43_18920	Periplasmic lipoprotein	FS44_5140	Periplasmic lipoprotein	WQG_4860	Periplasmic lipoprotein
4	FS42_1720	DNA replication and repair protein recF	FS43_1770	DNA replication and repair protein recF	FS44_21440	DNA replication and repair protein recF	WQG_20870	DNA replication and repair protein recF
4	FS42_17200	Tyrosyl-tRNA synthetase 2	FS43_18930	Tyrosyl-tRNA synthetase 2	FS44_5130	Tyrosyl-tRNA synthetase 2	WQG_4850	Tyrosyl-tRNA synthetase 2
4	FS42_17210	Protease HtpX	FS43_18940	Protease HtpX	FS44_5120	Protease HtpX	WQG_4840	Protease HtpX
4	FS42_17220	sulfate transporter	FS43_18950	sulfate transporter	FS44_5110	sulfate transporter	WQG_4830	sulfate transporter
4	FS42_17230	Adenylosuccinate synthetase	FS43_18970	Adenylosuccinate synthetase	FS44_5090	Adenylosuccinate synthetase	WQG_4810	Adenylosuccinate synthetase
4	FS42_17240	Protein HflC	FS43_18980	Protein HflC	FS44_5080	Protein HflC	WQG_4800	Protein HflC
4	FS42_17250	HflK protein	FS43_18990	HflK protein	FS44_5070	HflK protein	WQG_4790	HflK protein
4	FS42_17260	hypothetical protein	FS43_19000	hypothetical protein	FS44_5060	hypothetical protein	WQG_4780	hypothetical protein
4	FS42_17270	NrfD protein	FS43_19020	NrfD protein	FS44_5050	NrfD protein	WQG_4770	NrfD protein
4	FS42_17280	reductase	FS43_19030	reductase	FS44_5040	reductase	WQG_4760	reductase
4	FS42_17290	Cytochrome c-type protein NrfB	FS43_19040	Cytochrome c-type protein NrfB	FS44_5030	Cytochrome c-type protein NrfB	WQG_4750	Cytochrome c-type protein NrfB
4	FS42_1730	Transcription-repair-coupling factor	FS43_1790	Transcription-repair-coupling factor	FS44_21430	Transcription-repair-coupling factor	WQG_20860	Transcription-repair-coupling factor
4	FS42_17300	Cytochrome c-552	FS43_19050	Cytochrome c-552	FS44_5020	Cytochrome c-552	WQG_4740	Cytochrome c-552
4	FS42_17310	hypothetical protein	FS43_19060	hypothetical protein	FS44_5010	hypothetical protein	WQG_4730	hypothetical protein
4	FS42_17320	Cell division protein FtsN, sporulation domain-containing protein	FS43_19070	Cell division protein FtsN, sporulation domain-containing protein	FS44_5000	Cell division protein FtsN, sporulation domain-containing protein	WQG_4720	Cell division protein FtsN, sporulation domain-containing protein
4	FS42_17330	Thiamine import ATP-binding protein ThiQ	FS43_19080	Thiamine import ATP-binding protein ThiQ	FS44_4990	Thiamine import ATP-binding protein ThiQ	WQG_4710	Thiamine import ATP-binding protein ThiQ
4	FS42_17340	Thiamine transport system permease protein ThiP	FS43_19090	Thiamine transport system permease protein ThiP	FS44_4980	Thiamine transport system permease protein ThiP	WQG_4700	Thiamine transport system permease protein ThiP
4	FS42_17350	Trk system potassium uptake protein trkA	FS43_19100	Trk system potassium uptake protein trkA	FS44_4970	Trk system potassium uptake protein trkA	WQG_4690	Trk system potassium uptake protein trkA
4	FS42_17360	Ribosomal RNA small subunit methyltransferase B	FS43_19110	Ribosomal RNA small subunit methyltransferase B	FS44_4960	Ribosomal RNA small subunit methyltransferase B	WQG_4680	Ribosomal RNA small subunit methyltransferase B
4	FS42_17370	Methionyl-tRNA formyltransferase	FS43_19120	Methionyl-tRNA formyltransferase	FS44_4950	Methionyl-tRNA formyltransferase	WQG_4670	Methionyl-tRNA formyltransferase
4	FS42_17380	UspA protein	FS43_19130	UspA protein	FS44_4940	UspA protein	WQG_4660	UspA protein
4	FS42_17390	TRAP C4-dicarboxylate transport system permease DctM subunit	FS43_19140	TRAP C4-dicarboxylate transport system permease DctM subunit	FS44_4930	TRAP C4-dicarboxylate transport system permease DctM subunit	WQG_4650	TRAP C4-dicarboxylate transport system permease DctM subunit
4	FS42_1740	Autotransporter adhesin	FS43_1810	Autotransporter adhesin	FS44_21420	Autotransporter adhesin	WQG_20850	Autotransporter adhesin
4	FS42_17400	TRAP transporter solute receptor	FS43_19150	TRAP transporter solute receptor	FS44_4920	TRAP transporter solute receptor	WQG_4640	TRAP transporter solute receptor
4	FS42_17410	IMPACT family member	FS43_19160	IMPACT family member	FS44_4910	IMPACT family member	WQG_4630	IMPACT family member
4	FS42_17420	DNA polymerase III, epsilon subunit	FS43_19170	DNA polymerase III, epsilon subunit	FS44_4900	DNA polymerase III, epsilon subunit	WQG_4620	DNA polymerase III, epsilon subunit
4	FS42_17430	PTS system sucrose-specific EIIBC component	FS43_19180	PTS system sucrose-specific EIIBC component	FS44_4890	PTS system sucrose-specific EIIBC component	WQG_4610	PTS system sucrose-specific EIIBC component
4	FS42_17450	RNA polymerase sigma factor, ECF sub	FS43_19200	RNA polymerase sigma factor, ECF sub	FS44_4880	RNA polymerase sigma factor, ECF sub	WQG_4600	RNA polymerase sigma factor, ECF sub
4	FS42_17460	hypothetical protein	FS43_19210	hypothetical protein	FS44_4870	hypothetical protein	WQG_4590	hypothetical protein
4	FS42_17470	hypothetical protein	FS43_19220	hypothetical protein	FS44_4860	hypothetical protein	WQG_4580	hypothetical protein
4	FS42_17480	hypothetical protein	FS43_19230	hypothetical protein	FS44_4850	hypothetical protein	WQG_4570	hypothetical protein
4	FS42_17490	hypothetical protein	FS43_19240	hypothetical protein	FS44_4840	hypothetical protein	WQG_4560	hypothetical protein
4	FS42_1750	Peptidyl-prolyl cis-trans isomerase	FS43_2410	Peptidyl-prolyl cis-trans isomerase	FS44_20640	Peptidyl-prolyl cis-trans isomerase	WQG_20840	Peptidyl-prolyl cis-trans isomerase
4	FS42_17500	Anaerobic C4-dicarboxylate transporter	FS43_19250	Anaerobic C4-dicarboxylate transporter	FS44_4830	Anaerobic C4-dicarboxylate transporter	WQG_4550	Anaerobic C4-dicarboxylate transporter
4	FS42_17510	Biotin carboxylase	FS43_19260	Biotin carboxylase	FS44_4820	Biotin carboxylase	WQG_4540	Biotin carboxylase
4	FS42_17520	Biotin carboxyl carrier protein of acetyl-CoA carboxylase	FS43_19270	Biotin carboxyl carrier protein of acetyl-CoA carboxylase	FS44_4810	Biotin carboxyl carrier protein of acetyl-CoA carboxylase	WQG_4530	Biotin carboxyl carrier protein of acetyl-CoA carboxylase
4	FS42_17530	Thiamine transporter substrate binding subunit	FS43_19280	Thiamine transporter substrate binding subunit	FS44_4800	Thiamine transporter substrate binding subunit	WQG_4520	Thiamine transporter substrate binding subunit
4	FS42_17540	3-dehydroquinate dehydratase	FS43_19290	3-dehydroquinate dehydratase	FS44_4790	3-dehydroquinate dehydratase	WQG_4510	3-dehydroquinate dehydratase
4	FS42_17550	Arsenate reductase, arsenical pump modifier protein	FS43_19300	Arsenate reductase, arsenical pump modifier protein	FS44_4780	Arsenate reductase, arsenical pump modifier protein	WQG_4500	Arsenate reductase, arsenical pump modifier protein
4	FS42_17560	Inner membrane protein YtfL	FS43_19310	Inner membrane protein YtfL	FS44_4770	Inner membrane protein YtfL	WQG_4490	Inner membrane protein YtfL
4	FS42_17570	Alanine dehydrogenase	FS43_19320	Alanine dehydrogenase	FS44_4760	Alanine dehydrogenase	WQG_4480	Alanine dehydrogenase
4	FS42_17580	sodium-dependent transporter	FS43_19330	sodium-dependent transporter	FS44_4750	sodium-dependent transporter	WQG_4470	sodium-dependent transporter
4	FS42_17590	Spfh domain / band 7	FS43_19340	Spfh domain / band 7	FS44_4740	Spfh domain / band 7	WQG_4460	Spfh domain / band 7
4	FS42_17600	2,3,4,5-tetrahydropyridine-2,6-dicarboxylate N-succinyltransferase	FS43_19350	2,3,4,5-tetrahydropyridine-2,6-dicarboxylate N-succinyltransferase	FS44_4730	2,3,4,5-tetrahydropyridine-2,6-dicarboxylate N-succinyltransferase	WQG_4450	2,3,4,5-tetrahydropyridine-2,6-dicarboxylate N-succinyltransferase
4	FS42_17610	hypothetical protein	FS43_19360	hypothetical protein	FS44_4720	hypothetical protein	WQG_4440	hypothetical protein
4	FS42_17620	50S ribosomal protein L32	FS43_19370	50S ribosomal protein L32	FS44_4710	50S ribosomal protein L32	WQG_4430	50S ribosomal protein L32
4	FS42_17630	Phosphate acyltransferase	FS43_19380	Phosphate acyltransferase	FS44_4700	Phosphate acyltransferase	WQG_4420	Phosphate acyltransferase
4	FS42_17640	heavy metal-dependent transcriptional regulator	FS43_19400	heavy metal-dependent transcriptional regulator	FS44_4690	heavy metal-dependent transcriptional regulator	WQG_4410	heavy metal-dependent transcriptional regulator
4	FS42_17650	hypothetical protein	FS43_19410	hypothetical protein	FS44_4680	hypothetical protein	WQG_4400	hypothetical protein
4	FS42_17660	hypothetical protein	FS43_19420	hypothetical protein	FS44_4670	hypothetical protein	WQG_4390	hypothetical protein
4	FS42_17670	High-affinity zinc uptake system protein znuA	FS43_19430	High-affinity zinc uptake system protein znuA	FS44_4660	High-affinity zinc uptake system protein znuA	WQG_4380	High-affinity zinc uptake system protein znuA
4	FS42_17680	hypothetical protein	FS43_19440	hypothetical protein	FS44_4650	Integral membrane protein	WQG_4370	Integral membrane protein
4	FS42_17690	S6 family igA-specific metalloendopeptidase	FS43_19470	S6 family igA-specific metalloendopeptidase	FS44_4640	S6 family igA-specific metalloendopeptidase	WQG_4340	S6 family igA-specific metalloendopeptidase
4	FS42_1770	hypothetical protein	FS43_2430	hypothetical protein	FS44_20620	hypothetical protein	WQG_20810	hypothetical protein
4	FS42_17700	hypothetical protein	FS43_19490	hypothetical protein	FS44_4630	hypothetical protein	WQG_4320	hypothetical protein
4	FS42_17760	Capsule polysaccharide export protein BexD	FS43_19630	Capsule polysaccharide export protein BexD	FS44_4570	Capsule polysaccharide export protein BexD	WQG_4200	Capsule polysaccharide export protein BexD
4	FS42_17770	HexC	FS43_19640	HexC	FS44_4560	HexC	WQG_4190	HexC
4	FS42_17780	Capsule polysaccharide export inner-membrane protein BexB	FS43_19650	Capsule polysaccharide export inner-membrane protein BexB	FS44_4550	Capsule polysaccharide export inner-membrane protein BexB	WQG_4180	Capsule polysaccharide export inner-membrane protein BexB
4	FS42_17790	ATP-binding protein BexA	FS43_19660	ATP-binding protein BexA	FS44_4540	ATP-binding protein BexA	WQG_4170	ATP-binding protein BexA
4	FS42_17800	Cysteine synthase	FS43_19670	Cysteine synthase	FS44_4530	Cysteine synthase	WQG_4160	Cysteine synthase
4	FS42_17810	BASS family bile acid:sodium (Na+) symporter	FS43_19680	BASS family bile acid:sodium (Na+) symporter	FS44_4520	BASS family bile acid:sodium (Na+) symporter	WQG_4150	BASS family bile acid:sodium (Na+) symporter
4	FS42_17820	Ribonuclease	FS43_19690	Ribonuclease	FS44_4510	Ribonuclease	WQG_4140	Ribonuclease
4	FS42_17830	Protein sprT	FS43_19700	Protein sprT	FS44_4500	Protein sprT	WQG_4130	Protein sprT
4	FS42_17840	transporter component	FS43_19710	transporter component	FS44_4490	transporter component	WQG_4120	transporter component
4	FS42_17850	SirA	FS43_19720	SirA	FS44_4480	SirA	WQG_4110	SirA
4	FS42_17860	RNA polymerase-associated protein rapA	FS43_19730	RNA polymerase-associated protein rapA	FS44_4470	RNA polymerase-associated protein rapA	WQG_4100	RNA polymerase-associated protein rapA
4	FS42_17870	hypothetical protein	FS43_19740	hypothetical protein	FS44_4460	hypothetical protein	WQG_4090	hypothetical protein
4	FS42_17880	Deoxyguanosinetriphosphate triphosphohydrolase-like protein	FS43_19760	Deoxyguanosinetriphosphate triphosphohydrolase-like protein	FS44_4450	Deoxyguanosinetriphosphate triphosphohydrolase-like protein	WQG_4080	Deoxyguanosinetriphosphate triphosphohydrolase-like protein
4	FS42_17890	23S rRNA (uracil-5-)-methyltransferase RumA	FS43_19770	23S rRNA (uracil-5-)-methyltransferase RumA	FS44_4440	23S rRNA (uracil-5-)-methyltransferase RumA	WQG_4070	23S rRNA (uracil-5-)-methyltransferase RumA
4	FS42_1790	Site-specific DNA-methyltransferase	FS43_2460	Site-specific DNA-methyltransferase	FS44_20600	Site-specific DNA-methyltransferase	WQG_20790	Site-specific DNA-methyltransferase
4	FS42_17910	FxsA protein affecting phage T7 exclusion by the F plasmid	FS43_19790	FxsA protein affecting phage T7 exclusion by the F plasmid	FS44_4430	FxsA protein affecting phage T7 exclusion by the F plasmid	WQG_4050	FxsA protein affecting phage T7 exclusion by the F plasmid
4	FS42_17920	DNA polymerase III subunit chi	FS43_19800	DNA polymerase III subunit chi	FS44_4420	DNA polymerase III subunit chi	WQG_4040	DNA polymerase III subunit chi
4	FS42_17930	Acetyltransferase	FS43_19810	Acetyltransferase	FS44_4410	Acetyltransferase	WQG_4030	Acetyltransferase
4	FS42_17940	hypothetical protein	FS43_19820	hypothetical protein	FS44_4400	hypothetical protein	WQG_4020	hypothetical protein
4	FS42_17950	hypothetical protein	FS43_19830	hypothetical protein	FS44_4390	hypothetical protein	WQG_4010	hypothetical protein
4	FS42_17980	Valyl-tRNA synthetase	FS43_19860	Valyl-tRNA synthetase	FS44_4370	Valyl-tRNA synthetase	WQG_3980	Valyl-tRNA synthetase
4	FS42_17990	hypothetical protein	FS43_19870	hypothetical protein	FS44_4360	hypothetical protein	WQG_3970	hypothetical protein
4	FS42_180	reductase	FS43_190	reductase	FS44_22960	reductase	WQG_22430	reductase
4	FS42_18000	hypothetical protein	FS43_19880	hypothetical protein	FS44_4350	hypothetical protein	WQG_3960	hypothetical protein
4	FS42_18010	cell envelope integrity inner membrane protein TolA	FS43_19890	cell envelope integrity inner membrane protein TolA	FS44_4340	cell envelope integrity inner membrane protein TolA	WQG_3950	cell envelope integrity inner membrane protein TolA
4	FS42_18020	Ribonuclease E	FS43_19900	Ribonuclease E	FS44_4330	Ribonuclease E	WQG_3940	Ribonuclease E
4	FS42_18030	MacS protein	FS43_19910	MacS protein	FS44_4320	MacS protein	WQG_3930	MacS protein
4	FS42_18040	Branched-chain amino acid carrier protein	FS43_19920	Branched-chain amino acid carrier protein	FS44_4310	Branched-chain amino acid carrier protein	WQG_3920	Branched-chain amino acid carrier protein
4	FS42_18050	3-ketoacyl-(Acyl-carrier-protein) reductase	FS43_19930	3-ketoacyl-(Acyl-carrier-protein) reductase	FS44_4300	3-ketoacyl-(Acyl-carrier-protein) reductase	WQG_3910	3-ketoacyl-(Acyl-carrier-protein) reductase
4	FS42_18060	Malonyl CoA-acyl carrier protein transacylase	FS43_19940	Malonyl CoA-acyl carrier protein transacylase	FS44_4290	Malonyl CoA-acyl carrier protein transacylase	WQG_3900	Malonyl CoA-acyl carrier protein transacylase
4	FS42_18070	hypothetical protein	FS43_19950	hypothetical protein	FS44_4280	hypothetical protein	WQG_3890	hypothetical protein
4	FS42_18080	Octaprenyl-diphosphate synthase	FS43_19960	Octaprenyl-diphosphate synthase	FS44_4270	Octaprenyl-diphosphate synthase	WQG_3880	Octaprenyl-diphosphate synthase
4	FS42_18090	Hemolysin	FS43_19970	Hemolysin	FS44_4260	Hemolysin	WQG_3870	Hemolysin
4	FS42_1810	Type III restriction system methylase	FS43_2480	Type III restriction system methylase	FS44_20590	Type III restriction system methylase	WQG_20770	Type III restriction system methylase
4	FS42_18100	ABC-transport permease	FS43_19980	ABC-transport permease	FS44_4250	ABC-transport permease	WQG_3860	ABC-transport permease
4	FS42_18110	Phosphoglucosamine mutase 1	FS43_19990	Phosphoglucosamine mutase 1	FS44_4240	Phosphoglucosamine mutase 1	WQG_3850	Phosphoglucosamine mutase 1
4	FS42_18120	Ribose-5-phosphate isomerase A	FS43_20000	Ribose-5-phosphate isomerase A	FS44_4230	Ribose-5-phosphate isomerase A	WQG_3840	Ribose-5-phosphate isomerase A
4	FS42_18130	D-3-phosphoglycerate dehydrogenase	FS43_20010	D-3-phosphoglycerate dehydrogenase	FS44_4220	D-3-phosphoglycerate dehydrogenase	WQG_3830	D-3-phosphoglycerate dehydrogenase
4	FS42_18140	hypothetical protein	FS43_20020	hypothetical protein	FS44_4210	hypothetical protein	WQG_3820	hypothetical protein
4	FS42_18150	Type I secretion membrane fusion protein	FS43_20030	Type I secretion membrane fusion protein	FS44_4200	Type I secretion membrane fusion protein	WQG_3800	Type I secretion membrane fusion protein
4	FS42_18160	LapA secretion ATP-binding protein	FS43_20040	LapA secretion ATP-binding protein	FS44_4190	LapA secretion ATP-binding protein	WQG_3790	LapA secretion ATP-binding protein
4	FS42_18170	Outer membrane efflux protein	FS43_20050	Outer membrane efflux protein	FS44_4180	Outer membrane efflux protein	WQG_3780	Outer membrane efflux protein
4	FS42_18190	Transaldolase 1	FS43_20070	Transaldolase 1	FS44_4160	Transaldolase 1	WQG_3760	Transaldolase 1
4	FS42_1820	1,4-dihydroxy-2-naphthoate synthase	FS43_2490	1,4-dihydroxy-2-naphthoate synthase	FS44_20570	1,4-dihydroxy-2-naphthoate synthase	WQG_20760	1,4-dihydroxy-2-naphthoate synthase
4	FS42_18200	hypothetical protein	FS43_20080	hypothetical protein	FS44_4150	hypothetical protein	WQG_3750	hypothetical protein
4	FS42_18210	RNA polymerase sigma factor	FS43_20090	RNA polymerase sigma factor	FS44_4140	RNA polymerase sigma factor	WQG_3740	RNA polymerase sigma factor
4	FS42_18220	hypothetical protein	FS43_20100	hypothetical protein	FS44_4130	hypothetical protein	WQG_3730	hypothetical protein
4	FS42_18230	DNA primase	FS43_20110	DNA primase	FS44_4120	DNA primase	WQG_3720	DNA primase
4	FS42_18240	30S ribosomal protein S21	FS43_20120	30S ribosomal protein S21	FS44_4110	30S ribosomal protein S21	WQG_3710	30S ribosomal protein S21
4	FS42_18250	30S ribosomal protein S12	FS43_20130	30S ribosomal protein S12	FS44_4100	30S ribosomal protein S12	WQG_3700	30S ribosomal protein S12
4	FS42_18260	30S ribosomal protein S7	FS43_20140	30S ribosomal protein S7	FS44_4090	30S ribosomal protein S7	WQG_3690	30S ribosomal protein S7
4	FS42_18270	Elongation factor G	FS43_20150	Elongation factor G	FS44_4080	Elongation factor G	WQG_3680	Elongation factor G
4	FS42_18290	Peptide chain release factor 3	FS43_20170	Peptide chain release factor 3	FS44_4060	Peptide chain release factor 3	WQG_3660	Peptide chain release factor 3
4	FS42_1830	Glutamine synthetase	FS43_2500	Glutamine synthetase	FS44_20560	Glutamine synthetase	WQG_20750	Glutamine synthetase
4	FS42_18300	23S rRNA (guanosine-2'-O-)-methyltransferase RlmB	FS43_20180	23S rRNA (guanosine-2'-O-)-methyltransferase RlmB	FS44_4050	23S rRNA (guanosine-2'-O-)-methyltransferase RlmB	WQG_3650	23S rRNA (guanosine-2'-O-)-methyltransferase RlmB
4	FS42_18310	Cytochrome c peroxidase	FS43_20190	Cytochrome c peroxidase	FS44_4040	Cytochrome c peroxidase	WQG_3640	Cytochrome c peroxidase
4	FS42_18320	Transcription termination factor Rho	FS43_20210	Transcription termination factor Rho	FS44_4030	Transcription termination factor Rho	WQG_3630	Transcription termination factor Rho
4	FS42_18350	outer membrane protein	FS43_20230	outer membrane protein	FS44_4010	outer membrane protein	WQG_3610	outer membrane protein
4	FS42_18360	Heat shock protein 15	FS43_20240	Heat shock protein 15	FS44_4000	Heat shock protein 15	WQG_3600	Heat shock protein 15
4	FS42_18370	33 kDa chaperonin	FS43_20250	33 kDa chaperonin	FS44_3990	33 kDa chaperonin	WQG_3590	33 kDa chaperonin
4	FS42_1840	3-prime,5-prime cyclic nucleotide phosphodiesterase	FS43_2510	3-prime,5-prime cyclic nucleotide phosphodiesterase	FS44_20550	3-prime,5-prime cyclic nucleotide phosphodiesterase	WQG_20740	3-prime,5-prime cyclic nucleotide phosphodiesterase
4	FS42_18400	UDP-N-acetyl-D-mannosaminuronic acid transferase	FS43_20280	UDP-N-acetyl-D-mannosaminuronic acid transferase	FS44_3970	UDP-N-acetyl-D-mannosaminuronic acid transferase	WQG_3560	UDP-N-acetyl-D-mannosaminuronic acid transferase
4	FS42_18410	4-alpha-L-fucosyltransferase	FS43_20290	4-alpha-L-fucosyltransferase	FS44_3960	4-alpha-L-fucosyltransferase	WQG_3550	4-alpha-L-fucosyltransferase
4	FS42_18420	hypothetical protein	FS43_20300	hypothetical protein	FS44_3950	hypothetical protein	WQG_3540	hypothetical protein
4	FS42_18430	TDP-Fuc4NAc:lipid II Fuc4NAc transferase	FS43_20310	TDP-Fuc4NAc:lipid II Fuc4NAc transferase	FS44_3940	TDP-Fuc4NAc:lipid II Fuc4NAc transferase	WQG_3530	TDP-Fuc4NAc:lipid II Fuc4NAc transferase
4	FS42_18440	Lipopolysaccharide biosynthesis protein rffA	FS43_20320	Lipopolysaccharide biosynthesis protein rffA	FS44_3930	Lipopolysaccharide biosynthesis protein rffA	WQG_3520	Lipopolysaccharide biosynthesis protein rffA
4	FS42_18450	Hemin transport system periplasmic protein HmuT	FS43_20330	Hemin transport system periplasmic protein HmuT	FS44_3920	Hemin transport system periplasmic protein HmuT	WQG_3510	Hemin transport system periplasmic protein HmuT
4	FS42_18460	Hemin transport system permease protein hmuU	FS43_20340	Hemin transport system permease protein hmuU	FS44_3910	Hemin transport system permease protein hmuU	WQG_3500	Hemin transport system permease protein hmuU
4	FS42_18470	Hemin transport system ATP-binding protein HmuV	FS43_20350	Hemin transport system ATP-binding protein HmuV	FS44_3900	Hemin transport system ATP-binding protein HmuV	WQG_3490	Hemin transport system ATP-binding protein HmuV
4	FS42_18480	iron-regulated lipoprotein	FS43_20360	iron-regulated lipoprotein	FS44_3890	iron-regulated lipoprotein	WQG_3480	iron-regulated lipoprotein
4	FS42_18490	hypothetical protein	FS43_20370	hypothetical protein	FS44_3880	hypothetical protein	WQG_3470	hypothetical protein
4	FS42_1850	DNA mismatch repair protein mutL	FS43_2520	DNA mismatch repair protein mutL	FS44_20540	DNA mismatch repair protein mutL	WQG_20730	DNA mismatch repair protein mutL
4	FS42_18500	outer membrane colicin Js receptor	FS43_20380	outer membrane colicin Js receptor	FS44_3870	outer membrane colicin Js receptor	WQG_3460	outer membrane colicin Js receptor
4	FS42_18510	reductase	FS43_20390	reductase	FS44_3860	reductase	WQG_3450	reductase
4	FS42_18520	reductase	FS43_20400	reductase	FS44_3850	reductase	WQG_3440	reductase
4	FS42_18530	reductase	FS43_20410	reductase	FS44_3840	reductase	WQG_3430	reductase
4	FS42_18540	reductase	FS43_20420	reductase	FS44_3830	reductase	WQG_3420	reductase
4	FS42_18550	hypothetical protein	FS43_20430	hypothetical protein	FS44_3820	hypothetical protein	WQG_3410	hypothetical protein
4	FS42_18560	Integral membrane protein	FS43_20440	Integral membrane protein	FS44_3810	Integral membrane protein	WQG_3400	Integral membrane protein
4	FS42_18570	MmcQ	FS43_20450	MmcQ	FS44_3800	MmcQ	WQG_3390	MmcQ

4	FS42_18580	HAD superfamily phosphatase	FS43_20460	HAD superfamily phosphatase	FS44_3790	HAD superfamily phosphatase	WQG_3380	HAD superfamily phosphatase
4	FS42_18590	Regulatory protein AsnC	FS43_20470	Regulatory protein AsnC	FS44_3780	Regulatory protein AsnC	WQG_3370	Regulatory protein AsnC
4	FS42_1860	Multidrug-efflux transporter	FS43_2530	Multidrug-efflux transporter	FS44_20530	Multidrug-efflux transporter	WQG_20720	Multidrug-efflux transporter
4	FS42_18600	Aspartate--ammonia ligase	FS43_20480	Aspartate--ammonia ligase	FS44_3770	Aspartate--ammonia ligase	WQG_3360	Aspartate--ammonia ligase
4	FS42_18610	membrane protein	FS43_20490	membrane protein	FS44_3760	membrane protein	WQG_3350	membrane protein
4	FS42_18620	Tyrosine recombinase xerC	FS43_20500	Tyrosine recombinase xerC	FS44_3750	Tyrosine recombinase xerC	WQG_3340	Tyrosine recombinase xerC
4	FS42_18630	hypothetical protein	FS43_20510	hypothetical protein	FS44_3740	hypothetical protein	WQG_3330	hypothetical protein
4	FS42_18640	Isoprenylcysteine carboxyl methyltransferase	FS43_20520	Isoprenylcysteine carboxyl methyltransferase	FS44_3730	Isoprenylcysteine carboxyl methyltransferase	WQG_3320	Isoprenylcysteine carboxyl methyltransferase
4	FS42_18650	Glycyl-tRNA synthetase beta subunit	FS43_20530	Glycyl-tRNA synthetase beta subunit	FS44_3720	Glycyl-tRNA synthetase beta subunit	WQG_3310	Glycyl-tRNA synthetase beta subunit
4	FS42_18660	hypothetical protein	FS43_20540	hypothetical protein	FS44_3710	hypothetical protein	WQG_3300	hypothetical protein
4	FS42_18670	Glutaredoxin-related protein	FS43_20550	Glutaredoxin-related protein	FS44_3700	Glutaredoxin-related protein	WQG_3290	Glutaredoxin-related protein
4	FS42_18680	Glycyl-tRNA synthetase alpha subunit	FS43_20560	Glycyl-tRNA synthetase alpha subunit	FS44_3690	Glycyl-tRNA synthetase alpha subunit	WQG_3280	Glycyl-tRNA synthetase alpha subunit
4	FS42_18690	Biotin biosynthesis protein BioY	FS43_20570	Biotin biosynthesis protein BioY	FS44_3680	Biotin biosynthesis protein BioY	WQG_3270	Biotin biosynthesis protein BioY
4	FS42_1870	transport protein	FS43_2540	transport protein	FS44_20520	transport protein	WQG_20710	transport protein
4	FS42_18700	Biotin apo-protein ligase-related protein	FS43_20580	Biotin apo-protein ligase-related protein	FS44_3670	Biotin apo-protein ligase-related protein	WQG_3260	Biotin apo-protein ligase-related protein
4	FS42_18710	hypothetical protein	FS43_20590	hypothetical protein	FS44_3660	hypothetical protein	WQG_3250	hypothetical protein
4	FS42_18720	hypothetical protein	FS43_20600	hypothetical protein	FS44_3650	hypothetical protein	WQG_3240	hypothetical protein
4	FS42_18730	Deoxyribonuclease	FS43_20610	Deoxyribonuclease	FS44_3640	Deoxyribonuclease	WQG_3230	Deoxyribonuclease
4	FS42_18740	protein YjeA	FS43_20620	protein YjeA	FS44_3630	protein YjeA	WQG_3220	protein YjeA
4	FS42_18750	TrkG protein	FS43_20630	TrkG protein	FS44_3620	TrkG protein	WQG_3210	TrkG protein
4	FS42_18760	hypothetical protein	FS43_20640	hypothetical protein	FS44_3610	hypothetical protein	WQG_3200	hypothetical protein
4	FS42_18770	hypothetical protein	FS43_20650	hypothetical protein	FS44_3600	Peptidase propeptide and YPEB domain protein	WQG_3190	hypothetical protein
4	FS42_18780	hypothetical protein	FS43_20660	hypothetical protein	FS44_3590	hypothetical protein	WQG_3180	hypothetical protein
4	FS42_18790	hypothetical protein	FS43_20670	hypothetical protein	FS44_3580	hypothetical protein	WQG_3170	hypothetical protein
4	FS42_1880	Phosphatidylethanolamine-binding protein	FS43_2550	Phosphatidylethanolamine-binding protein	FS44_20510	Phosphatidylethanolamine-binding protein	WQG_20700	Phosphatidylethanolamine-binding protein
4	FS42_18800	transposase	FS43_20680	transposase	FS44_3570	transposase	WQG_3160	transposase
4	FS42_18810	hypothetical protein	FS43_20690	hypothetical protein	FS44_3560	hypothetical protein	WQG_3150	hypothetical protein
4	FS42_18820	Phosphoribosylformylglycinamidine synthase	FS43_20710	Phosphoribosylformylglycinamidine synthase	FS44_3550	Phosphoribosylformylglycinamidine synthase	WQG_3140	Phosphoribosylformylglycinamidine synthase
4	FS42_18830	Citrate transporter	FS43_20720	Citrate transporter	FS44_3540	Citrate transporter	WQG_3120	Citrate transporter
4	FS42_18840	Cytochrome c peroxidase	FS43_20730	Cytochrome c peroxidase	FS44_3530	Cytochrome c peroxidase	WQG_3110	Cytochrome c peroxidase
4	FS42_18850	Peptidase M15B and M15C DD-carboxypeptidase VanY/endolysin	FS43_20740	Peptidase M15B and M15C DD-carboxypeptidase VanY/endolysin	FS44_3520	Peptidase M15B and M15C DD-carboxypeptidase VanY/endolysin	WQG_3100	Peptidase M15B and M15C DD-carboxypeptidase VanY/endolysin
4	FS42_18860	hypothetical protein	FS43_20750	hypothetical protein	FS44_3510	hypothetical protein	WQG_3090	hypothetical protein
4	FS42_18880	Ribonuclease P protein component	FS43_20770	Ribonuclease P protein component	FS44_3490	Ribonuclease P protein component	WQG_3080	Ribonuclease P protein component
4	FS42_18890	50S ribosomal protein L34	FS43_20780	50S ribosomal protein L34	FS44_3480	50S ribosomal protein L34	WQG_3070	50S ribosomal protein L34
4	FS42_1890	Porphobilinogen synthase	FS43_2560	Porphobilinogen synthase	FS44_20500	Porphobilinogen synthase	WQG_20690	Porphobilinogen synthase
4	FS42_18900	Integral membrane protein	FS43_20790	Integral membrane protein	FS44_3470	Integral membrane protein	WQG_3060	Integral membrane protein
4	FS42_18910	Na(+)/H(+) antiporter nhaA	FS43_20800	Na(+)/H(+) antiporter nhaA	FS44_3460	Na(+)/H(+) antiporter nhaA	WQG_3050	Na(+)/H(+) antiporter nhaA
4	FS42_18920	Acyl carrier protein	FS43_20810	Acyl carrier protein	FS44_3450	Acyl carrier protein	WQG_3040	Acyl carrier protein
4	FS42_18940	transport system permease protein	FS43_20820	transport system permease protein	FS44_3440	transport system permease protein	WQG_3020	transport system permease protein
4	FS42_18950	hypothetical protein	FS43_20830	hypothetical protein	FS44_3430	hypothetical protein	WQG_3010	hypothetical protein
4	FS42_18960	Sugar diacid utilization regulator	FS43_20840	Sugar diacid utilization regulator	FS44_3420	Sugar diacid utilization regulator	WQG_3000	Sugar diacid utilization regulator
4	FS42_18970	GntP	FS43_20850	GntP	FS44_3410	GntP	WQG_2990	GntP
4	FS42_18980	Glycerate kinase	FS43_20860	Glycerate kinase	FS44_3400	Glycerate kinase	WQG_2980	Glycerate kinase
4	FS42_18990	Prolyl-tRNA synthetase	FS43_20870	Prolyl-tRNA synthetase	FS44_3390	Prolyl-tRNA synthetase	WQG_2970	Prolyl-tRNA synthetase
4	FS42_190	Inner-membrane translocator	FS43_200	Inner-membrane translocator	FS44_22950	Inner-membrane translocator	WQG_22420	Inner-membrane translocator
4	FS42_1900	ADP-heptose--LPS heptosyltransferase 2	FS43_2570	ADP-heptose--LPS heptosyltransferase 2	FS44_20490	ADP-heptose--LPS heptosyltransferase 2	WQG_20680	ADP-heptose--LPS heptosyltransferase 2
4	FS42_19010	Ser/Thr protein phosphatase	FS43_20890	Ser/Thr protein phosphatase	FS44_3360	Ser/Thr protein phosphatase	WQG_2950	Ser/Thr protein phosphatase
4	FS42_19020	Protein mioC	FS43_20900	Protein mioC	FS44_3350	Protein mioC	WQG_2940	Protein mioC
4	FS42_19030	hypothetical protein	FS43_20910	hypothetical protein	FS44_3340	hypothetical protein	WQG_2930	hypothetical protein
4	FS42_19040	RNA polymerase sigma-32 factor	FS43_20920	RNA polymerase sigma-32 factor	FS44_3330	RNA polymerase sigma-32 factor	WQG_2920	RNA polymerase sigma-32 factor
4	FS42_19050	Thiol:disulfide interchange protein DsbA	FS43_20930	Thiol:disulfide interchange protein DsbA	FS44_3320	Thiol:disulfide interchange protein DsbA	WQG_2910	Thiol:disulfide interchange protein DsbA
4	FS42_19060	YihD like protein	FS43_20940	YihD like protein	FS44_3310	YihD like protein	WQG_2900	YihD like protein
4	FS42_19070	Molybdopterin biosynthesis MoeA protein	FS43_20950	Molybdopterin biosynthesis MoeA protein	FS44_3300	Molybdopterin biosynthesis MoeA protein	WQG_2890	Molybdopterin biosynthesis MoeA protein
4	FS42_19080	Mannitol operon repressor	FS43_20960	Mannitol operon repressor	FS44_3290	Mannitol operon repressor	WQG_2880	Mannitol operon repressor
4	FS42_19090	Mannitol-1-phosphate 5-dehydrogenase	FS43_20970	Mannitol-1-phosphate 5-dehydrogenase	FS44_3280	Mannitol-1-phosphate 5-dehydrogenase	WQG_2870	Mannitol-1-phosphate 5-dehydrogenase
4	FS42_1910	Opacity associated protein B	FS43_2580	Opacity associated protein B	FS44_20480	Opacity associated protein B	WQG_20670	Opacity associated protein B
4	FS42_19100	PTS system mannitol-specific EIICBA component	FS43_20980	PTS system mannitol-specific EIICBA component	FS44_3270	PTS system mannitol-specific EIICBA component	WQG_2860	PTS system mannitol-specific EIICBA component
4	FS42_19110	YifE like protein	FS43_20990	YifE like protein	FS44_3260	YifE like protein	WQG_2850	YifE like protein
4	FS42_19120	tRNA (uracil-5-)-methyltransferase	FS43_21000	tRNA (uracil-5-)-methyltransferase	FS44_3250	tRNA (uracil-5-)-methyltransferase	WQG_2840	tRNA (uracil-5-)-methyltransferase
4	FS42_19130	Gamma-glutamyl phosphate reductase	FS43_21010	Gamma-glutamyl phosphate reductase	FS44_2320	Gamma-glutamyl phosphate reductase	WQG_2830	Gamma-glutamyl phosphate reductase
4	FS42_19140	hypothetical protein	FS43_21020	hypothetical protein	FS44_2310	hypothetical protein	WQG_2820	hypothetical protein
4	FS42_19150	D-tyrosyl-tRNA(Tyr) deacylase	FS43_21030	D-tyrosyl-tRNA(Tyr) deacylase	FS44_2300	D-tyrosyl-tRNA(Tyr) deacylase	WQG_2810	D-tyrosyl-tRNA(Tyr) deacylase
4	FS42_19160	tRNA(Ile)-lysidine synthase	FS43_21040	tRNA(Ile)-lysidine synthase	FS44_2290	tRNA(Ile)-lysidine synthase	WQG_2800	tRNA(Ile)-lysidine synthase
4	FS42_19170	NemA protein	FS43_21050	NemA protein	FS44_2270	NemA protein	WQG_2790	NemA protein
4	FS42_19180	Regulatory protein ArsR	FS43_21060	Regulatory protein ArsR	FS44_2260	Regulatory protein ArsR	WQG_2780	Regulatory protein ArsR
4	FS42_19190	30S ribosomal protein S20	FS43_21070	30S ribosomal protein S20	FS44_2250	30S ribosomal protein S20	WQG_2770	30S ribosomal protein S20
4	FS42_1920	Opacity associated protein A	FS43_2590	Opacity associated protein A	FS44_20470	Opacity associated protein A	WQG_20660	Opacity associated protein A
4	FS42_19200	Virulence factor-like MviN	FS43_21080	Virulence factor-like MviN	FS44_2240	Virulence factor-like MviN	WQG_2760	Virulence factor-like MviN
4	FS42_19210	Formate/nitrite transporter	FS43_21090	Formate/nitrite transporter	FS44_2230	Formate/nitrite transporter	WQG_2750	Formate/nitrite transporter
4	FS42_19220	hypothetical protein	FS43_21100	hypothetical protein	FS44_2220	hypothetical protein	WQG_2740	hypothetical protein
4	FS42_19230	Elongation factor Tu	FS43_21110	Elongation factor Tu	FS44_2210	Elongation factor Tu	WQG_2730	Elongation factor Tu
4	FS42_19250	Pantothenate kinase	FS43_21130	Pantothenate kinase	FS44_2200	Pantothenate kinase	WQG_2710	Pantothenate kinase
4	FS42_19260	tRNA (adenine-N(6)-)-methyltransferase	FS43_21140	tRNA (adenine-N(6)-)-methyltransferase	FS44_2190	tRNA (adenine-N(6)-)-methyltransferase	WQG_2700	tRNA (adenine-N(6)-)-methyltransferase
4	FS42_19270	DNA polymerase III subunit delta	FS43_21150	DNA polymerase III subunit delta	FS44_2180	DNA polymerase III subunit delta	WQG_2690	DNA polymerase III subunit delta
4	FS42_19280	hypothetical protein	FS43_21160	hypothetical protein	FS44_2170	hypothetical protein	WQG_2680	hypothetical protein
4	FS42_19290	Thymidylate kinase	FS43_21170	Thymidylate kinase	FS44_2160	Thymidylate kinase	WQG_2670	Thymidylate kinase
4	FS42_1930	DNA uptake protein	FS43_2600	DNA uptake protein	FS44_20460	DNA uptake protein	WQG_20650	DNA uptake protein
4	FS42_19300	UPF0755 protein	FS43_21180	UPF0755 protein	FS44_2150	UPF0755 protein	WQG_2660	UPF0755 protein
4	FS42_19310	Serotype-specific antigen 1	FS43_21190	Serotype-specific antigen 1	FS44_2140	Serotype-specific antigen 1	WQG_2650	Serotype-specific antigen 1
4	FS42_19320	Formamidopyrimidine-DNA glycosylase	FS43_21200	Formamidopyrimidine-DNA glycosylase	FS44_2130	Formamidopyrimidine-DNA glycosylase	WQG_2640	Formamidopyrimidine-DNA glycosylase
4	FS42_19330	Capsule polysaccharide modification protein HcsB	FS43_21210	Capsule polysaccharide modification protein HcsB	FS44_2120	Capsule polysaccharide modification protein HcsB	WQG_2630	Capsule polysaccharide modification protein HcsB
4	FS42_19340	Capsule polysaccharide modification protein LipA	FS43_21220	Capsule polysaccharide modification protein LipA	FS44_2110	Capsule polysaccharide modification protein LipA	WQG_2620	Capsule polysaccharide modification protein LipA
4	FS42_19350	Ribonuclease PH	FS43_21230	Ribonuclease PH	FS44_2090	Ribonuclease PH	WQG_2610	Ribonuclease PH
4	FS42_19360	UPF0701 protein	FS43_21240	UPF0701 protein	FS44_2080	UPF0701 protein	WQG_2600	UPF0701 protein
4	FS42_19370	transposase	FS43_21260	transposase	FS44_2280	transposase	WQG_2590	transposase
4	FS42_19380	Biotin operon repressor	FS43_21270	Biotin operon repressor	FS44_2070	Biotin operon repressor	WQG_2580	Biotin operon repressor
4	FS42_19390	UDP-N-acetylenolpyruvoylglucosamine reductase	FS43_21280	UDP-N-acetylenolpyruvoylglucosamine reductase	FS44_2060	UDP-N-acetylenolpyruvoylglucosamine reductase	WQG_2570	UDP-N-acetylenolpyruvoylglucosamine reductase
4	FS42_1940	Phosphatidylserine decarboxylase proenzyme	FS43_2610	Phosphatidylserine decarboxylase proenzyme	FS44_20450	Phosphatidylserine decarboxylase proenzyme	WQG_20640	Phosphatidylserine decarboxylase proenzyme
4	FS42_19400	Hydrogen peroxide-inducible gene activator protein	FS43_21290	Hydrogen peroxide-inducible gene activator protein	FS44_2050	Hydrogen peroxide-inducible gene activator protein	WQG_2560	Hydrogen peroxide-inducible gene activator
4	FS42_19410	HTH-type transcriptional repressor fabR	FS43_21300	HTH-type transcriptional repressor fabR	FS44_2040	HTH-type transcriptional repressor fabR	WQG_2550	HTH-type transcriptional repressor fabR
4	FS42_19420	Thioredoxin-like protein	FS43_21310	Thioredoxin-like protein	FS44_2030	Thioredoxin-like protein	WQG_2540	Thioredoxin-like protein
4	FS42_19430	Lipoate-protein ligase	FS43_21320	Lipoate-protein ligase	FS44_2020	Lipoate-protein ligase	WQG_2530	Lipoate-protein ligase
4	FS42_1950	ADP-ribose pyrophosphatase	FS43_2620	ADP-ribose pyrophosphatase	FS44_20440	ADP-ribose pyrophosphatase	WQG_20630	ADP-ribose pyrophosphatase
4	FS42_19520	reductase	FS43_21420	reductase	FS44_2010	reductase	WQG_2440	reductase
4	FS42_19530	D-isomer specific 2-hydroxyacid dehydrogenase	FS43_21430	D-isomer specific 2-hydroxyacid dehydrogenase	FS44_1990	D-isomer specific 2-hydroxyacid dehydrogenase	WQG_2430	D-isomer specific 2-hydroxyacid dehydrogenase
4	FS42_19540	NfnB protein	FS43_21440	NfnB protein	FS44_1980	NfnB protein	WQG_2420	NfnB protein
4	FS42_19550	NADPH dehydrogenase	FS43_21450	NADPH dehydrogenase	FS44_1970	NADPH dehydrogenase	WQG_2410	NADPH dehydrogenase
4	FS42_19560	LysR family transcriptional regulator	FS43_21460	LysR family transcriptional regulator	FS44_1960	LysR family transcriptional regulator	WQG_2400	LysR family transcriptional regulator
4	FS42_19570	DMT superfamily drug/metabolite transporter	FS43_21470	DMT superfamily drug/metabolite transporter	FS44_1950	DMT superfamily drug/metabolite transporter	WQG_2390	DMT superfamily drug/metabolite transporter
4	FS42_19580	DNA transformation protein	FS43_21480	DNA transformation protein	FS44_1940	DNA transformation protein	WQG_2380	DNA transformation protein
4	FS42_1960	PsiE-like protein	FS43_2630	PsiE-like protein	FS44_20430	PsiE-like protein	WQG_20620	PsiE-like protein
4	FS42_19620	Anaerobic C4-dicarboxylate transporter DcuA	FS43_21520	Anaerobic C4-dicarboxylate transporter DcuA	FS44_1910	Anaerobic C4-dicarboxylate transporter DcuA	WQG_2340	Anaerobic C4-dicarboxylate transporter DcuA
4	FS42_19630	aspartate racemase	FS43_21530	aspartate racemase	FS44_1900	aspartate racemase	WQG_2330	aspartate racemase
4	FS42_19640	HTH-type transcriptional regulator QseD	FS43_21540	HTH-type transcriptional regulator QseD	FS44_1890	HTH-type transcriptional regulator QseD	WQG_2320	HTH-type transcriptional regulator QseD
4	FS42_19650	Protease III	FS43_21550	Protease III	FS44_1880	Protease III	WQG_2310	Protease III
4	FS42_19660	Exodeoxyribonuclease V, gamma subunit	FS43_21560	Exodeoxyribonuclease V, gamma subunit	FS44_1870	Exodeoxyribonuclease V, gamma subunit	WQG_2300	Exodeoxyribonuclease V, gamma subunit
4	FS42_19670	hypothetical protein	FS43_21570	hypothetical protein	FS44_1860	hypothetical protein	WQG_2290	hypothetical protein
4	FS42_19680	hypothetical protein	FS43_21580	hypothetical protein	FS44_1850	hypothetical protein	WQG_2280	hypothetical protein
4	FS42_19690	hypothetical protein	FS43_21590	hypothetical protein	FS44_1840	hypothetical protein	WQG_2270	hypothetical protein
4	FS42_1970	Phosphopantetheinyl transferase	FS43_2640	Phosphopantetheinyl transferase	FS44_20420	Phosphopantetheinyl transferase	WQG_20610	Phosphopantetheinyl transferase
4	FS42_19700	Type II secretory pathway, pseudopilin PulG	FS43_21600	Type II secretory pathway, pseudopilin PulG	FS44_1830	Type II secretory pathway, pseudopilin PulG	WQG_2260	Type II secretory pathway, pseudopilin PulG
4	FS42_19710	3-hydroxydecanoyl-[acyl-carrier-protein] dehydratase	FS43_21610	3-hydroxydecanoyl-[acyl-carrier-protein] dehydratase	FS44_1820	3-hydroxydecanoyl-[acyl-carrier-protein] dehydratase	WQG_2250	3-hydroxydecanoyl-[acyl-carrier-protein] dehydratase
4	FS42_19720	Lon protease	FS43_21620	Lon protease	FS44_1810	Lon protease	WQG_2240	Lon protease
4	FS42_19730	hypothetical protein	FS43_21630	hypothetical protein	FS44_1800	hypothetical protein	WQG_2230	hypothetical protein
4	FS42_19740	Outer membrane protein P5	FS43_21640	Outer membrane protein P5	FS44_1790	Outer membrane protein P5	WQG_2220	Outer membrane protein P5
4	FS42_19750	Outer membrane protein P5	FS43_21650	Outer membrane protein P5	FS44_1780	Outer membrane protein P5	WQG_2210	Outer membrane protein P5
4	FS42_19760	FAD/FMN dehydrogenase	FS43_21660	FAD/FMN dehydrogenase	FS44_1770	FAD/FMN dehydrogenase	WQG_2200	FAD/FMN dehydrogenase
4	FS42_19770	membrane protein	FS43_21670	membrane protein	FS44_1760	membrane protein	WQG_2190	membrane protein
4	FS42_19780	HflX	FS43_21680	HflX	FS44_1750	HflX	WQG_2180	HflX
4	FS42_19790	Protein hfq	FS43_21690	Protein hfq	FS44_1740	Protein hfq	WQG_2170	Protein hfq
4	FS42_1980	DNA-3-methyladenine glycosylase I	FS43_2650	DNA-3-methyladenine glycosylase I	FS44_20410	DNA-3-methyladenine glycosylase I	WQG_20600	DNA-3-methyladenine glycosylase I
4	FS42_19800	tRNA dimethylallyltransferase	FS43_21700	tRNA dimethylallyltransferase	FS44_1730	tRNA dimethylallyltransferase	WQG_2160	tRNA dimethylallyltransferase
4	FS42_19810	Periplasmic protein TonB	FS43_21710	Periplasmic protein TonB	FS44_1720	Periplasmic protein TonB	WQG_2150	Periplasmic protein TonB
4	FS42_19820	hypothetical protein	FS43_21720	hypothetical protein	FS44_1710	Lipoprotein 5 domain containing protein	WQG_2140	hypothetical protein
4	FS42_19840	Hemoglobin receptor	FS43_21740	Hemoglobin receptor	FS44_1680	Hemoglobin receptor	WQG_2120	Hemoglobin receptor
4	FS42_19850	Heme utilization protein HuvX	FS43_21750	Heme utilization protein HuvX	FS44_1670	Heme utilization protein HuvX	WQG_2110	Heme utilization protein HuvX
4	FS42_19860	hypothetical protein	FS43_21760	hypothetical protein	FS44_1660	hypothetical protein	WQG_2100	hypothetical protein
4	FS42_19870	2-octaprenyl-3-methyl-6-methoxy-1,4-benzoquinol hydroxylase	FS43_21770	2-octaprenyl-3-methyl-6-methoxy-1,4-benzoquinol hydroxylase	FS44_1650	2-octaprenyl-3-methyl-6-methoxy-1,4-benzoquinol hydroxylase	WQG_2090	2-octaprenyl-3-methyl-6-methoxy-1,4-benzoquinol hydroxylase
4	FS42_19880	Competence protein ComM	FS43_21780	Competence protein ComM	FS44_1640	Competence protein ComM	WQG_2080	Competence protein ComM
4	FS42_19890	Chaperone protein DnaK	FS43_21790	Chaperone protein DnaK	FS44_1620	Chaperone protein DnaK	WQG_2070	Chaperone protein DnaK
4	FS42_1990	hypothetical protein	FS43_2660	hypothetical protein	FS44_20400	hypothetical protein	WQG_20590	hypothetical protein
4	FS42_19910	Chaperone protein DnaJ	FS43_21820	Chaperone protein DnaJ	FS44_1590	Chaperone protein DnaJ	WQG_2040	Chaperone protein DnaJ
4	FS42_19920	Aerobic respiration control protein ArcA	FS43_21830	Aerobic respiration control protein ArcA	FS44_1580	Aerobic respiration control protein ArcA	WQG_2030	Aerobic respiration control protein ArcA
4	FS42_19930	Cytidine deaminase	FS43_21840	Cytidine deaminase	FS44_1570	Cytidine deaminase	WQG_2020	Cytidine deaminase
4	FS42_19940	Dihydroneopterin aldolase	FS43_21850	Dihydroneopterin aldolase	FS44_1560	Dihydroneopterin aldolase	WQG_2010	Dihydroneopterin aldolase
4	FS42_19950	Glycerol-3-phosphate acyltransferase	FS43_21860	Glycerol-3-phosphate acyltransferase	FS44_1550	Glycerol-3-phosphate acyltransferase	WQG_2000	Glycerol-3-phosphate acyltransferase
4	FS42_19960	Abortive infection protein	FS43_21870	Abortive infection protein	FS44_1540	Abortive infection protein	WQG_1990	Abortive infection protein
4	FS42_19970	L-2,4-diaminobutyrate decarboxylase	FS43_21880	L-2,4-diaminobutyrate decarboxylase	FS44_1530	L-2,4-diaminobutyrate decarboxylase	WQG_1970	L-2,4-diaminobutyrate decarboxylase
4	FS42_19980	adhesin	FS43_21890	adhesin	FS44_1520	adhesin	WQG_1960	adhesin
4	FS42_19990	Diaminobutyrate--2-oxoglutarate aminotransferase	FS43_21900	Diaminobutyrate--2-oxoglutarate aminotransferase	FS44_1510	Diaminobutyrate--2-oxoglutarate aminotransferase	WQG_1950	Diaminobutyrate--2-oxoglutarate aminotransferase
4	FS42_20	Ribosomal RNA large subunit methyltransferase H	FS43_30	Ribosomal RNA large subunit methyltransferase H	FS44_10	Ribosomal RNA large subunit methyltransferase H	WQG_22590	Ribosomal RNA large subunit methyltransferase H
4	FS42_200	ABC transporter protein	FS43_210	ABC transporter protein	FS44_22940	ABC transporter protein	WQG_22410	ABC transporter protein
4	FS42_2000	Outer membrane antigenic lipoprotein B	FS43_2670	Outer membrane antigenic lipoprotein B	FS44_20390	Outer membrane antigenic lipoprotein B	WQG_20580	Outer membrane antigenic lipoprotein B
4	FS42_20000	hypothetical protein	FS43_21910	hypothetical protein	FS44_1500	hypothetical protein	WQG_1940	hypothetical protein
4	FS42_20010	Esterase YbdB	FS43_21920	Esterase YbdB	FS44_1490	Esterase YbdB	WQG_1930	Esterase YbdB
4	FS42_20020	UPF0350 protein	FS43_21930	UPF0350 protein	FS44_1480	UPF0350 protein	WQG_1920	UPF0350 protein
4	FS42_20030	4-hydroxy-3-methylbut-2-enyl diphosphate reductase	FS43_21940	4-hydroxy-3-methylbut-2-enyl diphosphate reductase	FS44_1470	4-hydroxy-3-methylbut-2-enyl diphosphate reductase	WQG_1910	4-hydroxy-3-methylbut-2-enyl diphosphate reductase
4	FS42_20040	Lipoprotein signal peptidase	FS43_21950	Lipoprotein signal peptidase	FS44_1460	Lipoprotein signal peptidase	WQG_1900	Lipoprotein signal peptidase

4	**FS42_20060**	Nitrogen regulatory protein P-II	FS43_21960	Nitrogen regulatory protein P-II	FS44_1440	Nitrogen regulatory protein P-II	WQG_1890	Nitrogen regulatory protein P-II
4	**FS42_20070**	hypothetical protein	FS43_21970	hypothetical protein	FS44_1430	hypothetical protein	WQG_1880	hypothetical protein
4	**FS42_20080**	Molybdopterin adenylyltransferase	FS43_21980	Molybdopterin adenylyltransferase	FS44_1420	Molybdopterin adenylyltransferase	WQG_1870	Molybdopterin adenylyltransferase
4	**FS42_20090**	UDP-N-acetylenolpyruvoylglucosamine reductase	FS43_21990	UDP-N-acetylenolpyruvoylglucosamine reductase	FS44_1410	UDP-N-acetylenolpyruvoylglucosamine reductase	WQG_1860	UDP-N-acetylenolpyruvoylglucosamine reductase
4	**FS42_20100**	Glycosyltransferase involved in cell wall biogenesis	FS43_22000	Glycosyltransferase involved in cell wall biogenesis	FS44_1400	Glycosyltransferase involved in cell wall biogenesis	WQG_1850	Glycosyltransferase involved in cell wall biogenesis
4	**FS42_20110**	UDP-2,3-diacylglucosamine hydrolase	FS43_22010	UDP-2,3-diacylglucosamine hydrolase	FS44_1390	UDP-2,3-diacylglucosamine hydrolase	WQG_1840	UDP-2,3-diacylglucosamine hydrolase
4	**FS42_20120**	lipoprotein	FS43_22020	lipoprotein	FS44_1380	lipoprotein	WQG_1830	lipoprotein
4	**FS42_20130**	Lipoprotein	FS43_22030	Lipoprotein	FS44_1370	Lipoprotein	WQG_1820	Lipoprotein
4	**FS42_20140**	Tryptophanyl-tRNA synthetase	FS43_22040	Tryptophanyl-tRNA synthetase	FS44_1360	Tryptophanyl-tRNA synthetase	WQG_1810	Tryptophanyl-tRNA synthetase
4	**FS42_20150**	hypothetical protein	FS43_22050	hypothetical protein	FS44_1340	hypothetical protein	WQG_1800	hypothetical protein
4	**FS42_20170**	Carbohydrate kinase, thermoresistant glucokinase	FS43_22070	Carbohydrate kinase, thermoresistant glucokinase	FS44_1330	Carbohydrate kinase, thermoresistant glucokinase	WQG_1790	Carbohydrate kinase, thermoresistant glucokinase
4	**FS42_20180**	Low-affinity gluconate/H+ symporter GntU	FS43_22080	Low-affinity gluconate/H+ symporter GntU	FS44_1320	Low-affinity gluconate/H+ symporter GntU	WQG_1780	Low-affinity gluconate/H+ symporter GntU
4	**FS42_20190**	Cof protein	FS43_22090	Cof protein	FS44_1310	Cof protein	WQG_1770	Cof protein
4	**FS42_2020**	Lipoprotein	FS43_2680	Lipoprotein	FS44_20380	Lipoprotein	WQG_20570	Lipoprotein
4	**FS42_20200**	hypothetical protein	FS43_22100	hypothetical protein	FS44_1300	hypothetical protein	WQG_1760	hypothetical protein
4	**FS42_20210**	Mannose permease iid component	FS43_22110	Mannose permease iid component	FS44_1290	Mannose permease iid component	WQG_1750	Mannose permease iid component
4	**FS42_20220**	Mannose permease IIC component	FS43_22120	Mannose permease IIC component	FS44_1280	Mannose permease IIC component	WQG_1740	Mannose permease IIC component
4	**FS42_20230**	Pts system mannose-specific eiiab component	FS43_22130	Pts system mannose-specific eiiab component	FS44_1270	Pts system mannose-specific eiiab component	WQG_1730	Pts system mannose-specific eiiab component
4	**FS42_20240**	3-keto-L-gulonate-6-phosphate decarboxylase	FS43_22140	3-keto-L-gulonate-6-phosphate decarboxylase	FS44_1260	3-keto-L-gulonate-6-phosphate decarboxylase	WQG_1720	3-keto-L-gulonate-6-phosphate decarboxylase
4	**FS42_20250**	PTS system, IIA component	FS43_22150	PTS system, IIA component	FS44_1250	PTS system, IIA component	WQG_1710	PTS system, IIA component
4	**FS42_20260**	Ascorbate-specific permease IIC component ulaA	FS43_22160	Ascorbate-specific permease IIC component ulaA	FS44_1240	Ascorbate-specific permease IIC component ulaA	WQG_1700	Ascorbate-specific permease IIC component ulaA
4	**FS42_20270**	L-ascorbate-6-phosphate lactonase ulaG	FS43_22170	L-ascorbate-6-phosphate lactonase ulaG	FS44_1230	L-ascorbate-6-phosphate lactonase ulaG	WQG_1690	L-ascorbate-6-phosphate lactonase ulaG
4	**FS42_20280**	HTH-type transcriptional regulator UlaR	FS43_22180	HTH-type transcriptional regulator UlaR	FS44_1220	HTH-type transcriptional regulator UlaR	WQG_1680	HTH-type transcriptional regulator UlaR
4	**FS42_20290**	SgaU protein	FS43_22190	SgaU protein	FS44_1210	SgaU protein	WQG_1670	SgaU protein
4	**FS42_2030**	hypothetical protein	FS43_2690	hypothetical protein	FS44_20370	hypothetical protein	WQG_20560	hypothetical protein
4	**FS42_20300**	L-ribulose-5-phosphate 4-epimerase UlaF	FS43_22200	L-ribulose-5-phosphate 4-epimerase UlaF	FS44_1200	L-ribulose-5-phosphate 4-epimerase UlaF	WQG_1660	L-ribulose-5-phosphate 4-epimerase UlaF
4	**FS42_20310**	Serine protease	FS43_22210	Serine protease	FS44_1190	Serine protease	WQG_1650	Serine protease
4	**FS42_20320**	hypothetical protein	FS43_22220	hypothetical protein	FS44_1180	hypothetical protein	WQG_1640	hypothetical protein
4	**FS42_20330**	ADP-L-glycero-D-manno-heptose-6-epimerase	FS43_22230	ADP-L-glycero-D-manno-heptose-6-epimerase	FS44_1170	ADP-L-glycero-D-manno-heptose-6-epimerase	WQG_1630	ADP-L-glycero-D-manno-heptose-6-epimerase
4	**FS42_20340**	Diaminopimelate epimerase	FS43_22240	Diaminopimelate epimerase	FS44_1160	Diaminopimelate epimerase	WQG_1620	Diaminopimelate epimerase
4	**FS42_20350**	Anaerobic C4-dicarboxylate transporter DcuA	FS43_22250	Anaerobic C4-dicarboxylate transporter DcuA	FS44_1150	Anaerobic C4-dicarboxylate transporter DcuA	WQG_1610	Anaerobic C4-dicarboxylate transporter DcuA
4	**FS42_20360**	outer membrane protein	FS43_22260	outer membrane protein	FS44_1130	outer membrane protein	WQG_1600	outer membrane protein
4	**FS42_20370**	DNA mismatch repair protein mutS	FS43_22270	DNA mismatch repair protein mutS	FS44_1120	DNA mismatch repair protein mutS	WQG_1590	DNA mismatch repair protein mutS
4	**FS42_20380**	Selenocysteine-specific elongation factor	FS43_22280	Selenocysteine-specific elongation factor	FS44_1110	Selenocysteine-specific elongation factor	WQG_1580	Selenocysteine-specific elongation factor
4	**FS42_20390**	Protein involved in oxidation of intracellular sulfur	FS43_22290	Protein involved in oxidation of intracellular sulfur	FS44_1100	Protein involved in oxidation of intracellular sulfur	WQG_1570	Protein involved in oxidation of intracellular sulfur
4	**FS42_2040**	5'-nucleotidase surE	FS43_2700	5'-nucleotidase surE	FS44_20360	5'-nucleotidase surE	WQG_20550	5'-nucleotidase surE
4	**FS42_20400**	sulfur oxidation protein DsrF	FS43_22300	sulfur oxidation protein DsrF	FS44_1090	sulfur oxidation protein DsrF	WQG_1560	sulfur oxidation protein DsrF
4	**FS42_20410**	Sulfurtransferase TusD-like protein	FS43_22310	Sulfurtransferase TusD-like protein	FS44_1080	Sulfurtransferase TusD-like protein	WQG_1550	Sulfurtransferase TusD-like protein
4	**FS42_20420**	hypothetical protein	FS43_22320	hypothetical protein	FS44_1070	hypothetical protein	WQG_1540	hypothetical protein
4	**FS42_20430**	Peptidyl-prolyl cis-trans isomerase	FS43_22330	Peptidyl-prolyl cis-trans isomerase	FS44_1060	Peptidyl-prolyl cis-trans isomerase	WQG_1530	Peptidyl-prolyl cis-trans isomerase
4	**FS42_20440**	Protein slyX	FS43_22340	Protein slyX	FS44_1050	Protein slyX	WQG_1520	Protein slyX
4	**FS42_20450**	YgiW like protein	FS43_22350	YgiW like protein	FS44_1040	YgiW like protein	WQG_1510	YgiW like protein
4	**FS42_20460**	Glutathione biosynthesis bifunctional protein gshAB	FS43_22360	Glutathione biosynthesis bifunctional protein gshAB	FS44_1030	Glutathione biosynthesis bifunctional protein gshAB	WQG_1500	Glutathione biosynthesis bifunctional protein gshAB
4	**FS42_20470**	hypothetical protein	FS43_22370	hypothetical protein	FS44_1020	hypothetical protein	WQG_1490	hypothetical protein
4	**FS42_20480**	Transcriptional regulator, MarR	FS43_22380	Transcriptional regulator, MarR	FS44_1010	Transcriptional regulator, MarR	WQG_1480	Transcriptional regulator, MarR
4	**FS42_20490**	hypothetical protein	FS43_22390	hypothetical protein	FS44_1000	hypothetical protein	WQG_1470	hypothetical protein
4	**FS42_2050**	tRNA pseudouridine synthase D	FS43_2710	tRNA pseudouridine synthase D	FS44_20350	tRNA pseudouridine synthase D	WQG_20540	tRNA pseudouridine synthase D
4	**FS42_20500**	LysR protein	FS43_22400	LysR protein	FS44_990	LysR protein	WQG_1460	LysR protein
4	**FS42_20520**	Transcriptional regulator, HxlR	FS43_22410	Transcriptional regulator, HxlR	FS44_980	Transcriptional regulator, HxlR	WQG_1440	Transcriptional regulator, HxlR
4	**FS42_20530**	NADPH dehydrogenase	FS43_22420	NADPH dehydrogenase	FS44_970	NADPH dehydrogenase	WQG_1430	NADPH dehydrogenase
4	**FS42_20580**	LysR substrate binding domain protein	FS43_22470	LysR substrate binding domain protein	FS44_950	LysR substrate binding domain protein	WQG_1380	LysR substrate binding domain protein
4	**FS42_20590**	Hydrolase	FS43_22480	Hydrolase	FS44_940	Hydrolase	WQG_1370	Hydrolase
4	**FS42_2060**	Triosephosphate isomerase	FS43_2720	Triosephosphate isomerase	FS44_20340	Triosephosphate isomerase	WQG_20530	Triosephosphate isomerase
4	**FS42_20600**	hypothetical protein	FS43_22490	hypothetical protein	FS44_930	hypothetical protein	WQG_1360	hypothetical protein
4	**FS42_20610**	LysR protein	FS43_22500	LysR protein	FS44_920	LysR protein	WQG_1350	LysR protein
4	**FS42_20630**	Glutathione-regulated potassium-efflux system ancillary protein KefF	FS43_22520	Glutathione-regulated potassium-efflux system ancillary protein KefF	FS44_910	MdaB protein	WQG_1330	Glutathione-regulated potassium-efflux system ancillary protein KefF
4	**FS42_20640**	YCII-related protein	FS43_22530	YCII-related protein	FS44_900	YCII-related protein	WQG_1320	YCII-related protein
4	**FS42_20650**	LysR family transcriptional regulator	FS43_22540	LysR family transcriptional regulator	FS44_890	LysR family transcriptional regulator	WQG_1310	LysR family transcriptional regulator
4	**FS42_20660**	Pyruvate kinase	FS43_22560	Pyruvate kinase	FS44_870	Pyruvate kinase	WQG_1300	Pyruvate kinase
4	**FS42_20670**	Tautomerase enzyme	FS43_22570	Tautomerase enzyme	FS44_860	Tautomerase enzyme	WQG_1290	Tautomerase enzyme
4	**FS42_20680**	DSBA oxidoreductase	FS43_22580	DSBA oxidoreductase	FS44_850	DSBA oxidoreductase	WQG_1280	DSBA oxidoreductase
4	**FS42_20690**	MFS family major facilitator transporter, multidrug:cation symporter	FS43_22590	MFS family major facilitator transporter, multidrug:cation symporter	FS44_840	MFS family major facilitator transporter, multidrug:cation symporter	WQG_1270	MFS family major facilitator transporter, multidrug:cation symporter
4	**FS42_2070**	hypothetical protein	FS43_2730	hypothetical protein	FS44_20330	hypothetical protein	WQG_20520	hypothetical protein
4	**FS42_20700**	Bifunctional phosphoribosylaminoimidazolecarboxamide formyltransferase/IMP cyclohydrolase	FS43_22600	Bifunctional phosphoribosylaminoimidazolecarboxamide formyltransferase/IMP cyclohydrolase	FS44_830	Bifunctional phosphoribosylaminoimidazolecarboxamide formyltransferase/IMP cyclohydrolase	WQG_1260	Bifunctional phosphoribosylaminoimidazolecarboxamide formyltransferase/IMP cyclohydrolase
4	**FS42_20790**	hypothetical protein	FS43_23080	hypothetical protein	FS44_800	hypothetical protein	WQG_780	hypothetical protein
4	**FS42_2080**	Esterase super	FS43_2740	Esterase super	FS44_20320	Esterase super	WQG_20510	Esterase super
4	**FS42_20800**	flavin-dependent dehydrogenase	FS43_23090	flavin-dependent dehydrogenase	FS44_780	flavin-dependent dehydrogenase	WQG_760	flavin-dependent dehydrogenase
4	**FS42_20830**	Glutamine ABC transporter	FS43_23100	Glutamine ABC transporter	FS44_770	Glutamine ABC transporter	WQG_750	Glutamine ABC transporter
4	**FS42_20840**	reductase	FS43_23110	reductase	FS44_760	reductase	WQG_740	reductase
4	**FS42_20860**	bacterioferritin, an iron storage homoprotein	FS43_23140	bacterioferritin, an iron storage homoprotein	FS44_730	bacterioferritin, an iron storage homoprotein	WQG_710	bacterioferritin, an iron storage homoprotein
4	**FS42_20870**	PykF protein	FS43_23150	PykF protein	FS44_720	PykF protein	WQG_700	PykF protein
4	**FS42_20880**	NADPH dehydrogenase	FS43_23160	NADPH dehydrogenase	FS44_710	NADPH dehydrogenase	WQG_690	NADPH dehydrogenase
4	**FS42_20890**	Luciferase family monooxygenase	FS43_23170	Luciferase family monooxygenase	FS44_700	Luciferase family monooxygenase	WQG_680	Luciferase family monooxygenase
4	**FS42_2090**	Phosphoribosylglycinamide synthetase	FS43_2750	Phosphoribosylglycinamide synthetase	FS44_20310	Phosphoribosylglycinamide synthetase	WQG_20500	Phosphoribosylglycinamide synthetase
4	**FS42_20900**	OsmC protein	FS43_23180	OsmC protein	FS44_690	OsmC protein	WQG_670	OsmC protein
4	**FS42_20910**	Glutathione S-transferase domain protein	FS43_23190	Glutathione S-transferase domain protein	FS44_680	Glutathione S-transferase domain protein	WQG_660	Glutathione S-transferase domain protein
4	**FS42_20920**	Phospholipase, patatin	FS43_23200	Phospholipase, patatin	FS44_670	Phospholipase, patatin	WQG_650	Phospholipase, patatin
4	**FS42_20950**	Phosphotransferase	FS43_23230	Phosphotransferase	FS44_640	Phosphotransferase	WQG_620	Phosphotransferase
4	**FS42_20960**	Ribokinase	FS43_23240	Ribokinase	FS44_630	Ribokinase	WQG_610	Ribokinase
4	**FS42_20970**	D-ribose-binding periplasmic protein	FS43_23250	D-ribose-binding periplasmic protein	FS44_620	D-ribose-binding periplasmic protein	WQG_600	D-ribose-binding periplasmic protein
4	**FS42_20980**	Ribose transport system permease protein rbsC	FS43_23260	Ribose transport system permease protein rbsC	FS44_610	Ribose transport system permease protein rbsC	WQG_590	Ribose transport system permease protein rbsC
4	**FS42_20990**	drug resistance protein	FS43_23270	hypothetical protein	FS44_600	drug resistance protein	WQG_580	drug resistance protein
4	**FS42_210**	Periplasmic binding protein/LacI transcriptional regulator	FS43_220	Periplasmic binding protein/LacI transcriptional regulator	FS44_22930	Periplasmic binding protein/LacI transcriptional regulator	WQG_22400	Periplasmic binding protein/LacI transcriptional regulator
4	**FS42_2100**	ArsC protein	FS43_2760	ArsC protein	FS44_20300	ArsC protein	WQG_20490	ArsC protein
4	**FS42_21000**	Ribose import ATP-binding protein RbsA	FS43_23280	Ribose import ATP-binding protein RbsA	FS44_590	Ribose import ATP-binding protein RbsA	WQG_570	Ribose import ATP-binding protein RbsA
4	**FS42_21010**	D-ribose pyranase	FS43_23290	D-ribose pyranase	FS44_580	D-ribose pyranase	WQG_560	D-ribose pyranase
4	**FS42_21020**	Alanine racemase	FS43_23300	Alanine racemase	FS44_570	Alanine racemase	WQG_550	Alanine racemase
4	**FS42_21030**	L-lactate dehydrogenase	FS43_23310	L-lactate dehydrogenase	FS44_560	L-lactate dehydrogenase	WQG_540	L-lactate dehydrogenase
4	**FS42_21040**	Sodium/proline symporter	FS43_23320	Sodium/proline symporter	FS44_550	Sodium/proline symporter	WQG_530	Sodium/proline symporter
4	**FS42_21050**	1-pyrroline-5-carboxylate dehydrogenase /proline dehydrogenase	FS43_23330	1-pyrroline-5-carboxylate dehydrogenase /proline dehydrogenase	FS44_540	1-pyrroline-5-carboxylate dehydrogenase /proline dehydrogenase	WQG_520	1-pyrroline-5-carboxylate dehydrogenase /proline dehydrogenase
4	**FS42_21060**	Lipoprotein	FS43_23340	Lipoprotein	FS44_530	hypothetical protein	WQG_510	Lipoprotein
4	**FS42_21070**	hypothetical protein	FS43_23350	hypothetical protein	FS44_520	hypothetical protein	WQG_500	hypothetical protein
4	**FS42_21080**	NADH:flavin oxidoreductase/NADH oxidase	FS43_23360	NADH:flavin oxidoreductase/NADH oxidase	FS44_510	NADH:flavin oxidoreductase/NADH oxidase	WQG_490	NADH:flavin oxidoreductase/NADH oxidase
4	**FS42_2110**	Dihydropteroate synthase	FS43_2770	Dihydropteroate synthase	FS44_20290	Dihydropteroate synthase	WQG_20480	Dihydropteroate synthase
4	**FS42_21110**	hypothetical protein	FS43_23390	hypothetical protein	FS44_500	hypothetical protein	WQG_460	hypothetical protein
4	**FS42_21120**	Transcriptional regulator, LysR	FS43_23400	Transcriptional regulator, LysR	FS44_490	Transcriptional regulator, LysR	WQG_450	Transcriptional regulator, LysR
4	**FS42_21130**	Dioxygenase	FS43_23410	Dioxygenase	FS44_460	Dioxygenase	WQG_440	Dioxygenase
4	**FS42_21170**	hypothetical protein	FS43_23450	hypothetical protein	FS44_450	hypothetical protein	WQG_400	hypothetical protein
4	**FS42_21190**	Transcriptional regulator, LysR	FS43_23470	Transcriptional regulator, LysR	FS44_430	Transcriptional regulator, LysR	WQG_380	Transcriptional regulator, LysR
4	**FS42_2120**	transport system permease protein	FS43_2780	transport system permease protein	FS44_20280	transport system permease protein	WQG_20470	transport system permease protein
4	**FS42_21200**	AcrR family transcriptional regulator	FS43_23480	AcrR family transcriptional regulator	FS44_410	AcrR family transcriptional regulator	WQG_370	AcrR family transcriptional regulator
4	**FS42_21210**	AraC family transcriptional regulator	FS43_23490	AraC family transcriptional regulator	FS44_390	AraC family transcriptional regulator	WQG_360	AraC family transcriptional regulator
4	**FS42_21220**	Thioredoxin	FS43_23500	Thioredoxin	FS44_380	Thioredoxin	WQG_350	Thioredoxin
4	**FS42_21230**	reductase	FS43_23510	reductase	FS44_370	reductase	WQG_340	reductase
4	**FS42_21240**	Carboxymuconolactone decarboxylase	FS43_23520	Carboxymuconolactone decarboxylase	FS44_360	Alkylhydroperoxidase like protein, AhpD	WQG_330	Carboxymuconolactone decarboxylase
4	**FS42_21250**	Thioredoxin	FS43_23530	Thioredoxin	FS44_350	Thioredoxin	WQG_320	Thioredoxin
4	**FS42_21260**	Transcriptional regulator, AraC	FS43_23540	Transcriptional regulator, AraC	FS44_340	Transcriptional regulator, AraC	WQG_310	Transcriptional regulator, AraC
4	**FS42_21270**	Phospholipase/Carboxylesterase	FS43_23550	Phospholipase/Carboxylesterase	FS44_330	Phospholipase/Carboxylesterase	WQG_300	Phospholipase/Carboxylesterase
4	**FS42_21280**	DSBA oxidoreductase	FS43_23560	DSBA oxidoreductase	FS44_320	DSBA oxidoreductase	WQG_290	DSBA oxidoreductase
4	**FS42_21290**	LysR family transcriptional regulator protein	FS43_23570	LysR family transcriptional regulator protein	FS44_16170	LysR protein	WQG_280	LysR family transcriptional regulator protein
4	**FS42_2130**	hypothetical protein	FS43_2790	hypothetical protein	FS44_20270	hypothetical protein	WQG_20460	hypothetical protein
4	**FS42_21310**	reductase	FS43_23590	reductase	FS44_310	reductase	WQG_260	reductase
4	**FS42_21320**	RhaT protein	FS43_23600	RhaT protein	FS44_290	RhaT protein	WQG_250	RhaT protein
4	**FS42_21330**	GloB protein	FS43_23610	GloB protein	FS44_280	GloB protein	WQG_240	GloB protein
4	**FS42_21340**	hypothetical protein	FS43_23620	hypothetical protein	FS44_270	hypothetical protein	WQG_230	hypothetical protein
4	**FS42_21350**	Beta-lactamase domain protein	FS43_23630	Beta-lactamase domain protein	FS44_260	Beta-lactamase domain protein	WQG_220	Beta-lactamase domain protein
4	**FS42_21360**	Phospholipase, patatin	FS43_23640	Phospholipase, patatin	FS44_250	Phospholipase, patatin	WQG_210	Phospholipase, patatin
4	**FS42_21370**	MgtC family membrane protein	FS43_23650	MgtC family membrane protein	FS44_240	MgtC family membrane protein	WQG_200	MgtC family membrane protein
4	**FS42_21380**	ATP synthase epsilon chain	FS43_23660	ATP synthase epsilon chain	FS44_230	ATP synthase epsilon chain	WQG_190	ATP synthase epsilon chain
4	**FS42_21390**	ATP synthase subunit beta	FS43_23670	ATP synthase subunit beta	FS44_220	ATP synthase subunit beta	WQG_180	ATP synthase subunit beta
4	**FS42_2140**	Succinyl-diaminopimelate desuccinylase	FS43_2800	Succinyl-diaminopimelate desuccinylase	FS44_20260	Succinyl-diaminopimelate desuccinylase	WQG_20450	Succinyl-diaminopimelate desuccinylase
4	**FS42_21400**	ATP synthase gamma chain	FS43_23680	ATP synthase gamma chain	FS44_210	ATP synthase gamma chain	WQG_170	ATP synthase gamma chain
4	**FS42_21410**	ATP synthase subunit alpha	FS43_23690	ATP synthase subunit alpha	FS44_200	ATP synthase subunit alpha	WQG_160	ATP synthase subunit alpha
4	**FS42_21420**	ATP synthase subunit delta	FS43_23700	ATP synthase subunit delta	FS44_190	ATP synthase subunit delta	WQG_150	ATP synthase subunit delta
4	**FS42_21430**	ATP synthase subunit b	FS43_23710	ATP synthase subunit b	FS44_180	ATP synthase subunit b	WQG_140	ATP synthase subunit b
4	**FS42_21440**	ATP synthase subunit c	FS43_23720	ATP synthase subunit c	FS44_170	ATP synthase subunit c	WQG_130	ATP synthase subunit c
4	**FS42_21450**	F0F1-type ATP synthase, alpha subunit	FS43_23730	F0F1-type ATP synthase, alpha subunit	FS44_160	F0F1-type ATP synthase, alpha subunit	WQG_120	F0F1-type ATP synthase, alpha subunit
4	**FS42_21460**	ATP synthase protein I	FS43_23740	ATP synthase protein I	FS44_150	ATP synthase protein I	WQG_110	ATP synthase protein I
4	**FS42_21470**	ABC transporter protein	FS43_23750	ABC transporter protein	FS44_140	ABC transporter protein	WQG_100	ABC transporter protein
4	**FS42_21480**	Binding-protein-dependent transport system inner membrane component	FS43_23760	Binding-protein-dependent transport system inner membrane component	FS44_130	Binding-protein-dependent transport system inner membrane component	WQG_90	Binding-protein-dependent transport system inner membrane component
4	**FS42_21490**	ABC transporter protein	FS43_23770	ABC transporter protein	FS44_120	ABC transporter protein	WQG_80	ABC transporter protein
4	**FS42_2150**	Autotransporter adhesin	FS43_2810	Autotransporter adhesin	FS44_20250	Autotransporter adhesin	WQG_20440	Autotransporter adhesin
4	**FS42_21500**	Extracellular solute-binding protein family 1	FS43_23780	Extracellular solute-binding protein family 1	FS44_110	Extracellular solute-binding protein family 1	WQG_70	Extracellular solute-binding protein family 1
4	**FS42_21510**	Two component transcriptional regulator, Fis	FS43_23790	Two component transcriptional regulator, Fis	FS44_90	Two component transcriptional regulator, Fis	WQG_60	Two component transcriptional regulator, Fis
4	**FS42_21520**	Ribosomal RNA small subunit methyltransferase G	FS43_23800	Ribosomal RNA small subunit methyltransferase G	FS44_80	Ribosomal RNA small subunit methyltransferase G	WQG_50	Ribosomal RNA small subunit methyltransferase G
4	**FS42_21530**	Cell filamentation protein Fic-related protein	FS43_23810	Cell filamentation protein Fic-related protein	FS44_70	Thiamine-phosphate kinase	WQG_40	Cell filamentation protein Fic-related protein
4	**FS42_21540**	tRNA uridine 5-carboxymethylaminomethyl modification enzyme MnmG	FS43_23820	tRNA uridine 5-carboxymethylaminomethyl modification enzyme MnmG	FS44_50	tRNA uridine 5-carboxymethylaminomethyl modification enzyme MnmG	WQG_30	tRNA uridine 5-carboxymethylaminomethyl modification enzyme MnmG
4	**FS42_21550**	Ribosomal protein L11 methyltransferase	FS43_23830	Ribosomal protein L11 methyltransferase	FS44_40	Ribosomal protein L11 methyltransferase	WQG_20	Ribosomal protein L11 methyltransferase
4	**FS42_2160**	ferredoxin-like protein	FS43_2820	ferredoxin-like protein	FS44_20240	ferredoxin-like protein	WQG_20430	ferredoxin-like protein

4	**FS42_2170**	NadR	FS43_2830	NadR	FS44_20230	NadR	WQG_20420	NadR
4	**FS42_2180**	hypothetical protein	FS43_2840	hypothetical protein	FS44_20220	hypothetical protein	WQG_20410	hypothetical protein
4	**FS42_2190**	Patatin	FS43_2850	Patatin	FS44_20210	Patatin	WQG_20400	Patatin
4	**FS42_220**	Methylmalonate-semialdehyde dehydrogenase	FS43_230	Methylmalonate-semialdehyde dehydrogenase	FS44_22920	Methylmalonate-semialdehyde dehydrogenase	WQG_22390	Methylmalonate-semialdehyde dehydrogenase
4	**FS42_2200**	membrane protein	FS43_2860	membrane protein	FS44_20200	membrane protein	WQG_20390	membrane protein
4	**FS42_2210**	Deoxyribose-phosphate aldolase	FS43_2870	Deoxyribose-phosphate aldolase	FS44_20190	Deoxyribose-phosphate aldolase	WQG_20380	Deoxyribose-phosphate aldolase
4	**FS42_2220**	Outer membrane lipoprotein pcp	FS43_2880	Outer membrane lipoprotein pcp	FS44_20180	Outer membrane lipoprotein pcp	WQG_20370	Outer membrane lipoprotein pcp
4	**FS42_2230**	hypothetical protein	FS43_2890	hypothetical protein	FS44_20170	hypothetical protein	WQG_20360	hypothetical protein
4	**FS42_2240**	Arginyl-tRNA synthetase	FS43_2900	Arginyl-tRNA synthetase	FS44_20160	Arginyl-tRNA synthetase	WQG_20350	Arginyl-tRNA synthetase
4	**FS42_2250**	50S ribosomal protein L14	FS43_2910	50S ribosomal protein L14	FS44_20150	50S ribosomal protein L14	WQG_20340	50S ribosomal protein L14
4	**FS42_2260**	50S ribosomal protein L24	FS43_2920	50S ribosomal protein L24	FS44_20140	50S ribosomal protein L24	WQG_20330	50S ribosomal protein L24
4	**FS42_2270**	50S ribosomal protein L5	FS43_2930	50S ribosomal protein L5	FS44_20130	50S ribosomal protein L5	WQG_20320	50S ribosomal protein L5
4	**FS42_2280**	30S ribosomal protein S14	FS43_2940	30S ribosomal protein S14	FS44_20120	30S ribosomal protein S14	WQG_20310	30S ribosomal protein S14
4	**FS42_2290**	30S ribosomal protein S8	FS43_2950	30S ribosomal protein S8	FS44_20110	30S ribosomal protein S8	WQG_20300	30S ribosomal protein S8
4	**FS42_230**	Myo-inositol catabolism IolB domain protein	FS43_240	Myo-inositol catabolism IolB domain protein	FS44_22910	Myo-inositol catabolism IolB domain protein	WQG_22380	Myo-inositol catabolism IolB domain protein
4	**FS42_2300**	50S ribosomal protein L6	FS43_2960	50S ribosomal protein L6	FS44_20100	50S ribosomal protein L6	WQG_20290	50S ribosomal protein L6
4	**FS42_2310**	50S ribosomal protein L18	FS43_2970	50S ribosomal protein L18	FS44_20090	50S ribosomal protein L18	WQG_20280	50S ribosomal protein L18
4	**FS42_2320**	30S ribosomal protein S5	FS43_2980	30S ribosomal protein S5	FS44_20080	30S ribosomal protein S5	WQG_20270	30S ribosomal protein S5
4	**FS42_2330**	50S ribosomal protein L30	FS43_2990	50S ribosomal protein L30	FS44_20070	50S ribosomal protein L30	WQG_20260	50S ribosomal protein L30
4	**FS42_2340**	50S ribosomal protein L15	FS43_3000	50S ribosomal protein L15	FS44_20060	50S ribosomal protein L15	WQG_20250	50S ribosomal protein L15
4	**FS42_2350**	Protein translocase subunit SecY	FS43_3010	Protein translocase subunit SecY	FS44_20050	Protein translocase subunit SecY	WQG_20240	Protein translocase subunit SecY
4	**FS42_2370**	30S ribosomal protein S13	FS43_3030	30S ribosomal protein S13	FS44_20040	30S ribosomal protein S13	WQG_20230	30S ribosomal protein S13
4	**FS42_2380**	30S ribosomal protein S11	FS43_3040	30S ribosomal protein S11	FS44_20030	30S ribosomal protein S11	WQG_20220	30S ribosomal protein S11
4	**FS42_2390**	30S ribosomal protein S4	FS43_3050	30S ribosomal protein S4	FS44_20020	30S ribosomal protein S4	WQG_20210	30S ribosomal protein S4
4	**FS42_240**	L-xylulose kinase	FS43_250	L-xylulose kinase	FS44_22900	L-xylulose kinase	WQG_22370	L-xylulose kinase
4	**FS42_2400**	DNA-directed RNA polymerase subunit alpha	FS43_3060	DNA-directed RNA polymerase subunit alpha	FS44_20010	DNA-directed RNA polymerase subunit alpha	WQG_20200	DNA-directed RNA polymerase subunit alpha
4	**FS42_2410**	50S ribosomal protein L17	FS43_3070	50S ribosomal protein L17	FS44_20000	50S ribosomal protein L17	WQG_20190	50S ribosomal protein L17
4	**FS42_2420**	30S ribosomal protein S16	FS43_3080	30S ribosomal protein S16	FS44_19990	30S ribosomal protein S16	WQG_20180	30S ribosomal protein S16
4	**FS42_2430**	Ribosome maturation factor rimM	FS43_3090	Ribosome maturation factor rimM	FS44_19980	Ribosome maturation factor rimM	WQG_20170	Ribosome maturation factor rimM
4	**FS42_2440**	tRNA (guanine-N(1)-)-methyltransferase	FS43_3100	tRNA (guanine-N(1)-)-methyltransferase	FS44_19970	tRNA (guanine-N(1)-)-methyltransferase	WQG_20160	tRNA (guanine-N(1)-)-methyltransferase
4	**FS42_2450**	50S ribosomal protein L19	FS43_3110	50S ribosomal protein L19	FS44_19960	50S ribosomal protein L19	WQG_20150	50S ribosomal protein L19
4	**FS42_2460**	hypothetical protein	FS43_3120	hypothetical protein	FS44_19950	hypothetical protein	WQG_20140	hypothetical protein
4	**FS42_2470**	hypothetical protein	FS43_3130	hypothetical protein	FS44_19930	hypothetical protein	WQG_20130	hypothetical protein
4	**FS42_2480**	hypothetical protein	FS43_3140	hypothetical protein	FS44_19920	hypothetical protein	WQG_20120	hypothetical protein
4	**FS42_2490**	L-asparaginase	FS43_3150	L-asparaginase	FS44_19910	L-asparaginase	WQG_20110	L-asparaginase
4	**FS42_250**	hypothetical protein	FS43_260	hypothetical protein	FS44_22890	hypothetical protein	WQG_22360	hypothetical protein
4	**FS42_2500**	Pyrroline-5-carboxylate reductase	FS43_3160	Pyrroline-5-carboxylate reductase	FS44_19900	Pyrroline-5-carboxylate reductase	WQG_20090	Pyrroline-5-carboxylate reductase
4	**FS42_2510**	hypothetical protein	FS43_3170	hypothetical protein	FS44_19890	hypothetical protein	WQG_20080	hypothetical protein
4	**FS42_2520**	Nucleoid occlusion factor SlmA	FS43_3180	Nucleoid occlusion factor SlmA	FS44_19880	Nucleoid occlusion factor SlmA	WQG_20070	Nucleoid occlusion factor SlmA
4	**FS42_2530**	Deoxyuridine 5'-triphosphate nucleotidohydrolase	FS43_3190	Deoxyuridine 5'-triphosphate nucleotidohydrolase	FS44_19870	Deoxyuridine 5'-triphosphate nucleotidohydrolase	WQG_20060	Deoxyuridine 5'-triphosphate nucleotidohydrolase
4	**FS42_2540**	Phosphopantothenoylcysteine decarboxylase/phosphopantothenate--cysteine ligase	FS43_3200	Phosphopantothenoylcysteine decarboxylase/phosphopantothenate--cysteine ligase	FS44_19860	Phosphopantothenoylcysteine decarboxylase/phosphopantothenate--cysteine ligase	WQG_20050	Phosphopantothenoylcysteine decarboxylase/phosphopantothenate--cysteine ligase
4	**FS42_2550**	hypothetical protein	FS43_3210	hypothetical protein	FS44_19850	hypothetical protein	WQG_20040	hypothetical protein
4	**FS42_2560**	hypothetical protein	FS43_3220	hypothetical protein	FS44_19840	hypothetical protein	WQG_20030	hypothetical protein
4	**FS42_2570**	50S ribosomal protein L28	FS43_3230	50S ribosomal protein L28	FS44_19830	50S ribosomal protein L28	WQG_20020	50S ribosomal protein L28
4	**FS42_2580**	50S ribosomal protein L33	FS43_3240	50S ribosomal protein L33	FS44_19820	50S ribosomal protein L33	WQG_20010	50S ribosomal protein L33
4	**FS42_2590**	XcbC	FS43_3250	XcbC	FS44_19810	XcbC	WQG_20000	XcbC
4	**FS42_260**	2,3-diketo-L-gulonate TRAP transporter large permease protein yiaN	FS43_270	2,3-diketo-L-gulonate TRAP transporter large permease protein yiaN	FS44_22880	2,3-diketo-L-gulonate TRAP transporter large permease protein yiaN	WQG_22350	2,3-diketo-L-gulonate TRAP transporter large permease protein yiaN
4	**FS42_2600**	Purine nucleosidase	FS43_3260	Purine nucleosidase	FS44_19800	Purine nucleosidase	WQG_19990	Purine nucleosidase
4	**FS42_2610**	Phosphomannomutase	FS43_3270	Phosphomannomutase	FS44_19790	Phosphomannomutase	WQG_19980	Phosphomannomutase
4	**FS42_2620**	Lipopolysaccharide biosynthesis protein RffC	FS43_3280	Lipopolysaccharide biosynthesis protein RffC	FS44_19780	Lipopolysaccharide biosynthesis protein RffC	WQG_19970	Lipopolysaccharide biosynthesis protein RffC
4	**FS42_2630**	UDP-N-acetyl-D-mannosamine dehydrogenase	FS43_3290	UDP-N-acetyl-D-mannosamine dehydrogenase	FS44_19770	UDP-N-acetyl-D-mannosamine dehydrogenase	WQG_19960	UDP-N-acetyl-D-mannosamine dehydrogenase
4	**FS42_2640**	UDP-N-acetylglucosamine 2-epimerase	FS43_3300	UDP-N-acetylglucosamine 2-epimerase	FS44_19760	UDP-N-acetylglucosamine 2-epimerase	WQG_19950	UDP-N-acetylglucosamine 2-epimerase
4	**FS42_2650**	hypothetical protein	FS43_3310	hypothetical protein	FS44_19750	hypothetical protein	WQG_19940	hypothetical protein
4	**FS42_2660**	Undecaprenyl-phosphate alpha-N-acetylglucosaminyltransferase	FS43_3320	Undecaprenyl-phosphate alpha-N-acetylglucosaminyltransferase	FS44_19740	Undecaprenyl-phosphate alpha-N-acetylglucosaminyltransferase	WQG_19930	Undecaprenyl-phosphate alpha-N-acetylglucosaminyltransferase
4	**FS42_2670**	Phosphoglycolate phosphatase	FS43_3330	Phosphoglycolate phosphatase	FS44_19730	Phosphoglycolate phosphatase	WQG_19920	Phosphoglycolate phosphatase
4	**FS42_2680**	hypothetical protein	FS43_3340	hypothetical protein	FS44_19720	hypothetical protein	WQG_19910	hypothetical protein
4	**FS42_2690**	50S ribosomal protein L27	FS43_3350	50S ribosomal protein L27	FS44_19710	50S ribosomal protein L27	WQG_19900	50S ribosomal protein L27
4	**FS42_270**	TRAP transporter small permease protein	FS43_280	TRAP transporter small permease protein	FS44_22870	TRAP transporter small permease protein	WQG_22340	TRAP transporter small permease protein
4	**FS42_2700**	50S ribosomal protein L21	FS43_3360	50S ribosomal protein L21	FS44_19700	50S ribosomal protein L21	WQG_19890	50S ribosomal protein L21
4	**FS42_2710**	hypothetical protein	FS43_3370	hypothetical protein	FS44_19690	hypothetical protein	WQG_19880	hypothetical protein
4	**FS42_2720**	N-acetylglucosamine-6-phosphate deacetylase	FS43_3380	N-acetylglucosamine-6-phosphate deacetylase	FS44_19680	N-acetylglucosamine-6-phosphate deacetylase	WQG_19870	N-acetylglucosamine-6-phosphate deacetylase
4	**FS42_2730**	Glucosamine-6-phosphate deaminase	FS43_3390	Glucosamine-6-phosphate deaminase	FS44_19670	Glucosamine-6-phosphate deaminase	WQG_19860	Glucosamine-6-phosphate deaminase
4	**FS42_2740**	Ribosomal RNA small subunit methyltransferase D	FS43_3400	Ribosomal RNA small subunit methyltransferase D	FS44_19660	Ribosomal RNA small subunit methyltransferase D	WQG_19850	Ribosomal RNA small subunit methyltransferase D
4	**FS42_2750**	Cell division protein FtsY-like protein	FS43_3410	Cell division protein FtsY-like protein	FS44_19650	Cell division protein FtsY-like protein	WQG_19840	Cell division protein FtsY-like protein
4	**FS42_2760**	Cell division ATP-binding protein FtsE	FS43_3420	Cell division ATP-binding protein FtsE	FS44_19640	Cell division ATP-binding protein FtsE	WQG_19830	Cell division ATP-binding protein FtsE
4	**FS42_2770**	Cell division protein FtsX	FS43_3430	Cell division protein FtsX	FS44_19630	Cell division protein FtsX	WQG_19820	Cell division protein FtsX
4	**FS42_2780**	CirA protein	FS43_3440	CirA protein	FS44_19620	CirA protein	WQG_19810	CirA protein
4	**FS42_2790**	Transport system permease protein	FS43_3450	Transport system permease protein	FS44_19610	Transport system permease protein	WQG_19800	Transport system permease protein
4	**FS42_280**	hypothetical protein	FS43_290	hypothetical protein	FS44_22860	hypothetical protein	WQG_22330	hypothetical protein
4	**FS42_2800**	ABC-type Fe3+-hydroxamate transport system, periplasmic component	FS43_3460	ABC-type Fe3+-hydroxamate transport system, periplasmic component	FS44_19600	ABC-type Fe3+-hydroxamate transport system, periplasmic component	WQG_19790	ABC-type Fe3+-hydroxamate transport system, periplasmic component
4	**FS42_2810**	ABC transporter protein	FS43_3470	ABC transporter protein	FS44_19590	ABC transporter protein	WQG_19780	ABC transporter protein
4	**FS42_2820**	3-octaprenyl-4-hydroxybenzoate carboxy-lyase	FS43_3480	3-octaprenyl-4-hydroxybenzoate carboxy-lyase	FS44_19580	3-octaprenyl-4-hydroxybenzoate carboxy-lyase	WQG_19770	3-octaprenyl-4-hydroxybenzoate carboxy-lyase
4	**FS42_2830**	Spermidine/putrescine-binding periplasmic protein 2	FS43_3490	Spermidine/putrescine-binding periplasmic protein 2	FS44_19570	Spermidine/putrescine-binding periplasmic protein 2	WQG_19760	Spermidine/putrescine-binding periplasmic protein 2
4	**FS42_2840**	GPR1/FUN34/yaaH	FS43_3500	GPR1/FUN34/yaaH	FS44_19560	GPR1/FUN34/yaaH	WQG_19750	GPR1/FUN34/yaaH
4	**FS42_2850**	PTS system mannose-specific EIIAB component	FS43_3510	PTS system mannose-specific EIIAB component	FS44_19550	PTS system mannose-specific EIIAB component	WQG_19740	PTS system mannose-specific EIIAB component
4	**FS42_2860**	Mannose permease IIC component	FS43_3520	Mannose permease IIC component	FS44_19540	Mannose permease IIC component	WQG_19730	Mannose permease IIC component
4	**FS42_2870**	Mannose permease IID component	FS43_3530	Mannose permease IID component	FS44_19530	Mannose permease IID component	WQG_19720	Mannose permease IID component
4	**FS42_2880**	hypothetical protein	FS43_3540	hypothetical protein	FS44_19520	hypothetical protein	WQG_19710	hypothetical protein
4	**FS42_2890**	Mannose-6-phosphate isomerase	FS43_3550	Mannose-6-phosphate isomerase	FS44_19510	Mannose-6-phosphate isomerase	WQG_19700	Mannose-6-phosphate isomerase
4	**FS42_290**	2,3-diketo-L-gulonate reductase	FS43_300	2,3-diketo-L-gulonate reductase	FS44_22850	2,3-diketo-L-gulonate reductase	WQG_22320	2,3-diketo-L-gulonate reductase
4	**FS42_2900**	Fe(3+) ions import ATP-binding protein FbpC	FS43_3560	Fe(3+) ions import ATP-binding protein FbpC	FS44_19500	Fe(3+) ions import ATP-binding protein FbpC	WQG_19690	Fe(3+) ions import ATP-binding protein FbpC
4	**FS42_2910**	Ferric transport system permease protein fbpB	FS43_3570	Ferric transport system permease protein fbpB	FS44_19490	Ferric transport system permease protein fbpB	WQG_19680	Ferric transport system permease protein fbpB
4	**FS42_2920**	hypothetical protein	FS43_3580	hypothetical protein	FS44_19480	hypothetical protein	WQG_19670	hypothetical protein
4	**FS42_2930**	Ribonucleotide reductase, alpha subunit	FS43_3590	Ribonucleotide reductase, alpha subunit	FS44_19470	Ribonucleotide reductase, alpha subunit	WQG_19660	Ribonucleotide reductase, alpha subunit
4	**FS42_2940**	reductase	FS43_3600	reductase	FS44_19460	reductase	WQG_19650	reductase
4	**FS42_2950**	Transcriptional regulator, DeoR	FS43_3610	Transcriptional regulator, DeoR	FS44_19450	Transcriptional regulator, DeoR	WQG_19640	Transcriptional regulator, DeoR
4	**FS42_2960**	Glucosamine--fructose-6-phosphate aminotransferase, isomerizing	FS43_3620	Glucosamine--fructose-6-phosphate aminotransferase, isomerizing	FS44_19440	Glucosamine--fructose-6-phosphate aminotransferase, isomerizing	WQG_19630	Glucosamine--fructose-6-phosphate aminotransferase, isomerizing
4	**FS42_2970**	Amino acid ABC transporter, periplasmic amino acid-binding protein	FS43_3630	Amino acid ABC transporter, periplasmic amino acid-binding protein	FS44_19430	Amino acid ABC transporter, periplasmic amino acid-binding protein	WQG_19620	Amino acid ABC transporter, periplasmic amino acid-binding protein
4	**FS42_2980**	hypothetical protein	FS43_3640	hypothetical protein	FS44_19420	hypothetical protein	WQG_19600	hypothetical protein
4	**FS42_2990**	hypothetical protein	FS43_3650	hypothetical protein	FS44_19410	hypothetical protein	WQG_19590	hypothetical protein
4	**FS42_300**	HTH-type transcriptional regulator	FS43_310	HTH-type transcriptional regulator	FS44_22840	HTH-type transcriptional regulator	WQG_22310	HTH-type transcriptional regulator
4	**FS42_3000**	Inner membrane protein oxaA	FS43_3660	Inner membrane protein oxaA	FS44_19400	Inner membrane protein oxaA	WQG_19570	Inner membrane protein oxaA
4	**FS42_3020**	Carbamoyl-phosphate synthase large chain	FS43_3670	Carbamoyl-phosphate synthase large chain	FS44_19380	Carbamoyl-phosphate synthase large chain	WQG_19560	Carbamoyl-phosphate synthase large chain
4	**FS42_3030**	Carbamoyl-phosphate synthase small chain	FS43_3680	Carbamoyl-phosphate synthase small chain	FS44_19370	Carbamoyl-phosphate synthase small chain	WQG_19550	Carbamoyl-phosphate synthase small chain
4	**FS42_3040**	hypothetical protein	FS43_3690	hypothetical protein	FS44_19360	hypothetical protein	WQG_19540	hypothetical protein
4	**FS42_3050**	hypothetical protein	FS43_3700	hypothetical protein	FS44_19350	hypothetical protein	WQG_19530	hypothetical protein
4	**FS42_3060**	(Dimethylallyl)adenosine tRNA methylthiotransferase MiaB	FS43_3710	(Dimethylallyl)adenosine tRNA methylthiotransferase MiaB	FS44_19340	(Dimethylallyl)adenosine tRNA methylthiotransferase MiaB	WQG_19520	(Dimethylallyl)adenosine tRNA methylthiotransferase MiaB
4	**FS42_3070**	phosphotransferase	FS43_3720	phosphotransferase	FS44_19330	phosphotransferase	WQG_19510	phosphotransferase
4	**FS42_3080**	Phosphoenolpyruvate-protein phosphotransferase	FS43_3730	Phosphoenolpyruvate-protein phosphotransferase	FS44_19320	Phosphoenolpyruvate-protein phosphotransferase	WQG_19500	Phosphoenolpyruvate-protein phosphotransferase
4	**FS42_3090**	Phosphocarrier protein HPr	FS43_3740	Phosphocarrier protein HPr	FS44_19310	Phosphocarrier protein HPr	WQG_19490	Phosphocarrier protein HPr
4	**FS42_310**	Membrane-bound lytic murein transglycosylase C	FS43_320	Membrane-bound lytic murein transglycosylase C	FS44_22830	Membrane-bound lytic murein transglycosylase C	WQG_22300	Membrane-bound lytic murein transglycosylase C
4	**FS42_3100**	hypothetical protein	FS43_3750	hypothetical protein	FS44_19300	hypothetical protein	WQG_19480	hypothetical protein
4	**FS42_3110**	Aspartate-semialdehyde dehydrogenase	FS43_3760	Aspartate-semialdehyde dehydrogenase	FS44_19290	Aspartate-semialdehyde dehydrogenase	WQG_19470	Aspartate-semialdehyde dehydrogenase
4	**FS42_3120**	hypothetical protein	FS43_3770	hypothetical protein	FS44_19280	hypothetical protein	WQG_19460	hypothetical protein
4	**FS42_3130**	LOG family protein ygdH	FS43_3780	LOG family protein ygdH	FS44_19270	LOG family protein ygdH	WQG_19450	LOG family protein ygdH
4	**FS42_3140**	hypothetical protein	FS43_3790	hypothetical protein	FS44_19260	hypothetical protein	WQG_19440	hypothetical protein
4	**FS42_3150**	Porin Gram-negative type	FS43_3800	Porin Gram-negative type	FS44_19250	Porin Gram-negative type	WQG_19430	Porin Gram-negative type
4	**FS42_3160**	Lipid-A-disaccharide synthase	FS43_3810	Lipid-A-disaccharide synthase	FS44_19240	Lipid-A-disaccharide synthase	WQG_19420	Lipid-A-disaccharide synthase
4	**FS42_3170**	alpha/beta superfamily hydrolase	FS43_3820	alpha/beta superfamily hydrolase	FS44_19230	alpha/beta superfamily hydrolase	WQG_19410	alpha/beta superfamily hydrolase
4	**FS42_3190**	Ribosomal large subunit pseudouridine synthase C	FS43_3840	Ribosomal large subunit pseudouridine synthase C	FS44_19220	Ribosomal large subunit pseudouridine synthase C	WQG_19390	Ribosomal large subunit pseudouridine synthase C
4	**FS42_320**	Fe(2+)-trafficking protein	FS43_330	Fe(2+)-trafficking protein	FS44_22820	Fe(2+)-trafficking protein	WQG_22290	Fe(2+)-trafficking protein
4	**FS42_3200**	Fructokinase	FS43_3850	Fructokinase	FS44_19210	Fructokinase	WQG_19380	Fructokinase
4	**FS42_3210**	SacC protein	FS43_3860	SacC protein	FS44_19200	SacC protein	WQG_19370	SacC protein
4	**FS42_3220**	Sucrose operon repressor	FS43_3870	Sucrose operon repressor	FS44_19190	Sucrose operon repressor	WQG_19360	Sucrose operon repressor
4	**FS42_3230**	sodium-dependent transporter	FS43_3880	sodium-dependent transporter	FS44_19180	sodium-dependent transporter	WQG_19350	sodium-dependent transporter
4	**FS42_3240**	iron--sulfur cluster insertion protein erpA	FS43_3890	iron--sulfur cluster insertion protein erpA	FS44_19170	iron--sulfur cluster insertion protein erpA	WQG_19340	iron--sulfur cluster insertion protein erpA
4	**FS42_3250**	dTDP-glucose 4,6-dehydratase 2	FS43_3900	dTDP-glucose 4,6-dehydratase 2	FS44_19160	dTDP-glucose 4,6-dehydratase 2	WQG_19330	dTDP-glucose 4,6-dehydratase 2
4	**FS42_3260**	Glucose-1-phosphate thymidylyltransferase	FS43_3910	Glucose-1-phosphate thymidylyltransferase	FS44_19150	Glucose-1-phosphate thymidylyltransferase	WQG_19320	Glucose-1-phosphate thymidylyltransferase
4	**FS42_3270**	dTDP-4-dehydrorhamnose reductase	FS43_3920	dTDP-4-dehydrorhamnose reductase	FS44_19140	dTDP-4-dehydrorhamnose reductase	WQG_19310	dTDP-4-dehydrorhamnose reductase
4	**FS42_3280**	dTDP-4-dehydrorhamnose 3,5-epimerase	FS43_3930	dTDP-4-dehydrorhamnose 3,5-epimerase	FS44_19130	dTDP-4-dehydrorhamnose 3,5-epimerase	WQG_19300	dTDP-4-dehydrorhamnose 3,5-epimerase
4	**FS42_3290**	ABC transporter integral membrane subunit	FS43_3940	ABC transporter integral membrane subunit	FS44_19120	ABC transporter integral membrane subunit	WQG_19290	ABC transporter integral membrane subunit
4	**FS42_330**	A/G-specific adenine glycosylase	FS43_340	A/G-specific adenine glycosylase	FS44_22810	A/G-specific adenine glycosylase	WQG_22280	A/G-specific adenine glycosylase
4	**FS42_3300**	ABC transporter ATP binding subunit	FS43_3950	ABC transporter ATP binding subunit	FS44_19110	Teichoic acids export ATP-binding protein TagH	WQG_19280	ABC transporter ATP binding subunit
4	**FS42_3330**	sugar transferase	FS43_3980	sugar transferase	FS44_19050	sugar transferase	WQG_19250	sugar transferase
4	**FS42_3340**	Ferrochelatase	FS43_3990	Ferrochelatase	FS44_19040	Ferrochelatase	WQG_19240	Ferrochelatase
4	**FS42_3350**	Metallopeptidase	FS43_4000	Metallopeptidase	FS44_19030	Metallopeptidase	WQG_19230	Metallopeptidase
4	**FS42_3360**	3-oxoacyl-[acyl-carrier-protein] synthase 1	FS43_4010	3-oxoacyl-[acyl-carrier-protein] synthase 1	FS44_19020	3-oxoacyl-[acyl-carrier-protein] synthase 1	WQG_19220	3-oxoacyl-[acyl-carrier-protein] synthase 1
4	**FS42_3370**	Soluble cytochrome b562	FS43_4020	Soluble cytochrome b562	FS44_19010	Soluble cytochrome b562	WQG_19210	Soluble cytochrome b562
4	**FS42_3380**	UPF0118 membrane protein	FS43_4030	UPF0118 membrane protein	FS44_19000	UPF0118 membrane protein	WQG_19200	UPF0118 membrane protein
4	**FS42_3390**	hypothetical protein	FS43_4040	hypothetical protein	FS44_18990	hypothetical protein	WQG_19190	hypothetical protein
4	**FS42_340**	hypothetical protein	FS43_350	hypothetical protein	FS44_22800	hypothetical protein	WQG_22270	hypothetical protein
4	**FS42_3400**	Outer membrane protein 18/16	FS43_4050	Outer membrane protein 18/16	FS44_18980	Outer membrane protein 18/16	WQG_19180	Outer membrane protein 18/16
4	**FS42_3410**	ABC superfamily ATP binding cassette transporter, binding protein	FS43_4060	ABC superfamily ATP binding cassette transporter, binding protein	FS44_18970	ABC superfamily ATP binding cassette transporter, binding protein	WQG_19170	ABC superfamily ATP binding cassette transporter, binding protein
4	**FS42_3420**	3-oxoacyl-[acyl-carrier-protein] synthase 3	FS43_4070	3-oxoacyl-[acyl-carrier-protein] synthase 3	FS44_18960	3-oxoacyl-[acyl-carrier-protein] synthase 3	WQG_19160	3-oxoacyl-[acyl-carrier-protein] synthase 3
4	**FS42_3430**	tRNA (guanine-N(7)-)-methyltransferase	FS43_4080	tRNA (guanine-N(7)-)-methyltransferase	FS44_18950	tRNA (guanine-N(7)-)-methyltransferase	WQG_19150	tRNA (guanine-N(7)-)-methyltransferase
4	**FS42_3440**	hypothetical protein	FS43_4090	hypothetical protein	FS44_18940	hypothetical protein	WQG_19140	hypothetical protein
4	**FS42_3450**	Integral membrane protein	FS43_4100	Integral membrane protein	FS44_18930	Integral membrane protein	WQG_19130	Integral membrane protein
4	**FS42_3460**	hypothetical protein	FS43_4110	hypothetical protein	FS44_18920	hypothetical protein	WQG_19120	hypothetical protein
4	**FS42_3470**	Met repressor	FS43_4120	Met repressor	FS44_18910	Met repressor	WQG_19110	Met repressor

4	**FS42_3480**	membrane protein	FS43_4130	membrane protein	FS44_18900	membrane protein	WQG_19100	membrane protein
4	**FS42_3490**	Sodium/pantothenate symporter	FS43_4140	Sodium/pantothenate symporter	FS44_18890	Sodium/pantothenate symporter	WQG_19090	Sodium/pantothenate symporter
4	**FS42_350**	GloA protein	FS43_360	GloA protein	FS44_22790	GloA protein	WQG_22260	GloA protein
4	**FS42_3500**	N-acetylmannosamine kinase	FS43_4150	N-acetylmannosamine kinase	FS44_18880	N-acetylmannosamine kinase	WQG_19080	N-acetylmannosamine kinase
4	**FS42_3510**	N-acetylmannosamine-6-phosphate 2-epimerase	FS43_4160	N-acetylmannosamine-6-phosphate 2-epimerase	FS44_18870	N-acetylmannosamine-6-phosphate 2-epimerase	WQG_19070	N-acetylmannosamine-6-phosphate 2-epimerase
4	**FS42_3520**	transporter	FS43_4170	transporter	FS44_18860	transporter	WQG_19060	transporter
4	**FS42_3530**	Deoxyribose-phosphate aldolase	FS43_4180	Deoxyribose-phosphate aldolase	FS44_18850	Deoxyribose-phosphate aldolase	WQG_19050	Deoxyribose-phosphate aldolase
4	**FS42_3540**	Purine nucleoside phosphorylase deoD-type	FS43_4190	Purine nucleoside phosphorylase deoD-type	FS44_18840	Purine nucleoside phosphorylase deoD-type	WQG_19040	Purine nucleoside phosphorylase deoD-type
4	**FS42_3550**	Transcriptional regulator	FS43_4200	Transcriptional regulator	FS44_18830	Transcriptional regulator	WQG_19030	Transcriptional regulator
4	**FS42_3560**	hypothetical protein	FS43_4210	hypothetical protein	FS44_18820	hypothetical protein	WQG_19020	hypothetical protein
4	**FS42_3570**	Permease of the drug/metabolite transporter (DMT) superfamily, conserved membrane protein	FS43_4220	Permease of the drug/metabolite transporter (DMT) superfamily, conserved membrane protein	FS44_18810	Permease of the drug/metabolite transporter (DMT) superfamily, conserved membrane protein	WQG_19010	Permease of the drug/metabolite transporter (DMT) superfamily, conserved membrane protein
4	**FS42_3580**	GTPase obg	FS43_4230	GTPase obg	FS44_18800	GTPase obg	WQG_19000	GTPase obg
4	**FS42_360**	Sugar fermentation stimulation protein	FS43_370	Sugar fermentation stimulation protein	FS44_22780	Sugar fermentation stimulation protein	WQG_22250	Sugar fermentation stimulation protein
4	**FS42_3600**	Threonine dehydratase biosynthetic	FS43_4250	Threonine dehydratase biosynthetic	FS44_18790	Threonine dehydratase biosynthetic	WQG_18980	Threonine dehydratase biosynthetic
4	**FS42_3610**	FKBP-type peptidyl-prolyl cis-trans isomerase slyD	FS43_4260	FKBP-type peptidyl-prolyl cis-trans isomerase slyD	FS44_18780	FKBP-type peptidyl-prolyl cis-trans isomerase slyD	WQG_18970	FKBP-type peptidyl-prolyl cis-trans isomerase slyD
4	**FS42_3620**	hypothetical protein	FS43_4270	hypothetical protein	FS44_18770	hypothetical protein	WQG_18960	hypothetical protein
4	**FS42_3630**	Aquaporin Z	FS43_4280	Aquaporin Z	FS44_18760	Aquaporin Z	WQG_18950	Aquaporin Z
4	**FS42_3640**	Periplasmic/secreted protein	FS43_4290	Periplasmic/secreted protein	FS44_18750	Periplasmic/secreted protein	WQG_18940	Periplasmic/secreted protein
4	**FS42_3650**	Protein YaeB	FS43_4300	Protein YaeB	FS44_18740	Protein YaeB	WQG_18930	Protein YaeB
4	**FS42_3660**	1,4-dihydroxy-2-naphthoate octaprenyltransferase	FS43_4310	1,4-dihydroxy-2-naphthoate octaprenyltransferase	FS44_18730	1,4-dihydroxy-2-naphthoate octaprenyltransferase	WQG_18920	1,4-dihydroxy-2-naphthoate octaprenyltransferase
4	**FS42_3670**	hypothetical protein	FS43_4320	hypothetical protein	FS44_18720	hypothetical protein	WQG_18910	hypothetical protein
4	**FS42_3680**	hydrolase	FS43_4330	hydrolase	FS44_18710	hydrolase	WQG_18900	hydrolase
4	**FS42_3690**	PTS system, beta-glucoside-specific IIABC subunit	FS43_4340	PTS system, beta-glucoside-specific IIABC subunit	FS44_18700	PTS system, beta-glucoside-specific IIABC subunit	WQG_18890	PTS system, beta-glucoside-specific IIABC subunit
4	**FS42_370**	Inner membrane protein yagU	FS43_380	Inner membrane protein yagU	FS44_22770	Inner membrane protein yagU	WQG_22240	Inner membrane protein yagU
4	**FS42_3700**	hypothetical protein	FS43_4350	hypothetical protein	FS44_18680	hypothetical protein	WQG_18880	hypothetical protein
4	**FS42_3710**	Transcriptional antiterminator, BglG	FS43_4360	Transcriptional antiterminator, BglG	FS44_18670	Transcriptional antiterminator, BglG	WQG_18870	Transcriptional antiterminator, BglG
4	**FS42_3720**	3-hydroxydecanoyl-(Acyl carrier protein) dehydratase	FS43_4370	3-hydroxydecanoyl-(Acyl carrier protein) dehydratase	FS44_18660	3-hydroxydecanoyl-(Acyl carrier protein) dehydratase	WQG_18860	3-hydroxydecanoyl-(Acyl carrier protein) dehydratase
4	**FS42_3730**	Ribosomal large subunit pseudouridine synthase A	FS43_4380	Ribosomal large subunit pseudouridine synthase A	FS44_18650	Ribosomal large subunit pseudouridine synthase A	WQG_18850	Ribosomal large subunit pseudouridine synthase A
4	**FS42_3740**	multidrug resistance protein norM	FS43_4390	multidrug resistance protein norM	FS44_18640	multidrug resistance protein norM	WQG_18840	multidrug resistance protein norM
4	**FS42_3750**	Membrane-bound lytic murein transglycosylase F	FS43_4400	Membrane-bound lytic murein transglycosylase F	FS44_18630	Membrane-bound lytic murein transglycosylase F	WQG_18830	Membrane-bound lytic murein transglycosylase F
4	**FS42_3760**	hypothetical protein	FS43_4410	hypothetical protein	FS44_18620	hypothetical protein	WQG_18820	hypothetical protein
4	**FS42_3770**	ABC transporter ATP-binding protein	FS43_4420	ABC transporter ATP-binding protein	FS44_18610	ABC transporter ATP-binding protein	WQG_18810	ABC transporter ATP-binding protein
4	**FS42_3780**	S-ribosylhomocysteine lyase	FS43_4430	S-ribosylhomocysteine lyase	FS44_18600	S-ribosylhomocysteine lyase	WQG_18800	S-ribosylhomocysteine lyase
4	**FS42_3790**	hypothetical protein	FS43_4440	hypothetical protein	FS44_18590	hypothetical protein	WQG_18790	hypothetical protein
4	**FS42_380**	ATP-dependent protease subunit HslV	FS43_390	ATP-dependent protease subunit HslV	FS44_22750	ATP-dependent protease subunit HslV	WQG_22230	ATP-dependent protease subunit HslV
4	**FS42_3800**	Ferric uptake regulation protein	FS43_4450	Ferric uptake regulation protein	FS44_18580	Ferric uptake regulation protein	WQG_18780	Ferric uptake regulation protein
4	**FS42_3810**	Flavodoxin	FS43_4460	Flavodoxin	FS44_18570	Flavodoxin	WQG_18770	Flavodoxin
4	**FS42_3820**	LexA regulated protein	FS43_4470	LexA regulated protein	FS44_18560	LexA regulated protein	WQG_18760	LexA regulated protein
4	**FS42_3830**	putative transcription regulator	FS43_4480	putative transcription regulator	FS44_18550	putative transcription regulator	WQG_18750	Conserved possible transcription regulator
4	**FS42_3840**	Aspartate-semialdehyde dehydrogenase	FS43_4490	Aspartate-semialdehyde dehydrogenase	FS44_18540	Aspartate-semialdehyde dehydrogenase	WQG_18740	Aspartate-semialdehyde dehydrogenase
4	**FS42_3850**	Catalase-peroxidase	FS43_4500	Catalase-peroxidase	FS44_18530	Catalase-peroxidase	WQG_18730	Catalase-peroxidase
4	**FS42_3860**	hypothetical protein	FS43_4510	hypothetical protein	FS44_18520	hypothetical protein	WQG_18720	hypothetical protein
4	**FS42_3870**	adenine permease PurP	FS43_4520	adenine permease PurP	FS44_18510	adenine permease PurP	WQG_18710	adenine permease PurP
4	**FS42_3880**	UPF0263 protein	FS43_4530	UPF0263 protein	FS44_18500	UPF0263 protein	WQG_18700	UPF0263 protein
4	**FS42_3890**	4-hydroxyphenylacetate 3-monooxygenase	FS43_4540	4-hydroxyphenylacetate 3-monooxygenase	FS44_18490	4-hydroxyphenylacetate 3-monooxygenase	WQG_18690	4-hydroxyphenylacetate 3-monooxygenase
4	**FS42_390**	ATP-dependent protease ATPase subunit HslU	FS43_400	ATP-dependent protease ATPase subunit HslU	FS44_22740	ATP-dependent protease ATPase subunit HslU	WQG_22220	ATP-dependent protease ATPase subunit HslU
4	**FS42_3900**	Tellurite resistance protein TehB	FS43_4550	Tellurite resistance protein TehB	FS44_18480	Tellurite resistance protein TehB	WQG_18680	Tellurite resistance protein TehB
4	**FS42_3910**	Regulator of ribonuclease activity A	FS43_4560	Regulator of ribonuclease activity A	FS44_18470	Regulator of ribonuclease activity A	WQG_18670	Regulator of ribonuclease activity A
4	**FS42_3920**	hypothetical protein	FS43_4570	hypothetical protein	FS44_18460	hypothetical protein	WQG_18660	hypothetical protein
4	**FS42_3930**	Ribonuclease HII	FS43_4580	Ribonuclease HII	FS44_18450	Ribonuclease HII	WQG_18650	Ribonuclease HII
4	**FS42_3940**	GCV family glycine cleavage complex aminomethyltransferase	FS43_4590	GCV family glycine cleavage complex aminomethyltransferase	FS44_18440	GCV family glycine cleavage complex aminomethyltransferase	WQG_18640	GCV family glycine cleavage complex aminomethyltransferase
4	**FS42_3950**	Threonine synthase	FS43_4600	Threonine synthase	FS44_18430	Threonine synthase	WQG_18630	Threonine synthase
4	**FS42_3960**	Translation initiation factor IF-1	FS43_4610	Translation initiation factor IF-1	FS44_18420	Translation initiation factor IF-1	WQG_18620	Translation initiation factor IF-1
4	**FS42_3970**	AphA-like protein/membrane protein affecting hemolysin expression	FS43_4620	AphA-like protein/membrane protein affecting hemolysin expression	FS44_18410	AphA-like protein/membrane protein affecting hemolysin expression	WQG_18610	AphA-like protein/membrane protein affecting hemolysin expression
4	**FS42_3980**	SerB protein	FS43_4630	SerB protein	FS44_18400	SerB protein	WQG_18600	SerB protein
4	**FS42_3990**	hypothetical protein	FS43_4640	hypothetical protein	FS44_18390	hypothetical protein	WQG_18590	hypothetical protein
4	**FS42_40**	Rod shape-determining protein RodA	FS43_50	Rod shape-determining protein RodA	FS44_23090	Rod shape-determining protein RodA	WQG_22570	Rod shape-determining protein RodA
4	**FS42_400**	Thiol disulfide oxidoreductase	FS43_410	Thiol disulfide oxidoreductase	FS44_22730	Thiol disulfide oxidoreductase	WQG_22210	Thiol disulfide oxidoreductase
4	**FS42_4000**	OrdL	FS43_4650	OrdL	FS44_18380	OrdL	WQG_18580	OrdL
4	**FS42_4010**	Biotin synthase	FS43_4660	Biotin synthase	FS44_18370	Biotin synthase	WQG_18570	Biotin synthase
4	**FS42_4020**	Ribosomal large subunit pseudouridine synthase B	FS43_4670	Ribosomal large subunit pseudouridine synthase B	FS44_18360	Ribosomal large subunit pseudouridine synthase B	WQG_18560	Ribosomal large subunit pseudouridine synthase B
4	**FS42_4030**	hypothetical protein	FS43_4680	hypothetical protein	FS44_18350	hypothetical protein	WQG_18550	hypothetical protein
4	**FS42_4040**	hypothetical protein	FS43_4690	hypothetical protein	FS44_18340	hypothetical protein	WQG_18540	hypothetical protein
4	**FS42_4050**	ArsR family transcriptional regulator	FS43_4700	ArsR family transcriptional regulator	FS44_18330	ArsR family transcriptional regulator	WQG_18530	ArsR family transcriptional regulator
4	**FS42_4060**	C4-dicarboxylate transporter/malic acid transport protein	FS43_4710	C4-dicarboxylate transporter/malic acid transport protein	FS44_18320	C4-dicarboxylate transporter/malic acid transport protein	WQG_18520	C4-dicarboxylate transporter/malic acid transport protein
4	**FS42_4070**	Inner membrane protein YgaP	FS43_4720	Inner membrane protein YgaP	FS44_18310	Inner membrane protein YgaP	WQG_18510	Inner membrane protein YgaP
4	**FS42_4080**	hypothetical protein	FS43_4730	hypothetical protein	FS44_18300	hypothetical protein	WQG_18500	hypothetical protein
4	**FS42_4090**	FAD-dependent pyridine nucleotide-disulfide oxidoreductase	FS43_4740	FAD-dependent pyridine nucleotide-disulfide oxidoreductase	FS44_18290	FAD-dependent pyridine nucleotide-disulfide oxidoreductase	WQG_18490	FAD-dependent pyridine nucleotide-disulfide oxidoreductase
4	**FS42_410**	5'-methylthioadenosine/S-adenosylhomocysteine nucleosidase	FS43_420	5'-methylthioadenosine/S-adenosylhomocysteine nucleosidase	FS44_22720	5'-methylthioadenosine/S-adenosylhomocysteine nucleosidase	WQG_22200	5'-methylthioadenosine/S-adenosylhomocysteine nucleosidase
4	**FS42_4110**	Transporter, drug/metabolite exporter	FS43_4760	Transporter, drug/metabolite exporter	FS44_18270	Transporter, drug/metabolite exporter	WQG_18470	Transporter, drug/metabolite exporter
4	**FS42_4120**	hypothetical protein	FS43_4770	hypothetical protein	FS44_18260	hypothetical protein	WQG_18460	hypothetical protein
4	**FS42_4130**	6,7-dimethyl-8-ribityllumazine synthase	FS43_4780	6,7-dimethyl-8-ribityllumazine synthase	FS44_18240	6,7-dimethyl-8-ribityllumazine synthase	WQG_18450	6,7-dimethyl-8-ribityllumazine synthase
4	**FS42_4140**	Riboflavin biosynthesis bifunctional protein RibBA	FS43_4790	Riboflavin biosynthesis bifunctional protein RibBA	FS44_18220	Riboflavin biosynthesis bifunctional protein RibBA	WQG_18440	Riboflavin biosynthesis bifunctional protein RibBA
4	**FS42_4150**	Riboflavin synthase alpha chain	FS43_4800	Riboflavin synthase alpha chain	FS44_18210	Riboflavin synthase alpha chain	WQG_18430	Riboflavin synthase alpha chain
4	**FS42_4160**	Riboflavin biosynthesis protein RibD	FS43_4810	Riboflavin biosynthesis protein RibD	FS44_18200	Riboflavin biosynthesis protein RibD	WQG_18420	Riboflavin biosynthesis protein RibD
4	**FS42_4170**	hypothetical protein	FS43_4820	hypothetical protein	FS44_18190	hypothetical protein	WQG_18410	hypothetical protein
4	**FS42_4180**	Peptidyl-prolyl cis-trans isomerase D	FS43_4830	Peptidyl-prolyl cis-trans isomerase D	FS44_18180	Peptidyl-prolyl cis-trans isomerase D	WQG_18400	Peptidyl-prolyl cis-trans isomerase D
4	**FS42_4190**	hypothetical protein	FS43_4840	hypothetical protein	FS44_18170	hypothetical protein	WQG_18390	hypothetical protein
4	**FS42_420**	hypothetical protein	FS43_430	hypothetical protein	FS44_22710	hypothetical protein	WQG_22190	hypothetical protein
4	**FS42_4200**	Na+/H+ antiporter	FS43_4850	Na+/H+ antiporter	FS44_18160	Na+/H+ antiporter	WQG_18380	Na+/H+ antiporter
4	**FS42_4210**	30S ribosomal protein S15	FS43_4860	30S ribosomal protein S15	FS44_18150	30S ribosomal protein S15	WQG_18370	30S ribosomal protein S15
4	**FS42_4220**	Autonomous glycyl radical cofactor	FS43_4870	Autonomous glycyl radical cofactor	FS44_18140	Autonomous glycyl radical cofactor	WQG_18360	Autonomous glycyl radical cofactor
4	**FS42_4230**	Uracil-DNA glycosylase	FS43_4880	Uracil-DNA glycosylase	FS44_18130	Uracil-DNA glycosylase	WQG_18350	Uracil-DNA glycosylase
4	**FS42_4240**	DNA recombination protein rmuC	FS43_4890	DNA recombination protein rmuC	FS44_18120	DNA recombination protein rmuC	WQG_18340	DNA recombination protein rmuC
4	**FS42_4250**	Anthranilate synthase component II	FS43_4900	Anthranilate synthase component II	FS44_18110	Anthranilate synthase component II	WQG_18330	Anthranilate synthase component II
4	**FS42_4260**	Fimbrial biogenesis and twitching motility protein PilF-like protein	FS43_4910	Fimbrial biogenesis and twitching motility protein PilF-like protein	FS44_18100	Fimbrial biogenesis and twitching motility protein PilF-like protein	WQG_18320	Fimbrial biogenesis and twitching motility protein PilF-like protein
4	**FS42_4270**	AEC family possible malonate efflux carrier	FS43_4920	AEC family possible malonate efflux carrier	FS44_18090	AEC family possible malonate efflux carrier	WQG_18310	AEC family possible malonate efflux carrier
4	**FS42_4280**	SufE protein probably involved in Fe-S center assembly	FS43_4930	SufE protein probably involved in Fe-S center assembly	FS44_18080	SufE protein probably involved in Fe-S center assembly	WQG_18300	SufE protein probably involved in Fe-S center assembly
4	**FS42_4290**	ATP-dependent Clp protease proteolytic subunit	FS43_4940	ATP-dependent Clp protease proteolytic subunit	FS44_18070	ATP-dependent Clp protease proteolytic subunit	WQG_18290	ATP-dependent Clp protease proteolytic subunit
4	**FS42_430**	Ribosomal protein S12 methylthiotransferase RimO	FS43_440	Ribosomal protein S12 methylthiotransferase RimO	FS44_22700	Ribosomal protein S12 methylthiotransferase RimO	WQG_22180	Ribosomal protein S12 methylthiotransferase RimO
4	**FS42_4300**	ATP-dependent Clp protease ATP-binding subunit ClpX	FS43_4950	ATP-dependent Clp protease ATP-binding subunit ClpX	FS44_18060	ATP-dependent Clp protease ATP-binding subunit ClpX	WQG_18280	ATP-dependent Clp protease ATP-binding subunit ClpX
4	**FS42_4320**	Tetraacyldisaccharide 4'-kinase	FS43_4970	Tetraacyldisaccharide 4'-kinase	FS44_18050	Tetraacyldisaccharide 4'-kinase	WQG_18260	Tetraacyldisaccharide 4'-kinase
4	**FS42_4330**	hypothetical protein	FS43_4980	hypothetical protein	FS44_18040	hypothetical protein	WQG_18250	hypothetical protein
4	**FS42_4340**	deoxyribonuclease	FS43_4990	deoxyribonuclease	FS44_18030	deoxyribonuclease	WQG_18240	deoxyribonuclease
4	**FS42_4350**	Competence protein F	FS43_5000	Competence protein F	FS44_18020	Competence protein F	WQG_18230	Competence protein F
4	**FS42_4360**	hydrolase	FS43_5010	hydrolase	FS44_18010	hydrolase	WQG_18220	hydrolase
4	**FS42_4370**	outer membrane protein	FS43_5020	outer membrane protein	FS44_18000	outer membrane protein	WQG_18210	outer membrane protein
4	**FS42_4380**	PtsG protein	FS43_5030	PtsG protein	FS44_17990	PtsG protein	WQG_18200	PtsG protein
4	**FS42_4390**	Trehalose-6-phosphate hydrolase	FS43_5040	Trehalose-6-phosphate hydrolase	FS44_17980	Trehalose-6-phosphate hydrolase	WQG_18190	Trehalose-6-phosphate hydrolase
4	**FS42_440**	O-linked N-acetylglucosaminetransferase	FS43_450	O-linked N-acetylglucosaminetransferase	FS44_22690	O-linked N-acetylglucosaminetransferase	WQG_22170	O-linked N-acetylglucosaminetransferase
4	**FS42_4400**	Tetratricopeptide repeat domain protein	FS43_5050	Tetratricopeptide repeat domain protein	FS44_17970	Tetratricopeptide repeat domain protein	WQG_18180	Tetratricopeptide repeat domain protein
4	**FS42_4410**	ModF protein	FS43_5070	ModF protein	FS44_17960	ModF protein	WQG_18170	ModF protein
4	**FS42_4420**	Threonine efflux protein	FS43_5080	Threonine efflux protein	FS44_17950	Threonine efflux protein	WQG_18160	Threonine efflux protein
4	**FS42_4430**	Acid phosphatase	FS43_5090	Acid phosphatase	FS44_17940	Acid phosphatase	WQG_18150	Acid phosphatase
4	**FS42_4440**	Thiamine-monophosphate kinase	FS43_5100	Thiamine-monophosphate kinase	FS44_17930	Thiamine-monophosphate kinase	WQG_18140	Thiamine-monophosphate kinase
4	**FS42_4450**	N utilization substance protein B	FS43_5110	N utilization substance protein B	FS44_17920	N utilization substance protein B	WQG_18130	N utilization substance protein B
4	**FS42_4460**	Competence protein E	FS43_5120	Competence protein E	FS44_17910	Competence protein E	WQG_18120	Competence protein E
4	**FS42_4470**	hypothetical protein	FS43_5130	hypothetical protein	FS44_17900	hypothetical protein	WQG_18110	hypothetical protein
4	**FS42_4480**	hypothetical protein	FS43_5140	hypothetical protein	FS44_17890	hypothetical protein	WQG_18100	hypothetical protein
4	**FS42_4490**	hypothetical protein	FS43_5150	hypothetical protein	FS44_17880	hypothetical protein	WQG_18090	hypothetical protein
4	**FS42_450**	lipoprotein	FS43_460	lipoprotein	FS44_22680	lipoprotein	WQG_22160	lipoprotein
4	**FS42_4500**	Tfp pilus assembly protein	FS43_5160	Tfp pilus assembly protein	FS44_17870	Tfp pilus assembly protein	WQG_18080	Tfp pilus assembly protein
4	**FS42_4510**	Penicillin-binding protein 1A	FS43_5170	Penicillin-binding protein 1A	FS44_17860	Penicillin-binding protein 1A	WQG_18070	Penicillin-binding protein 1A
4	**FS42_4520**	hypothetical protein	FS43_5180	hypothetical protein	FS44_17850	hypothetical protein	WQG_18060	hypothetical protein
4	**FS42_4530**	Shikimate kinase	FS43_5190	Shikimate kinase	FS44_17840	Shikimate kinase	WQG_18050	Shikimate kinase
4	**FS42_4540**	3-dehydroquinate synthase	FS43_5200	3-dehydroquinate synthase	FS44_17830	3-dehydroquinate synthase	WQG_18040	3-dehydroquinate synthase
4	**FS42_4550**	Dam	FS43_5210	Dam	FS44_17820	Dam	WQG_18030	Dam
4	**FS42_4560**	DNA polymerase IV	FS43_5220	DNA polymerase IV	FS44_17810	DNA polymerase IV	WQG_18020	DNA polymerase IV
4	**FS42_4570**	membrane protein, surface protein	FS43_5230	membrane protein, surface protein	FS44_17800	membrane protein, surface protein	WQG_18010	membrane protein, surface protein
4	**FS42_4580**	hypothetical protein	FS43_5240	hypothetical protein	FS44_9860	hypothetical protein	WQG_9480	hypothetical protein
4	**FS42_460**	Diaminopimelate decarboxylase	FS43_480	Diaminopimelate decarboxylase	FS44_22660	Diaminopimelate decarboxylase	WQG_22140	Diaminopimelate decarboxylase
4	**FS42_4600**	member of ShlA/HecA/FhaA exoprotein	FS43_5260	member of ShlA/HecA/FhaA exoprotein	FS44_17780	Filamentous hemagglutinin family outer membrane protein	WQG_17980	member of ShlA/HecA/FhaA exoprotein
4	**FS42_4680**	Carbon starvation protein CstA	FS43_5350	Carbon starvation protein CstA	FS44_17760	Carbon starvation protein CstA	WQG_17900	Carbon starvation protein CstA
4	**FS42_470**	Preprotein translocase SecE subunit	FS43_490	Preprotein translocase SecE subunit	FS44_22650	Preprotein translocase SecE subunit	WQG_22130	Preprotein translocase SecE subunit
4	**FS42_4710**	GMP reductase	FS43_5390	GMP reductase	FS44_17730	GMP reductase	WQG_17860	GMP reductase
4	**FS42_4720**	Glucose-6-phosphate isomerase	FS43_5400	Glucose-6-phosphate isomerase	FS44_17720	Glucose-6-phosphate isomerase	WQG_17850	Glucose-6-phosphate isomerase
4	**FS42_4730**	hypothetical protein	FS43_5410	hypothetical protein	FS44_17710	hypothetical protein	WQG_17840	hypothetical protein
4	**FS42_4740**	Shikimate dehydrogenase	FS43_5420	Shikimate dehydrogenase	FS44_17700	Shikimate dehydrogenase	WQG_17830	Shikimate dehydrogenase
4	**FS42_4750**	tRNA threonylcarbamoyladenosine biosynthesis protein RimN	FS43_5430	tRNA threonylcarbamoyladenosine biosynthesis protein RimN	FS44_17690	tRNA threonylcarbamoyladenosine biosynthesis protein RimN	WQG_17820	tRNA threonylcarbamoyladenosine biosynthesis protein RimN
4	**FS42_4760**	hypothetical protein	FS43_5440	hypothetical protein	FS44_17680	hypothetical protein	WQG_17810	hypothetical protein
4	**FS42_4770**	hypothetical protein	FS43_5450	hypothetical protein	FS44_17670	hypothetical protein	WQG_17800	hypothetical protein
4	**FS42_4780**	Regulatory protein recX	FS43_5460	Regulatory protein recX	FS44_17660	Regulatory protein recX	WQG_17790	Regulatory protein recX
4	**FS42_4790**	Protein RecA	FS43_5470	Protein RecA	FS44_17650	Protein RecA	WQG_17780	Protein RecA
4	**FS42_480**	Transcription antitermination protein nusG	FS43_500	Transcription antitermination protein nusG	FS44_22640	Transcription antitermination protein nusG	WQG_22120	Transcription antitermination protein nusG
4	**FS42_4800**	Branched-chain amino acid aminotransferase/4-amino-4-deoxychorismate lyase	FS43_5480	Branched-chain amino acid aminotransferase/4-amino-4-deoxychorismate lyase	FS44_17640	Branched-chain amino acid aminotransferase/4-amino-4-deoxychorismate lyase	WQG_17770	Branched-chain amino acid aminotransferase/4-amino-4-deoxychorismate lyase
4	**FS42_4810**	pabA-like protein	FS43_5490	pabA-like protein	FS44_17630	pabA-like protein	WQG_17760	pabA-like protein
4	**FS42_4820**	Thiamine-binding periplasmic protein	FS43_5500	Thiamine-binding periplasmic protein	FS44_17620	Thiamine-binding periplasmic protein	WQG_17750	Thiamine-binding periplasmic protein
4	**FS42_4830**	PhoH-like protein	FS43_5510	PhoH-like protein	FS44_17610	PhoH-like protein	WQG_17740	PhoH-like protein
4	**FS42_4840**	Transcription elongation factor greB	FS43_5520	Transcription elongation factor greB	FS44_17590	Transcription elongation factor greB	WQG_17730	Transcription elongation factor greB
4	**FS42_4850**	phosphosugar isomerase	FS43_5530	phosphosugar isomerase	FS44_17580	phosphosugar isomerase	WQG_17720	phosphosugar isomerase
4	**FS42_4860**	hypothetical protein	FS43_5540	hypothetical protein	FS44_17570	hypothetical protein	WQG_17710	hypothetical protein
4	**FS42_4870**	Glutamyl-tRNA synthetase	FS43_5550	Glutamyl-tRNA synthetase	FS44_17560	Glutamyl-tRNA synthetase	WQG_17700	Glutamyl-tRNA synthetase
4	**FS42_4880**	AraC family transcriptional regulator	FS43_5560	AraC family transcriptional regulator	FS44_17550	AraC family transcriptional regulator	WQG_17690	AraC family transcriptional regulator
4	**FS42_4890**	Isochorismatase	FS43_5570	Isochorismatase	FS44_17540	Isochorismatase	WQG_17680	Isochorismatase
4	**FS42_490**	50S ribosomal protein L11	FS43_510	50S ribosomal protein L11	FS44_22630	50S ribosomal protein L11	WQG_22110	50S ribosomal protein L11
4	**FS42_4900**	uracil permease	FS43_5580	uracil permease	FS44_17530	uracil permease	WQG_17670	uracil permease
4	**FS42_4910**	Dihydroorotate dehydrogenase	FS43_5590	Dihydroorotate dehydrogenase	FS44_17520	Dihydroorotate dehydrogenase	WQG_17660	Dihydroorotate dehydrogenase
4	**FS42_4920**	reductase	FS43_5600	reductase	FS44_17510	reductase	WQG_17650	reductase
4	**FS42_4930**	reductase	FS43_5610	reductase	FS44_17500	reductase	WQG_17640	reductase
4	**FS42_4940**	Thiol:disulfide interchange protein DsbE	FS43_5620	Thiol:disulfide interchange protein DsbE	FS44_17490	Thiol:disulfide interchange protein DsbE	WQG_17630	Thiol:disulfide interchange protein DsbE
4	**FS42_4950**	Membrane protein	FS43_5630	Membrane protein	FS44_17480	hypothetical protein	WQG_17620	hypothetical protein
4	**FS42_4960**	NrfE	FS43_5640	NrfE	FS44_17470	NrfE	WQG_17610	NrfE
4	**FS42_4970**	Glutathione-regulated potassium-efflux system protein KefC (K(+)/H(+)antiporter)	FS43_5650	Glutathione-regulated potassium-efflux system protein KefC (K(+)/H(+)antiporter)	FS44_17460	Glutathione-regulated potassium-efflux system protein KefC (K(+)/H(+)antiporter)	WQG_17600	Glutathione-regulated potassium-efflux system protein KefC (K(+)/H(+)antiporter)
4	**FS42_4980**	Adenylate cyclase	FS43_5670	Adenylate cyclase	FS44_17450	Adenylate cyclase	WQG_17580	Adenylate cyclase
4	**FS42_4990**	Glycerol kinase	FS43_5680	Glycerol kinase	FS44_17440	Glycerol kinase	WQG_17570	Glycerol kinase
4	**FS42_50**	Rare lipoprotein A	FS43_60	Rare lipoprotein A	FS44_23080	Rare lipoprotein A	WQG_22560	Rare lipoprotein A

4	**FS42_500**	50S ribosomal protein L1	FS43_520	50S ribosomal protein L1	FS44_22620	50S ribosomal protein L1	WQG_22100	50S ribosomal protein L1
4	**FS42_5000**	Glycerol uptake facilitator protein	FS43_5690	Glycerol uptake facilitator protein	FS44_17430	Glycerol uptake facilitator protein	WQG_17560	Glycerol uptake facilitator protein
4	**FS42_5010**	Anaerobic glycerol-3-phosphate dehydrogenase subunit C	FS43_6180	Anaerobic glycerol-3-phosphate dehydrogenase subunit C	FS44_17420	Anaerobic glycerol-3-phosphate dehydrogenase subunit C	WQG_17070	Anaerobic glycerol-3-phosphate dehydrogenase subunit C
4	**FS42_5020**	Anaerobic glycerol-3-phosphate dehydrogenase subunit B	FS43_6190	Anaerobic glycerol-3-phosphate dehydrogenase subunit B	FS44_17410	Anaerobic glycerol-3-phosphate dehydrogenase subunit B	WQG_17060	Anaerobic glycerol-3-phosphate dehydrogenase subunit B
4	**FS42_5030**	Anaerobic glycerol-3-phosphate dehydrogenase subunit A	FS43_6200	Anaerobic glycerol-3-phosphate dehydrogenase subunit A	FS44_17400	Anaerobic glycerol-3-phosphate dehydrogenase subunit A	WQG_17050	Anaerobic glycerol-3-phosphate dehydrogenase subunit A
4	**FS42_5040**	Glycerophosphoryl diester phosphodiesterase	FS43_6210	Glycerophosphoryl diester phosphodiesterase	FS44_17390	Glycerophosphoryl diester phosphodiesterase	WQG_17040	Glycerophosphoryl diester phosphodiesterase
4	**FS42_5050**	Glycerol-3-phosphate transporter	FS43_6220	Glycerol-3-phosphate transporter	FS44_17380	Glycerol-3-phosphate transporter	WQG_17030	Glycerol-3-phosphate transporter
4	**FS42_5060**	Ribulose-phosphate 3-epimerase	FS43_6230	Ribulose-phosphate 3-epimerase	FS44_17370	Ribulose-phosphate 3-epimerase	WQG_17020	Ribulose-phosphate 3-epimerase
4	**FS42_5070**	50S ribosomal protein L31 type B	FS43_6240	50S ribosomal protein L31 type B	FS44_17360	50S ribosomal protein L31 type B	WQG_17010	50S ribosomal protein L31 type B
4	**FS42_5080**	50S ribosomal protein L36	FS43_6250	50S ribosomal protein L36	FS44_17350	50S ribosomal protein L36	WQG_17000	50S ribosomal protein L36
4	**FS42_5090**	Ferredoxin-type protein NapF	FS43_6260	Ferredoxin-type protein NapF	FS44_17340	Ferredoxin-type protein NapF	WQG_16990	Ferredoxin-type protein NapF
4	**FS42_510**	50S ribosomal protein L10	FS43_530	50S ribosomal protein L10	FS44_22610	50S ribosomal protein L10	WQG_22090	50S ribosomal protein L10
4	**FS42_5100**	reductase	FS43_6270	reductase	FS44_17330	reductase	WQG_16980	reductase
4	**FS42_5110**	Periplasmic nitrate reductase	FS43_6280	Periplasmic nitrate reductase	FS44_17320	Periplasmic nitrate reductase	WQG_16970	Periplasmic nitrate reductase
4	**FS42_5120**	Ferredoxin-type protein napG	FS43_6290	Ferredoxin-type protein napG	FS44_17310	Ferredoxin-type protein napG	WQG_16960	Ferredoxin-type protein napG
4	**FS42_5130**	Ferredoxin-type protein, NapH/MauN	FS43_6300	Ferredoxin-type protein, NapH/MauN	FS44_17300	Ferredoxin-type protein, NapH/MauN	WQG_16950	Ferredoxin-type protein, NapH/MauN
4	**FS42_5140**	PeriplasmiC nitrate reductase, diheme cytochrome c subunit	FS43_6310	PeriplasmiC nitrate reductase, diheme cytochrome c subunit	FS44_17290	PeriplasmiC nitrate reductase, diheme cytochrome c subunit	WQG_16940	PeriplasmiC nitrate reductase, diheme cytochrome c subunit
4	**FS42_5150**	Cytochrome c-type protein NapC	FS43_6320	Cytochrome c-type protein NapC	FS44_17280	Cytochrome c-type protein NapC	WQG_16930	Cytochrome c-type protein NapC
4	**FS42_5160**	hypothetical protein	FS43_6330	hypothetical protein	FS44_17270	hypothetical protein	WQG_16920	hypothetical protein
4	**FS42_5170**	tRNA modification GTPase MnmE	FS43_6340	tRNA modification GTPase MnmE	FS44_17260	tRNA modification GTPase MnmE	WQG_16910	tRNA modification GTPase MnmE
4	**FS42_5180**	hypothetical protein	FS43_6350	hypothetical protein	FS44_17250	hypothetical protein	WQG_16900	hypothetical protein
4	**FS42_5190**	Methionine aminopeptidase	FS43_6360	Methionine aminopeptidase	FS44_17240	Methionine aminopeptidase	WQG_16890	Methionine aminopeptidase
4	**FS42_520**	50S ribosomal protein L7/L12	FS43_540	50S ribosomal protein L7/L12	FS44_22600	50S ribosomal protein L7/L12	WQG_22080	50S ribosomal protein L7/L12
4	**FS42_5200**	hypothetical protein	FS43_6370	hypothetical protein	FS44_17230	hypothetical protein	WQG_16880	hypothetical protein
4	**FS42_5210**	Ribosomal RNA small subunit methyltransferase I	FS43_6380	Ribosomal RNA small subunit methyltransferase I	FS44_17220	Ribosomal RNA small subunit methyltransferase I	WQG_16870	Ribosomal RNA small subunit methyltransferase I
4	**FS42_5220**	Penicillin-binding protein activator LpoA	FS43_6390	Penicillin-binding protein activator LpoA	FS44_17210	Penicillin-binding protein activator LpoA	WQG_16860	Penicillin-binding protein activator LpoA
4	**FS42_5230**	UPF0102 protein	FS43_6400	UPF0102 protein	FS44_17200	UPF0102 protein	WQG_16850	UPF0102 protein
4	**FS42_5240**	Phosphoheptose isomerase	FS43_6410	Phosphoheptose isomerase	FS44_17190	Phosphoheptose isomerase	WQG_16840	Phosphoheptose isomerase
4	**FS42_5250**	21 kDa hemolysin	FS43_6420	21 kDa hemolysin	FS44_17180	21 kDa hemolysin	WQG_16830	21 kDa hemolysin
4	**FS42_5260**	ABC transporter ATP-binding protein	FS43_6430	ABC transporter ATP-binding protein	FS44_17170	ABC transporter ATP-binding protein	WQG_16820	ABC transporter ATP-binding protein
4	**FS42_5270**	ABC transporter permease protein	FS43_6440	ABC transporter permease protein	FS44_17160	ABC transporter permease protein	WQG_16810	ABC transporter permease protein
4	**FS42_5280**	ABC-type transport system involved in resistance to organic solvent	FS43_6450	ABC-type transport system involved in resistance to organic solvent	FS44_17150	ABC-type transport system involved in resistance to organic solvent	WQG_16800	ABC-type transport system involved in resistance to organic solvent
4	**FS42_5290**	ABC-type transport system involved in resistance to organic solvent	FS43_6460	ABC-type transport system involved in resistance to organic solvent	FS44_17140	ABC-type transport system involved in resistance to organic solvent	WQG_16790	ABC-type transport system involved in resistance to organic solvent
4	**FS42_530**	DNA-directed RNA polymerase subunit beta	FS43_550	DNA-directed RNA polymerase subunit beta	FS44_22590	DNA-directed RNA polymerase subunit beta	WQG_22070	DNA-directed RNA polymerase subunit beta
4	**FS42_5300**	NTP binding protein (Contains STAS domain)	FS43_6470	NTP binding protein (Contains STAS domain)	FS44_17130	NTP binding protein (Contains STAS domain)	WQG_16780	NTP binding protein (Contains STAS domain)
4	**FS42_5310**	hypothetical protein	FS43_6480	hypothetical protein	FS44_17120	hypothetical protein	WQG_16770	hypothetical protein
4	**FS42_5320**	UDP-N-acetylglucosamine 1-carboxyvinyltransferase	FS43_6490	UDP-N-acetylglucosamine 1-carboxyvinyltransferase	FS44_17110	UDP-N-acetylglucosamine 1-carboxyvinyltransferase	WQG_16760	UDP-N-acetylglucosamine 1-carboxyvinyltransferase
4	**FS42_5330**	Cytochrome c-type biogenesis protein CcmE	FS43_6500	Cytochrome c-type biogenesis protein CcmE	FS44_17100	Cytochrome c-type biogenesis protein CcmE	WQG_16750	Cytochrome c-type biogenesis protein CcmE
4	**FS42_5340**	Cytochrome c-type biogenesis protein CcmF	FS43_6510	Cytochrome c-type biogenesis protein CcmF	FS44_17090	Cytochrome c-type biogenesis protein CcmF	WQG_16740	Cytochrome c-type biogenesis protein CcmF
4	**FS42_5350**	hypothetical protein	FS43_6520	hypothetical protein	FS44_17080	hypothetical protein	WQG_16730	hypothetical protein
4	**FS42_5360**	4-alpha-glucanotransferase	FS43_6530	4-alpha-glucanotransferase	FS44_17070	4-alpha-glucanotransferase	WQG_16720	4-alpha-glucanotransferase
4	**FS42_5370**	glycosyltransferase	FS43_6540	glycosyltransferase	FS44_17060	glycosyltransferase	WQG_16710	glycosyltransferase
4	**FS42_5380**	DNA topoisomerase 4 subunit B	FS43_6550	DNA topoisomerase 4 subunit B	FS44_17050	DNA topoisomerase 4 subunit B	WQG_16700	DNA topoisomerase 4 subunit B
4	**FS42_5390**	hypothetical protein	FS43_6560	hypothetical protein	FS44_17040	hypothetical protein	WQG_16690	hypothetical protein
4	**FS42_540**	DNA-directed RNA polymerase subunit beta'	FS43_560	DNA-directed RNA polymerase subunit beta'	FS44_22580	DNA-directed RNA polymerase subunit beta'	WQG_22060	DNA-directed RNA polymerase subunit beta'
4	**FS42_5420**	Transcriptional regulator, XRE	FS43_6590	Transcriptional regulator, XRE	FS44_17010	Transcriptional regulator, XRE	WQG_16660	Transcriptional regulator, XRE
4	**FS42_5430**	ABC transporter ATP-binding protein uup-1	FS43_6600	ABC transporter ATP-binding protein uup-1	FS44_17000	ABC transporter ATP-binding protein uup-1	WQG_16650	ABC transporter ATP-binding protein uup-1
4	**FS42_5440**	Thiamine pyrophosphokinase	FS43_6610	Thiamine pyrophosphokinase	FS44_16990	Thiamine pyrophosphokinase	WQG_16640	Thiamine pyrophosphokinase
4	**FS42_5450**	ThiF domain protein	FS43_6620	ThiF domain protein	FS44_16980	ThiF domain protein	WQG_16630	ThiF domain protein
4	**FS42_5460**	Transcriptional regulator	FS43_6630	Transcriptional regulator	FS44_16970	Transcriptional regulator	WQG_16620	Transcriptional regulator
4	**FS42_5470**	Membrane-fusion protein	FS43_6640	Membrane-fusion protein	FS44_16960	Membrane-fusion protein	WQG_16610	Membrane-fusion protein
4	**FS42_5480**	CcmA protein	FS43_6650	CcmA protein	FS44_16950	CcmA protein	WQG_16600	CcmA protein
4	**FS42_5490**	ABC-type multidrug transport system, permease component	FS43_6660	ABC-type multidrug transport system, permease component	FS44_16940	ABC-type multidrug transport system, permease component	WQG_16590	ABC-type multidrug transport system, permease component
4	**FS42_5500**	outer membrane protein	FS43_6670	outer membrane protein	FS44_16930	outer membrane protein	WQG_16580	outer membrane protein
4	**FS42_5510**	Cytosine deaminase	FS43_6680	Cytosine deaminase	FS44_16920	Cytosine deaminase	WQG_16570	Cytosine deaminase
4	**FS42_5520**	1,6-anhydro-N-acetylmuramyl-L-alanine amidase AmpD	FS43_6690	AmpD protein	FS44_16910	1,6-anhydro-N-acetylmuramyl-L-alanine amidase AmpD	WQG_16560	AmpD protein
4	**FS42_5530**	Protein involved in catabolism of external DNA	FS43_6700	Protein involved in catabolism of external DNA	FS44_16900	Protein involved in catabolism of external DNA	WQG_16550	Protein involved in catabolism of external DNA
4	**FS42_5540**	Orotate phosphoribosyltransferase	FS43_6710	Orotate phosphoribosyltransferase	FS44_16890	Orotate phosphoribosyltransferase	WQG_16540	Orotate phosphoribosyltransferase
4	**FS42_5550**	GlpG protein	FS43_6720	GlpG protein	FS44_16880	GlpG protein	WQG_16530	GlpG protein
4	**FS42_5560**	DSBA oxidoreductase	FS43_6730	DSBA oxidoreductase	FS44_16870	polyketide synthase PKS	WQG_16520	DSBA oxidoreductase
4	**FS42_5570**	hypothetical protein	FS43_6740	hypothetical protein	FS44_16860	hypothetical protein	WQG_16510	hypothetical protein
4	**FS42_5580**	Major facilitator superfamily MFS_1	FS43_6750	Major facilitator superfamily MFS_1	FS44_16840	Major facilitator superfamily MFS_1	WQG_16500	Major facilitator superfamily MFS_1
4	**FS42_5590**	Hemin import ATP-binding protein HmuV	FS43_6760	Hemin import ATP-binding protein HmuV	FS44_16830	FepC protein	WQG_16490	Hemin import ATP-binding protein HmuV
4	**FS42_560**	MFS family major facilitator transporter	FS43_590	MFS family major facilitator transporter	FS44_22560	MFS family major facilitator transporter	WQG_22040	MFS family major facilitator transporter
4	**FS42_5690**	Methionine synthase	FS43_6860	Methionine synthase	FS44_16800	Methionine synthase	WQG_16390	Methionine synthase
4	**FS42_570**	DNA-directed RNA polymerase subunit beta'	FS43_600	DNA-directed RNA polymerase subunit beta'	FS44_22550	DNA-directed RNA polymerase subunit beta'	WQG_22030	DNA-directed RNA polymerase subunit beta'
4	**FS42_5700**	Aldose 1-epimerase	FS43_6870	Aldose 1-epimerase	FS44_16790	Aldose 1-epimerase	WQG_16380	Aldose 1-epimerase
4	**FS42_5710**	Uroporphyrinogen decarboxylase	FS43_6880	Uroporphyrinogen decarboxylase	FS44_16780	Uroporphyrinogen decarboxylase	WQG_16370	Uroporphyrinogen decarboxylase
4	**FS42_5720**	hypothetical protein	FS43_6890	hypothetical protein	FS44_16770	hypothetical protein	WQG_16360	hypothetical protein
4	**FS42_5730**	DNA-binding protein HU	FS43_6900	DNA-binding protein HU	FS44_16760	DNA-binding protein HU	WQG_16350	DNA-binding protein HU
4	**FS42_5740**	Peptide chain release factor 1	FS43_6910	Peptide chain release factor 1	FS44_16750	Peptide chain release factor 1	WQG_16340	Peptide chain release factor 1
4	**FS42_5750**	Release factor glutamine methyltransferase	FS43_6920	Release factor glutamine methyltransferase	FS44_16740	Release factor glutamine methyltransferase	WQG_16320	Release factor glutamine methyltransferase
4	**FS42_5760**	hypothetical protein	FS43_6930	hypothetical protein	FS44_16730	hypothetical protein	WQG_16310	hypothetical protein
4	**FS42_5770**	2-dehydro-3-deoxyphosphooctonate aldolase	FS43_6940	2-dehydro-3-deoxyphosphooctonate aldolase	FS44_16720	2-dehydro-3-deoxyphosphooctonate aldolase	WQG_16300	2-dehydro-3-deoxyphosphooctonate aldolase
4	**FS42_5780**	2-hydroxyacid dehydrogenase	FS43_6950	2-hydroxyacid dehydrogenase	FS44_16710	2-hydroxyacid dehydrogenase	WQG_16290	2-hydroxyacid dehydrogenase
4	**FS42_5790**	Leukotoxin-activating lysine-acyltransferase lktC serotype A1	FS43_6960	Leukotoxin-activating lysine-acyltransferase lktC serotype A1	FS44_16700	Leukotoxin-activating lysine-acyltransferase lktC serotype A1	WQG_16280	Leukotoxin-activating lysine-acyltransferase lktC serotype A1
4	**FS42_580**	hypothetical protein	FS43_610	hypothetical protein	FS44_22540	hypothetical protein	WQG_22020	hypothetical protein
4	**FS42_5800**	Leukotoxin	FS43_6970	Leukotoxin	FS44_16690	Leukotoxin	WQG_16270	Leukotoxin
4	**FS42_5810**	Alpha-hemolysin translocation ATP-binding protein HlyB	FS43_6980	Alpha-hemolysin translocation ATP-binding protein HlyB	FS44_16680	Alpha-hemolysin translocation ATP-binding protein HlyB	WQG_16260	Alpha-hemolysin translocation ATP-binding protein HlyB
4	**FS42_5820**	RTX-III toxin determinant D	FS43_6990	RTX-III toxin determinant D	FS44_16670	RTX-III toxin determinant D	WQG_16250	RTX-III toxin determinant D
4	**FS42_5830**	CP4-6 prophage	FS43_7000	CP4-6 prophage	FS44_16660	CP4-6 prophage	WQG_16240	prophage protein
4	**FS42_5840**	Lipoprotein-releasing system transmembrane protein lolC	FS43_7010	Lipoprotein-releasing system transmembrane protein lolC	FS44_16630	Lipoprotein-releasing system transmembrane protein lolC	WQG_16230	Lipoprotein-releasing system transmembrane protein lolC
4	**FS42_5850**	reductase	FS43_7020	reductase	FS44_16620	reductase	WQG_16220	reductase
4	**FS42_5860**	Component of anaerobic dehydrogenase	FS43_7030	Component of anaerobic dehydrogenase	FS44_16610	Component of anaerobic dehydrogenase	WQG_16210	Component of anaerobic dehydrogenase
4	**FS42_5870**	endonuclease 4	FS43_7040	endonuclease 4	FS44_16600	endonuclease 4	WQG_16200	endonuclease 4
4	**FS42_5880**	C40 family peptidase/lipoprotein	FS43_7050	C40 family peptidase/lipoprotein	FS44_16590	C40 family peptidase/lipoprotein	WQG_16190	C40 family peptidase/lipoprotein
4	**FS42_5890**	SOS-response transcriptional repressors/RecA-mediated autopeptidase	FS43_7060	SOS-response transcriptional repressors/RecA-mediated autopeptidase	FS44_16580	SOS-response transcriptional repressors/RecA-mediated autopeptidase	WQG_16180	SOS-response transcriptional repressors/RecA-mediated autopeptidase
4	**FS42_590**	3-oxoacyl-[acyl-carrier-protein[synthase (Beta-ketoacyl-ACP synthase)	FS43_620	3-oxoacyl-[acyl-carrier-protein[synthase (Beta-ketoacyl-ACP synthase)	FS44_22530	3-oxoacyl-[acyl-carrier-protein[synthase (Beta-ketoacyl-ACP synthase)	WQG_22010	3-oxoacyl-[acyl-carrier-protein] synthase (Beta-ketoacyl-ACP synthase)
4	**FS42_5900**	Peptidase B	FS43_7070	Peptidase B	FS44_16570	Peptidase B	WQG_16170	Peptidase B
4	**FS42_5910**	Membrane-fusion protein	FS43_7080	Membrane-fusion protein	FS44_16560	Membrane-fusion protein	WQG_16160	Membrane-fusion protein
4	**FS42_5920**	hypothetical protein	FS43_7090	hypothetical protein	FS44_16550	hypothetical protein	WQG_16150	hypothetical protein
4	**FS42_5930**	2-isopropylmalate synthase	FS43_7100	2-isopropylmalate synthase	FS44_16540	2-isopropylmalate synthase	WQG_16140	2-isopropylmalate synthase
4	**FS42_5940**	Exodeoxyribonuclease V beta chain	FS43_7110	Exodeoxyribonuclease V beta chain	FS44_16530	Exodeoxyribonuclease V beta chain	WQG_16130	Exodeoxyribonuclease V beta chain
4	**FS42_5950**	ATP-dependent DNA helicase rep	FS43_7130	ATP-dependent DNA helicase rep	FS44_16520	ATP-dependent DNA helicase rep	WQG_16110	ATP-dependent DNA helicase rep
4	**FS42_5960**	microcin-H47 secretion/processing ATP-binding protein mchF	FS43_7140	microcin-H47 secretion/processing ATP-binding protein mchF	FS44_16510	microcin-H47 secretion/processing ATP-binding protein mchF	WQG_16100	microcin-H47 secretion/processing ATP-binding protein mchF
4	**FS42_5970**	Cupin 4	FS43_7150	Cupin 4	FS44_16500	Cupin 4	WQG_16090	Cupin 4
4	**FS42_5980**	hypothetical protein	FS43_7160	hypothetical protein	FS44_16490	hypothetical protein	WQG_16080	hypothetical protein
4	**FS42_60**	D-alanyl-D-alanine carboxypeptidase dacA	FS43_70	D-alanyl-D-alanine carboxypeptidase dacA	FS44_23070	D-alanyl-D-alanine carboxypeptidase dacA	WQG_22550	D-alanyl-D-alanine carboxypeptidase dacA
4	**FS42_600**	Thioester dehydrase	FS43_630	Thioester dehydrase	FS44_22520	Thioester dehydrase	WQG_22000	Thioester dehydrase
4	**FS42_6000**	Secretion permease	FS43_7170	Secretion permease	FS44_16450	Secretion permease	WQG_16060	Secretion permease
4	**FS42_6020**	N-acetylmuramoyl-L-alanine amidase	FS43_7190	N-acetylmuramoyl-L-alanine amidase	FS44_16420	N-acetylmuramoyl-L-alanine amidase	WQG_16040	N-acetylmuramoyl-L-alanine amidase
4	**FS42_6030**	ATPase or kinase	FS43_7200	ATPase or kinase	FS44_16410	ATPase or kinase	WQG_16030	ATPase or kinase
4	**FS42_6040**	Oligoribonuclease	FS43_7210	Oligoribonuclease	FS44_16400	Oligoribonuclease	WQG_16020	Oligoribonuclease
4	**FS42_6050**	Phosphopantetheine adenylyltransferase	FS43_7220	Phosphopantetheine adenylyltransferase	FS44_16390	Phosphopantetheine adenylyltransferase	WQG_16010	Phosphopantetheine adenylyltransferase
4	**FS42_6060**	3-deoxy-D-manno-octulosonic-acid transferase	FS43_7230	3-deoxy-D-manno-octulosonic-acid transferase	FS44_16380	3-deoxy-D-manno-octulosonic-acid transferase	WQG_16000	3-deoxy-D-manno-octulosonic-acid transferase
4	**FS42_6070**	hypothetical protein	FS43_7250	hypothetical protein	FS44_16370	hypothetical protein	WQG_15990	hypothetical protein
4	**FS42_6080**	DNA polymerase III subunit alpha	FS43_7260	DNA polymerase III subunit alpha	FS44_16360	DNA polymerase III subunit alpha	WQG_15980	DNA polymerase III subunit alpha
4	**FS42_6090**	Lipopolysaccharide export system permease protein lptG	FS43_7270	Lipopolysaccharide export system permease protein lptG	FS44_16350	Lipopolysaccharide export system permease protein lptG	WQG_15970	Lipopolysaccharide export system permease protein lptG
4	**FS42_610**	reductase	FS43_640	reductase	FS44_22500	reductase	WQG_21990	reductase
4	**FS42_6100**	Lipopolysaccharide export system permease protein lptF	FS43_7280	Lipopolysaccharide export system permease protein lptF	FS44_16340	Lipopolysaccharide export system permease protein lptF	WQG_15960	Lipopolysaccharide export system permease protein lptF
4	**FS42_6110**	hypothetical protein	FS43_7290	hypothetical protein	FS44_16330	hypothetical protein	WQG_15950	hypothetical protein
4	**FS42_6120**	Cytosol aminopeptidase	FS43_7300	Cytosol aminopeptidase	FS44_16320	Cytosol aminopeptidase	WQG_15940	Cytosol aminopeptidase
4	**FS42_6130**	hypothetical protein	FS43_7310	hypothetical protein	FS44_16310	hypothetical protein	WQG_15930	hypothetical protein
4	**FS42_6140**	DNA ligase	FS43_7320	DNA ligase	FS44_16300	DNA ligase	WQG_15920	DNA ligase
4	**FS42_6150**	hydrolase	FS43_7330	hydrolase	FS44_16290	hydrolase	WQG_15910	hydrolase
4	**FS42_6160**	hypothetical protein	FS43_7340	hypothetical protein	FS44_16280	hypothetical protein	WQG_15900	hypothetical protein
4	**FS42_6170**	hypothetical protein	FS43_7350	hypothetical protein	FS44_16270	hypothetical protein	WQG_15890	hypothetical protein
4	**FS42_6180**	membrane protein	FS43_7360	membrane protein	FS44_16260	membrane protein	WQG_15880	membrane protein
4	**FS42_6190**	hypothetical protein	FS43_7370	hypothetical protein	FS44_16250	hypothetical protein	WQG_15870	hypothetical protein
4	**FS42_620**	Beta-ketoacyl synthase	FS43_650	Beta-ketoacyl synthase	FS44_22490	Beta-ketoacyl synthase	WQG_21980	Beta-ketoacyl synthase
4	**FS42_6200**	Oxaloacetate decarboxylase gamma chain	FS43_7380	Oxaloacetate decarboxylase gamma chain	FS44_16240	Oxaloacetate decarboxylase gamma chain	WQG_15860	Oxaloacetate decarboxylase gamma chain
4	**FS42_6210**	Oxaloacetate decarboxylase alpha chain	FS43_7390	Oxaloacetate decarboxylase alpha chain	FS44_16230	Oxaloacetate decarboxylase alpha chain	WQG_15850	Oxaloacetate decarboxylase alpha chain
4	**FS42_6220**	oxaloacetate decarboxylase beta chain	FS43_7400	oxaloacetate decarboxylase beta chain	FS44_16220	oxaloacetate decarboxylase beta chain	WQG_15840	oxaloacetate decarboxylase beta chain
4	**FS42_6230**	hypothetical protein	FS43_7410	hypothetical protein	FS44_16200	hypothetical protein	WQG_15820	hypothetical protein
4	**FS42_6240**	Protein phnA	FS43_7420	Protein phnA	FS44_16210	Protein phnA	WQG_15830	Protein phnA
4	**FS42_6250**	LysR protein	FS43_7430	LysR protein	FS44_16190	LysR protein	WQG_15810	LysR protein
4	**FS42_6260**	hypothetical protein	FS43_7440	hypothetical protein	FS44_16180	hypothetical protein	WQG_15800	hypothetical protein
4	**FS42_6270**	Bile acid 7-alpha-dehydratase	FS43_7450	Bile acid 7-alpha-dehydratase	FS44_16140	Bile acid 7-alpha-dehydratase	WQG_15790	Bile acid 7-alpha-dehydratase
4	**FS42_6280**	Thiol:disulfide interchange protein DsbD	FS43_7460	Thiol:disulfide interchange protein DsbD	FS44_16130	Thiol:disulfide interchange protein DsbD	WQG_15780	Thiol:disulfide interchange protein DsbD
4	**FS42_6290**	Arginine transport ATP-binding protein ArtP	FS43_7470	Arginine transport ATP-binding protein ArtP	FS44_16120	Glutamine transport ATP-binding protein GlnQ	WQG_15770	Arginine transport ATP-binding protein ArtP
4	**FS42_630**	hypothetical protein	FS43_660	hypothetical protein	FS44_22480	hypothetical protein	WQG_21970	hypothetical protein
4	**FS42_6300**	ABC transporter arginine-binding protein	FS43_7480	ABC transporter arginine-binding protein	FS44_16110	ABC transporter arginine-binding protein	WQG_15760	ABC transporter arginine-binding protein
4	**FS42_6310**	Arginine ABC transporter permease protein ArtQ	FS43_7490	Arginine ABC transporter permease protein ArtQ	FS44_16100	Arginine ABC transporter permease protein ArtQ	WQG_15750	Arginine ABC transporter permease protein ArtQ
4	**FS42_6320**	Arginine ABC transporter permease protein ArtM	FS43_7500	Arginine ABC transporter permease protein ArtM	FS44_16090	Arginine ABC transporter permease protein ArtM	WQG_15740	Arginine ABC transporter permease protein ArtM
4	**FS42_6330**	hypothetical protein	FS43_7510	hypothetical protein	FS44_16080	hypothetical protein	WQG_15730	hypothetical protein
4	**FS42_6340**	Transcriptional regulator, AraC	FS43_7520	Transcriptional regulator, AraC	FS44_16070	Transcriptional regulator, AraC	WQG_15720	Transcriptional regulator, AraC
4	**FS42_6350**	Molybdate ABC transporter permease protein	FS43_7530	Molybdate ABC transporter permease protein	FS44_16060	Molybdate ABC transporter permease protein	WQG_15710	Molybdate ABC transporter permease protein
4	**FS42_6360**	N-acylneuraminate cytidylyltransferase	FS43_7540	N-acylneuraminate cytidylyltransferase	FS44_16050	N-acylneuraminate cytidylyltransferase	WQG_15700	N-acylneuraminate cytidylyltransferase
4	**FS42_6370**	hypothetical protein	FS43_7550	hypothetical protein	FS44_16040	hypothetical protein	WQG_15690	hypothetical protein
4	**FS42_6380**	CMP-N-acetylneuraminate-beta-galactosamide- alpha-2, 3-sialyltransferase	FS43_7560	CMP-N-acetylneuraminate-beta-galactosamide- alpha-2, 3-sialyltransferase	FS44_16030	CMP-N-acetylneuraminate-beta-galactosamide- alpha-2, 3-sialyltransferase	WQG_15680	CMP-N-acetylneuraminate-beta-galactosamide- alpha-2, 3-sialyltransferase

4	FS42_6390	Lsg locus protein 4	FS43_7570	Lsg locus protein 4	FS44_16020	Lsg locus protein 4	WQG_15670	Lsg locus protein 4
4	FS42_640	hypothetical protein	FS43_670	hypothetical protein	FS44_22470	hypothetical protein	WQG_21960	hypothetical protein
4	FS42_6400	Membrane protein	FS43_7580	Membrane protein	FS44_16010	Membrane protein	WQG_15660	Membrane protein
4	FS42_6410	glycosyltransferase	FS43_7590	glycosyltransferase	FS44_16000	glycosyltransferase	WQG_15650	glycosyltransferase
4	FS42_6420	glycosyltransferase	FS43_7600	glycosyltransferase	FS44_15990	glycosyltransferase	WQG_15640	glycosyltransferase
4	FS42_6430	MerTP family mercury (Hg2+) permease, binding protein MerP	FS43_7610	MerTP family mercury (Hg2+) permease, binding protein MerP	FS44_15980	MerTP family mercury (Hg2+) permease, binding protein MerP	WQG_15630	MerTP family mercury (Hg2+) permease, binding protein MerP
4	FS42_6440	transport protein	FS43_7620	transport protein	FS44_15970	transport protein	WQG_15620	transport protein
4	FS42_6450	hypothetical protein	FS43_7630	hypothetical protein	FS44_15960	hypothetical protein	WQG_15610	hypothetical protein
4	FS42_6460	DNA polymerase III subunit delta	FS43_7640	DNA polymerase III subunit delta	FS44_15950	DNA polymerase III subunit delta	WQG_15600	DNA polymerase III subunit delta
4	FS42_6470	rare lipoprotein B	FS43_7650	rare lipoprotein B	FS44_15940	rare lipoprotein B	WQG_15590	rare lipoprotein B
4	FS42_6480	Leucyl-tRNA synthetase	FS43_7660	Leucyl-tRNA synthetase	FS44_15930	Leucyl-tRNA synthetase	WQG_15580	Leucyl-tRNA synthetase
4	FS42_6490	Threonyl-tRNA synthetase	FS43_7670	Threonyl-tRNA synthetase	FS44_15920	Threonyl-tRNA synthetase	WQG_15570	Threonyl-tRNA synthetase
4	FS42_650	Phospholipid/glycerol acyltransferase	FS43_680	Phospholipid/glycerol acyltransferase	FS44_22460	Phospholipid/glycerol acyltransferase	WQG_21950	Phospholipid/glycerol acyltransferase
4	FS42_6510	Translation initiation factor IF-3	FS43_7690	Translation initiation factor IF-3	FS44_15910	Translation initiation factor IF-3	WQG_15550	Translation initiation factor IF-3
4	FS42_6520	50S ribosomal protein L35	FS43_7700	50S ribosomal protein L35	FS44_15900	50S ribosomal protein L35	WQG_15540	50S ribosomal protein L35
4	FS42_6530	50S ribosomal protein L20	FS43_7710	50S ribosomal protein L20	FS44_15890	50S ribosomal protein L20	WQG_15530	50S ribosomal protein L20
4	FS42_6540	hypothetical protein	FS43_7720	hypothetical protein	FS44_15880	hypothetical protein	WQG_15520	hypothetical protein
4	FS42_6550	hypothetical protein	FS43_7730	hypothetical protein	FS44_15870	hypothetical protein	WQG_15510	hypothetical protein
4	FS42_6560	ABC-transporter, ATP-binding protein	FS43_7740	ABC-transporter, ATP-binding protein	FS44_15860	ABC-transporter, ATP-binding protein	WQG_15500	ABC-transporter, ATP-binding protein
4	FS42_6570	ABC transporter, permease protein	FS43_7750	ABC transporter, permease protein	FS44_15850	ABC transporter, permease protein	WQG_15490	ABC transporter, permease protein
4	FS42_6580	L-lactate dehydrogenase	FS43_7760	L-lactate dehydrogenase	FS44_15840	L-lactate dehydrogenase	WQG_15480	L-lactate dehydrogenase
4	FS42_6590	SecF	FS43_7770	SecF	FS44_15830	SecF	WQG_15470	SecF
4	FS42_660	Acyl carrier protein	FS43_690	Acyl carrier protein	FS44_22450	Acyl carrier protein	WQG_21940	Acyl carrier protein
4	FS42_6600	Protein translocase subunit SecD	FS43_7780	Protein translocase subunit SecD	FS44_15820	Protein translocase subunit SecD	WQG_15460	Protein translocase subunit SecD
4	FS42_6610	UPF0092 membrane protein	FS43_7790	UPF0092 membrane protein	FS44_15810	UPF0092 membrane protein	WQG_15450	UPF0092 membrane protein
4	FS42_6620	hypothetical protein	FS43_7800	hypothetical protein	FS44_15800	hypothetical protein	WQG_15440	hypothetical protein
4	FS42_6630	Glycine cleavage system transcriptional activator	FS43_7810	Glycine cleavage system transcriptional activator	FS44_15790	Glycine cleavage system transcriptional activator	WQG_15430	Glycine cleavage system transcriptional activator
4	FS42_6640	hypothetical protein	FS43_7820	hypothetical protein	FS44_15780	hypothetical protein	WQG_15420	hypothetical protein
4	FS42_6650	Branched-chain-amino-acid aminotransferase	FS43_7830	Branched-chain-amino-acid aminotransferase	FS44_15770	Branched-chain-amino-acid aminotransferase	WQG_15410	Branched-chain-amino-acid aminotransferase
4	FS42_6660	RutC	FS43_7840	RutC	FS44_15760	RutC	WQG_15400	RutC
4	FS42_6670	HAD superfamily haloacid dehalogenase hydrolase	FS43_7850	HAD superfamily haloacid dehalogenase hydrolase	FS44_15750	HAD superfamily haloacid dehalogenase hydrolase	WQG_15390	HAD superfamily haloacid dehalogenase hydrolase
4	FS42_6680	HTH-type transcriptional regulator CysB	FS43_7860	HTH-type transcriptional regulator CysB	FS44_15740	HTH-type transcriptional regulator CysB	WQG_15380	HTH-type transcriptional regulator CysB
4	FS42_6690	outer membrane protein	FS43_7870	outer membrane protein	FS44_15730	outer membrane protein	WQG_15370	outer membrane protein
4	FS42_670	hypothetical protein	FS43_700	hypothetical protein	FS44_22440	hypothetical protein	WQG_21930	hypothetical protein
4	FS42_6700	Thioredoxin	FS43_7880	Thioredoxin	FS44_15720	Thioredoxin	WQG_15360	Thioredoxin
4	FS42_6710	Uracil-xanthine permease	FS43_7890	Uracil-xanthine permease	FS44_15710	Uracil-xanthine permease	WQG_15350	Uracil-xanthine permease
4	FS42_6720	Cation transport ATPase	FS43_7900	Cation transport ATPase	FS44_15700	Cation transport ATPase	WQG_15340	Cation transport ATPase
4	FS42_6730	Copper-transporting P-type ATPase	FS43_7920	Copper-transporting P-type ATPase	FS44_15690	Copper-transporting P-type ATPase	WQG_15330	Copper-transporting P-type ATPase
4	FS42_6740	Periplasmic chelated iron-binding protein yfeA	FS43_7930	Periplasmic chelated iron-binding protein yfeA	FS44_15680	Periplasmic chelated iron-binding protein yfeA	WQG_15320	Periplasmic chelated iron-binding protein yfeA
4	FS42_6750	iron transport system ATP-binding protein	FS43_7940	iron transport system ATP-binding protein	FS44_15670	iron transport system ATP-binding protein	WQG_15310	iron transport system ATP-binding protein
4	FS42_6760	iron transport system membrane protein	FS43_7960	iron transport system membrane protein	FS44_15660	iron transport system membrane protein	WQG_15300	iron transport system membrane protein
4	FS42_6770	Chelated iron transport system membrane protein yfeD	FS43_7970	Chelated iron transport system membrane protein yfeD	FS44_15650	Chelated iron transport system membrane protein yfeD	WQG_15290	Chelated iron transport system membrane protein yfeD
4	FS42_6780	transcriptional regulator	FS43_7980	transcriptional regulator	FS44_15640	transcriptional regulator	WQG_15280	transcriptional regulator
4	FS42_6790	Bifunctional phosphoribosylaminoimidazolecarboxamide formyltransferase/IMP cyclohydrolase	FS43_7990	Bifunctional phosphoribosylaminoimidazolecarboxamide formyltransferase/IMP cyclohydrolase	FS44_15630	Bifunctional phosphoribosylaminoimidazolecarboxamide formyltransferase/IMP cyclohydrolase	WQG_15270	Bifunctional phosphoribosylaminoimidazolecarboxamide formyltransferase/IMP cyclohydrolase
4	FS42_680	AMP-binding enzyme	FS43_710	AMP-binding enzyme	FS44_22430	AMP-binding enzyme	WQG_21920	AMP-binding enzyme
4	FS42_6800	Tellurium resistance protein	FS43_8000	Tellurium resistance protein	FS44_15620	Tellurium resistance protein	WQG_15260	Tellurium resistance protein
4	FS42_6810	ribosome biogenesis GTPase RsgA	FS43_8010	ribosome biogenesis GTPase RsgA	FS44_15610	ribosome biogenesis GTPase RsgA	WQG_15250	ribosome biogenesis GTPase RsgA
4	FS42_6820	Sulfate transporter	FS43_8020	Sulfate transporter	FS44_15600	Sulfate transporter	WQG_15240	Sulfate transporter
4	FS42_6830	Exodeoxyribonuclease V, alpha subunit	FS43_8030	Exodeoxyribonuclease V, alpha subunit	FS44_15590	Exodeoxyribonuclease V, alpha subunit	WQG_15230	Exodeoxyribonuclease V, alpha subunit
4	FS42_6840	Pyruvate kinase	FS43_8040	Pyruvate kinase	FS44_15580	Pyruvate kinase	WQG_15220	Pyruvate kinase
4	FS42_6850	phosphohistidine phosphatase	FS43_8050	phosphohistidine phosphatase	FS44_15570	phosphohistidine phosphatase	WQG_15210	phosphohistidine phosphatase
4	FS42_6860	Homoserine kinase	FS43_8070	Homoserine kinase	FS44_15560	Homoserine kinase	WQG_15200	Homoserine kinase
4	FS42_6870	Bifunctional aspartokinase/homoserine dehydrogenase	FS43_8080	Bifunctional aspartokinase/homoserine dehydrogenase	FS44_15550	Bifunctional aspartokinase/homoserine dehydrogenase	WQG_15190	Bifunctional aspartokinase/homoserine dehydrogenase
4	FS42_6880	Autotransporter adhesin	FS43_8090	Autotransporter adhesin	FS44_15520	hypothetical protein	WQG_15180	Autotransporter adhesin
4	FS42_6890	Superoxide dismutase	FS43_8100	Superoxide dismutase	FS44_15510	Superoxide dismutase	WQG_15170	Superoxide dismutase
4	FS42_690	Integral membrane protein	FS43_720	Integral membrane protein	FS44_22420	Integral membrane protein	WQG_21910	Integral membrane protein
4	FS42_6900	Uracil phosphoribosyltransferase	FS43_8110	Uracil phosphoribosyltransferase	FS44_15500	Uracil phosphoribosyltransferase	WQG_15160	Uracil phosphoribosyltransferase
4	FS42_6910	HTH-type transcriptional activator ilvY	FS43_8120	HTH-type transcriptional activator ilvY	FS44_15490	HTH-type transcriptional activator ilvY	WQG_15150	HTH-type transcriptional activator ilvY
4	FS42_6920	50S ribosomal protein L31	FS43_8130	50S ribosomal protein L31	FS44_15480	50S ribosomal protein L31	WQG_15140	50S ribosomal protein L31
4	FS42_6930	Transketolase 2	FS43_8140	Transketolase 2	FS44_15470	Transketolase 2	WQG_15130	Transketolase 2
4	FS42_6940	Holliday junction resolvase	FS43_8150	Holliday junction resolvase	FS44_15460	Holliday junction resolvase	WQG_15120	Holliday junction resolvase
4	FS42_6950	hypothetical protein	FS43_8160	hypothetical protein	FS44_15450	hypothetical protein	WQG_15110	hypothetical protein
4	FS42_6960	Ribosomal RNA small subunit methyltransferase E	FS43_8170	Ribosomal RNA small subunit methyltransferase E	FS44_15440	Ribosomal RNA small subunit methyltransferase E	WQG_15100	Ribosomal RNA small subunit methyltransferase E
4	FS42_6970	23S rRNA pseudouridine synthase D	FS43_8180	23S rRNA pseudouridine synthase D	FS44_15430	23S rRNA pseudouridine synthase D	WQG_15090	23S rRNA pseudouridine synthase D
4	FS42_6980	Transcriptional regulator, BolA protein	FS43_8190	Transcriptional regulator, BolA protein	FS44_15420	Transcriptional regulator, BolA protein	WQG_15080	Transcriptional regulator, BolA protein
4	FS42_6990	Lipoprotein	FS43_8200	Lipoprotein	FS44_15410	Lipoprotein	WQG_15070	Lipoprotein
4	FS42_70	hypothetical protein	FS43_80	hypothetical protein	FS44_23060	hypothetical protein	WQG_22540	hypothetical protein
4	FS42_700	acyl-coenzyme A synthetase-related protein	FS43_730	acyl-coenzyme A synthetase-related protein	FS44_22410	acyl-coenzyme A synthetase-related protein	WQG_21900	Conserved acyl-coenzyme A synthetase-related protein
4	FS42_7000	hypothetical protein	FS43_8210	hypothetical protein	FS44_15400	hypothetical protein	WQG_15060	hypothetical protein
4	FS42_7010	hypothetical protein	FS43_8220	hypothetical protein	FS44_15390	hypothetical protein	WQG_15050	hypothetical protein
4	FS42_7020	Protein translocase subunit SecA	FS43_8230	Protein translocase subunit SecA	FS44_15380	Protein translocase subunit SecA	WQG_15040	Protein translocase subunit SecA
4	FS42_7030	Mutator mutT protein	FS43_8240	Mutator mutT protein	FS44_15370	Mutator mutT protein	WQG_15030	Mutator mutT protein
4	FS42_7040	Anthranilate synthase component I	FS43_8250	Anthranilate synthase component I	FS44_15360	Anthranilate synthase component I	WQG_15020	Anthranilate synthase component I
4	FS42_7050	Anthranilate synthase component II	FS43_8270	Anthranilate synthase component II	FS44_15350	Anthranilate synthase component II	WQG_15010	Anthranilate synthase component II
4	FS42_7060	Anthranilate phosphoribosyltransferase	FS43_8280	Anthranilate phosphoribosyltransferase	FS44_15340	Anthranilate phosphoribosyltransferase	WQG_15000	Anthranilate phosphoribosyltransferase
4	FS42_7070	Anthranilate phosphoribosyltransferase	FS43_8290	Anthranilate phosphoribosyltransferase	FS44_15330	Anthranilate phosphoribosyltransferase	WQG_14990	Anthranilate phosphoribosyltransferase
4	FS42_7080	Inner membrane protein YedI	FS43_8300	Inner membrane protein YedI	FS44_15320	Inner membrane protein YedI	WQG_14980	Inner membrane protein YedI
4	FS42_7090	hypothetical protein	FS43_8310	hypothetical protein	FS44_15310	hypothetical protein	WQG_14970	hypothetical protein
4	FS42_710	Glycosyl transferase family 2	FS43_740	Glycosyl transferase family 2	FS44_22400	Glycosyl transferase family 2	WQG_21890	Glycosyl transferase family 2
4	FS42_7100	Periplasmic oligopeptide-binding protein	FS43_8320	Periplasmic oligopeptide-binding protein	FS44_15290	Periplasmic oligopeptide-binding protein	WQG_14960	Periplasmic oligopeptide-binding protein
4	FS42_7110	ABC-type oligopeptide transport system	FS43_8330	ABC-type oligopeptide transport system	FS44_15280	ABC-type oligopeptide transport system	WQG_14950	ABC-type oligopeptide transport system
4	FS42_7120	Binding-protein-dependent transport system inner membrane component	FS43_8340	Binding-protein-dependent transport system inner membrane component	FS44_15270	Binding-protein-dependent transport system inner membrane component	WQG_14940	Binding-protein-dependent transport system inner membrane component
4	FS42_7130	ABC transporter, ATP-binding protein	FS43_8350	ABC transporter, ATP-binding protein	FS44_15260	ABC transporter, ATP-binding protein	WQG_14930	ABC transporter, ATP-binding protein
4	FS42_7140	Glycerol-3-phosphate acyltransferase	FS43_8360	Glycerol-3-phosphate acyltransferase	FS44_15250	Glycerol-3-phosphate acyltransferase	WQG_14920	Glycerol-3-phosphate acyltransferase
4	FS42_7150	LexA repressor	FS43_8370	LexA repressor	FS44_15240	LexA repressor	WQG_14910	LexA repressor
4	FS42_7160	Protein CyaY	FS43_8380	Protein CyaY	FS44_15230	Protein CyaY	WQG_14900	Protein CyaY
4	FS42_7170	ATP-dependent DNA helicase recQ	FS43_8390	ATP-dependent DNA helicase recQ	FS44_15220	ATP-dependent DNA helicase recQ	WQG_14890	ATP-dependent DNA helicase recQ
4	FS42_7180	glycosyltransferase	FS43_8400	glycosyltransferase	FS44_15210	glycosyltransferase	WQG_14880	glycosyltransferase
4	FS42_7190	hypothetical protein	FS43_8410	hypothetical protein	FS44_15200	hypothetical protein	WQG_14870	hypothetical protein
4	FS42_720	Lipid A biosynthesis acyltransferase	FS43_750	Lipid A biosynthesis acyltransferase	FS44_22390	Lipid A biosynthesis acyltransferase	WQG_21880	Lipid A biosynthesis acyltransferase
4	FS42_7200	Enolase	FS43_8420	Enolase	FS44_15190	Enolase	WQG_14860	Enolase
4	FS42_7210	Lipoprotein releasing system, ATP-binding protein	FS43_8430	Lipoprotein releasing system, ATP-binding protein	FS44_15180	Lipoprotein releasing system, ATP-binding protein	WQG_14850	Lipoprotein releasing system, ATP-binding protein
4	FS42_7220	Lipoprotein-releasing system transmembrane protein lolC	FS43_8440	Lipoprotein-releasing system transmembrane protein lolC	FS44_15170	Lipoprotein-releasing system transmembrane protein lolC	WQG_14840	Lipoprotein-releasing system transmembrane protein lolC
4	FS42_7230	hypothetical protein	FS43_8450	hypothetical protein	FS44_15160	hypothetical protein	WQG_14830	hypothetical protein
4	FS42_7240	Chaperone protein htpG	FS43_8460	Chaperone protein htpG	FS44_15150	Chaperone protein htpG	WQG_14820	Chaperone protein htpG
4	FS42_7250	HI0074 family nucleotidyltransferase substrate binding protein	FS43_8470	HI0074 family nucleotidyltransferase substrate binding protein	FS44_15140	HI0074 family nucleotidyltransferase substrate binding protein	WQG_14810	HI0074 family nucleotidyltransferase substrate binding protein
4	FS42_7270	phosphohydrolase	FS43_8490	phosphohydrolase	FS44_15130	phosphohydrolase	WQG_14790	phosphohydrolase
4	FS42_7280	2-amino-4-hydroxy-6- hydroxymethyldihydropteridine pyrophosphokinase	FS43_8500	2-amino-4-hydroxy-6- hydroxymethyldihydropteridine pyrophosphokinase	FS44_15120	2-amino-4-hydroxy-6- hydroxymethyldihydropteridine pyrophosphokinase	WQG_14780	2-amino-4-hydroxy-6- hydroxymethyldihydropteridine pyrophosphokinase
4	FS42_7290	Poly(A) polymerase (PAP) (Plasmid copy number protein)	FS43_8510	Poly(A) polymerase (PAP) (Plasmid copy number protein)	FS44_15110	Poly(A) polymerase (PAP) (Plasmid copy number protein)	WQG_14770	Poly(A) polymerase (PAP) (Plasmid copy number protein)
4	FS42_730	Excinuclease ATPase subunit	FS43_760	Excinuclease ATPase subunit	FS44_22380	Excinuclease ATPase subunit	WQG_21870	Excinuclease ATPase subunit
4	FS42_7300	DnaK suppressor protein	FS43_8520	DnaK suppressor protein	FS44_15100	DnaK suppressor protein	WQG_14760	DnaK suppressor protein
4	FS42_7310	Aspartate ammonia-lyase	FS43_8530	Aspartate ammonia-lyase	FS44_15090	Aspartate ammonia-lyase	WQG_14750	Aspartate ammonia-lyase
4	FS42_7320	hypothetical protein	FS43_8540	hypothetical protein	FS44_15080	hypothetical protein	WQG_14740	hypothetical protein
4	FS42_7330	Outer membrane lipoprotein 1	FS43_8550	Outer membrane lipoprotein 1	FS44_15070	Outer membrane lipoprotein 1	WQG_14730	Outer membrane lipoprotein 1
4	FS42_7340	Outer membrane lipoprotein 1	FS43_8560	Outer membrane lipoprotein 1	FS44_15060	Outer membrane lipoprotein 1	WQG_14720	Outer membrane lipoprotein 1
4	FS42_7350	D-methionine transport system permease protein metI	FS43_8570	D-methionine transport system permease protein metI	FS44_15050	D-methionine transport system permease protein metI	WQG_14710	D-methionine transport system permease protein metI
4	FS42_7360	Methionine import ATP-binding protein MetN	FS43_8580	Methionine import ATP-binding protein MetN	FS44_15040	Methionine import ATP-binding protein MetN	WQG_14700	Methionine import ATP-binding protein MetN
4	FS42_7370	Argininosuccinate synthase	FS43_8590	Argininosuccinate synthase	FS44_15030	Argininosuccinate synthase	WQG_14690	Argininosuccinate synthase
4	FS42_7380	hypothetical protein	FS43_8600	hypothetical protein	FS44_15020	hypothetical protein	WQG_14680	hypothetical protein
4	FS42_7390	glycosyltransferase	FS43_8610	glycosyltransferase	FS44_15010	glycosyltransferase	WQG_14670	glycosyltransferase
4	FS42_740	thioesterase	FS43_770	thioesterase	FS44_22370	thioesterase	WQG_21860	Conserved thioesterase
4	FS42_7400	RNA-binding protein	FS43_8620	RNA-binding protein	FS44_15000	RNA-binding protein	WQG_14660	RNA-binding protein
4	FS42_7410	Phosphatidylglycerophosphatase B	FS43_8630	Phosphatidylglycerophosphatase B	FS44_14990	Phosphatidylglycerophosphatase B	WQG_14650	Phosphatidylglycerophosphatase B
4	FS42_7420	N-acetyl-D-glucosamine kinase	FS43_8640	N-acetyl-D-glucosamine kinase	FS44_14980	N-acetyl-D-glucosamine kinase	WQG_14640	N-acetyl-D-glucosamine kinase
4	FS42_7430	Hydroxyacylglutathione hydrolase	FS43_8650	Hydroxyacylglutathione hydrolase	FS44_14970	Hydroxyacylglutathione hydrolase	WQG_14630	Hydroxyacylglutathione hydrolase
4	FS42_7440	hypothetical protein	FS43_8660	hypothetical protein	FS44_14960	hypothetical protein	WQG_14620	hypothetical protein
4	FS42_7450	Undecaprenyl pyrophosphate synthase	FS43_8670	Undecaprenyl pyrophosphate synthase	FS44_14950	Undecaprenyl pyrophosphate synthase	WQG_14610	Undecaprenyl pyrophosphate synthase
4	FS42_7460	Phosphatidate cytidylyltransferase	FS43_8680	Phosphatidate cytidylyltransferase	FS44_14940	Phosphatidate cytidylyltransferase	WQG_14600	Phosphatidate cytidylyltransferase
4	FS42_7470	Protease EcfE	FS43_8690	Protease EcfE	FS44_14930	Protease EcfE	WQG_14590	Protease EcfE
4	FS42_7480	Protective surface antigen D15	FS43_8700	Protective surface antigen D15	FS44_14920	Protective surface antigen D15	WQG_14580	Protective surface antigen D15
4	FS42_7490	Outer membrane protein 26	FS43_8720	Outer membrane protein	FS44_14910	Outer membrane protein 26	WQG_14570	Outer membrane protein 26
4	FS42_750	Outer membrane lipoprotein carrier protein LolA	FS43_780	Outer membrane lipoprotein carrier protein LolA	FS44_22360	Outer membrane lipoprotein carrier protein LolA	WQG_21850	Outer membrane lipoprotein carrier protein LolA
4	FS42_7500	UDP-3-O-acylglucosamine N-acyltransferase	FS43_8730	UDP-3-O-acylglucosamine N-acyltransferase	FS44_14900	UDP-3-O-acylglucosamine N-acyltransferase	WQG_14560	UDP-3-O-acylglucosamine N-acyltransferase
4	FS42_7510	(3R)-hydroxymyristoyl-[acyl-carrier-protein] dehydratase	FS43_8740	(3R)-hydroxymyristoyl-[acyl-carrier-protein] dehydratase	FS44_14890	(3R)-hydroxymyristoyl-[acyl-carrier-protein] dehydratase	WQG_14550	(3R)-hydroxymyristoyl-[acyl-carrier-protein] dehydratase
4	FS42_7520	Acyl-[acyl-carrier-protein]--UDP-N- acetylglucosamine O-acyltransferase	FS43_8750	Acyl-[acyl-carrier-protein]--UDP-N- acetylglucosamine O-acyltransferase	FS44_14880	Acyl-[acyl-carrier-protein]--UDP-N- acetylglucosamine O-acyltransferase	WQG_14540	Acyl-[acyl-carrier-protein]--UDP-N- acetylglucosamine O-acyltransferase
4	FS42_7530	NAD(P) transhydrogenase subunit beta	FS43_8760	NAD(P) transhydrogenase subunit beta	FS44_14860	NAD(P) transhydrogenase subunit beta	WQG_14530	NAD(P) transhydrogenase subunit beta
4	FS42_7540	NAD(P) transhydrogenase alpha subunit	FS43_8770	NAD(P) transhydrogenase alpha subunit	FS44_14850	NAD(P) transhydrogenase alpha subunit	WQG_14520	NAD(P) transhydrogenase alpha subunit
4	FS42_7550	Rod shape-determining protein MreB	FS43_8780	Rod shape-determining protein MreB	FS44_14840	Rod shape-determining protein MreB	WQG_14510	Rod shape-determining protein MreB
4	FS42_7560	MreC protein	FS43_8790	MreC protein	FS44_14830	MreC protein	WQG_14500	MreC protein
4	FS42_7570	Rod shape-determining protein mreD	FS43_8800	Rod shape-determining protein mreD	FS44_14820	Rod shape-determining protein mreD	WQG_14490	Rod shape-determining protein mreD
4	FS42_7580	Replication-associated recombination protein A	FS43_8810	Replication-associated recombination protein A	FS44_14810	Replication-associated recombination protein A	WQG_14480	Replication-associated recombination protein A
4	FS42_7590	ATP-dependent helicase	FS43_8820	ATP-dependent helicase	FS44_14800	ATP-dependent helicase	WQG_14470	ATP-dependent helicase
4	FS42_760	membrane protein	FS43_790	membrane protein	FS44_22350	membrane protein	WQG_21840	Conserved membrane protein
4	FS42_7600	hydrolase	FS43_8840	hydrolase	FS44_14790	hydrolase	WQG_14460	hydrolase
4	FS42_7610	hypothetical protein	FS43_8850	hypothetical protein	FS44_14780	hypothetical protein	WQG_14450	hypothetical protein
4	FS42_7620	Lipid A export ATP-binding/permease protein MsbA	FS43_8860	Lipid A export ATP-binding/permease protein MsbA	FS44_14770	Lipid A export ATP-binding/permease protein MsbA	WQG_14440	Lipid A export ATP-binding/permease protein MsbA
4	FS42_7630	DNA gyrase subunit A	FS43_8870	DNA gyrase subunit A	FS44_14760	DNA gyrase subunit A	WQG_14430	DNA gyrase subunit A
4	FS42_7640	Inner membrane protein YccA	FS43_8880	Inner membrane protein YccA	FS44_14750	Inner membrane protein YccA	WQG_14420	Inner membrane protein YccA
4	FS42_7650	DsrC	FS43_8890	DsrC	FS44_14740	DsrC	WQG_14410	DsrC
4	FS42_7660	ADP-heptose:LPS heptosyltransferase I	FS43_8900	ADP-heptose:LPS heptosyltransferase I	FS44_14730	ADP-heptose:LPS heptosyltransferase I	WQG_14400	ADP-heptose:LPS heptosyltransferase I
4	FS42_7670	Phospho-2-dehydro-3-deoxyheptonate aldolase, Tyr-sensitive	FS43_8910	Phospho-2-dehydro-3-deoxyheptonate aldolase, Tyr-sensitive	FS44_14720	Phospho-2-dehydro-3-deoxyheptonate aldolase, Tyr-sensitive	WQG_14390	Phospho-2-dehydro-3-deoxyheptonate aldolase, Tyr-sensitive
4	FS42_7680	T-protein	FS43_8920	T-protein	FS44_14710	T-protein	WQG_14380	T-protein
4	FS42_7690	Small protein A	FS43_8930	Small protein A	FS44_14700	Small protein A	WQG_14370	Small protein A
4	FS42_770	FAD-binding protein	FS43_800	FAD-binding protein	FS44_22340	FAD-binding protein	WQG_21830	FAD-binding protein
4	FS42_7700	Nucleoid-associated protein	FS43_8940	Nucleoid-associated protein	FS44_14690	Nucleoid-associated protein	WQG_14360	Nucleoid-associated protein

4	FS42_7710	RutC	FS43_8950	RutC	FS44_14680	RutC	WQG_14350	RutC
4	FS42_7720	tRNA (5-methylaminomethyl-2-thiouridylate)-methyltransferase	FS43_8960	tRNA (5-methylaminomethyl-2-thiouridylate)-methyltransferase	FS44_14670	tRNA (5-methylaminomethyl-2-thiouridylate)-methyltransferase	WQG_14340	tRNA (5-methylaminomethyl-2-thiouridylate)-methyltransferase
4	FS42_7730	Der GTPase-activating protein YihI	FS43_8970	Der GTPase-activating protein YihI	FS44_14660	Der GTPase-activating protein YihI	WQG_14330	Der GTPase-activating protein YihI
4	FS42_7740	Orf3	FS43_8980	Orf3	FS44_14650	Orf3	WQG_14320	Orf3
4	FS42_7750	Oxygen-independent coproporphyrinogen-III oxidase	FS43_8990	Oxygen-independent coproporphyrinogen-III oxidase	FS44_14640	Oxygen-independent coproporphyrinogen-III oxidase	WQG_14310	Oxygen-independent coproporphyrinogen-III oxidase
4	FS42_7760	LPS-assembly protein lptD	FS43_9000	LPS-assembly protein lptD	FS44_14630	LPS-assembly protein lptD	WQG_14300	LPS-assembly protein lptD
4	FS42_7770	UPF0208 membrane protein	FS43_9010	UPF0208 membrane protein	FS44_14620	UPF0208 membrane protein	WQG_14290	UPF0208 membrane protein
4	FS42_7780	hypothetical protein	FS43_9020	hypothetical protein	FS44_14610	hypothetical protein	WQG_14280	hypothetical protein
4	FS42_7790	intracellular septation protein A	FS43_9030	intracellular septation protein A	FS44_14600	intracellular septation protein A	WQG_14270	intracellular septation protein A
4	FS42_780	Branched-chain amino acid transport	FS43_810	Branched-chain amino acid transport	FS44_22330	Branched-chain amino acid transport	WQG_21820	Branched-chain amino acid transport
4	FS42_7800	acyl-CoA thioester hydrolase	FS43_9040	acyl-CoA thioester hydrolase	FS44_14590	acyl-CoA thioester hydrolase	WQG_14260	acyl-CoA thioester hydrolase
4	FS42_7810	hypothetical protein	FS43_9050	hypothetical protein	FS44_14580	hypothetical protein	WQG_14250	hypothetical protein
4	FS42_7820	Glutamate-ammonia-ligase adenylyltransferase	FS43_9060	Glutamate-ammonia-ligase adenylyltransferase	FS44_14570	Glutamate-ammonia-ligase adenylyltransferase	WQG_14240	Glutamate-ammonia-ligase adenylyltransferase
4	FS42_7830	hypothetical protein	FS43_9070	hypothetical protein	FS44_14560	hypothetical protein	WQG_14230	hypothetical protein
4	FS42_7840	Periplasmic negative regulator of sigmaE	FS43_9080	Periplasmic negative regulator of sigmaE	FS44_14550	Periplasmic negative regulator of sigmaE	WQG_14220	Periplasmic negative regulator of sigmaE
4	FS42_7850	Sigma-E factor regulatory protein	FS43_9090	Sigma-E factor regulatory protein	FS44_14540	Sigma-E factor regulatory protein	WQG_14210	Sigma-E factor regulatory protein
4	FS42_7860	Long-chain-fatty-acid--CoA ligase	FS43_9100	Long-chain-fatty-acid--CoA ligase	FS44_14530	Long-chain-fatty-acid--CoA ligase	WQG_14200	Long-chain-fatty-acid--CoA ligase
4	FS42_7870	Ribosomal RNA small subunit methyltransferase A	FS43_9110	Ribosomal RNA small subunit methyltransferase A	FS44_14520	Ribosomal RNA small subunit methyltransferase A	WQG_14190	Ribosomal RNA small subunit methyltransferase A
4	FS42_7880	Survival protein SurA-like protein	FS43_9120	Survival protein SurA-like protein	FS44_14510	Survival protein SurA-like protein	WQG_14180	Survival protein SurA-like protein
4	FS42_7890	Bifunctional protein hldE	FS43_9130	Bifunctional protein hldE	FS44_14500	Bifunctional protein hldE	WQG_14170	Bifunctional protein hldE
4	FS42_790	LIV-E family branched chain amino acid exporter, membrane protein	FS43_820	LIV-E family branched chain amino acid exporter, membrane protein	FS44_22320	LIV-E family branched chain amino acid exporter, membrane protein	WQG_21810	LIV-E family branched chain amino acid exporter, membrane protein
4	FS42_7900	CRISPR-associated protein, Cas2	FS43_9150	CRISPR-associated protein, Cas2	FS44_14470	CRISPR-associated protein, Cas2	WQG_14140	CRISPR-associated protein, Cas2
4	FS42_7910	CRISPR-associated protein Cas1	FS43_9160	CRISPR-associated protein Cas1	FS44_8250	CRISPR-associated protein Cas1	WQG_14130	CRISPR-associated protein Cas1
4	FS42_7920	Crispr-associated protein Cas4	FS43_9170	CRISPR-associated protein Cas4	FS44_14450	Crispr-associated protein Cas4	WQG_14120	CRISPR-associated protein Cas4
4	FS42_7930	CRISPR-associated protein, Csd2	FS43_9180	CRISPR-associated protein, Csd2	FS44_14440	CRISPR-associated protein, Csd2	WQG_14110	CRISPR-associated protein, Csd2
4	FS42_7940	CRISPR-associated protein, Csd1	FS43_9190	CRISPR-associated protein, Csd1	FS44_14430	CRISPR-associated protein, Csd1	WQG_14100	CRISPR-associated protein, Csd1
4	FS42_7950	CRISPR-associated protein Cas5	FS43_9200	CRISPR-associated protein Cas5	FS44_14420	CRISPR-associated protein Cas5	WQG_14090	CRISPR-associated protein Cas5
4	FS42_7960	CRISPR-associated helicase Cas3	FS43_9210	CRISPR-associated helicase Cas3	FS44_14410	CRISPR-associated helicase Cas3	WQG_14080	CRISPR-associated helicase Cas3
4	FS42_7970	hypothetical protein	FS43_9220	hypothetical protein	FS44_14400	hypothetical protein	WQG_14070	hypothetical protein
4	FS42_7980	GTPase Der	FS43_9230	GTPase Der	FS44_14390	GTPase Der	WQG_14060	GTPase Der
4	FS42_7990	Glutamyl-tRNA reductase	FS43_9240	Glutamyl-tRNA reductase	FS44_14380	Glutamyl-tRNA reductase	WQG_14050	Glutamyl-tRNA reductase
4	FS42_80	Octanoyltransferase	FS43_90	Octanoyltransferase	FS44_23050	Octanoyltransferase	WQG_22530	Octanoyltransferase
4	FS42_8000	GTP pyrophosphokinase	FS43_9260	GTP pyrophosphokinase	FS44_14370	GTP pyrophosphokinase	WQG_14040	GTP pyrophosphokinase
4	FS42_8010	hypothetical protein	FS43_9270	hypothetical protein	FS44_14360	hypothetical protein	WQG_14030	hypothetical protein
4	FS42_8020	1-deoxy-D-xylulose 5-phosphate reductoisomerase	FS43_9280	1-deoxy-D-xylulose 5-phosphate reductoisomerase	FS44_14350	1-deoxy-D-xylulose 5-phosphate reductoisomerase	WQG_14020	1-deoxy-D-xylulose 5-phosphate reductoisomerase
4	FS42_8030	hypothetical protein	FS43_9290	hypothetical protein	FS44_14340	hypothetical protein	WQG_14010	hypothetical protein
4	FS42_8040	PotE protein	FS43_9310	PotE protein	FS44_14330	PotE protein	WQG_14000	PotE protein
4	FS42_8050	MHT1 protein	FS43_9320	MHT1 protein	FS44_14320	MHT1 protein	WQG_13990	MHT1 protein
4	FS42_8060	S-adenosylmethionine synthase	FS43_9330	S-adenosylmethionine synthase	FS44_14310	S-adenosylmethionine synthase	WQG_13980	S-adenosylmethionine synthase
4	FS42_8070	hypothetical protein	FS43_9340	hypothetical protein	FS44_14300	hypothetical protein	WQG_13970	hypothetical protein
4	FS42_8080	CirA protein	FS43_9360	CirA protein	FS44_14290	CirA protein	WQG_13960	CirA protein
4	FS42_8090	ABC transporter domain protein	FS43_9370	ABC transporter domain protein	FS44_14280	ABC transporter domain protein	WQG_13950	ABC transporter domain protein
4	FS42_8110	Ribosome-recycling factor	FS43_9390	Ribosome-recycling factor	FS44_14260	Ribosome-recycling factor	WQG_13930	Ribosome-recycling factor
4	FS42_8120	Phosphoheptose isomerase	FS43_9400	Phosphoheptose isomerase	FS44_14250	Phosphoheptose isomerase	WQG_13920	Phosphoheptose isomerase
4	FS42_8130	membrane protein	FS43_9410	membrane protein	FS44_14240	membrane protein	WQG_13910	membrane protein
4	FS42_8140	HTH-type transcriptional repressor purR	FS43_9420	HTH-type transcriptional repressor purR	FS44_14230	HTH-type transcriptional repressor purR	WQG_13900	HTH-type transcriptional repressor purR
4	FS42_8150	tRNA 5-methylaminomethyl-2-thiouridine biosynthesis bifunctional protein MnmC	FS43_9430	tRNA 5-methylaminomethyl-2-thiouridine biosynthesis bifunctional protein MnmC	FS44_14220	tRNA 5-methylaminomethyl-2-thiouridine biosynthesis bifunctional protein MnmC	WQG_13890	tRNA 5-methylaminomethyl-2-thiouridine biosynthesis bifunctional protein MnmC
4	FS42_8160	Acyl-CoA dehydrogenase	FS43_9440	Acyl-CoA dehydrogenase	FS44_14210	Acyl-CoA dehydrogenase	WQG_13880	Acyl-CoA dehydrogenase
4	FS42_8170	permease	FS43_9450	permease	FS44_14200	permease	WQG_13870	permease
4	FS42_8180	Cystathionine beta-lyase	FS43_9460	Cystathionine beta-lyase	FS44_14190	Cystathionine beta-lyase	WQG_13860	Cystathionine beta-lyase
4	FS42_8190	Peptide chain release factor 2	FS43_9480	Peptide chain release factor 2	FS44_14180	Peptide chain release factor 2	WQG_13850	Peptide chain release factor 2
4	FS42_8200	hypothetical protein	FS43_9490	hypothetical protein	FS44_14170	hypothetical protein	WQG_13840	hypothetical protein
4	FS42_8260	Phage-related protein	FS43_9550	Phage-related protein	FS44_14160	Phage-related protein	WQG_13780	Phage-related protein
4	FS42_8280	hypothetical protein	FS43_9570	hypothetical protein	FS44_14150	hypothetical protein	WQG_13760	hypothetical protein
4	FS42_8290	hypothetical protein	FS43_9580	hypothetical protein	FS44_14140	hypothetical protein	WQG_13750	hypothetical protein
4	FS42_8300	Magnesium and cobalt efflux protein CorC	FS43_9590	Magnesium and cobalt efflux protein CorC	FS44_14130	Magnesium and cobalt efflux protein CorC	WQG_13740	Magnesium and cobalt efflux protein CorC
4	FS42_8310	Apolipoprotein N-acyltransferase	FS43_9600	Apolipoprotein N-acyltransferase	FS44_14120	Apolipoprotein N-acyltransferase	WQG_13730	Apolipoprotein N-acyltransferase
4	FS42_8320	RNA polymerase sigma-E factor	FS43_9610	RNA polymerase sigma-E factor	FS44_14110	RNA polymerase sigma-E factor	WQG_13720	RNA polymerase sigma-E factor
4	FS42_8330	Sigma-E factor negative regulatory protein	FS43_9620	Sigma-E factor negative regulatory protein	FS44_14100	Sigma-E factor negative regulatory protein	WQG_13710	Sigma-E factor negative regulatory protein
4	FS42_8340	3-deoxy-D-manno-octulosonic acid kinase	FS43_9630	3-deoxy-D-manno-octulosonic acid kinase	FS44_14090	3-deoxy-D-manno-octulosonic acid kinase	WQG_13700	3-deoxy-D-manno-octulosonic acid kinase
4	FS42_8350	hydrolase	FS43_9640	hydrolase	FS44_14080	hydrolase	WQG_13690	hydrolase
4	FS42_8360	tRNA pseudouridine synthase A	FS43_9650	tRNA pseudouridine synthase A	FS44_14070	tRNA pseudouridine synthase A	WQG_13680	tRNA pseudouridine synthase A
4	FS42_8370	Dihydroneopterin triphosphate pyrophosphatase	FS43_9660	Dihydroneopterin triphosphate pyrophosphatase	FS44_14060	Dihydroneopterin triphosphate pyrophosphatase	WQG_13670	Dihydroneopterin triphosphate pyrophosphatase
4	FS42_8380	hypothetical protein	FS43_9670	hypothetical protein	FS44_14050	hypothetical protein	WQG_13660	hypothetical protein
4	FS42_8390	Crossover junction endodeoxyribonuclease RuvC	FS43_9680	Crossover junction endodeoxyribonuclease RuvC	FS44_14040	Crossover junction endodeoxyribonuclease RuvC	WQG_13650	Crossover junction endodeoxyribonuclease RuvC
4	FS42_840	Sensor protein qseC	FS43_870	Sensor protein qseC	FS44_22310	Sensor protein qseC	WQG_21760	Sensor protein qseC
4	FS42_8400	UbiH protein	FS43_9690	UbiH protein	FS44_14030	UbiH protein	WQG_13640	UbiH protein
4	FS42_8410	Selenide, water dikinase	FS43_9700	Selenide, water dikinase	FS44_14020	Selenide, water dikinase	WQG_13630	Selenide, water dikinase
4	FS42_8420	hypothetical protein	FS43_9710	hypothetical protein	FS44_14010	hypothetical protein	WQG_13620	hypothetical protein
4	FS42_8430	Cyclase/dehydrase	FS43_9720	Cyclase/dehydrase	FS44_14000	Cyclase/dehydrase	WQG_13610	Cyclase/dehydrase
4	FS42_8440	UPF0125 protein	FS43_9730	UPF0125 protein	FS44_13990	UPF0125 protein	WQG_13600	Protein rnfH
4	FS42_8450	Methylated-DNA--protein-cysteine S-methyltransferase	FS43_9740	Methylated-DNA--protein-cysteine S-methyltransferase	FS44_13980	Methylated-DNA--protein-cysteine S-methyltransferase	WQG_13590	Methylated-DNA--protein-cysteine S-methyltransferase
4	FS42_8460	transport protein	FS43_9750	transport protein	FS44_13960	transport protein	WQG_13580	transport protein
4	FS42_8470	DNA mismatch repair protein	FS43_9760	DNA mismatch repair protein	FS44_13950	DNA mismatch repair protein	WQG_13570	DNA mismatch repair protein
4	FS42_8480	HTH-type transcriptional regulator iscR	FS43_9770	HTH-type transcriptional regulator iscR	FS44_13940	HTH-type transcriptional regulator iscR	WQG_13560	HTH-type transcriptional regulator iscR
4	FS42_8490	Cysteine desulfurase	FS43_9780	Cysteine desulfurase	FS44_13930	Cysteine desulfurase	WQG_13550	Cysteine desulfurase
4	FS42_850	Transcriptional regulatory protein qseB	FS43_880	Transcriptional regulatory protein qseB	FS44_22300	Transcriptional regulatory protein qseB	WQG_21750	Transcriptional regulatory protein qseB
4	FS42_8500	NifU	FS43_9790	NifU	FS44_13920	NifU	WQG_13540	NifU
4	FS42_8510	Iron-binding protein iscA	FS43_9800	Iron-binding protein iscA	FS44_13910	Iron-binding protein iscA	WQG_13530	Iron-binding protein iscA
4	FS42_8520	Co-chaperone protein hscB	FS43_9810	Co-chaperone protein hscB	FS44_13900	Co-chaperone protein hscB	WQG_13520	Co-chaperone protein hscB
4	FS42_8530	hypothetical protein	FS43_9820	hypothetical protein	FS44_13890	hypothetical protein	WQG_13510	hypothetical protein
4	FS42_8540	Chaperone protein hscA	FS43_9830	Chaperone protein hscA	FS44_13880	Chaperone protein hscA	WQG_13500	Chaperone protein hscA
4	FS42_8550	hypothetical protein	FS43_9840	hypothetical protein	FS44_13870	hypothetical protein	WQG_13490	hypothetical protein
4	FS42_8560	2Fe-2S ferredoxin	FS43_9850	2Fe-2S ferredoxin	FS44_13860	2Fe-2S ferredoxin	WQG_13480	2Fe-2S ferredoxin
4	FS42_8570	Protein iscX	FS43_9860	Protein iscX	FS44_13850	Protein iscX	WQG_13470	Protein iscX
4	FS42_8580	S11 family D-Ala-D-Ala carboxypeptidase	FS43_9880	S11 family D-Ala-D-Ala carboxypeptidase	FS44_13840	S11 family D-Ala-D-Ala carboxypeptidase	WQG_13460	S11 family D-Ala-D-Ala carboxypeptidase
4	FS42_8590	Elongation factor P	FS43_9890	Elongation factor P	FS44_13830	Elongation factor P	WQG_13450	Elongation factor P
4	FS42_860	hypothetical protein	FS43_890	hypothetical protein	FS44_22290	hypothetical protein	WQG_21740	hypothetical protein
4	FS42_8600	hypothetical protein	FS43_9910	hypothetical protein	FS44_13820	hypothetical protein	WQG_13440	hypothetical protein
4	FS42_8610	7-cyano-7-deazaguanine reductase	FS43_9920	7-cyano-7-deazaguanine reductase	FS44_13810	7-cyano-7-deazaguanine reductase	WQG_13430	7-cyano-7-deazaguanine reductase
4	FS42_8620	3-octaprenyl-4-hydroxybenzoate carboxy-lyase	FS43_9930	3-octaprenyl-4-hydroxybenzoate carboxy-lyase	FS44_13800	3-octaprenyl-4-hydroxybenzoate carboxy-lyase	WQG_13420	3-octaprenyl-4-hydroxybenzoate carboxy-lyase
4	FS42_8630	reductase	FS43_9940	reductase	FS44_13790	reductase	WQG_13410	reductase
4	FS42_8640	Glyceraldehyde-3-phosphate dehydrogenase	FS43_9950	Glyceraldehyde-3-phosphate dehydrogenase	FS44_13780	Glyceraldehyde-3-phosphate dehydrogenase	WQG_13400	Glyceraldehyde-3-phosphate dehydrogenase
4	FS42_8650	Farnesyl diphosphate synthase	FS43_9960	Farnesyl diphosphate synthase	FS44_13770	Farnesyl diphosphate synthase	WQG_13390	Farnesyl diphosphate synthase
4	FS42_8660	hypothetical protein	FS43_9970	hypothetical protein	FS44_13760	hypothetical protein	WQG_13380	hypothetical protein
4	FS42_8670	Fe-S protein	FS43_9980	Fe-S protein	FS44_13750	Fe-S protein	WQG_13370	Fe-S protein
4	FS42_8680	Acyl-CoA thioesterase II	FS43_9990	Acyl-CoA thioesterase II	FS44_13740	Acyl-CoA thioesterase II	WQG_13360	Acyl-CoA thioesterase II
4	FS42_8690	tRNA (mo5U34)-methyltransferase	FS43_10000	tRNA (mo5U34)-methyltransferase	FS44_13730	tRNA (mo5U34)-methyltransferase	WQG_13350	tRNA (mo5U34)-methyltransferase
4	FS42_870	hypothetical protein	FS43_900	hypothetical protein	FS44_22280	hypothetical protein	WQG_21730	hypothetical protein
4	FS42_8700	Peroxiredoxin 2 family protein/glutaredoxin	FS43_10010	Peroxiredoxin 2 family protein/glutaredoxin	FS44_13720	Peroxiredoxin 2 family protein/glutaredoxin	WQG_13340	Peroxiredoxin 2 family protein/glutaredoxin
4	FS42_8710	Phosphoenolpyruvate carboxylase	FS43_10020	Phosphoenolpyruvate carboxylase	FS44_13710	Phosphoenolpyruvate carboxylase	WQG_13330	Phosphoenolpyruvate carboxylase
4	FS42_8720	50S ribosomal protein L3 glutamine methyltransferase	FS43_10030	50S ribosomal protein L3 glutamine methyltransferase	FS44_13700	50S ribosomal protein L3 glutamine methyltransferase	WQG_13320	50S ribosomal protein L3 glutamine methyltransferase
4	FS42_8730	UPF0115 protein	FS43_10040	UPF0115 protein	FS44_13690	UPF0115 protein	WQG_13310	UPF0115 protein
4	FS42_8740	integral membrane protein	FS43_10050	integral membrane protein	FS44_13680	integral membrane protein	WQG_13300	integral membrane protein
4	FS42_8750	Aerobic respiration control sensor protein ArcB	FS43_10060	Aerobic respiration control sensor protein ArcB	FS44_13670	Aerobic respiration control sensor protein ArcB	WQG_13290	Aerobic respiration control sensor protein ArcB
4	FS42_8760	Polyribonucleotide nucleotidyltransferase	FS43_10080	Polyribonucleotide nucleotidyltransferase	FS44_13660	Polyribonucleotide nucleotidyltransferase	WQG_13280	Polyribonucleotide nucleotidyltransferase
4	FS42_8770	Lipoprotein NlpI	FS43_10090	Lipoprotein NlpI	FS44_13650	Lipoprotein NlpI	WQG_13270	Lipoprotein NlpI
4	FS42_8780	Cold-shock DEAD box protein-A	FS43_10100	Cold-shock DEAD box protein-A	FS44_13640	Cold-shock DEAD box protein-A	WQG_13260	Cold-shock DEAD box protein-A
4	FS42_8790	tRNA-specific adenosine deaminase	FS43_10110	tRNA-specific adenosine deaminase	FS44_13630	tRNA-specific adenosine deaminase	WQG_13250	tRNA-specific adenosine deaminase
4	FS42_880	hypothetical protein	FS43_910	hypothetical protein	FS44_22270	hypothetical protein	WQG_21720	hypothetical protein
4	FS42_8800	Macrophage infectivity potentiator protein	FS43_10120	Macrophage infectivity potentiator protein	FS44_13570	Macrophage infectivity potentiator protein	WQG_13240	Macrophage infectivity potentiator protein
4	FS42_8810	Dihydrolipoamide succinyltransferase	FS43_10130	Dihydrolipoamide succinyltransferase	FS44_13560	Dihydrolipoamide succinyltransferase	WQG_13230	Dihydrolipoamide succinyltransferase
4	FS42_8820	2-oxoglutarate dehydrogenase E1 component	FS43_10140	2-oxoglutarate dehydrogenase E1 component	FS44_13550	2-oxoglutarate dehydrogenase E1 component	WQG_13220	2-oxoglutarate dehydrogenase E1 component
4	FS42_8830	Serine transporter	FS43_10150	Serine transporter	FS44_13540	Serine transporter	WQG_13210	Serine transporter
4	FS42_8840	L-serine dehydratase	FS43_10160	L-serine dehydratase	FS44_13530	L-serine dehydratase	WQG_13200	L-serine dehydratase
4	FS42_8850	Oxygen-independent coproporphyrinogen-III oxidase-like protein	FS43_10170	Oxygen-independent coproporphyrinogen-III oxidase-like protein	FS44_13520	Oxygen-independent coproporphyrinogen-III oxidase-like protein	WQG_13190	Oxygen-independent coproporphyrinogen-III oxidase-like protein
4	FS42_8860	peroxiredoxin bcp	FS43_10180	peroxiredoxin bcp	FS44_13510	peroxiredoxin bcp	WQG_13180	peroxiredoxin bcp
4	FS42_8870	D,D-heptose 1,7-bisphosphate phosphatase 1	FS43_10190	D,D-heptose 1,7-bisphosphate phosphatase 1	FS44_13500	D,D-heptose 1,7-bisphosphate phosphatase 1	WQG_13170	D,D-heptose 1,7-bisphosphate phosphatase 1
4	FS42_8880	NADPH-dependent 7-cyano-7-deazaguanine reductase	FS43_10200	NADPH-dependent 7-cyano-7-deazaguanine reductase	FS44_13490	NADPH-dependent 7-cyano-7-deazaguanine reductase	WQG_13160	NADPH-dependent 7-cyano-7-deazaguanine reductase
4	FS42_8890	heptosyltransferase II (inner core)	FS43_10210	hypothetical protein	FS44_13480	heptosyltransferase II (inner core)	WQG_13150	heptosyltransferase II (inner core)
4	FS42_890	Inorganic pyrophosphatase	FS43_920	Inorganic pyrophosphatase	FS44_22260	Inorganic pyrophosphatase	WQG_21710	Inorganic pyrophosphatase
4	FS42_8900	hypothetical protein	FS43_10230	hypothetical protein	FS44_13470	hypothetical protein	WQG_13140	hypothetical protein
4	FS42_8910	Lipooligosaccharide D-glycero-D-manno-heptosyltransferase	FS43_10240	Lipooligosaccharide D-glycero-D-manno-heptosyltransferase	FS44_13460	Heptosyltransferase II	WQG_13130	Lipooligosaccharide D-glycero-D-manno-heptosyltransferase
4	FS42_8920	Anhydro-N-acetylmuramic acid kinase	FS43_10250	Anhydro-N-acetylmuramic acid kinase	FS44_13450	Anhydro-N-acetylmuramic acid kinase	WQG_13120	Anhydro-N-acetylmuramic acid kinase
4	FS42_8930	N-acetylmuramic acid 6-phosphate etherase	FS43_10260	N-acetylmuramic acid 6-phosphate etherase	FS44_13440	N-acetylmuramic acid 6-phosphate etherase	WQG_13110	N-acetylmuramic acid 6-phosphate etherase
4	FS42_8940	hypothetical protein	FS43_10270	hypothetical protein	FS44_13430	hypothetical protein	WQG_13100	hypothetical protein
4	FS42_8950	[Protein-PII] uridylyltransferase	FS43_10280	[Protein-PII] uridylyltransferase	FS44_13420	[Protein-PII] uridylyltransferase	WQG_13090	[Protein-PII] uridylyltransferase
4	FS42_8960	Replicative DNA helicase	FS43_10300	Replicative DNA helicase	FS44_13410	Replicative DNA helicase	WQG_13080	Replicative DNA helicase
4	FS42_8970	UPF0745 protein	FS43_10310	UPF0745 protein	FS44_13400	UPF0745 protein	WQG_13070	UPF0745 protein
4	FS42_8980	Dihydrofolate reductase	FS43_10320	Dihydrofolate reductase	FS44_13390	Dihydrofolate reductase	WQG_13060	Dihydrofolate reductase
4	FS42_8990	L-lactate permease	FS43_10330	L-lactate permease	FS44_13380	L-lactate permease	WQG_13050	L-lactate permease
4	FS42_90	Lipoyl synthase	FS43_100	Lipoyl synthase	FS44_23040	Lipoyl synthase	WQG_22520	Lipoyl synthase
4	FS42_900	hypothetical protein	FS43_930	hypothetical protein	FS44_22250	hypothetical protein	WQG_21700	hypothetical protein
4	FS42_9000	Glycolate oxidase	FS43_10340	Glycolate oxidase	FS44_13370	Glycolate oxidase	WQG_13040	Glycolate oxidase
4	FS42_9010	Lactate utilization protein B	FS43_10350	Lactate utilization protein B	FS44_13360	Lactate utilization protein B	WQG_13030	Lactate utilization protein B
4	FS42_9020	hypothetical protein	FS43_10360	hypothetical protein	FS44_13350	hypothetical protein	WQG_13020	hypothetical protein
4	FS42_9030	Trimethylamine-N-oxide reductase	FS43_10370	Trimethylamine-N-oxide reductase	FS44_13340	Trimethylamine-N-oxide reductase	WQG_13010	Trimethylamine-N-oxide reductase
4	FS42_9040	S-adenosylmethionine:tRNA ribosyltransferase-isomerase	FS43_10380	S-adenosylmethionine:tRNA ribosyltransferase-isomerase	FS44_13330	S-adenosylmethionine:tRNA ribosyltransferase-isomerase	WQG_13000	S-adenosylmethionine:tRNA ribosyltransferase-isomerase
4	FS42_9050	hypothetical protein	FS43_10390	hypothetical protein	FS44_13320	hypothetical protein	WQG_12990	hypothetical protein
4	FS42_9060	permease perM	FS43_10400	permease perM	FS44_13310	permease perM	WQG_12980	permease perM
4	FS42_9070	hypothetical protein	FS43_10410	hypothetical protein	FS44_13300	hypothetical protein	WQG_12970	hypothetical protein
4	FS42_9080	hypothetical protein	FS43_10420	hypothetical protein	FS44_13290	hypothetical protein	WQG_12960	hypothetical protein
4	FS42_9090	5,10-methylenetetrahydrofolate reductase	FS43_10430	5,10-methylenetetrahydrofolate reductase	FS44_13280	5,10-methylenetetrahydrofolate reductase	WQG_12950	5,10-methylenetetrahydrofolate reductase
4	FS42_910	Ketol-acid reductoisomerase	FS43_940	Ketol-acid reductoisomerase	FS44_22220	Ketol-acid reductoisomerase	WQG_21690	Ketol-acid reductoisomerase
4	FS42_9100	sulfite reductase (NADPH) alpha subunit	FS43_10440	sulfite reductase (NADPH) alpha subunit	FS44_13270	sulfite reductase (NADPH) alpha subunit	WQG_12940	sulfite reductase (NADPH) alpha subunit
4	FS42_9110	Diguanylate cyclase	FS43_10450	Diguanylate cyclase	FS44_13260	Diguanylate cyclase	WQG_12930	Diguanylate cyclase
4	FS42_9130	[citrate (Pro-3S)-lyase] ligase	FS43_10470	[citrate (Pro-3S)-lyase] ligase	FS44_13250	[citrate (Pro-3S)-lyase] ligase	WQG_12910	[citrate (Pro-3S)-lyase] ligase
4	FS42_9140	Citrate lyase acyl carrier protein	FS43_10480	Citrate lyase acyl carrier protein	FS44_13240	Citrate lyase acyl carrier protein	WQG_12900	Citrate lyase acyl carrier protein
4	FS42_9150	Citrate lyase subunit beta	FS43_10490	Citrate lyase subunit beta	FS44_13230	Citrate lyase subunit beta	WQG_12890	Citrate lyase subunit beta
4	FS42_9160	Citrate lyase alpha chain	FS43_10500	Citrate lyase alpha chain	FS44_13220	Citrate lyase alpha chain	WQG_12880	Citrate lyase alpha chain
4	FS42_9170	Protein CitXG	FS43_10510	Protein CitXG	FS44_13210	Protein CitXG	WQG_12870	Protein CitXG

4	**FS42_9180**	transporter	FS43_10520	transporter	FS44_13200	transporter	WQG_12860	transporter
4	**FS42_9190**	Isocitrate dehydrogenase	FS43_10530	Isocitrate dehydrogenase	FS44_13190	Isocitrate dehydrogenase	WQG_12850	Isocitrate dehydrogenase
4	**FS42_9200**	Aconitate hydratase 2	FS43_10540	Aconitate hydratase 2	FS44_13180	Aconitate hydratase 2	WQG_12840	Aconitate hydratase 2
4	**FS42_9210**	Citrate synthase I	FS43_10550	Citrate synthase I	FS44_13170	Citrate synthase I	WQG_12830	Citrate synthase I
4	**FS42_9220**	UPF0135 protein	FS43_10560	UPF0135 protein	FS44_13160	UPF0135 protein	WQG_12820	UPF0135 protein
4	**FS42_9230**	Macrolide export ATP-binding/permease protein MacB	FS43_10570	Macrolide export ATP-binding/permease protein MacB	FS44_13150	Macrolide export ATP-binding/permease protein MacB	WQG_12810	Macrolide export ATP-binding/permease protein MacB
4	**FS42_9240**	Two-component sensor protein/signal transduction histidine kinase	FS43_10580	Two-component sensor protein/signal transduction histidine kinase	FS44_13140	Two-component sensor protein/signal transduction histidine kinase	WQG_12800	Two-component sensor protein/signal transduction histidine kinase
4	**FS42_9250**	Transcriptional regulatory protein CpxR	FS43_10590	Transcriptional regulatory protein CpxR	FS44_13130	Transcriptional regulatory protein CpxR	WQG_12790	Transcriptional regulatory protein CpxR
4	**FS42_9260**	Nucleoside triphosphate pyrophosphohydrolase	FS43_10600	Nucleoside triphosphate pyrophosphohydrolase	FS44_13120	Nucleoside triphosphate pyrophosphohydrolase	WQG_12780	Nucleoside triphosphate pyrophosphohydrolase
4	**FS42_9270**	Exopolyphosphatase	FS43_10610	Exopolyphosphatase	FS44_13110	Exopolyphosphatase	WQG_12770	Exopolyphosphatase
4	**FS42_9280**	hypothetical protein	FS43_10620	hypothetical protein	FS44_13100	hypothetical protein	WQG_12760	hypothetical protein
4	**FS42_9290**	ATPase component of ABC transporter with duplicated ATPase domains-containing protein	FS43_10630	ATPase component of ABC transporter with duplicated ATPase domains-containing protein	FS44_13090	ATPase component of ABC transporter with duplicated ATPase domains-containing protein	WQG_12750	ATPase component of ABC transporter with duplicated ATPase domains-containing protein
4	**FS42_930**	Pertactin	FS43_960	Pertactin	FS44_22210	hypothetical protein	WQG_21670	Pertactin
4	**FS42_9300**	Ferrous-iron efflux pump FieF	FS43_10640	Ferrous-iron efflux pump FieF	FS44_13080	Ferrous-iron efflux pump FieF	WQG_12740	Ferrous-iron efflux pump FieF
4	**FS42_9310**	hypothetical protein	FS43_10650	hypothetical protein	FS44_13070	hypothetical protein	WQG_12730	hypothetical protein
4	**FS42_9360**	50S ribosomal protein L25	FS43_10700	50S ribosomal protein L25	FS44_13060	50S ribosomal protein L25	WQG_12680	50S ribosomal protein L25
4	**FS42_9370**	hypothetical protein	FS43_10710	hypothetical protein	FS44_13050	hypothetical protein	WQG_12670	hypothetical protein
4	**FS42_9380**	Phosphorylase	FS43_10720	Phosphorylase	FS44_13040	Phosphorylase	WQG_12660	Phosphorylase
4	**FS42_9390**	Anaerobic regulatory protein	FS43_10730	Anaerobic regulatory protein	FS44_13030	Anaerobic regulatory protein	WQG_12650	Anaerobic regulatory protein
4	**FS42_940**	Glutathione S-transferase domain protein	FS43_970	Glutathione S-transferase domain protein	FS44_22200	Glutathione S-transferase domain protein	WQG_21660	Glutathione S-transferase domain protein
4	**FS42_9400**	Thymidine kinase	FS43_10740	Thymidine kinase	FS44_13020	Thymidine kinase	WQG_12640	Thymidine kinase
4	**FS42_9410**	Cell division protein ZapB	FS43_10750	Cell division protein ZapB	FS44_13010	Cell division protein ZapB	WQG_12630	Cell division protein ZapB
4	**FS42_9420**	UPF0382 membrane protein	FS43_10760	UPF0382 membrane protein	FS44_13000	UPF0382 membrane protein	WQG_12620	UPF0382 membrane protein
4	**FS42_9430**	Ribosomal RNA large subunit methyltransferase M	FS43_10770	Ribosomal RNA large subunit methyltransferase M	FS44_12990	Ribosomal RNA large subunit methyltransferase M	WQG_12610	Ribosomal RNA large subunit methyltransferase M
4	**FS42_9440**	Dihydrodipicolinate reductase	FS43_10780	Dihydrodipicolinate reductase	FS44_12980	Dihydrodipicolinate reductase	WQG_12600	Dihydrodipicolinate reductase
4	**FS42_9450**	Cystathionine gamma-synthase	FS43_10790	Cystathionine gamma-synthase	FS44_12970	Cystathionine gamma-synthase	WQG_12590	Cystathionine gamma-synthase
4	**FS42_9460**	tRNA (cmo5U34)-methyltransferase 2	FS43_10810	tRNA (cmo5U34)-methyltransferase 2	FS44_12960	tRNA (cmo5U34)-methyltransferase 2	WQG_12580	tRNA (cmo5U34)-methyltransferase 2
4	**FS42_9470**	hypothetical protein	FS43_10820	hypothetical protein	FS44_12950	hypothetical protein	WQG_12570	hypothetical protein
4	**FS42_9480**	hypothetical protein	FS43_10830	hypothetical protein	FS44_12940	hypothetical protein	WQG_12560	hypothetical protein
4	**FS42_9490**	Protein pmbA	FS43_10840	Protein pmbA	FS44_12930	Protein pmbA	WQG_12550	Protein pmbA
4	**FS42_950**	HTH-type transcriptional regulator gntR	FS43_980	HTH-type transcriptional regulator gntR	FS44_22190	HTH-type transcriptional regulator gntR	WQG_21650	HTH-type transcriptional regulator gntR
4	**FS42_9500**	transport protein	FS43_10850	transport protein	FS44_12920	transport protein	WQG_12540	transport protein
4	**FS42_9510**	hypothetical protein	FS43_10860	hypothetical protein	FS44_12910	hypothetical protein	WQG_12530	hypothetical protein
4	**FS42_9520**	Protein UshA	FS43_10870	Protein UshA	FS44_12900	Protein UshA	WQG_12520	Protein UshA
4	**FS42_9530**	Diacylglycerol kinase	FS43_10880	Diacylglycerol kinase	FS44_12890	Diacylglycerol kinase	WQG_12510	Diacylglycerol kinase
4	**FS42_9540**	Serine/threonine transporter sstT	FS43_10890	Serine/threonine transporter sstT	FS44_12880	Serine/threonine transporter sstT	WQG_12500	Serine/threonine transporter sstT
4	**FS42_9550**	Recombination protein 2	FS43_10900	Recombination protein 2	FS44_12870	Recombination protein 2	WQG_12490	Recombination protein 2
4	**FS42_9560**	DNA polymerase I (POL I)	FS43_10910	DNA polymerase I (POL I)	FS44_12860	DNA polymerase I (POL I)	WQG_12480	DNA polymerase I (POL I)
4	**FS42_9570**	Aspartate aminotransferase	FS43_10920	Aspartate aminotransferase	FS44_12850	Aspartate aminotransferase	WQG_12470	Aspartate aminotransferase
4	**FS42_9580**	acetyltransferase	FS43_10930	acetyltransferase	FS44_12840	acetyltransferase	WQG_12460	acetyltransferase
4	**FS42_9590**	ATPase	FS43_10940	ATPase	FS44_12830	ATPase	WQG_12450	ATPase
4	**FS42_960**	L-seryl-tRNA(Sec) selenium transferase	FS43_990	L-seryl-tRNA(Sec) selenium transferase	FS44_22180	L-seryl-tRNA(Sec) selenium transferase	WQG_21640	L-seryl-tRNA(Sec) selenium transferase
4	**FS42_9600**	hypothetical protein	FS43_10950	hypothetical protein	FS44_12820	hypothetical protein	WQG_12440	hypothetical protein
4	**FS42_9610**	ATP-dependent RNA helicase rhlB	FS43_10960	ATP-dependent RNA helicase rhlB	FS44_12810	ATP-dependent RNA helicase rhlB	WQG_12430	ATP-dependent RNA helicase rhlB
4	**FS42_9620**	Ornithine carbamoyltransferase	FS43_10980	Ornithine carbamoyltransferase	FS44_12800	Ornithine carbamoyltransferase	WQG_12420	Ornithine carbamoyltransferase
4	**FS42_9630**	Serine hydroxymethyltransferase 1	FS43_10990	Serine hydroxymethyltransferase 1	FS44_12790	Serine hydroxymethyltransferase 1	WQG_12410	Serine hydroxymethyltransferase 1
4	**FS42_9640**	hypothetical protein	FS43_11000	hypothetical protein	FS44_12780	hypothetical protein	WQG_12400	hypothetical protein
4	**FS42_9650**	Phosphoglycerate mutase	FS43_11010	Phosphoglycerate mutase	FS44_12770	Phosphoglycerate mutase	WQG_12390	Phosphoglycerate mutase
4	**FS42_9660**	Pseudouridine synthase	FS43_11020	Pseudouridine synthase	FS44_12760	Pseudouridine synthase	WQG_12380	Pseudouridine synthase
4	**FS42_9670**	hypothetical protein	FS43_11030	hypothetical protein	FS44_12750	hypothetical protein	WQG_12370	hypothetical protein
4	**FS42_9680**	Outer membrane protein W	FS43_11040	Outer membrane protein W	FS44_12740	Outer membrane protein W	WQG_12360	Outer membrane protein W
4	**FS42_9690**	GMP synthase	FS43_11050	GMP synthase	FS44_12730	GMP synthase	WQG_12350	GMP synthase
4	**FS42_970**	Pyridoxine/pyridoxamine 5'-phosphate oxidase	FS43_1000	Pyridoxine/pyridoxamine 5'-phosphate oxidase	FS44_22170	Pyridoxine/pyridoxamine 5'-phosphate oxidase	WQG_21630	Pyridoxine/pyridoxamine 5'-phosphate oxidase
4	**FS42_9700**	hypothetical protein	FS43_11060	hypothetical protein	FS44_12720	hypothetical protein	WQG_12340	hypothetical protein
4	**FS42_9710**	Inosine-5'-monophosphate dehydrogenase	FS43_11070	Inosine-5'-monophosphate dehydrogenase	FS44_12710	Inosine-5'-monophosphate dehydrogenase	WQG_12330	Inosine-5'-monophosphate dehydrogenase
4	**FS42_9720**	Glycogen synthase	FS43_11080	Glycogen synthase	FS44_12700	Glycogen synthase	WQG_12320	Glycogen synthase
4	**FS42_9730**	Glucose-1-phosphate adenylyltransferase	FS43_11090	Glucose-1-phosphate adenylyltransferase	FS44_12690	Glucose-1-phosphate adenylyltransferase	WQG_12310	Glucose-1-phosphate adenylyltransferase
4	**FS42_9740**	Glycogen operon protein GlgX	FS43_11100	Glycogen operon protein GlgX	FS44_12680	Glycogen operon protein GlgX	WQG_12300	Glycogen operon protein GlgX
4	**FS42_9750**	1,4-alpha-glucan branching enzyme GlgB	FS43_11110	1,4-alpha-glucan branching enzyme GlgB	FS44_12670	1,4-alpha-glucan branching enzyme GlgB	WQG_12290	1,4-alpha-glucan branching enzyme GlgB
4	**FS42_9760**	FTR1 family iron permease	FS43_11120	FTR1 family iron permease	FS44_12660	FTR1 family iron permease	WQG_12280	FTR1 family iron permease
4	**FS42_9770**	Dyp-type peroxidase	FS43_11130	Dyp-type peroxidase	FS44_12650	Dyp-type peroxidase	WQG_12270	Dyp-type peroxidase
4	**FS42_9780**	periplasmic iron (Fe) transport lipoprotein	FS43_11140	periplasmic iron (Fe) transport lipoprotein	FS44_12640	periplasmic iron (Fe) transport lipoprotein	WQG_12260	periplasmic iron (Fe) transport lipoprotein
4	**FS42_9790**	Glutaminyl-tRNA synthetase	FS43_11150	Glutaminyl-tRNA synthetase	FS44_12630	Glutaminyl-tRNA synthetase	WQG_12250	Glutaminyl-tRNA synthetase
4	**FS42_980**	Fumarate hydratase	FS43_1010	Fumarate hydratase	FS44_22160	Fumarate hydratase	WQG_21620	Fumarate hydratase
4	**FS42_9800**	Fructose-1,6-bisphosphatase class 2	FS43_11160	Fructose-1,6-bisphosphatase class 2	FS44_12620	Fructose-1,6-bisphosphatase class 2	WQG_12240	Fructose-1,6-bisphosphatase class 2
4	**FS42_9810**	Ribosome maturation factor rimP	FS43_11170	Ribosome maturation factor rimP	FS44_12610	Ribosome maturation factor rimP	WQG_12230	Ribosome maturation factor rimP
4	**FS42_9820**	NusA antitermination factor	FS43_11180	NusA antitermination factor	FS44_12600	NusA antitermination factor	WQG_12220	NusA antitermination factor
4	**FS42_9830**	Translation initiation factor IF-2	FS43_11200	Translation initiation factor IF-2	FS44_12590	Translation initiation factor IF-2	WQG_12210	Translation initiation factor IF-2
4	**FS42_9840**	Lysine efflux permease	FS43_11210	Lysine efflux permease	FS44_12580	Lysine efflux permease	WQG_12200	Lysine efflux permease
4	**FS42_9850**	Uridine kinase	FS43_11220	Uridine kinase	FS44_12570	Uridine kinase	WQG_12190	Uridine kinase
4	**FS42_9860**	Deoxycytidine triphosphate deaminase	FS43_11230	Deoxycytidine triphosphate deaminase	FS44_12560	Deoxycytidine triphosphate deaminase	WQG_12180	Deoxycytidine triphosphate deaminase
4	**FS42_9870**	ClpB protein	FS43_11240	ClpB protein	FS44_12550	ClpB protein	WQG_12170	ClpB protein
4	**FS42_9890**	Multifunctional CCA protein	FS43_11260	Multifunctional CCA protein	FS44_12540	Multifunctional CCA protein	WQG_12150	Multifunctional CCA protein
4	**FS42_990**	Smf protein, Rossmann fold nucleotide-binding protein involved in DNA uptake	FS43_1020	Smf protein, Rossmann fold nucleotide-binding protein involved in DNA uptake	FS44_22150	Smf protein, Rossmann fold nucleotide-binding protein involved in DNA uptake	WQG_21610	Smf protein, Rossmann fold nucleotide-binding protein involved in DNA uptake
4	**FS42_9900**	Recombination-associated protein rdgC	FS43_11270	Recombination-associated protein rdgC	FS44_12530	Recombination-associated protein rdgC	WQG_12140	Recombination-associated protein rdgC
4	**FS42_9910**	Pyrroline-5-carboxylate reductase	FS43_11280	Pyrroline-5-carboxylate reductase	FS44_12520	Pyrroline-5-carboxylate reductase	WQG_12130	Pyrroline-5-carboxylate reductase
4	**FS42_9920**	MFS family major facilitator transporter	FS43_11290	MFS family major facilitator transporter	FS44_12510	MFS family major facilitator transporter	WQG_12120	MFS family major facilitator transporter
4	**FS42_9930**	Adenine phosphoribosyltransferase	FS43_11300	Adenine phosphoribosyltransferase	FS44_12500	Adenine phosphoribosyltransferase	WQG_12110	Adenine phosphoribosyltransferase
4	**FS42_9940**	DNA polymerase III subunits gamma and tau	FS43_11310	DNA polymerase III subunits gamma and tau	FS44_12490	DNA polymerase III subunits gamma and tau	WQG_12100	DNA polymerase III subunits gamma and tau
4	**FS42_9950**	ABC transporter ATP-binding protein YadG	FS43_11330	ABC transporter ATP-binding protein YadG	FS44_12480	ABC transporter ATP-binding protein YadG	WQG_12090	ABC transporter ATP-binding protein YadG
4	**FS42_9960**	Inner membrane transport permease yadH	FS43_11340	Inner membrane transport permease yadH	FS44_12470	Inner membrane transport permease yadH	WQG_12080	Inner membrane transport permease yadH
4	**FS42_9970**	2',3'-cyclic-nucleotide 2'-phosphodiesterase/3'-nucleotidase	FS43_11350	2',3'-cyclic-nucleotide 2'-phosphodiesterase/3'-nucleotidase	FS44_12460	2',3'-cyclic-nucleotide 2'-phosphodiesterase/3'-nucleotidase	WQG_12070	2',3'-cyclic-nucleotide 2'-phosphodiesterase/3'-nucleotidase
4	**FS42_9980**	Peptidyl-prolyl cis-trans isomerase B	FS43_11370	Peptidyl-prolyl cis-trans isomerase B	FS44_12450	Peptidyl-prolyl cis-trans isomerase B	WQG_12060	Peptidyl-prolyl cis-trans isomerase B
4	**FS42_9990**	Cysteinyl-tRNA synthetase	FS43_11390	Cysteinyl-tRNA synthetase	FS44_12440	Cysteinyl-tRNA synthetase	WQG_12050	Cysteinyl-tRNA synthetase
3	**FS42_10070**	filamentous hemagglutinin	FS43_11460	filamentous hemagglutinin			WQG_11980	filamentous hemagglutinin
3	**FS42_10080**	transport protein			FS44_12300	transport protein	WQG_11970	transport protein
3	**FS42_10120**	type I restriction enzyme HindVIIP R protein	FS43_11520	type I restriction enzyme HindVIIP R protein			WQG_11930	type I restriction enzyme HindVIIP R protein
3	**FS42_10130**	hypothetical protein	FS43_11530	hypothetical protein			WQG_11920	hypothetical protein
3	**FS42_10140**	hypothetical protein	FS43_11540	hypothetical protein			WQG_11910	hypothetical protein
3	**FS42_10150**	hypothetical protein	FS43_11550	hypothetical protein			WQG_11900	hypothetical protein
3	**FS42_10160**	N-6 DNA methylase	FS43_11560	type I restriction enzyme HindVIIP M protein			WQG_11890	type I restriction enzyme HindVIIP M protein
3	**FS42_10280**	hypothetical protein	FS43_11680	hypothetical protein			WQG_11770	hypothetical protein
3	**FS42_10370**	hypothetical protein	FS43_11770	hypothetical protein			WQG_11680	hypothetical protein
3	**FS42_11300**	hypothetical protein	FS43_12770	hypothetical protein			WQG_10760	hypothetical protein
3	**FS42_11310**	ATPase, predicted ATPase (AAA+ superfamily)	FS43_12780	ATPase, predicted ATPase (AAA+ superfamily)			WQG_10750	ATPase, predicted ATPase (AAA+ superfamily)
3	**FS42_11320**	ATPase, predicted ATPase (AAA+ superfamily)	FS43_12790	ATPase, predicted ATPase (AAA+ superfamily)			WQG_10740	ATPase, predicted ATPase (AAA+ superfamily)
3	**FS42_1140**	hypothetical protein	FS43_1170	hypothetical protein			WQG_21460	hypothetical protein
3	**FS42_11450**	ABC transporter permease protein	FS43_12930	ABC transporter permease protein			WQG_10610	ABC transporter permease protein
3	**FS42_11710**	ATP-dependent RNA helicase HrpA	FS43_13200	ATP-dependent RNA helicase HrpA			WQG_10340	ATP-dependent RNA helicase HrpA
3	**FS42_11720**	Excinuclease ABC subunit B	FS43_13210	Excinuclease ABC subunit B			WQG_10330	Excinuclease ABC subunit B
3	**FS42_11730**	hypothetical protein	FS43_13220	hypothetical protein			WQG_10320	hypothetical protein
3	**FS42_11740**	hypothetical protein	FS43_13230	hypothetical protein			WQG_10310	hypothetical protein
3	**FS42_1190**	transcriptional regulator	FS43_1220	transcriptional regulator			WQG_21410	transcriptional regulator
3	**FS42_1200**	type I restriction-modification system specificity protein	FS43_1230	type I restriction-modification system specificity protein			WQG_21400	type I restriction-modification system specificity protein
3	**FS42_1210**	Filamentation induced by cAMP protein Fic	FS43_1240	Filamentation induced by cAMP protein Fic			WQG_21390	Filamentation induced by cAMP protein Fic
3	**FS42_12140**	IS1016 transposase	FS43_13670	IS1016 transposase			WQG_9910	IS1016 transposase
3	**FS42_12150**	hypothetical protein	FS43_13680	hypothetical protein			WQG_9900	hypothetical protein
3	**FS42_12160**	IS1016 transposase	FS43_13690	IS1016 transposase			WQG_9890	IS1016 transposase
3	**FS42_12530**	hypothetical protein	FS43_14080	hypothetical protein			WQG_9500	hypothetical protein
3	**FS42_13470**	hypothetical protein	FS43_15100	hypothetical protein			WQG_8560	hypothetical protein
3	**FS42_13860**	hypothetical protein	FS43_15480	hypothetical protein			WQG_8180	hypothetical protein
3	**FS42_13870**	hypothetical protein	FS43_15490	hypothetical protein			WQG_8170	hypothetical protein
3	**FS42_13890**	hypothetical protein	FS43_15500	hypothetical protein			WQG_8160	hypothetical protein
3	**FS42_13900**	hypothetical protein	FS43_15510	hypothetical protein			WQG_8150	hypothetical protein
3	**FS42_13910**	hypothetical protein	FS43_15520	hypothetical protein			WQG_8140	hypothetical protein
3	**FS42_13920**	Hemagglutinin/hemolysin-related protein	FS43_15530	Hemagglutinin/hemolysin-related protein			WQG_8130	Hemagglutinin/hemolysin-related protein
3	**FS42_13930**	Hemolysin activation protein	FS43_15550	Hemolysin activation protein			WQG_8120	Hemolysin activation protein
3	**FS42_14050**	transposase-like protein	FS43_15670	transposase-like protein			WQG_8000	transposase-like protein
3	**FS42_14120**	hypothetical protein	FS43_21510	hypothetical protein			WQG_2350	hypothetical protein
3	**FS42_14130**	Molybdopterin oxidoreductase	FS43_15740	Molybdopterin oxidoreductase			WQG_7920	Molybdopterin oxidoreductase
3	**FS42_14140**	hypothetical protein	FS43_15760	hypothetical protein			WQG_7910	hypothetical protein
3	**FS42_14150**	ATPase	FS43_15780	ATPase			WQG_7900	ATPase
3	**FS42_14160**	reductase	FS43_15790	reductase			WQG_7890	reductase
3	**FS42_14170**	reductase	FS43_15800	reductase			WQG_7880	reductase
3	**FS42_14180**	hypothetical protein	FS43_15810	hypothetical protein			WQG_7870	hypothetical protein
3	**FS42_14190**	TtrS	FS43_15820	TtrS			WQG_7860	TtrS
3	**FS42_14200**	reductase	FS43_15830	reductase			WQG_7850	reductase
3	**FS42_14210**	CRISPR-associated protein, Csn1	FS43_15840	CRISPR-associated protein, Csn1			WQG_7840	CRISPR-associated protein, Csn1
3	**FS42_14220**	CRISPR-associated protein Cas1	FS43_15870	CRISPR-associated protein Cas1			WQG_7830	CRISPR-associated protein Cas1
3	**FS42_14230**	CRISPR-associated protein Cas2	FS43_15880	CRISPR-associated protein Cas2			WQG_7820	CRISPR-associated protein Cas2
3	**FS42_14670**	hypothetical protein	FS43_16330	hypothetical protein			WQG_7380	hypothetical protein
3	**FS42_1470**	hypothetical protein	FS43_1510	hypothetical protein			WQG_21120	hypothetical protein
3	**FS42_1480**	hypothetical protein	FS43_1520	hypothetical protein			WQG_21110	hypothetical protein
3	**FS42_1490**	hypothetical protein	FS43_1530	hypothetical protein			WQG_21100	hypothetical protein
3	**FS42_15020**	Autotransporter adhesin	FS43_16690	Autotransporter adhesin			WQG_7030	Autotransporter adhesin
3	**FS42_15030**	hypothetical protein	FS43_16700	hypothetical protein			WQG_7020	hypothetical protein
3	**FS42_15410**	Electron transport complex protein rnfE			FS44_6980	Electron transport complex protein rnfE	WQG_6650	Electron transport complex protein rnfE
3	**FS42_15480**	hypothetical protein	FS43_17150	hypothetical protein			WQG_6580	hypothetical protein
3	**FS42_16330**	hypothetical protein	FS43_18020	hypothetical protein			WQG_5720	hypothetical protein
3	**FS42_16540**	RhtB protein	FS43_18250	RhtB protein			WQG_5500	RhtB protein
3	**FS42_170**	hypothetical protein	FS43_180	hypothetical protein			WQG_22440	hypothetical protein
3	**FS42_17020**	DNA (Cytosine-5-)-methyltransferase	FS43_18750	DNA (Cytosine-5-)-methyltransferase			WQG_5030	DNA (Cytosine-5-)-methyltransferase
3	**FS42_17030**	hypothetical protein	FS43_18760	hypothetical protein			WQG_5020	hypothetical protein
3	**FS42_17050**	hypothetical protein	FS43_18780	hypothetical protein			WQG_5000	hypothetical protein
3	**FS42_1760**	hypothetical protein	FS43_2420	hypothetical protein			WQG_20830	hypothetical protein
3	**FS42_17710**	hypothetical protein	FS43_19560	hypothetical protein			WQG_4250	hypothetical protein
3	**FS42_17720**	Teichoic acid biosynthesis protein	FS43_19570	Teichoic acid biosynthesis protein			WQG_4240	Teichoic acid biosynthesis protein
3	**FS42_17730**	Cps7C	FS43_19590	Cps7C			WQG_4230	Cps7C
3	**FS42_17740**	Glycerol-3-phosphate cytidylyltransferase	FS43_19600	Glycerol-3-phosphate cytidylyltransferase			WQG_4220	Glycerol-3-phosphate cytidylyltransferase
3	**FS42_17750**	Glycerophosphotransferase	FS43_19610	Glycerophosphotransferase			WQG_4210	Glycerophosphotransferase
3	**FS42_1780**	FRG domain protein	FS43_2440	FRG domain protein			WQG_20800	FRG domain protein
3	**FS42_17900**	RhaT protein	FS43_19780	RhaT protein			WQG_4060	RhaT protein

3	FS42_17960	hypothetical protein	FS43_19840	hypothetical protein			WQG_4000	hypothetical protein
3	FS42_17970	hypothetical protein	FS43_19850	hypothetical protein			WQG_3990	hypothetical protein
3	FS42_1800	adenine-specific methylase	FS43_2470	adenine-specific methylase			WQG_20780	adenine-specific methylase
3	FS42_18280	Elongation factor Tu			FS44_4070	Elongation factor Tu	WQG_3670	Elongation factor Tu
3	FS42_18330	hypothetical protein	FS43_20220	hypothetical protein			WQG_3620	hypothetical protein
3	FS42_18870	membrane protein insertion efficiency factor	FS43_20760	membrane protein insertion efficiency factor	FS44_3500	membrane protein insertion efficiency factor		
3	FS42_19000	hypothetical protein	FS43_20880	hypothetical protein			WQG_2960	hypothetical protein
3	FS42_19440	Carboxylesterase type B	FS43_21330	Carboxylesterase type B			WQG_2520	Carboxylesterase type B
3	FS42_19450	hypothetical protein	FS43_21340	hypothetical protein			WQG_2510	hypothetical protein
3	FS42_19460	Hydrolase	FS43_21350	Hydrolase			WQG_2500	Hydrolase
3	FS42_19470	hypothetical protein	FS43_21360	hypothetical protein			WQG_2490	hypothetical protein
3	FS42_19480	Aldo/keto reductase	FS43_21370	Aldo/keto reductase			WQG_2480	Aldo/keto reductase
3	FS42_19490	hypothetical protein	FS43_21380	hypothetical protein			WQG_2470	hypothetical protein
3	FS42_19500	Flavodoxin	FS43_21390	Flavodoxin			WQG_2460	Flavodoxin
3	FS42_19510	LysR protein	FS43_21410	LysR protein			WQG_2450	LysR protein
3	FS42_19830	hypothetical protein	FS43_21730	hypothetical protein			WQG_2130	hypothetical protein
3	FS42_20540	Phosphatidate cytidylyltransferase	FS43_22430	Phosphatidate cytidylyltransferase			WQG_1420	Phosphatidate cytidylyltransferase
3	FS42_20550	Phospholipid/glycerol acyltransferase	FS43_22440	Phospholipid/glycerol acyltransferase			WQG_1410	Phospholipid/glycerol acyltransferase
3	FS42_20560	CDP-diacylglycerol-glycerol-3-phosphate 3-phosphatidyltransferase	FS43_22450	CDP-diacylglycerol-glycerol-3-phosphate 3-phosphatidyltransferase			WQG_1400	CDP-diacylglycerol-glycerol-3-phosphate 3-phosphatidyltransferase
3	FS42_20570	Antibiotic biosynthesis monooxygenase	FS43_22460	Antibiotic biosynthesis monooxygenase			WQG_1390	Antibiotic biosynthesis monooxygenase
3	FS42_20620	hypothetical protein	FS43_22510	hypothetical protein			WQG_1340	hypothetical protein
3	FS42_20820	Quaternary ammonium compound-resistance protein qacE	FS43_22690	Quaternary ammonium compound-resistance protein			WQG_920	hypothetical protein
3	FS42_20930	hypothetical protein	FS43_23210	hypothetical protein			WQG_640	hypothetical protein
3	FS42_21090	Filamentation induced by cAMP protein, Fic	FS43_23370	Filamentation induced by cAMP protein, Fic			WQG_480	Filamentation induced by cAMP protein, Fic
3	FS42_21100	hypothetical protein	FS43_23380	hypothetical protein			WQG_470	hypothetical protein
3	FS42_21140	hydrolase	FS43_23420	hydrolase			WQG_430	hydrolase
3	FS42_21150	reductase	FS43_23430	reductase			WQG_420	reductase
3	FS42_21160	FMN-dependent oxidoreductase, nitrilotriacetate monooxygenase	FS43_23440	FMN-dependent oxidoreductase, nitrilotriacetate monooxygenase			WQG_410	FMN-dependent oxidoreductase, nitrilotriacetate monooxygenase
3	FS42_21180	DSBA oxidoreductase	FS43_23460	DSBA oxidoreductase			WQG_390	DSBA oxidoreductase
3	FS42_21300	hypothetical protein	FS43_23580	hypothetical protein			WQG_270	hypothetical protein
3	FS42_30	Penicillin-binding protein 2	FS43_40	Penicillin-binding protein 2			WQG_22580	Penicillin-binding protein 2
3	FS42_3180	hypothetical protein	FS43_3830	hypothetical protein			WQG_19400	hypothetical protein
3	FS42_3310	Methyltransferase type 11	FS43_3960	Methyltransferase type 11			WQG_19270	Methyltransferase type 11
3	FS42_3320	O antigen biosynthesis rhamnosyltransferase rfbN	FS43_3970	O antigen biosynthesis rhamnosyltransferase rfbN			WQG_19260	O antigen biosynthesis rhamnosyltransferase rfbN
3	FS42_3590	hypothetical protein	FS43_4240	hypothetical protein			WQG_18990	hypothetical protein
3	FS42_4100	hypothetical protein			FS44_18280	hypothetical protein	WQG_18480	hypothetical protein
3	FS42_4310	hypothetical protein	FS43_4960	hypothetical protein			WQG_18270	hypothetical protein
3	FS42_4590	Large exoprotein involved in heme utilizationor adhesion	FS43_5250	Large exoprotein involved in heme utilizationor adhesion			WQG_17990	Large exoprotein involved in heme utilizationor adhesion
3	FS42_4610	hypothetical protein	FS43_5270	hypothetical protein			WQG_17970	hypothetical protein
3	FS42_4620	Cytotoxic domain protein	FS43_5280	Cytotoxic domain protein			WQG_17960	hypothetical protein
3	FS42_4630	hypothetical protein	FS43_5290	hypothetical protein			WQG_17950	hypothetical protein
3	FS42_4640	hypothetical protein	FS43_5310	hypothetical protein			WQG_17940	hypothetical protein
3	FS42_4650	hypothetical protein	FS43_5320	hypothetical protein			WQG_17930	hypothetical protein
3	FS42_4660	hypothetical protein	FS43_5330	hypothetical protein			WQG_17920	hypothetical protein
3	FS42_4670	periplasmic serine protease do/hhoA-like protein	FS43_5340	periplasmic serine protease do/hhoA-like protein			WQG_17910	periplasmic serine protease do/hhoA-like protein
3	FS42_4690	hypothetical protein	FS43_5360	hypothetical protein			WQG_17880	hypothetical protein
3	FS42_4700	transposase-like protein	FS43_5370	transposase-like protein			WQG_17890	transposase-like protein
3	FS42_550	molybdopterin/thiamine biosynthesis protein	FS43_580	molybdopterin/thiamine biosynthesis protein			WQG_22050	molybdopterin/thiamine biosynthesis protein
3	FS42_5600	hypothetical protein	FS43_6770	hypothetical protein			WQG_16480	hypothetical protein
3	FS42_5610	Helix-turn-helix domain protein	FS43_6780	Helix-turn-helix domain protein			WQG_16470	Helix-turn-helix domain protein
3	FS42_5620	HipA N-terminal domain protein	FS43_6790	HipA N-terminal domain protein			WQG_16460	HipA N-terminal domain protein
3	FS42_5630	Helix-turn-helix domain-containing protein	FS43_6800	Helix-turn-helix domain-containing protein			WQG_16450	Helix-turn-helix domain-containing protein
3	FS42_5640	Tellurium resistance protein	FS43_6810	Tellurium resistance protein			WQG_16440	Tellurium resistance protein
3	FS42_5650	hypothetical protein	FS43_6820	hypothetical protein			WQG_16430	hypothetical protein
3	FS42_5660	Tellurium resistance protein	FS43_6830	Tellurium resistance protein			WQG_16420	Tellurium resistance protein
3	FS42_5670	Stress protein	FS43_6840	Stress protein			WQG_16410	Stress protein
3	FS42_5680	hypothetical protein	FS43_6850	hypothetical protein			WQG_16400	hypothetical protein
3	FS42_5990	hypothetical protein			FS44_16480	hypothetical protein	WQG_16070	hypothetical protein
3	FS42_6010	hypothetical protein	FS43_7180	hypothetical protein			WQG_16050	hypothetical protein
3	FS42_7260	hypothetical protein	FS43_8480	hypothetical protein			WQG_14800	hypothetical protein
3	FS42_800	hypothetical protein	FS43_830	hypothetical protein			WQG_21800	hypothetical protein
3	FS42_810	hypothetical protein	FS43_840	hypothetical protein			WQG_21790	hypothetical protein
3	FS42_8100	hypothetical protein	FS43_9380	hypothetical protein			WQG_13940	hypothetical protein
3	FS42_820	Cytosine-specific methyltransferase	FS43_850	Cytosine-specific methyltransferase			WQG_21780	Cytosine-specific methyltransferase
3	FS42_8210	virulence protein	FS43_9500	virulence protein			WQG_13830	Conserved virulence protein
3	FS42_8220	Phosphoprotein phosphatase	FS43_9510	Phosphoprotein phosphatase			WQG_13820	Phosphoprotein phosphatase
3	FS42_8230	NAD-dependent deacetylase sirtuin 5	FS43_9520	NAD-dependent deacetylase sirtuin 5			WQG_13810	NAD-dependent deacetylase sirtuin 5
3	FS42_8240	hypothetical protein	FS43_9530	hypothetical protein			WQG_13800	hypothetical protein
3	FS42_8250	hypothetical protein	FS43_9540	hypothetical protein			WQG_13790	hypothetical protein
3	FS42_8270	hypothetical protein	FS43_9560	hypothetical protein			WQG_13770	hypothetical protein
3	FS42_830	hypothetical protein	FS43_860	hypothetical protein			WQG_21770	hypothetical protein
3	FS42_9120	hypothetical protein	FS43_10460	hypothetical protein			WQG_12920	hypothetical protein
3	FS42_920	hypothetical protein	FS43_950	hypothetical protein			WQG_21680	hypothetical protein
3	FS42_9320	hypothetical protein	FS43_10660	hypothetical protein			WQG_12720	hypothetical protein
3	FS42_9330	hypothetical protein	FS43_10670	hypothetical protein			WQG_12710	hypothetical protein
3	FS42_9350	hypothetical protein	FS43_10690	hypothetical protein			WQG_12690	hypothetical protein
3	FS42_9880	hypothetical protein	FS43_11250	hypothetical protein			WQG_12160	hypothetical protein
3			FS43_10	Protein tldD	FS44_30	Protein tldD	WQG_10	Protein tldD
3			FS43_470	hypothetical protein	FS44_22670	hypothetical protein	WQG_22150	hypothetical protein
3			FS43_6570	hypothetical protein	FS44_17030	hypothetical protein	WQG_16680	hypothetical protein
3			FS43_6580	hypothetical protein	FS44_17020	hypothetical protein	WQG_16670	hypothetical protein
3			FS43_13980	hypothetical protein	FS44_10030	hypothetical protein	WQG_9610	hypothetical protein
3			FS43_16710	hypothetical protein	FS44_7340	hypothetical protein	WQG_7010	hypothetical protein
3			FS43_16750	hypothetical protein	FS44_7300	hypothetical protein	WQG_6970	hypothetical protein
3			FS43_18960	sulfate transporter	FS44_5100	sulfate transporter	WQG_4820	sulfate transporter
3			FS43_22650	hypothetical protein	FS44_2490	hypothetical protein	WQG_880	hypothetical protein
3			FS43_22700	Tetracycline resistance protein, class E	FS44_2460	Tetracycline resistance protein, class E	WQG_1160	Tetracycline resistance protein, class E
3			FS43_22710	Synthetic iM2 transcription activator	FS44_2470	Synthetic iM2 transcription activator	WQG_900	Synthetic iM2 transcription activator
3			FS43_22790	Aminoglycoside 3'-phosphotransferase	FS44_3130	Aminoglycoside 3'-phosphotransferase	WQG_1070	Aminoglycoside 3'-phosphotransferase
3			FS43_22800	Aminoglycoside 6''-phosphotransferase	FS44_3120	Aminoglycoside 6''-phosphotransferase	WQG_1060	Aminoglycoside 6''-phosphotransferase
3			FS43_22810	StrA	FS44_3110	StrA	WQG_1050	StrA
3			FS43_22820	Dihydropteroate synthase type-2	FS44_3040	Dihydropteroate synthase type-2	WQG_1040	Dihydropteroate synthase type-2
3			FS43_22830	ISCR20 transposase	FS44_3090	ISCR20 transposase	WQG_1030	ISCR20 transposase
3			FS43_22840	Type IV secretory pathway VirD2 component	FS44_3080	Type IV secretory pathway VirD2 component	WQG_1020	Type IV secretory pathway VirD2 component
3			FS43_22850	Florfenicol export protein	FS44_3070	Florfenicol export protein	WQG_1010	Florfenicol export protein
3			FS43_22860	Transposase	FS44_3060	Transposase	WQG_1000	Transposase
3			FS43_22910	Beta-lactamase ROB-1	FS44_3200	Beta-lactamase ROB-1	WQG_950	Beta-lactamase ROB-1
3			FS43_22920	Transposase	FS44_3210	Transposase	WQG_940	Transposase
3			FS43_22970	hypothetical protein	FS44_2480	hypothetical protein	WQG_890	hypothetical protein
3			FS43_23120	hypothetical protein	FS44_750	hypothetical protein	WQG_730	hypothetical protein
3			FS43_23130	NlnB protein	FS44_740	NlnB protein	WQG_720	NlnB protein
3			FS43_20220	hypothetical protein	FS44_4020	hypothetical protein	WQG_3620	hypothetical protein
3			FS43_11460	filamentous hemagglutinin	FS44_12310	filamentous hemagglutinin	WQG_11980	filamentous hemagglutinin
2	FS42_10520	hypothetical protein			FS44_11910	hypothetical protein		
2	FS42_12540	hypothetical protein	FS43_14090	hypothetical protein				
2	FS42_15040	hypothetical protein					WQG_7010	hypothetical protein
2	FS42_15170	membrane protein YoaT	FS43_16840	membrane protein YoaT				
2	FS42_17440	hypothetical protein	FS43_19190	hypothetical protein				
2	FS42_18930	hypothetical protein					WQG_3030	hypothetical protein
2	FS42_19610	hypothetical protein			FS44_8280	hypothetical protein		
2	FS42_19900	hypothetical protein			FS44_1600	hypothetical protein		
2	FS42_20050	hypothetical protein			FS44_1450	hypothetical protein		
2	FS42_20160	hypothetical protein	FS43_22060	hypothetical protein				
2	FS42_20510	hypothetical protein					WQG_1450	hypothetical protein
2	FS42_20710	Transcriptional regulator, LysR			FS44_820	Transcriptional regulator, LysR		
2	FS42_20720	Alcohol dehydrogenase zinc-binding domain protein			FS44_810	Alcohol dehydrogenase zinc-binding domain protein		
2	FS42_2360	50S ribosomal protein L36	FS43_3020	50S ribosomal protein L36				
2	FS42_3010	hypothetical protein			FS44_19390	hypothetical protein		
2	FS42_6500	hypothetical protein	FS43_7680	hypothetical protein				
2	FS42_9340	hypothetical protein	FS43_10680	hypothetical protein				
2			FS43_1250	hypothetical protein			WQG_21380	hypothetical protein
2			FS43_1820	Phage integrase	FS44_21410	Phage integrase		
2			FS43_1830	hypothetical protein	FS44_21400	hypothetical protein		
2			FS43_1840	hypothetical protein	FS44_21380	hypothetical protein		
2			FS43_1850	Pyruvate kinase	FS44_21370	Pyruvate kinase		
2			FS43_1860	hypothetical protein	FS44_21360	hypothetical protein		
2			FS43_1870	putative bacteriophage protein	FS44_21350	putative bacteriophage protein		
2			FS43_1880	hypothetical protein	FS44_21340	hypothetical protein		
2			FS43_1890	hypothetical protein	FS44_21330	hypothetical protein		
2			FS43_1900	hypothetical protein	FS44_21320	hypothetical protein		
2			FS43_1910	hypothetical protein	FS44_21310	hypothetical protein		
2			FS43_1920	hypothetical protein	FS44_21290	hypothetical protein		
2			FS43_1940	hypothetical protein	FS44_21280	hypothetical protein		
2			FS43_1950	Transcriptional activator-regulator PrtR/CI repressor	FS44_21270	Transcriptional activator-regulator PrtR/CI repressor		
2			FS43_1960	hypothetical protein	FS44_21260	hypothetical protein		
2			FS43_1970	hypothetical protein	FS44_21250	hypothetical protein		
2			FS43_1980	hypothetical protein	FS44_21240	hypothetical protein		
2			FS43_1990	hypothetical protein	FS44_21230	hypothetical protein		
2			FS43_2000	bacteriophage antirepressor	FS44_21220	bacteriophage antirepressor		
2			FS43_2020	hypothetical protein	FS44_21210	hypothetical protein		
2			FS43_2030	DNA N-6-adenine-methyltransferase	FS44_21200	DNA N-6-adenine-methyltransferase		
2			FS43_2040	hypothetical protein	FS44_21190	hypothetical protein		
2			FS43_2050	putative bacteriophage protein	FS44_21180	putative bacteriophage protein		
2			FS43_2060	hypothetical protein	FS44_21160	hypothetical protein		
2			FS43_2070	hypothetical protein	FS44_21150	hypothetical protein		
2			FS43_2080	hypothetical protein	FS44_21140	hypothetical protein		
2			FS43_2090	Lipoprotein	FS44_21130	Lipoprotein		
2			FS43_2100	hypothetical protein	FS44_21120	hypothetical protein		
2			FS43_2110	Antirepressor protein Ant	FS44_21110	Antirepressor protein Ant		
2			FS43_2120	hypothetical protein	FS44_21100	hypothetical protein		
2			FS43_2130	Hemophilus-specific protein	FS44_21090	Hemophilus-specific protein		
2			FS43_2140	Lysozyme	FS44_21080	Lysozyme		
2			FS43_2150	putative bacteriophage protein	FS44_21070	putative bacteriophage protein		
2			FS43_2160	hypothetical protein	FS44_21060	hypothetical protein		
2			FS43_2170	hypothetical protein	FS44_21050	hypothetical protein		
2			FS43_2180	DNA packaging protein	FS44_21040	DNA packaging protein		
2			FS43_2190	hypothetical protein	FS44_21030	hypothetical protein		

2			F543_2200	Bacteriophage capsid protein	F544_21020	Bacteriophage capsid protein		
2			F543_2210	ATP-dependent protease	F544_20990	ATP-dependent protease		
2			F543_2220	hypothetical protein	F544_20980	hypothetical protein		
2			F543_2230	bacteriophage tail protein	F544_20970	bacteriophage tail protein		
2			F543_2240	bacteriophage tail protein	F544_20960	bacteriophage tail protein		
2			F543_2250	bacteriophage tail protein	F544_20950	bacteriophage tail protein		
2			F543_2260	Phage protein	F544_20940	Phage protein		
2			F543_2270	Phage protein	F544_20930	Phage protein		
2			F543_2280	Phage protein	F544_20920	Phage protein		
2			F543_2290	hypothetical protein	F544_20910	hypothetical protein		
2			F543_2300	Phage tape measure protein	F544_20900	Phage tape measure protein		
2			F543_2310	Phage minor tail protein	F544_20890	Phage minor tail protein		
2			F543_2320	Phage minor tail protein L	F544_20880	Phage minor tail protein L		
2			F543_2330	tail assembly protein	F544_20870	tail assembly protein		
2			F543_2340	Bacteriophage lambda tail assembly I	F544_20860	Bacteriophage lambda tail assembly I		
2			F543_2350	Phage-related protein, tail component	F544_20850	Phage-related protein, tail component		
2			F543_2360	hypothetical protein	F544_20840	hypothetical protein		
2			F543_2370	cytoplasmic protein	F544_20830	cytoplasmic protein		
2			F543_2380	hypothetical protein	F544_20810	hypothetical protein		
2			F543_2390	phosphotransferase	F544_20800	phosphotransferase		
2			F543_2400	hypothetical protein	F544_20790	hypothetical protein		
2			F543_5380	hypothetical protein			WQG_17870	hypothetical protein
2			F543_5660	hypothetical protein			WQG_17590	hypothetical protein
2			F543_5700	D12 class N6 adenine-specific DNA methyltransferase			WQG_17550	D12 class N6 adenine-specific DNA methyltransferase
2			F543_5710	Enoyl-CoA hydratase/carnithine racemase-like protein			WQG_17540	Enoyl-CoA hydratase/carnithine racemase-like protein
2			F543_5720	hypothetical protein			WQG_17530	hypothetical protein
2			F543_5730	hypothetical protein			WQG_17520	hypothetical protein
2			F543_5740	Mu-like prophage FluMu protein gp48			WQG_17510	Mu-like prophage FluMu protein gp48
2			F543_5750	Mu-like prophage FluMu protein gp47			WQG_17500	Mu-like prophage FluMu protein gp47
2			F543_5760	Mu-like prophage FluMu protein gp46			WQG_17490	Mu-like prophage FluMu protein gp46
2			F543_5770	Mu-like prophage FluMu protein gp45			WQG_17480	Mu-like prophage FluMu protein gp45
2			F543_5780	Bacteriophage Mu P			WQG_17470	Bacteriophage Mu P
2			F543_5790	tail protein			WQG_17460	tail protein
2			F543_5800	Mu-like prophage FluMu DNA circularization protein			WQG_17450	Mu-like prophage FluMu DNA circularization protein
2			F543_5810	Monofunctional biosynthetic peptidoglycan transglycosylase			WQG_17440	Monofunctional biosynthetic peptidoglycan transglycosylase
2			F543_5820	Mu-like prophage FluMu protein gp41			WQG_17430	Mu-like prophage FluMu protein gp41
2			F543_5830	Mu-like prophage FluMu tail tube protein			WQG_17420	Mu-like prophage FluMu tail tube protein
2			F543_5840	Mu-like prophage FluMu tail sheath protein			WQG_17410	Mu-like prophage FluMu tail sheath protein
2			F543_5850	Mu-like prophage FluMu protein gp38			WQG_17400	Mu-like prophage FluMu protein gp38
2			F543_5860	Mu-like prophage FluMu protein gp37			WQG_17390	Mu-like prophage FluMu protein gp37
2			F543_5870	Mu-like prophage FluMu protein gp36			WQG_17380	Mu-like prophage FluMu protein gp36
2			F543_5880	hypothetical protein			WQG_17370	hypothetical protein
2			F543_5890	Mu-like prophage FluMu major head subunit			WQG_17360	Mu-like prophage FluMu major head subunit
2			F543_5900	Mu-like prophage FluMu I protein			WQG_17350	Mu-like prophage FluMu I protein
2			F543_5910	Mu-like prophage FluMu G protein 1			WQG_17340	Mu-like prophage FluMu G protein 1
2			F543_5920	hypothetical protein			WQG_17330	hypothetical protein
2			F543_5930	Mu-like prophage FluMu protein gp29			WQG_17320	Mu-like prophage FluMu protein gp29
2			F543_5940	Mu-like prophage FluMu protein gp28			WQG_17310	Mu-like prophage FluMu protein gp28
2			F543_5950	Mu-like phage gp27			WQG_17300	Mu-like phage gp27
2			F543_5960	Ribonuclease R winged-helix domain protein			WQG_17290	Ribonuclease R winged-helix domain protein
2			F543_5970	Mu-like phage gp25			WQG_17280	Mu-like phage gp25
2			F543_5980	hypothetical protein			WQG_17270	hypothetical protein
2			F543_5990	hypothetical protein			WQG_17260	hypothetical protein
2			F543_6000	hypothetical protein			WQG_17250	hypothetical protein
2			F543_6010	hypothetical protein			WQG_17240	hypothetical protein
2			F543_6020	N-acetylmuramoyl-L-alanine amidase			WQG_17230	N-acetylmuramoyl-L-alanine amidase
2			F543_6030	Mor transcription activator			WQG_17220	Mor transcription activator
2			F543_6040	hypothetical protein			WQG_17210	hypothetical protein
2			F543_6050	Mu-like prophage FluMu protein gp16			WQG_17200	Mu-like prophage FluMu protein gp16
2			F543_6060	hypothetical protein			WQG_17190	hypothetical protein
2			F543_6070	hypothetical protein			WQG_17180	hypothetical protein
2			F543_6080	hypothetical protein			WQG_17170	hypothetical protein
2			F543_6090	hypothetical protein			WQG_17160	hypothetical protein
2			F543_6100	Mu-like prophage FluMu host-nuclease inhibitor protein gam			WQG_17150	Mu-like prophage FluMu host-nuclease inhibitor protein gam
2			F543_6110	hypothetical protein			WQG_17140	hypothetical protein
2			F543_6120	hypothetical protein			WQG_17130	hypothetical protein
2			F543_6130	Bifunctional N-acetylglucosamine-1-phosphate uridyltransferase/glucosamine-1-phosphate acetyltransferase			WQG_17120	Bifunctional N-acetylglucosamine-1-phosphate uridyltransferase/glucosamine-1-phosphate acetyltransferase
2			F543_6140	Transposase			WQG_17110	Transposase
2			F543_6150	DNA-binding protein			WQG_17100	DNA-binding protein
2			F543_6160	bacteriophage transcriptional regulator			WQG_17090	bacteriophage transcriptional regulator
2			F543_6170	hypothetical protein			WQG_17080	hypothetical protein
2			F543_7120	hypothetical protein			WQG_16120	hypothetical protein
2			F543_11480	transport protein	F544_12300	transport protein		
2			F543_11890	Transcriptional regulator acrR protein			WQG_11560	Transcriptional regulator acrR protein
2			F543_12120	Dihydrolipoyl dehydrogenase			WQG_11360	Dihydrolipoyl dehydrogenase
2			F543_12490	hypothetical protein			WQG_11030	hypothetical protein
2			F543_13100	Surface antigen (D15), outer membrane protein			WQG_10440	Surface antigen (D15), outer membrane protein
2			F543_14810	ATP-dependent RNA helicase			WQG_8850	ATP-dependent RNA helicase
2			F543_17370	hypothetical protein			WQG_6360	hypothetical protein
2			F543_18230	hypothetical protein			WQG_5520	hypothetical protein
2			F543_19450	S6 family igA-specific metalloendopeptidase			WQG_4360	S6 family igA-specific metalloendopeptidase
2			F543_19460	S6 family igA-specific metalloendopeptidase			WQG_4350	S6 family igA-specific metalloendopeptidase
2			F543_19480	S6 family igA-specific metalloendopeptidase			WQG_4330	S6 family igA-specific metalloendopeptidase
2			F543_19500	Hemophilus-specific protein			WQG_4310	Hemophilus-specific protein
2			F543_19510	EcbG			WQG_4300	EcbG
2			F543_19520	WcnH			WQG_4290	WcnH
2			F543_19530	Capsular polysaccharide synthesis enzyme CapSH			WQG_4280	Capsular polysaccharide synthesis enzyme CapSH
2			F543_19540	BcbE			WQG_4270	BcbE
2			F543_19550	hypothetical protein			WQG_4260	hypothetical protein
2			F543_19580	Cps7C			WQG_4230	Cps7C
2			F543_20160	Elongation factor Tu			WQG_3670	Elongation factor Tu
2			F543_21250	hypothetical protein	F544_1690	hypothetical protein		
2			F543_21800	hypothetical protein			WQG_2060	hypothetical protein
2			F543_21810	hypothetical protein			WQG_2050	hypothetical protein
2			F543_22610	Isochorismatase			WQG_1250	Isochorismatase
2			F543_22620	MerR family transcriptional regulator			WQG_1240	MerR family transcriptional regulator
2			F543_22630	Cation efflux protein			WQG_1230	Cation efflux protein
2			F543_22640	MerR family transcriptional regulator, heavy metal-dependent			WQG_1220	MerR family transcriptional regulator, heavy metal-dependent
2			F543_22660	hypothetical protein			WQG_1200	hypothetical protein
2			F543_22670	Membrane protein			WQG_1190	Membrane protein
2			F543_22680	Transcriptional regulator, MarR			WQG_1180	Transcriptional regulator, MarR
2			F543_22720	hypothetical protein			WQG_1140	hypothetical protein
2			F543_22730	Transposase			WQG_1130	Transposase
2			F543_22740	Insertion sequence domain protein			WQG_1120	Insertion sequence domain protein
2			F543_22750	Macrolide 2'-phosphotransferase			WQG_1110	Macrolide 2'-phosphotransferase
2			F543_22760	Macrolide efflux protein			WQG_1100	Macrolide efflux protein
2			F543_22770	IS6100 transposase			WQG_1090	IS6100 transposase
2			F543_22780	Transposase			WQG_1080	Transposase
2			F543_22870	Hydrolase, alpha/beta domain protein			WQG_960	Hydrolase, alpha/beta domain protein
2			F543_22880	hypothetical protein			WQG_980	hypothetical protein
2			F543_22890	rRNA adenine N-6-methyltransferase			WQG_970	rRNA adenine N-6-methyltransferase
2			F543_22900	Hydrolase, alpha/beta domain protein	F544_3020	Hydrolase, alpha/beta domain protein		
2			F543_22930	Transcriptional regulator, MarR			WQG_930	Transcriptional regulator, MarR
2			F543_22950	Tetracycline resistance protein, class E			WQG_910	Tetracycline resistance protein, class E
2			F543_22990	hypothetical protein			WQG_870	hypothetical protein
2			F543_23000	transcriptional regulator			WQG_860	transcriptional regulator
2			F543_23010	3-oxoacyl-(Acyl-carrier-protein) reductase			WQG_850	3-oxoacyl-(Acyl-carrier-protein) reductase
2			F543_23020	hypothetical protein			WQG_840	hypothetical protein
2			F543_23030	hypothetical protein			WQG_830	hypothetical protein
2			F543_23040	hypothetical protein			WQG_820	hypothetical protein
2			F543_23050	Isochorismatase hydrolase			WQG_810	Isochorismatase hydrolase
2			F543_23060	SpoVR like protein			WQG_800	SpoVR like protein
2			F543_23070	Membrane transporter of cations and cationic drugs			WQG_790	Membrane transporter of cations and cationic drugs
2					F544_790	hypothetical protein	WQG_770	hypothetical protein
2					F544_9970	hypothetical protein	WQG_9550	hypothetical protein
2					F544_10060	KamA	WQG_9630	KamA
2					F544_14490	hypothetical protein	WQG_14160	hypothetical protein
2					F544_20620	hypothetical protein	WQG_20820	hypothetical protein
1	**FS42_10060**	hypothetical protein						
1	**FS42_10670**	hypothetical protein						
1	**FS42_11030**	hypothetical protein						
1	**FS42_13730**	hypothetical protein						
1	**FS42_13880**	FhaB protein						
1	**FS42_15080**	hypothetical protein						
1	**FS42_16580**	hypothetical protein						
1	**FS42_18340**	outer membrane protein						
1	**FS42_2010**	Lipoprotein						
1	**FS42_20730**	AcrR family transcriptional regulator						
1	**FS42_20740**	hypothetical protein						
1	**FS42_20750**	TPR repeat protein						
1	**FS42_20760**	hypothetical protein						
1	**FS42_20770**	hypothetical protein						
1	**FS42_20780**	hypothetical protein						
1	**FS42_20810**	Transcriptional regulator, MarR						
1	**FS42_20850**	hypothetical protein						

1	**FS42_21560**	Protein tldD						
1	**FS42_5400**	hypothetical protein						
1	**FS42_5410**	hypothetical protein						
1			FS43_570	molybdopterin/thiamine biosynthesis protein				
1			FS43_1760	DNA polymerase III subunit beta				
1			FS43_1780	DNA replication and repair protein recF				
1			FS43_1800	hypothetical protein				
1			FS43_1930	hypothetical protein				
1			FS43_2010	hypothetical protein				
1			FS43_2450	Site-specific DNA-methyltransferase				
1			FS43_4750	hypothetical protein				
1			FS43_5060	ModF protein				
1			FS43_5300	hypothetical protein				
1			FS43_7240	RfaL protein				
1			FS43_7910	Copper-transporting P-type ATPase				
1			FS43_7950	iron transport system membrane protein				
1			FS43_8060	Homoserine kinase				
1			FS43_8260	Anthranilate synthase component I				
1			FS43_8710	Outer membrane protein				
1			FS43_8830	ATP-dependent helicase				
1			FS43_9140	hypothetical protein				
1			FS43_9250	hypothetical protein				
1			FS43_9300	hypothetical protein				
1			FS43_9350	CirA protein				
1			FS43_9470	Cystathionine beta-lyase				
1			FS43_9870	S11 family D-Ala-D-Ala carboxypeptidase				
1			FS43_9900	hypothetical protein				
1			FS43_10070	Polyribonucleotide nucleotidyltransferase				
1			FS43_10220	Heptosyltransferase II (inner core)				
1			FS43_10290	Replicative DNA helicase				
1			FS43_10800	tRNA (cmo5U34)-methyltransferase 2				
1			FS43_10970	ATP-dependent RNA helicase rhlB				
1			FS43_11190	Translation initiation factor IF-2				
1			FS43_11320	DNA polymerase III subunits gamma and tau				
1			FS43_11360	2',3'-cyclic-nucleotide 2'-phosphodiesterase/3'-nucleotidase				
1			FS43_11380	Peptidyl-prolyl cis-trans isomerase B				
1			FS43_11470	transport protein				
1			FS43_11990	Potassium efflux protein KefA, small-conductance mechanosensitive channel				
1			FS43_12000	Potassium efflux protein KefA, small-conductance mechanosensitive channel				
1			FS43_12110	Dihydrolipoyllysine-residue acetyltransferase component of pyruvate dehydrogenase complex				
1			FS43_12370	Amidophosphoribosyltransferase				
1			FS43_12450	N5-carboxyaminoimidazole ribonucleotide synthase				
1			FS43_12470	Phosphoserine aminotransferase				
1			FS43_12760	hypothetical protein				
1			FS43_12820	Molybdenum cofactor biosynthesis protein A				
1			FS43_13310	hypothetical protein				
1			FS43_13470	Class II glutamine amidotransferase domain protein				
1			FS43_13510	rRNA maturation factor				
1			FS43_13620	Exonuclease I				
1			FS43_13710	Chromosome partition protein MukB				
1			FS43_14100	Filamentous hemagglutinin outer membrane protein				
1			FS43_14110	Filamentous hemagglutinin outer membrane protein				
1			FS43_14270	Tfp pilus assembly pathway, component PilC				
1			FS43_14430	hypothetical protein				
1			FS43_14460	Signal peptidase I				
1			FS43_14530	Molybdenum transport system permease protein modB				
1			FS43_14770	Aspartyl-tRNA synthetase				
1			FS43_15540	Hemagglutinin/hemolysin-related protein				
1			FS43_15750	Molybdopterin oxidoreductase				
1			FS43_15770	hypothetical protein				
1			FS43_15850	CRISPR-associated protein, Csn1				
1			FS43_15860	hypothetical protein				
1			FS43_15980	PTS system protein, glucitol/sorbitol-specific, IIB component				
1			FS43_16380	Autotransporter adhesin				
1			FS43_17120	electron transport complex protein RnfC				
1			FS43_17550	ABC transport system periplasmic protein				
1			FS43_18050	ABC superfamily ATP binding cassette transporter				
1			FS43_18360	Methionyl-tRNA synthetase				
1			FS43_18630	Ribonuclease R				
1			FS43_18650	Serine acetyltransferase				
1			FS43_19010	hypothetical protein				
1			FS43_19390	Phosphate acyltransferase				
1			FS43_19620	hypothetical protein				
1			FS43_19750	Deoxyguanosinetriphosphate triphosphohydrolase-like protein				
1			FS43_20200	Cytochrome c peroxidase				
1			FS43_20700	NACHT domain protein				
1			FS43_21400	hypothetical protein				
1			FS43_22550	hypothetical protein				
1					FS44_60	Cell filamentation protein Fic-related protein		
1					FS44_100	Two component transcriptional regulator, Fis		
1					FS44_300	RhaT protein		
1					FS44_400	Beta-lactamase-like protein		
1					FS44_420	2-nitropropane dioxygenase NPD		
1					FS44_440	DSBA oxidoreductase		
1					FS44_470	outer membrane protein		
1					FS44_480	outer membrane protein		
1					FS44_880	hypothetical protein		
1					FS44_960	hypothetical protein		
1					FS44_1140	hypothetical protein		
1					FS44_1350	Glutathione S-transferase domain protein		
1					FS44_1610	hypothetical protein		
1					FS44_1630	Lipoprotein		
1					FS44_1700	transposase		
1					FS44_1920	hypothetical protein		
1					FS44_2000	S-formylglutathione hydrolase		
1					FS44_2100	hypothetical protein		
1					FS44_2330	tRNA (uracil-5-)-methyltransferase		
1					FS44_2340	hypothetical protein		
1					FS44_2350	Replicative DNA helicase		
1					FS44_2360	ParB		
1					FS44_2370	hypothetical protein		
1					FS44_2380	hypothetical protein		
1					FS44_2390	hypothetical protein		
1					FS44_2400	hypothetical protein		
1					FS44_2410	Single-stranded DNA-binding protein		
1					FS44_2420	hypothetical protein		
1					FS44_2430	hypothetical protein		
1					FS44_2440	Transposase TnpA, ISL3		
1					FS44_2450	TetG		
1					FS44_2500	Alcohol dehydrogenase zinc-binding domain protein		
1					FS44_2510	Multicopper oxidase type 3		
1					FS44_2520	Metal-binding protein		
1					FS44_2530	hypothetical protein		
1					FS44_2540	Aldo/keto reductase		
1					FS44_2550	Hemophilus-specific protein		
1					FS44_2560	hemophilus-specific protein		
1					FS44_2570	DNA topoisomerase		
1					FS44_2580	hypothetical protein		
1					FS44_2590	hypothetical protein		
1					FS44_2600	hypothetical protein		
1					FS44_2610	heme utilization protein		
1					FS44_2620	hemophilus-specific protein		
1					FS44_2630	hypothetical protein		
1					FS44_2640	lipoprotein		
1					FS44_2650	hypothetical protein		
1					FS44_2660	soluble lytic murein transglycosylase		
1					FS44_2670	hypothetical protein		
1					FS44_2680	VirD4, Type IV secretory pathway, VirD4 component		
1					FS44_2690	hemophilus-specific protein		
1					FS44_2700	membrane protein		
1					FS44_2710	hypothetical protein		
1					FS44_2720	hypothetical protein		
1					FS44_2730	hemophilus-specific protein		
1					FS44_2740	Hemophilus-specific protein		
1					FS44_2750	hypothetical protein		
1					FS44_2760	exported protein		
1					FS44_2770	Integrating conjugative element protein		
1					FS44_2780	hypothetical protein		
1					FS44_2790	lipoprotein		
1					FS44_2800	F pilus assembly Type-IV secretion system for plasmid transfer		
1					FS44_2810	hypothetical protein		
1					FS44_2820	hemophilus-specific protein		
1					FS44_2830	hypothetical protein		
1					FS44_2840	hypothetical protein		
1					FS44_2850	hypothetical protein		
1					FS44_2860	hypothetical protein		
1					FS44_2870	hypothetical protein		
1					FS44_2880	exported protein		
1					FS44_2890	hypothetical protein		
1					FS44_2900	hypothetical protein		
1					FS44_2910	hemophilus-specific protein		
1					FS44_2920	hypothetical protein		
1					FS44_2930	hypothetical protein		
1					FS44_2940	Hemophilus-specific protein		
1					FS44_2950	Antirestriction protein		
1					FS44_2960	hypothetical protein		
1					FS44_2970	type I restriction enzyme M subunit		
1					FS44_2980	Relaxase		
1					FS44_2990	Integrase		
1					FS44_3000	Transposase		
1					FS44_3010	Transposase		
1					FS44_3030	Transposase		
1					FS44_3050	Erythromycin resistance protein		
1					FS44_3140	Peptidase C45 acyl-coenzyme A:6-aminopenicillanic acid acyl-transferase		
1					FS44_3150	MobA		
1					FS44_3170	Transposase		
1					FS44_3180	Acetyl-CoA carboxylase, biotin carboxyl carrier protein		
1					FS44_3190	hypothetical protein		
1					FS44_3220	Phage integrase		
1					FS44_3230	hypothetical protein		

1					F544_3240	hypothetical protein		
1					F544_3370	lipoprotein		
1					F544_3380	lipoprotein		
1					F544_4380	hypothetical protein		
1					F544_4580	Bcs2		
1					F544_4590	hypothetical protein		
1					F544_4600	2-C-methyl-D-erythritol 4-phosphate cytidylyltransferase 1		
1					F544_4610	Alcohol dehydrogenase, zinc-containing		
1					F544_4620	hypothetical protein		
1					F544_5240	hypothetical protein		
1					F544_5290	hypothetical protein		
1					F544_5310	Prophage antirepressor		
1					F544_5320	hypothetical protein		
1					F544_5440	hypothetical protein		
1					F544_5550	Maltose-binding periplasmic protein		
1					F544_5620	4-alpha-glucanotransferase		
1					F544_5650	hypothetical protein		
1					F544_5840	Sel1 domain protein repeat-containing protein		
1					F544_6180	CirA protein		
1					F544_6700	tRNA-dihydrouridine synthase		
1					F544_6850	hypothetical protein		
1					F544_6930	electron transport complex protein RnfC		
1					F544_7350	cell envelope integrity inner membrane protein TolA		
1					F544_7360	Extracellular matrix protein adhesin A		
1					F544_7370	Outer membrane pathogenesis protein		
1					F544_7380	Autotransporter adhesin		
1					F544_7730	hypothetical protein		
1					F544_8170	Metal dependent phosphohydrolase		
1					F544_8180	VirR		
1					F544_8190	DEAD/DEAH box helicase		
1					F544_8200	CRISPR-associated protein, Csd5d		
1					F544_8210	Crispr-associated protein, Csd1		
1					F544_8220	Csd3 family CRISPR-associated protein		
1					F544_8230	Crispr-associated protein Cas4		
1					F544_8240	hypothetical protein		
1					F544_8260	CRISPR-associated protein Cas2		
1					F544_8270	hypothetical protein		
1					F544_8650	tRNA methyltransferase		
1					F544_8830	hypothetical protein		
1					F544_8840	hypothetical protein		
1					F544_9410	hypothetical protein		
1					F544_9420	hypothetical protein		
1					F544_9430	hypothetical protein		
1					F544_9560	hypothetical protein		
1					F544_9570	hypothetical protein		
1					F544_9880	hypothetical protein		
1					F544_9890	hypothetical protein		
1					F544_9900	hypothetical protein		
1					F544_9910	hypothetical protein		
1					F544_9920	hypothetical protein		
1					F544_10720	hypothetical protein		
1					F544_10750	Tetratricopeptide repeat protein		
1					F544_10920	DNA gyrase subunit B		
1					F544_11120	hypothetical protein		
1					F544_11130	hypothetical protein		
1					F544_11400	hypothetical protein		
1					F544_11770	hypothetical protein		
1					F544_12250	Phosphoribosylglycinamide formyltransferase		
1					F544_12260	Type II restriction modification enzyme methyltransferase		
1					F544_12320	filamentous hemagglutinin		
1					F544_12330	hypothetical protein		
1					F544_12340	filamentous hemagglutinin		
1					F544_12350	Exoprotein adhesin or hemolysin		
1					F544_12360	hypothetical protein		
1					F544_12380	hypothetical protein		
1					F544_13580	Acyl-CoA dehydrogenase		
1					F544_13590	ABC-type nitrate/sulfonate/bicarbonate transport system, ATPase		
1					F544_13600	ABC-type nitrate/sulfonate/bicarbonate transport system periplasmic component protein		
1					F544_13610	Binding-protein-dependent transport system inner membrane component		
1					F544_13620	hypothetical protein		
1					F544_13970	Methylated-DNA--protein-cysteine S-methyltransferase		
1					F544_14270	hypothetical protein		
1					F544_14460	CRISPR-associated protein Cas1		
1					F544_14480	hypothetical protein		
1					F544_14870	hypothetical protein		
1					F544_15300	Oligopeptide ABC transporter, periplasmic oligopeptide-binding protein		
1					F544_15530	Autotransporter adhesin		
1					F544_15540	hypothetical protein		
1					F544_16150	Alpha/beta hydrolase		
1					F544_16160	4-oxalocrotonate tautomerase		
1					F544_16430	hypothetical protein		
1					F544_16440	Secretion permease		
1					F544_16460	Secretion permease		
1					F544_16470	hypothetical protein		
1					F544_16640	CP4-6 prophage		
1					F544_16650	hypothetical protein		
1					F544_16810	lysogenic conversion protein		
1					F544_16820	hypothetical protein		
1					F544_16850	hypothetical protein		
1					F544_17600	hypothetical protein		
1					F544_17740	hypothetical protein		
1					F544_17770	hypothetical protein		
1					F544_17790	hypothetical protein		
1					F544_18230	hypothetical protein		
1					F544_18250	hypothetical protein		
1					F544_18690	PTS system, beta-glucoside-specific IIABC subunit		
1					F544_19060	EpsF		
1					F544_19070	Glycosyl transferase, family 2		
1					F544_19080	UDP-galactopyranose mutase		
1					F544_19090	hypothetical protein		
1					F544_19100	hypothetical protein		
1					F544_19940	hypothetical protein		
1					F544_20580	hypothetical protein		
1					F544_20610	hypothetical protein		
1					F544_20630	hypothetical protein		
1					F544_20650	hypothetical protein		
1					F544_20660	Phage DNA primase-like protein		
1					F544_20670	hypothetical protein		
1					F544_20680	phage P4 Ash-like protein		
1					F544_20690	hypothetical protein		
1					F544_20700	Hemophilus-specific protein		
1					F544_20710	anti-repressor protein		
1					F544_20720	Transcriptional regulator, AlpA		
1					F544_20730	hypothetical protein		
1					F544_20740	hypothetical protein		
1					F544_20750	XRE family transcriptional regulator		
1					F544_20760	hypothetical protein		
1					F544_20770	hypothetical protein		
1					F544_20780	Phage integrase family site-specific recombinase		
1					F544_20820	hypothetical protein		
1					F544_21000	antirepressor protein		
1					F544_21010	hypothetical protein		
1					F544_21170	Crossover junction endodeoxyribonuclease rusA		
1					F544_21300	hypothetical protein		
1					F544_21390	hypothetical protein		
1					F544_21830	RNA pyrophosphohydrolase		
1					F544_21930	DNA-damage-inducible protein D		
1					F544_21940	CiaB PROTEIN		
1					F544_21950	hypothetical protein		
1					F544_21990	hypothetical protein		
1					F544_22230	hypothetical protein		
1					F544_22240	hypothetical protein		
1					F544_22510	hypothetical protein		
1					F544_22570	hypothetical protein		
1					F544_22760	hypothetical protein		
1					F544_23100	Penicillin-binding protein 2		
1							WQG_1980	hypothetical protein
1							WQG_3130	hypothetical protein
1							WQG_3810	protein prodIIA
1							WQG_7930	hypothetical protein
1							WQG_12700	hypothetical protein
1							WQG_14150	hypothetical protein
1							WQG_15560	hypothetical protein
1							WQG_16330	hypothetical protein
1							WQG_19580	hypothetical protein
1							WQG_19610	Amino acid ABC transporter, periplasmic amino acid-binding protein
1							WQG_20100	hypothetical protein
